# Dorzagliatin add-on therapy to metformin in patients with type 2 diabetes: a randomized, double-blind, placebo-controlled phase 3 trial

**DOI:** 10.1038/s41591-022-01803-5

**Published:** 2022-05-12

**Authors:** Wenying Yang, Dalong Zhu, Shenglian Gan, Xiaolin Dong, Junping Su, Wenhui Li, Hongwei Jiang, Wenjuan Zhao, Minxiu Yao, Weihong Song, Yibing Lu, Xiuzhen Zhang, Huifang Li, Guixia Wang, Wei Qiu, Guoyue Yuan, Jianhua Ma, Wei Li, Ziling Li, Xiaoyue Wang, Jiao’e Zeng, Zhou Yang, Jingdong Liu, Yongqian Liang, Song Lu, Huili Zhang, Hui Liu, Ping Liu, Kuanlu Fan, Xiaozhen Jiang, Yufeng Li, Qing Su, Tao Ning, Huiwen Tan, Zhenmei An, Zhaoshun Jiang, Lijun Liu, Zunhai Zhou, Qiu Zhang, Xuefeng Li, Zhongyan Shan, Yaoming Xue, Hong Mao, Lixin Shi, Shandong Ye, Xiaomei Zhang, Jiao Sun, Ping Li, Tao Yang, Feng Li, Jingna Lin, Zhinong Zhang, Ying Zhao, Ruonan Li, Xiaohui Guo, Qi Yao, Weiping Lu, Shen Qu, Hongmei Li, Liling Tan, Wenbo Wang, Yongli Yao, Daoxiong Chen, Yulan Li, Jialin Gao, Wen Hu, Xiaoqiang Fei, Tianfeng Wu, Song Dong, Wenlong Jin, Chenzhong Li, Dong Zhao, Bo Feng, Yu Zhao, Yi Zhang, Xiaoying Li, Li Chen

**Affiliations:** 1 China-Japan Friendship Hospital, Beijing, China; 2 Affiliated Drum Tower Hospital, Medical School of Nanjing University, Nanjing, China; 3 The First People’s Hospital of Changde City, Changde, China; 4 Jinan Central Hospital Affiliated to Shandong First Medical University, Jinan, China; 5 Cangzhou People’s Hospital, Cangzhou, China; 6 Peking Union Medical College Hospital, Beijing, China; 7 The First Affiliated Hospital, and College of Clinical Medicine of Henan University of Science and Technology, Luoyang, China; 8 The Affiliated Hospital of Qingdao University, Qingdao, China; 9 Qingdao Central Hospital, Qingdao, China; 10 Chenzhou First People’s Hospital, Chenzhou, China; 11 The Second Affiliated Hospital of Nanjing Medical University, Nanjing, China; 12 Tongji Hospital of Tongji University, Shanghai, China; 13 The First Affiliated Hospital of Kunming Medical University, Kunming, China; 14 The First Bethune Hospital of Jilin University, Changchun, China; 15 Huzhou Central Hospital, Huzhou, China; 16 The Affiliated Hospital of Jiangsu University, Zhenjiang, China; 17 Nanjing First Hospital, Nanjing, China; 18 The Affiliated Hospital of Xuzhou Medical University, Xuzhou, China; 19 Inner Mongolia Baogang Hospital, Baotou, China; 20 The First People’s Hospital of Yue Yang, Yueyang, China; 21 Jingzhou Hospital Affiliated to Yangtze University, Jingzhou, China; 22 Jiangxi Pingxiang People’s Hospital, Pingxiang, China; 23 Jiangxi Provincial People’s Hospital, Nanchang, China; 24 The First People’s Hospital of Shunde, Foshan, China; 25 Chongqing General Hospital, Chongqing, China; 26 Qinghai University Affiliated Hospital, Xining, China; 27 Luoyang Central Hospital, Luoyang, China; 28 General Hospital of Ningxia Medical University, Yinchuan, China; 29 The General Hospital of Xuzhou City Mining Group, Xuzhou, China; 30 Shanghai Pudong New Area People’s Hospital, Shanghai, China; 31 Beijing Friendship Hospital Pinggu Campus, Capital Medical University, Beijing, China; 32 Xinhua Hospital Affiliated to Shanghai Jiao Tong University School of Medicine, Shanghai, China; 33 Baotou Central Hospital, Baotou, China; 34 West China Hospital of Sichuan University, Chengdu, China; 35 The 960th Hospital of the PLA Joint Logistics Support Force, Jinan, China; 36 Yiyang Central Hospital, Yiyang, China; 37 Yangpu Hospital, Tongji University, Shanghai, China; 38 The First Affiliated Hospital of Anhui Medical University, Hefei, China; 39 Taihe Hospital, Hubei University of Medicine, Shiyan, China; 40 The First Hospital of China Medical University, Shenyang, China; 41 Southern Medical University Nanfang Hospital, Guangzhou, China; 42 The Central Hospital of Wuhan, Wuhan, China; 43 The Affiliated Hospital of Guizhou Medical University, Guiyang, China; 44 Anhui Provincial Hospital, Hefei, China; 45 The First Affiliated Hospital of Bengbu Medical College, Bengbu, China; 46 Huadong Hospital Affiliated to Fudan University, Shanghai, China; 47 Jiangsu Province Hospital, Nanjing, China; 48 Jining No. 1 People’s Hospital, Jining, China; 49 Tianjin People’s Hospital, Tianjin, China; 50 The First Hospital of Qiqihar, Qiqihar, China; 51 Jilin Central General Hospital, Jilin, China; 52 Third People’s Hospital of Yunnan Province, Kunming, China; 53 Peking University First Hospital, Beijing, China; 54 Ningbo First Hospital, Ningbo, China; 55 The Affiliated Huai’an No. 1 People’s Hospital of Nanjing Medical University, Huai’an, China; 56 Shanghai Tenth People’s Hospital, Shanghai, China; 57 Emergency General Hospital, Beijing, China; 58 The First Affiliated Hospital of Hainan Medical University, Haikou, China; 59 Peking University Shougang Hospital, Beijing, China; 60 Qinghai Provincial People’s Hospital, Xining, China; 61 Hainan General Hospital, Haikou, China; 62 Liuzhou People’s Hospital, Liuzhou, China; 63 Yijishan Hospital, The First Affiliated Hospital of Wannan Medical University, Wuhu, China; 64 The Second People’s Hospital of Huai’an, Huai’an, China; 65 Jiangsu Taizhou People’s Hospital, Taizhou, China; 66 Zhejiang Hospital, Hangzhou, China; 67 Aerospace Center Hospital, Beijing, China; 68 Yanbian University Hospital, Yanji, China; 69 The Third Affiliated Hospital of Southern Medical University, Guangzhou, China; 70 Beijing Luhe Hospital Affiliated to Capital Medical University, Beijing, China; 71 Shanghai East Hospital, Tongji University, Shanghai, China; 72 Hua Medicine (Shanghai) Ltd., Shanghai, China; 73 Zhongshan Hospital, Fudan University, Shanghai, China

**Keywords:** Type 2 diabetes, Drug development, Type 2 diabetes

## Abstract

Metformin, the first-line therapy for type 2 diabetes (T2D), decreases hepatic glucose production and reduces fasting plasma glucose levels. Dorzagliatin, a dual-acting orally bioavailable glucokinase activator targeting both the pancreas and liver glucokinase, decreases postprandial glucose in patients with T2D. In this randomized, double-blind, placebo-controlled phase 3 trial, the efficacy and safety of dorzagliatin as an add-on therapy to metformin were assessed in patients with T2D who had inadequate glycemic control using metformin alone. Eligible patients with T2D (*n* = 767) were randomly assigned to receive dorzagliatin or placebo (1:1 ratio) as an add-on to metformin (1,500 mg per day) for 24 weeks of double-blind treatment, followed by 28 weeks of open-label treatment with dorzagliatin for all patients. The primary efficacy endpoint was the change in glycated hemoglobin (HbA1c) levels from baseline to week 24, and safety was assessed throughout the trial. At week 24, the least-squares mean change from baseline in HbA1c (95% confidence interval (CI)) was −1.02% (−1.11, −0.93) in the dorzagliatin group and −0.36% (−0.45, −0.26) in the placebo group (estimated treatment difference, −0.66%; 95% CI: −0.79, −0.53; *P* < 0.0001). The incidence of adverse events was similar between groups. There were no severe hypoglycemia events or drug-related serious adverse events in the dorzagliatin and metformin combined therapy group. In patients with T2D who experienced inadequate glycemic control with metformin alone, dorzagliatin resulted in effective glycemic control with good tolerability and safety profile (NCT03141073).

## Abstract (web summary)

The DAWN study is a phase 3 clinical trial that demonstrates effective glycemic control of dorzagliatin as an add-on therapy to metfomin in patients with type 2 diabetes.

## Main

The prevalence of T2D continues to increase worldwide. In 2021, approximately 537 million (one in ten) adults (aged 20–79 years) were living with diabetes worldwide, of whom 90–95% were diagnosed with T2D^1^. As recommended in various guidelines^2,3^, metformin is the preferred first-line therapy for T2D. The anti-hyperglycemic effect of metformin primarily originates from a reduction in hepatic glucose output^4^. The loss of efficacy of metformin monotherapy treatment in patients with T2D is associated with continued deterioration of β-cell function^5,6^. An addition of other anti-diabetic medications to metformin is often required after a few years, and various drugs with different mechanisms of action have emerged as add-on options^2^. However, the ongoing decline in β-cell function remains an unmet need in patients with T2D, some of whom eventually progress to insulin-dependent diabetes and develop additional complications.

Glucokinase is an enzyme that acts as a glucose sensor and plays a key role in glucose homeostasis^7^. Glucokinase regulates glucose-stimulated insulin secretion (GSIS) and intrinsic glucagon release in the pancreas and glucagon-like peptide-1 (GLP-1) secretion in the intestine while modulating hepatic glucose uptake and glycogen synthesis in the liver^8–11^. Decreased glucokinase expression in patients with T2D leads to the loss of glucose sensitivity with increased blood glucose fluctuations^12–14^. Pharmacological activation of glucokinase by small-molecule glucokinase activators (GKAs) showed their effects on the glucose-sensing function of glucokinase through regulating the secretion of hormones such as insulin, glucagon and GLP-1 in response to glucose changes^11,15–18^. These results suggested that GKAs have the potential to improve glucose sensitivity and help maintain glycemic homeostasis in patients with diabetes and might represent an alternative treatment for T2D^19,20^.

The development of GKAs to treat diabetes has experienced a long journey with many challenges. Although several GKAs have progressed into clinical studies, few have been studied beyond phase 2 due to hypoglycemia, hypertriglyceridemia, tachyphylaxis or liver-associated adverse effects^21–26^. Among the initially promising of several GKAs, MK-0941, a dual-acting full GKA, improved glycemic control in early studies but lost its efficacy in a time-dependent manner from 14 to 22 weeks of treatment, resulting in a high incidence of hypoglycemia when added to stable-dose insulin glargine in patients with T2D^26^. The primary explanation for this effect appears to be that MK-0941 allosterically activates glucokinase but also reduces the cooperativity of the enzyme for glucose, as shown by a reduction in the Hill coefficient^27^. This reduction in cooperativity with glucose allows some activation of glucokinase even at low glucose concentrations, leading to a substantial decrease in the threshold of GSIS. A lack of sustained glycemic control in patients with T2D is likely attributable to the continued activation of glucokinase in the face of normoglycemia^26,27^. Thus, it is critical that GKAs maintain the role of glucokinase as a glucose sensor by retaining glucose cooperativity and avoiding inappropriate activation of the enzyme under conditions of low plasma glucose levels.

Dorzagliatin is an orally bioavailable, dual-acting, full GKA that activates both pancreatic and hepatic glucokinase in a glucose-dependent manner. Dorzagliatin regulates the glucose-stimulated secretion of the hormones, such as the insulin in the pancreas and GLP-1 in the intestine, while optimizing the glucose and insulin signals in the liver to control hepatic glucose metabolism in patients with T2D^11,15–17^. A phase 2 clinical study (12-week monotherapy) demonstrated that dorzagliatin improved the glucose disposition index and reduced insulin resistance index homeostasis model assessment-IR (HOMA-IR), and these effects were maintained 1 week after drug withdrawal^17^. Dorzagliatin improved glycemic control when administered orally for 24 weeks at a dose of 75 mg twice daily (BID) to drug-naive patients with T2D as monotherapy in the phase 3 SEED trial^28^ with an improvement in β-cell function index homeostasis model assessment 2-β (HOMA2-β) over placebo. Given that dorzagliatin acts by improving glucose sensitivity and β-cell function, its combination with metformin has the potential to offer synergistic benefits in the treatment of T2D. No drug–drug interactions between dorzagliatin and metformin were observed in the phase 1 clinical study in patients with T2D^29^. Here we report the efficacy and safety of dorzagliatin in combination with metformin in the DAWN trial, a phase 3 clinical trial in patients with T2D who were unable to achieve adequate glycemic control with metformin alone.

## Results

### Patient demographics and clinical characteristics

**Fig. 1:**
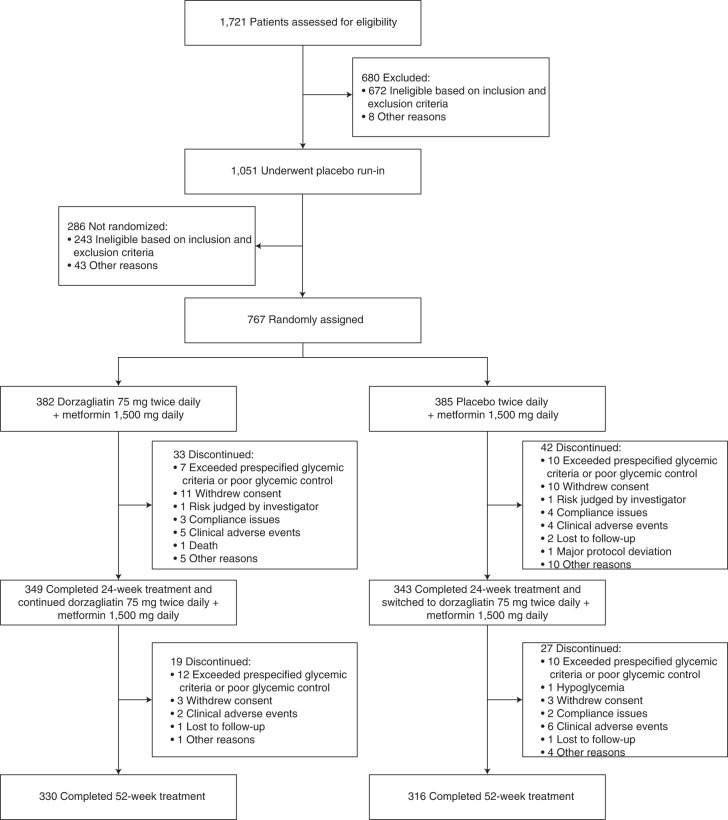
DAWN study patient disposition. DAWN study patient disposition flow diagram for the entire trial. Note that ten patients did not meet the inclusion and exclusion criteria but were included in the run-in period. In total, 767 patients were randomized, and 766 patients who took at least one dose of the study drug were included in the SS. The FAS included 751 patients who took at least one dose of the study drug and had at least one post-treatment measurement of the primary endpoint during the double-blind treatment period. Two patients did not meet the criteria for randomization but were randomized, one of whom was included in the FAS and the other was not, as this patient did not take at least one dose of the study drug.

**Extended Data Fig. 1:**
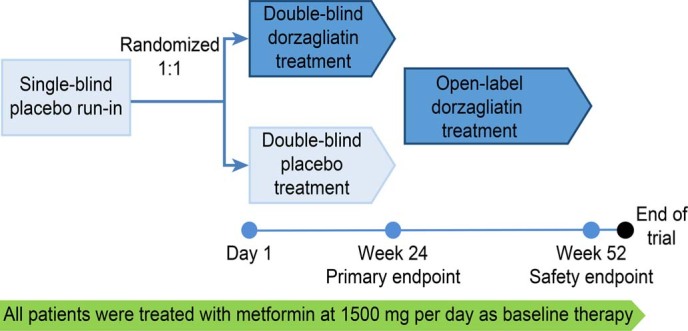
DAWN study design.

Patients were recruited from 11 October 2017 to 30 August 2019, and the last patient visit was conducted on 31 August 2020. Of the 1,721 patients screened, 767 eligible patients were randomly assigned to one of the two treatment groups: 382 patients were assigned to receive dorzagliatin (75 mg BID) and metformin (1,500 mg daily), and 385 patients were assigned to receive placebo and metformin (1,500 mg daily) (Fig. 1). In total, 766 patients took at least one dose of the study drug and were included in the safety analysis set (SS); 751 patients completed at least one post-randomization measurement and were included in the full analysis set (FAS). Among patients who underwent randomization, 692 (90.2%) completed 24 weeks of double-blind treatment (349 patients (91.4%) in the dorzagliatin and metformin group and 343 patients (89.1%) in the placebo and metformin group). All patients were switched to dorzagliatin and metformin after 24 weeks. Subsequently, 646 patients (84.2%) completed 52 weeks of treatment (330 patients (86.4%) in the original dorzagliatin and metformin group and 316 patients (82.1%) in the original placebo and metformin group) (Fig. 1 and Extended Data Fig. 1). Among the 767 randomized patients, 475 (62%) were male and 292 (38%) were female. The average age of the study patients was 54.5 ± 9.6 years, and the average disease duration was 71.5 ± 55.8 months. The patients had an average body mass index (BMI) of 25.9 ± 3.1 kg m^−^^2^ and a mean HbA1c value of 8.3 ± 0.6% at baseline. The demographic and baseline characteristics were similar between the two groups (Table 1).

**Table 1:** Baseline characteristics of the patients

Characteristic	Dorzagliatin and metformin (*n* = 382)	Placebo and metformin (*n* = 385)	Total (*n* = 767)
Age, years	54.6 ± 10.0	54.4 ± 9.2	54.5 ± 9.6
Sex, *n* (%)			
Female	137 (36)	155 (40)	292 (38)
Male	245 (64)	230 (60)	475 (62)
Duration of diabetes, months	73.7 ± 58.5	69.4 ± 53.0	71.5 ± 55.8
Body weight, kg	70.8 ± 11.4	71.5 ± 12.2	71.1 ± 11.8
Body mass index^a^, kg m^−2^	25.7 ± 3.0	26.1 ± 3.2	25.9 ± 3.1
Blood pressure, mmHg			
Systolic	125.7 ± 12.5	125.3 ± 12.3	125.5 ± 12.4
Diastolic	79.9 ± 8.0	80.3 ± 8.1	80.1 ± 8.1
HbA1c, %	8.3 ± 0.6	8.3 ± 0.6	8.3 ± 0.6
FPG, mg dl^−1^	175.9 ± 28.9	179.1 ± 30.0	177.5 ± 29.5
2h-PPG, mg dl^−1^	338.8 ± 55.0	343.4 ± 54.0	341.1 ± 54.6
Fasting C-peptide^b^, ng ml^−1^	1.52 ± 0.55	1.61 ± 0.65	1.56 ± 0.60
HOMA2-β^b^	31.11 ± 11.82	31.43 ± 12.65	31.27 ± 12.24
HOMA2-IR^b^	1.39 ± 0.52	1.49 ± 0.70	1.44 ± 0.62
ALT^c^, U L^−1^	23.0 ± 13.7	24.3 ± 13.9	23.7 ± 13.8
AST^c^, U L^−1^	19.8 ± 8.6	21.0 ± 9.2	20.4 ± 8.9
TBil^c^, μmol L^−1^	10.9 ± 4.9	11.0 ± 4.8	11.0 ± 4.9
TG^c^, mmol L^−1^	1.88 ± 1.30	1.90 ± 1.13	1.89 ± 1.22
TC^c^, mmol L^−1^	4.51 ± 0.89	4.54 ± 0.86	4.53 ± 0.88
LDL-C^c^, mmol L^−1^	2.47 ± 0.78	2.47 ± 0.74	2.47 ± 0.76
HDL-C^c^, mmol L^−1^	1.22 ± 0.28	1.23 ± 0.28	1.22 ± 0.28
Creatinine, µmol L^−1^	67.6 ± 12.92	67.5 ± 13.32	67.5 ± 13.11
Urea nitrogen, mmol L^−1^	5.31 ± 1.316	5.17 ± 1.300	5.24 ± 1.309
Serum uric acid, µmol L^−1^	314.0 ± 69.74	320.5 ± 78.58	317.3 ± 74.33
eGFR^c^, ml/min/1.73 m^−2^	99.6 ± 19.3	98.2 ± 19.0	98.9 ± 19.2

The values are reported as the means ± s.d. Plasma glucose levels were converted to millimoles per liter by dividing the value by 18.

^a^BMI is calculated as weight in kilograms divided by the square of height in meters.

^b^Fasting C-peptide levels, HOMA2-β and HOMA2-IR were calculated in 751 patients included in the FAS.

^c^ALT, AST, TBil, TG, TC, LDL-C, HDL-C, creatinine, urea nitrogen, serum uric acid levels and eGFR were calculated in 766 patients included in the SS population.

### Efficacy outcomes

**Fig. 2:**
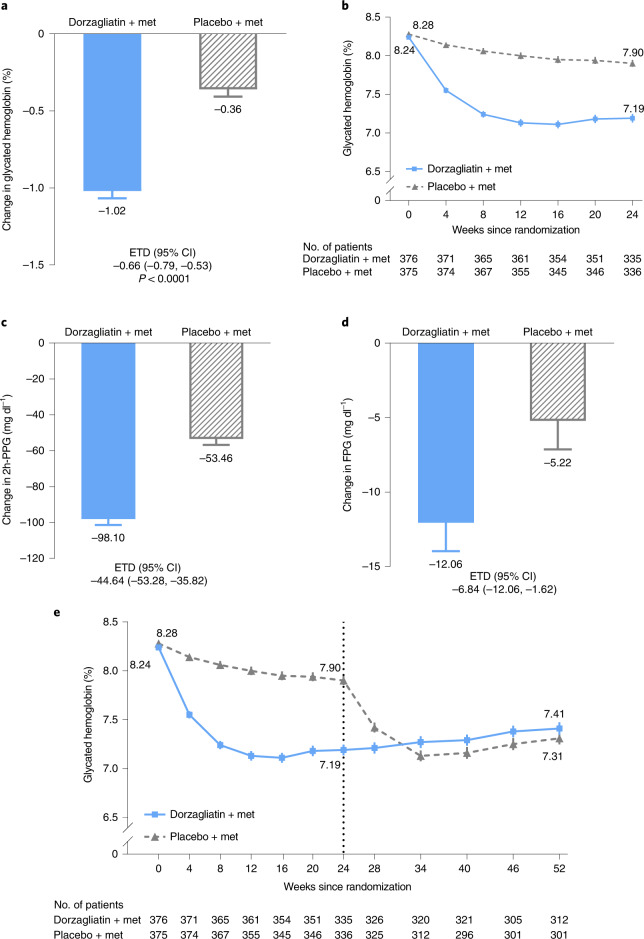
Primary and secondary efficacy endpoints. **a**, The primary endpoint: LS mean changes in the HbA1c level from baseline to week 24 in patients who received either dorzagliatin and metformin or placebo and metformin. The ETD and corresponding 95% CI were estimated using, in the FAS, an MMRM without missing value imputation (dorzagliatin, *n* = 374; placebo, *n* = 374) (*P* < 0.0001). **b**, The mean HbA1c level recorded at each visit over 24 weeks in patients who received either dorzagliatin and metformin or placebo and metformin. **c**, The LS mean change in 2h-PPG levels from baseline. ETD and 95% CI were estimated in the FAS using an MMRM (dorzagliatin, *n* = 360; placebo, *n* = 355). **d**, The LS mean change in FPG from baseline. ETD and 95% CI were estimated in the FAS using an MMRM (dorzagliatin, *n* = 374; placebo, *n* = 374). **e**, The mean HbA1c levels measured at each visit over 52 weeks. The FAS comprised all randomized patients who took at least one dose of the study drug and had at least one post-treatment measurement of the primary endpoint during the double-blind treatment period. All statistical tests were two-sided at a significance level of 0.05, and no adjustments were made for multiplicity. Data in **a**, **c** and **d** are presented as LS mean ± s.e.; data in **b** and **e** are presented as mean ± s.e. Met: metformin.

The study met the primary endpoint. HbA1c levels were reduced from baseline by 1.02% (95% CI: −1.11, −0.93) in the dorzagliatin and metformin group at week 24, whereas the HbA1c decreased by 0.36% (95% CI: −0.45, −0.26) in the placebo and metformin group (estimated treatment difference (ETD), −0.66%; 95% CI: −0.79, −0.53; *P* < 0.0001) (Fig. 2a, Table 2 and Supplementary Table 1).

**Table 2:** Changes in the efficacy endpoints from baseline to weeks 24 and 52

Endpoints	Dorzagliatin and metformin (*n* = 376)	Placebo and metformin (*n* = 375)	ETD, dorzagliatin vs. placebo (95% CI)
HbA1c, %			
Week 4	−0.71 ± 0.02	−0.17 ± 0.02	−0.54 (−0.61, 0.48)
Week 12	−1.13 ± 0.04	−0.28 ± 0.04	−0.85 (−0.96, 0.74)
Week 24	−1.02 ± 0.05	−0.36 ± 0.05	−0.66 (−0.79, −0.53)
*P* value	–	–	<0.0001
Week 52^a^	−0.81 ± 1.059	−0.96 ± 0.971	–
2h-PPG, mg dl^−1^			
Week 12	−99.5 ± 3.1	−45.0 ± 3.1	−54.5 (−62.82, −46.44)
Week 24	−98.1 ± 3.3	−53.5 ± 3.3	−44.6 (−53.28, −35.82)
Week 52^a^	−92.2 ± 69.8	−104.9 ± 72.5	–
FPG, mg dl^−1^			
Week 4	−16.9 ± 1.5	−0.9 ± 1.5	−16.0 (−19.98, −11.88)
Week 12	−15.7 ± 1.6	−4.5 ± 1.6	−11.0 (−15.30, −6.84)
Week 24	−12.1 ± 1.9	−5.2 ± 1.9	−6.8 (−12.06, −1.62)
Week 52^a^	−8.6 ± 37.4	−15.3 ± 35.8	–
HbA1c <7.0%^b^, %			
Week 8	40.6	5.6	-
Week 24	44.4	10.7	7.60 (5.05, 11.42)
Composite endpoint^b^ (HbA1c <7.0% without hypoglycemia and no weight gain), %			
Week 24	26.6	7.7	4.31 (2.75, 6.75)
Fasting insulin, µIU ml^−1^			
Week 24	−0.59 ± 2.76	3.51 ± 2.756	−4.10 (−11.76, 3.56)
HOMA2-β^c^			
Week 24	3.82 ± 0.71	1.40 ± 0.70	2.43 (0.59, 4.26)
HOMA2-IR^c^			
Week 24	−0.17 ± 0.03	−0.09 ± 0.03	−0.08 (−0.15, −0.01)

The values are presented as the estimated LS mean changes ± s.e. from baseline. The LS mean differences and corresponding 95% CIs were estimated in the FAS using an MMRM.

^a^Values are presented as arithmetic means ± s.d. and were calculated using paired *t*-tests for baseline comparisons.

^b^The response rate was estimated in the FAS with the data imputed by LOCF. ETD is presented as the OR and 95% CI and was calculated using logistic regression analysis.

^c^The values are presented as the estimated LS means ± s.e. The LS mean differences in the FAS were calculated using ANCOVA with the data imputed by the LOCF. Data were computed using the HOMA2 Calculator version 2.2.3: https://www.dtu.ox.ac.uk/homacalculator/download.php.

All statistical tests were two-sided at a significance level of 0.05.

The secondary endpoint of the time course of decreases in the HbA1c levels showed that reductions of HbA1c in the dorzagliatin and metformin group started at week 4 and were maintained compared to the placebo group until the end of the 24-week treatment period (Fig. 2b and Table 2). At week 24, 44.4% of the patients in the dorzagliatin and metformin group and 10.7% of the patients in the placebo and metformin group reached HbA1c levels of <7.0% (odds ratio (OR), 7.60; 95% CI: 5.05, 11.42). Around 40.6% of patients in the dorzagliatin and metformin group achieved HbA1c levels of <7.0% at week 8 (versus 5.6% in the placebo and metformin group) and were sustained until week 24 (Table 2).

The change from baseline of fasting plasma glucose (FPG) and 2-hour post-challenge plasma glucose (2h-PPG) at week 24 were also pre-specified secondary endpoints (Fig. 2c,d, Table 2 and Supplementary Tables 2 and 3). At week 24, the reduction in the 2h-PPG level from baseline with dorzagliatin and metformin was greater than that observed for placebo and metformin (ETD, −44.64 mg dl^−1^; 95% CI: −53.28, −35.82) (Fig. 2c, Table 2 and Supplementary Table 3). The FPG levels in the dorzagliatin and metformin group were also reduced further than those in the placebo and metformin group at week 24 (ETD, −6.84 mg dl^−1^; 95% CI: −12.06, −1.62) (Fig. 2d, Table 2 and Supplementary Table 2).

Exploratory endpoints of improvements in HOMA2-β and reduction of HOMA2-IR from baseline to week 24 in the dorzagliatin and metformin group were greater than those in the placebo and metformin group (ETD for HOMA2-β, 2.43; 95% CI: 0.59, 4.26 and ETD for HOMA2-IR, −0.08; 95% CI: −0.15, −0.01) (Table 2).

The reduced HbA1c levels in the dorzagliatin and metformin group were sustained from week 24 to week 52 in the open-label extension arm of the study (Fig. 2e). A similar decrease in HbA1c levels was observed when the placebo and metformin group was crossed over to dorzagliatin and metformin treatment during the open-label extension; HbA1c levels were reduced over the first 4–8 weeks after the crossover and sustained out to week 52 (Fig. 2e and Supplementary Table 4).

Post hoc analyses were performed to explore outcomes other than primary and secondary outcomes. The composite endpoint (HbA1c level of <7.0% without hypoglycemia and no weight gain) was achieved by 26.6% of patients in the dorzagliatin and metformin group, whereas that in the placebo and metformin group was 7.7% at week 24 (OR, 4.31; 95% CI: 2.75, 6.75) (Table 2).

### Safety outcomes

During the 24-week double-blind treatment period, at least one adverse event (AE) was reported in 299 of the 382 patients (78%) in the dorzagliatin and metformin group and in 278 of the 384 patients (72%) in the placebo and metformin group (Table 3). Most AEs were mild and resolved while on treatment, and the investigators considered these AEs unrelated to drug administration (Supplementary Table 7). No clustering of serious adverse events (SAEs) was observed in any organ system (Supplementary Table 8). No severe hypoglycemia events or drug-related SAEs were observed. The AEs that occurred in at least 5% were upper respiratory tract infection, hyperlipidemia, protein present in urine, hyperuricemia and hypertriglyceridemia, in order, which were mostly not related to the investigational drug, as judged by investigators (Table 3). No significant differences in the type and severity of AEs were observed between the two groups (Table 3). The incidence of drug-related AEs, as assessed by the investigators, was low. During the open-label treatment period, the type and incidence rate of both AEs and SAEs remained the same as those that occurred during the double-blind treatment period (Supplementary Table 7).

**Table 3:** AEs and hypoglycemic events recorded during the 24-week double-blind treatment period

Event	Dorzagliatin and metformin (*n* = 382)	Placebo and metformin (*n* = 384)
	No. of patients (%)	No. of patients (%)
Any AEs	299 (78)	278 (72)
AEs related to dorzagliatin#	53 (14)	36 (9)
AEs leading to drug discontinuation^a^	1 (0.3)	3 (0.8)
AEs in ≥5% of patients^b^		
Upper respiratory tract infection	54 (14)	52 (14)
Related to the study drug^c^	0	0
Hyperlipidemia	52 (14)	33 (9)
Related to the study drug^c^	2 (0.5)	1 (0.3)
Protein present in urine	30 (8)	30 (8)
Related to the study drug^c^	0	3 (0.8)
Hyperuricemia	37 (10)	14 (4)
Related to the study drug^c^	3 (0.8)	0
Hypertriglyceridemia	21 (6)	11 (3)
Related to the study drug^c^	7 (2)	2 (0.5)
Any SAEs	19 (5)	17 (4)
SAEs leading to drug discontinuation^d^	3 (0.8)	4 (1.0)
SAEs related to the study drug	0	0
Any hypoglycemia		
Severe hypoglycemia^e^	0	0
Clinically significant hypoglycemia (blood glucose level <54 mg dl^−1^)	3 (0.8)	0

AEs and SAEs that occurred during the 24-week double-blind treatment period among the patients in the safety population are included in the table and presented with their preferred terms based on the *Medical Dictionary for Regulatory Activities*, version 23.0 (ref. ^33^). The data were obtained from patients who received at least one dose of a study drug and included events that occurred during treatment or within 7 days after the last receipt of a study drug.

#Possibly or very likely related to dorzagliatin, as assessed by the investigators.

^a^SAEs that led to drug discontinuation are not included.

^b^AEs in at least 5% of the patients in the dorzagliatin and metformin group or the placebo and metformin group are listed in the table.

^c^AEs in at least 5% of the patients related to dorzagliatin were defined as AEs and deemed by the investigators to be very likely or probably related to dorzagliatin or placebo.

^d^During the 24-week double-blind treatment period, one patient in the dorzagliatin and metformin group died (confirmed by the external drug safety committee as sudden death). The patient had T2D and established cardiovascular disease, and the event was considered unlikely to be related to dorzagliatin by both the investigator and the sponsor.

^e^Severe hypoglycemia is defined as severe cognitive impairment requiring assistance from another person for recovery.

Clinically meaningful hypoglycemia (that is, blood glucose level <54 mg dl^−1^) was reported in three (0.8%) of the 382 patients in the dorzagliatin and metformin group during the 24-week double-blind period and in one (0.1%) of the 692 patients during the open-label treatment period (Supplementary Table 7), whereas zero was reported in the placebo and metformin group during either the 24-week double-blind or the open-label treatment period (Table 3 and Supplementary Table 7).

During the double-blind treatment period, BMI decreased, and no significant differences were observed between the two groups. No significant differences in blood pressure were observed between the two groups. All laboratory indicators related to liver and kidney function were within the normal range during the study. Regarding blood lipid levels, the total cholesterol (TC), low-density lipoprotein cholesterol (LDL-C) and high-density lipoprotein cholesterol (HDL-C) levels were within the normal range during the study, and triglyceride (TG) levels showed slight increases at week 24 without further changes over the 52 weeks of treatment (Table 4). The incidence of hyperlipidemia AEs was 14% in the dorzagliatin and metformin group and 9% in the placebo and metformin group. Only two cases in the dorzagliatin and metformin group and one case in the placebo and metformin group of hyperlipidemia AEs were judged by investigators to be related to the investigational product (Table 3).

**Table 4:** Changes in selected laboratory results and vital signs

	Dorzagliatin and metformin (*n* = 382)		Placebo and metformin (*n* = 384)	
	Week 24	Week 52	Week 24	Week 52
Body weight, kg	−0.19 ± 2.27	−0.50 ± 2.02	−0.45 ± 2.16	−0.30 ± 2.22
BMI, kg m^−2^	−0.07 ± 0.82	−0.18 ± 0.74	−0.17 ± 0.79	−0.11 ± 0.84
Systolic blood pressure, mmHg	−0.18 ± 12.18	−0.20 ± 12.94	−1.20 ± 12.64	2.02 ± 12.52
Diastolic blood pressure, mmHg	0.16 ± 8.47	0.00 ± 9.08	−1.42 ± 7.93	−0.08 ± 8.00
TG, mmol L^−1^	0.41 ± 1.40	0.40 ± 1.64	0.03 ± 0.97	0.59 ± 1.83
TC, mmol L^−1^	0.11 ± 0.70	0.18 ± 0.77	0.01 ± 0.69	0.17 ± 0.68
LDL-C, mmol L^−1^	−0.09 ± 0.57	−0.03 ± 0.62	−0.01 ± 0.59	−0.02 ± 0.56
HDL-C, mmol L^−1^	0.01 ± 0.17	0.04 ± 0.16	0.00 ± 0.17	0.02 ± 0.17
ALT, U L^−1^	5.4 ± 12.2	3.7 ± 12.9	0.1 ± 15.7	5.0 ± 16.0
AST, U L^−1^	4.8 ± 8.5	3.8 ± 9.1	0.0 ± 8.7	5.1 ± 13.0
TBil, μmol L^−1^	−1.0 ± 3.8	−0.6 ± 3.9	−0.3 ± 3.5	−1.0 ± 3.4
Creatinine, µmol L^−1^	0.3 ± 7.13	1.9 ± 7.47	0.3 ± 7.02	1.4 ± 6.75
Urea nitrogen, mmol L^−1^	−0.03 ± 1.331	−0.02 ± 1.255	−0.06 ± 1.269	0.03 ± 1.240
Serum uric acid, µmol L^−1^	29.9 ± 69.75	26.3 ± 57.23	−4.0 ± 56.30	21.5 ± 56.42
eGFR, ml/min/1.73 m^2^	−0.61 ± 11.74	−2.53 ± 11.70	−0.46 ± 12.56	−2.13 ± 11.84

The values are presented as the arithmetic means ± s.d.

## Discussion

This randomized, placebo-controlled phase 3 study evaluated the 24-week efficacy and the 52-week safety of dorzagliatin, a dual-acting full allosteric GKA, in combination with metformin in patients with T2D who had inadequate glycemic control with metformin alone. The DAWN study achieved its primary efficacy endpoint of reduction in HbA1c levels at 24 weeks, with a significant placebo-adjusted reduction in HbA1c of 0.66% when dorzagliatin was added to 1,500 mg of daily metformin. Dorzagliatin as an add-on therapy to metformin had a good tolerability and safety profile during the entire 52-week treatment period, with no excess occurrence of drug-related SAEs or severe hypoglycemia in the dorzagliatin and metformin group.

The participants enrolled in the DAWN trial represented patients with T2D with a mean disease duration of approximately 6 years who have a baseline HbA1c level of approximately 8.3% and a relatively high 2h-PPG level of approximately 340 mg dl^−1^ (Table 1) with a relatively low BMI of 26 kg m^−^^2^ compared to patients with diabetes from the United States^30^. The substantially high level of post-challenge glucose in these patients likely resulted from impaired GSIS and decreased glucokinase expression in both the pancreas and liver, which may have led to reduced hepatic glucose uptake and increased hepatic glucose production^12,13^. Significant reduction in 2h-PPG was observed when dorzagliatin was used as an add-on to metformin, which was in line with a significant improvement in both the β-cell function and insulin resistance, as measured by HOMA2-β and HOMA2-IR. The fast onset of HbA1c reduction was similar to that observed in the SEED study^28^, suggesting that dorzagliatin addresses the common defects in patients with diabetes at the different stages of the disease. Further studies to evaluate the mechanism of action of dorzagliatin in the modulation of gluconeogenesis, glycogenolysis and the glucokinase activity on endogenous glucose production in T2D are ongoing (NCT05098470).

Several GKAs have been tested in clinical studies in combination with metformin. PF-04937319, a dual-acting partial allosteric GKA, was studied as an add-on therapy to metformin compared to glimepiride and sitagliptin in adults with T2D^24^. PF-04937319 showed a similar placebo-adjusted reduction in HbA1c as sitagliptin (0.46% versus 0.43%) with a similar incidence of hypoglycemia (5.1% versus 1.8%) after 12-month treatment. This partial GKA has shown no effect on the reduction of FPG. Glimepiride as an add-on to metformin resulted in a higher placebo-adjusted HbA1c reduction of 0.83% but a much higher incidence of hypoglycemia of 34.4% in the same study. PF-04937319 showed good safety and tolerability profile in this 12-week study with no clinically significant changes in lipids, liver enzyme levels and blood pressure profile, which is different from what has been reported in a phase 2 study of MK-0941 as an add-on to insulin in patients with T2D. PF-04937319 produces human metabolite with N-demethylation and is currently under phase 3 evaluation in patients with T2D with an alternative dose regimen^31,32^.

Our study does have limitations, which should be considered when interpreting the results. The foremost limitation was that it was designed for registration in China and was conducted in only one country. Additionally, the DAWN study was designed to evaluate the efficacy and safety of dorzagliatin as an add-on to metformin in patients who failed to achieve optimal glycemic control with a full dose of metformin at 1,500 mg daily. Therefore, results from the population selected by the inclusion and exclusion criteria, including medication history and ranges of HbA1c levels, C-peptide levels and hepatic and renal parameters in this study, might not be generalizable to broader patient populations. A second limitation is that this study was designed as an add-on to metformin standard of care therapy and did not compare the effects of dorzagliatin with other anti-diabetic drugs. The efficacy and safety outcomes of combination treatments involving dorzagliatin with multiple anti-diabetic drugs need to be evaluated in future international multi-center clinical trials. Results from a clinical pharmacological evaluation indicated the benefits of dorzagliatin as an add-on to sitagliptin and empagliflozin in patients with T2D in the United States^11^. A significant reduction in the plasma glucose and an increase in the GSIS in the combination therapy were observed^11^. Third, because the study objective was to observe the efficacy at 24 weeks and safety over 52 weeks, no control group was incorporated into the design of the open-label treatment period from week 24 to week 52. Thus, a prolonged and placebo-controlled study will address the question of longer-term efficacy after 24 weeks of treatment. The total observation period of 1 year was also insufficient to evaluate the long-term safety profile and benefits of dorzagliatin. Finally, we note that self-monitoring of blood glucose and self-reporting of hypoglycemia might not fully capture the incidence of hypoglycemia in this study, which might have been observed with additional clinical monitoring; therefore, it is possible that asymptomatic incidents of hypoglycemia were underreported.

In summary, DAWN showed that using dorzagliatin as an add-on to metformin can provide effective glycemic control with a good tolerability and safety profile in patients with T2D who had inadequate control with metformin alone.

## Methods

### Trial design

DAWN is a phase 3 trial conducted at 73 sites in China that enrolled patients with T2D experiencing inadequate glycemic control with metformin alone. The trial included a 2-week screening period, a 4-week single-blind placebo run-in period, a 24-week double-blind placebo-controlled treatment period, an extended 28-week open-label treatment period and a 1-week safety follow-up period (Extended Data Fig. 1).

### Patients

Patients were recruited at the study site by investigators. The patients were required to sign an informed consent form before participating in any study procedures. Afterwards, the patients were screened for eligibility. Patients were not paid for taking part in the study, but they were compensated for travel to the site visit and blood draw at the site. During the study, patients were paid for completing different procedures at each visit. If the patients suffer any trial-related injury during the trial period, they can receive treatment at the study hospital, and the sponsor will cover the relevant medical costs as well as financial compensation as defined in relevant laws and regulations of China. Individuals (aged 18–75 years) were included in the trial if they were diagnosed with T2D; underwent diet and exercise interventions; were receiving stable daily doses of metformin (a minimum of 1,500 mg per day) for at least 12 weeks before screening; had an HbA1c level of 7.5–10.0% and a BMI of 18.5–35.0 kg m^−^^2^ at screening; were willing to conduct self-monitoring of blood glucose; and were willing to provide the written informed consent form and comply with the study protocol.

The exclusion criteria were as follows: any previous anti-diabetic treatment other than metformin within the previous 12 weeks before screening; previous insulin treatment for more than 30 days within 1 year before screening; severe hypoglycemia without cause within the previous 3 months and frequent hypoglycemia occurring more than three times within the previous month; a fasting C-peptide level of less than 0.81 ng ml^−1^ (normal range, 0.81–3.85 ng ml^−1^) at screening; a medical history of diabetic ketoacidosis, diabetes lactic acidosis or hyperosmotic non-ketotic diabetic coma; and clinically diagnosed with type 1 diabetes or diabetes caused by pancreatic damage or other special types of diabetes. Medical history and concomitant disease-related exclusion criteria were as follows: major cardio-cerebrovascular diseases within 6 months before screening; unstable or rapidly progressive kidney disease; active liver diseases; psychiatric diseases; hemoglobinopathies and immunocompromise; any type of malignancy; any endocrine system diseases or unstable immune diseases that need medical interventions and were determined by the investigator as not being suitable for this study; history of drug abuse; received oral or injected corticosteroids treatment within 1 year before screening; and alcohol intake of more than two units per day or more than 14 units per week. Physical examination and laboratory results-related exclusion criteria were alanine aminotransferase (ALT) or aspartate aminotransferase (AST) >2.5× the upper limit of normal (ULN); serum total bilirubin (TBil) level >1.5× the ULN; serological evidence of hepatitis virus infection at screening; estimated glomerular filtration rate (eGFR) <60 ml/min/1.73 m^−2^ at screening; TG >5.7 mmol L^−1^ at screening; anemia of any cause; abnormal laboratory or electrocardiogram results that may interfere with the safety evaluation; uncontrolled hypertension to anti-hypertensive treatment with stable doses for at least 4 weeks at screening; and any accompanied disease, treatment or participant status that may impede completion of the whole study or potentially affect the interpretation of efficacy and safety data.

### Trial procedures

During the run-in period, all patients were treated with metformin (Glucophage, immediate-release hydrochloride tablets, 1,500 mg per day, provided by the sponsor) as the baseline therapy. The patients were advised to maintain their original dosage regimen of metformin 2–3 times per day for a total of 1,500 mg. The patients who received a dose of metformin >1,500 mg per day before entering the study were required to reduce the dose to 1,500 mg per day. After the run-in period, eligible patients were randomly assigned in a 1:1 ratio to receive either 75 mg of dorzagliatin BID and metformin (1,500 mg per day) or placebo BID and metformin (1,500 mg per day) for 24 weeks on a background of a diet and exercise regimen. Then, all patients received 75 mg of dorzagliatin BID and metformin (1,500 mg per day) treatment for 28 weeks.

At week 3 of the 4-week run-in period, the patients were reevaluated before randomization to confirm eligibility. Eligibility criteria included an HbA1c level of 7.5–10.0% and an FPG level of 126.0–239.4 mg dl^−1^ at randomization. Diet and exercise counseling was provided throughout the experiment. Randomization and drug dispensing were performed with an interactive web response system (IWRS) (Medidata RAVE RTSM 2020.3.2). A stratified randomization method with the permuted block randomization algorithm was used. The blocks were dynamically allocated to each site and stratum from the randomization list. A unique ID number was provided by the vendor and marked on the drug box. Through central randomization, the randomization codes were generated by the IWRS system based on stratification factors (T2D disease duration ≤3 years or >3 years and HbA1c level ≤8.5% or >8.5% at randomization) and the block size. During the double-blind treatment, the random allocation sequences were concealed from the patients, investigators and other study members until week 24, and the blinding was maintained during the whole 52 weeks of treatment. The placebo tablets had the same size, color, smell and appearance as the active drug tablets.

### Efficacy endpoints

The primary efficacy endpoint was the change in HbA1c level from baseline to week 24. Key secondary efficacy endpoints included the changes in 2h-PPG and FPG levels from baseline to week 24, HbA1c level at each visit (except week 24) and the percentage of patients who reached an HbA1c level of <7.0% at week 24. Additional efficacy endpoints included the changes in HOMA2-β (an index of β-cell function) and HOMA2-IR (an index of insulin resistance) from baseline. A complete list of other efficacy endpoints is provided in the statistical analysis plan in the supplementary materials. During the study, HbA1c and FPG levels were measured and evaluated at each visit from visits 4–15. At visits 4, 7, 10 and 15, 2h-PPG levels were measured using a standardized mixed-meal tolerance test (MMTT).

### Safety endpoints

Safety assessments were performed at each study visit. Blood and urine samples were collected at each visit. Adverse Events (AEs) and Serious Adverse Events (SAEs) were assessed throughout the trial. Hypoglycemic episodes were classified according to American Diabetes Association definitions. Vital signs and clinical laboratory test results were assessed, and physical examinations were performed. All laboratory tests were conducted in the central laboratory. All samples were collected at the site.

### Trial oversight

The trial was conducted in accordance with the principles of the Declaration of Helsinki, Good Clinical Practice guidelines and laws and regulations in China. The study protocol was amended once during the study. The important changes to the protocol, which mainly involve exclusion criteria and randomization criteria, are listed as follows: adjusted to fasting C-peptide <0.81 ng ml^−1^ (0.27 nmol L^−1^) at screening; short-term external use of corticosteroids treatment within 1 year at screening was permissible; inhaled glucocorticoid treatment was unacceptable; stable coronary heart disease was allowed; exclusion and randomization criteria related to blood pressure and anti-hypertensive drugs were redefined as systolic blood pressure ≥160 mmHg or diastolic blood pressure ≥100 mmHg at screening or patients who added/changed anti-hypertensive drugs or adjusted dose within 4 weeks before screening; 12-lead electrocardiogram was added to visit 3 as a pre-randomization criterion to ensure smooth conduct of the study; a visit window period of ±3 days for visit 2 was added to match actual operational needs; clarified that all blood samples were collected in fasting status except for MMTT 30 minutes and 120 minutes; 30-minute (±3 minutes were allowed) testing point for the MMTT was added to optimize the study protocol; clarified that the blood sampling time (MMTT, 120 minutes) for pharmacokinetics was before taking study medications; and the rule that patients need not to be in fasting status at visit 2 was removed for compliance. Minor protocol revisions involve wording, consistency and accuracy. The trial protocol and amendments were approved by the local ethics committees of all study sites: China-Japan Friendship Hospital, Beijing, China; Affiliated Drum Tower Hospital, Medical School of Nanjing University, Nanjing, China; The First People’s Hospital of Changde City, Changde, China; Jinan Central Hospital Affiliated to Shandong First Medical University, Jinan, China; Cangzhou People’s Hospital, Cangzhou, China; Peking Union Medical College Hospital, Beijing, China; The First Affiliated Hospital and College of Clinical Medicine of Henan University of Science and Technology, Luoyang, China; The Affiliated Hospital of Qingdao University, Qingdao, China; Qingdao Central Hospital, Qingdao, China; Chenzhou First People’s Hospital, Chenzhou, China; The Second Affiliated Hospital of Nanjing Medical University, Nanjing, China; Tongji Hospital of Tongji University, Shanghai, China; The First Affiliated Hospital of Kunming Medical University, Kunming, China; The First Bethune Hospital of Jilin University, Changchun, China; Huzhou Central Hospital, Huzhou, China; The Affiliated Hospital of Jiangsu University, Zhenjiang, China; Nanjing First Hospital, Nanjing, China;The Affiliated Hospital of Xuzhou Medical University, Xuzhou, China; Inner Mongolia Baogang Hospital, Baotou, China; The First People’s Hospital of Yue Yang, Yueyang, China; Jingzhou Hospital Affiliated to Yangtze University, Jingzhou, China; Jiangxi Pingxiang People’s Hospital, Pingxiang, China; Jiangxi Provincial People’s Hospital, Nanchang, China; The First People’s Hospital of Shunde, Foshan, China;Chongqing General Hospital, Chongqing, China; Qinghai University Affiliated Hospital, Xining, China; Luoyang Central Hospital, Luoyang, China; General Hospital of Ningxia Medical University, Yinchuan, China; The General Hospital of Xuzhou City Mining Group, Xuzhou, China; Shanghai Pudong New Area People’s Hospital, Shanghai, China; Beijing Friendship Hospital Pinggu Campus, Capital Medical University, Beijing, China; Xinhua Hospital Affiliated to Shanghai Jiao Tong University School of Medicine, Shanghai, China; Baotou Central Hospital, Baotou, China; West China Hospital of Sichuan University, Chengdu, China; The 960th Hospital of the PLA Joint Logistics Support Force, Jinan, China; Yiyang Central Hospital, Yiyang, China; Yangpu Hospital, Tongji University, Shanghai, China; The First Affiliated Hospital of Anhui Medical University, Hefei, China; Taihe Hospital, Hubei University of Medicine, Shiyan, China; The First Hospital of China Medical University, Shenyang, China; Southern Medical University Nanfang Hospital, Guangzhou, China; The Central Hospital of Wuhan, Wuhan, China; The Affiliated Hospital of Guizhou Medical University, Guiyang, China; Anhui Provincial Hospital, Hefei, China; The First Affiliated Hospital of Bengbu Medical College, Bengbu, China; Huadong Hospital Affiliated to Fudan University, Shanghai, China; Jiangsu Province Hospital, Nanjing, China; Jining No. 1 People’s Hospital, Jining, China; Tianjin People’s Hospital, Tianjin, China; The First Hospital of Qiqihar, Qiqihar, China; Jilin Central General Hospital, Jilin, China; Third People’s Hospital of Yunnan Province, Kunming, China; Peking University First Hospital, Beijing, China; Ningbo First Hospital, Ningbo, China; The Affiliated Huai’an No. 1 People’s Hospital of Nanjing Medical University, Huai’an, China; Shanghai Tenth People’s Hospital, Shanghai, China; Emergency General Hospital, Beijing, China; The First Affiliated Hospital of Hainan Medical University, Haikou, China; Peking University Shougang Hospital, Beijing, China; Qinghai Provincial People’s Hospital, Xining, China; Hainan General Hospital, Haikou, China; Liuzhou People’s Hospital, Liuzhou, China; Yijishan Hospital, The First Affiliated Hospital of Wannan Medical University, Wuhu, China; The Second People’s Hospital of Huai’an, Huai’an, China; Jiangsu Taizhou People’s Hospital, Taizhou, China; Zhejiang Hospital, Hangzhou, China; Aerospace Center Hospital, Beijing, China; Yanbian University Hospital, Yanji, China; The Third Affiliated Hospital of Southern Medical University, Guangzhou, China; Beijing Luhe Hospital Affiliated to Capital Medical University, Beijing, China; Shanghai East Hospital, Tongji University, Shanghai, China; and Zhongshan Hospital, Fudan University, Shanghai, China. All patients provided written informed consent before trial entry. The trial was conducted in accordance with Chinese Diabetes Society guidelines, which require physicians to educate and strictly enforce improved exercise and dietary control and self-monitoring of blood glucose levels (at least two times per week) while treating patients with T2D.

### Statistical analyses

We hypothesized that dorzagliatin would show superiority to placebo in decreasing HbA1c levels in patients after 24 weeks of treatment. For the primary endpoint HbA1c levels, we calculated that a total sample size of 750 patients would provide the trial with 95.4% power to detect a difference of 0.4% between the dorzagliatin and metformin group and the placebo and metformin group in a 1:1 ratio of allocation at a two-sided significance level of 0.05, assuming a standard deviation (s.d.) of 1.5%. The FAS included all randomized patients who took at least one dose of the study drug and completed at least one post-treatment measurement of the primary endpoint during the double-blind treatment period. All major protocol deviations were reviewed in the blind data review meeting to determine whether a participant with any major protocol deviations should be excluded from the per-protocol set (PPS). The safety set (SS) included all randomized patients who took at least one dose of the study drug. Because this trial included only one confirmatory hypothesis test—that is, to test the null hypothesis of no difference in the primary endpoint between the dorzagliatin and metformin and placebo and metformin groups—no adjustment for multiple comparisons was needed.

The primary analysis method for the primary endpoint in the FAS was a mixed model for repeated measures (MMRM) without missing value imputation, which included treatment group, scheduled visit, the interaction of treatment group with the scheduled visit, pooled site, duration of diabetes (≤3 years or >3 years) and baseline HbA1c levels as fixed effects. In the MMRM, data collected after the initiation of prohibited anti-diabetic medications were handled as missing data. The least-squares (LS) means of each treatment group, the ETD between the dorzagliatin and metformin and placebo and metformin groups and its 95% CI were calculated. A sensitivity analysis of the primary endpoint was performed in the FAS and an analysis of covariance (ANCOVA) model with factors of treatment group, pooled site, duration of diabetes (≤3 years or >3 years) and baseline HbA1c levels. In the ANCOVA model, missing values were imputed using the method of last observation carried forward (LOCF), which was applied after excluding data collected after the initiation of administering prohibited anti-diabetic medications. Another sensitivity analysis of the primary endpoint was performed using the same MMRM in the PPS. Primary endpoint data that were missing due to the inconvenience of on-site visits during the Coronavirus Disease 2019 epidemic (21 patients, including 13 patients in the dorzagliatin and metformin group and eight patients in the placebo and metformin group) were processed using the methods described in the statistical analysis plan. Then, a corresponding sensitivity analysis was performed using the same method as described in the primary analysis.

The secondary efficacy endpoints, including 2h-PPG, FPG and HbA1c levels at each visit (except week 24), were assessed using the same MMRM for the primary endpoint. Another secondary efficacy endpoint—that is, the percentage of patients who reached an HbA1c level of <7.0%—was estimated in the FAS based on the data imputed with the LOCF approach. The ORs and 95% CIs between the two treatment groups were estimated using the logistic regression model with the factors of the treatment group, pooled site, interaction of the treatment group with the pooled site (interaction effect removed from the model if not significant at a two-sided α level of 0.1), duration of diabetes (≤3 years or >3 years) and baseline HbA1c level. Exploratory efficacy endpoints of the change from baseline in HOMA2-β and HOMA2-IR were analyzed in the FAS using an ANCOVA model with the factors treatment group, pooled site, duration of diabetes (≤3 years or >3 years) and baseline level of the analyzed variable. A post hoc analysis was conducted to analyze an exploratory efficacy endpoint of the composite endpoint (HbA1c <7.0% without hypoglycemia and no weight gain), which was estimated based on the data imputed by the LOCF approach in the FAS. The OR and 95% CI between two treatment groups were estimated using the logistic regression model with categorical independent variables of the treatment group, pooled site, duration of diabetes (≤3 years or >3 years), interaction of the treatment group with the pooled site (interaction effect removed from the model if not significant at a two-sided α level of 0.1) and a continuous independent variable of baseline HbA1c level. Furthermore, as another post hoc analysis to explore the in-group reduction in HbA1c from baseline at week 24 and week 52, paired *t*-tests based on observed measurements were carried out to compare HbA1c between at baseline and at week 24 or week 52 in each of dorzagliatin and placebo groups.

The incidence of treatment-emergent AEs between the first intake of the double-blind study drug and the 7th day after the last dose was summarized by the treatment group and compared between the two groups. The incidence of hypoglycemic events was also summarized by the treatment group and compared between treatment groups. The commercially available software Electronic Data Capture (Medidata RAVE, Classic Rave version 2018.2.4) was used for clinical trial patient data collection. Additional details regarding the statistical analysis are provided in the statistical analysis plan in the supplementary materials, and SAS software (SAS Institute, version 9.4) was used for analyses.

### Reporting Summary

Further information on research design is available in the Nature Research Reporting Summary linked to this article.

## Online content

Any methods, additional references, Nature Research reporting summaries, source data, extended data, supplementary information, acknowledgements, peer review information; details of author contributions and competing interests; and statements of data and code availability are available at 10.1038/s41591-022-01803-5.

## Supplementary information

Supplementary Information Supplementary Tables 1–9, Clinical Study Protocol (original protocol (version 1.0), final protocol (version 1.1) and summary of changes) and statistical analysis plan

Reporting Summary

## Data Availability

Data from these analyses in the DAWN study cannot be made publicly available due to the sponsor’s contractual obligations. We encourage researchers or parties interested in collaboration for non-commercial use to apply to the corresponding author (lichen@huamedicine.com). Applications should outline specifically what data they are interested in receiving and how the data will be used. The use of data must also comply with the requirements of the Human Genetics Resources Administration of China and other country- or region-specific regulations. All data shared will be de-identified and will be made available 2 years after the date of publication. A signed data access agreement with the sponsor is required before accessing the shared data. The study protocol and statistical analysis plan are provided with the paper.
