# Supplementary material for: Dorzagliatin add-on therapy to metformin in patients with type 2 diabetes: a randomized, double-blind, placebo-controlled phase 3 trial
Source: Nat Med. 2022 May 12;28(5):974–81. doi: 10.1038/s41591-022-01803-5 (PMC9117147; doi:10.1038/s41591-022-01803-5)
Supplement: Supplementary file 1 — Supplementary Tables 1–9, Clinical Study Protocol (original protocol (version 1.0), final protocol (version 1.1) and summary of changes) and statistical analysis plan [file 41591_2022_1803_MOESM1_ESM.pdf]

nature portfolio

https://doi.org/10.1038/s41591-022-01803-5

Supplementary information

Dorzagliatin add-on therapy to metformin in patients with type 2 diabetes: a randomized, double-blind, placebo-controlled phase 3 trial

In the format provided by the authors and unedited

Supplementary Table 1. Changes in Glycated Hemoglobin (%) by Scheduled Visits in 24-Week Double-Blind Treatment Period.

	Dorzagliatin and Metformin				Placebo and Metformin				Dorzagliatin vs. Placebo	
	N	Mean (SD)	vs. Baseline		N	Mean (SD)	vs. Baseline		LS Mean Difference (95% CI)	P-value
			LS Mean (SE)	95% CI			LS Mean (SE)	95% CI		
Baseline	376	8.24(0.595)	-	-	375	8.28(0.590)	-	-	-	-
Week 4	371	7.55(0.714)	-0.71(0.024)	(-0.76, -0.66)	374	8.14(0.674)	-0.17(0.024)	(-0.21, -0.12)	-0.54(-0.61, -0.48)	<0.0001
Week 8	365	7.24(0.822)	-1.03(0.034)	(-1.10, -0.97)	366	8.06(0.790)	-0.24(0.034)	(-0.31, -0.18)	-0.79(-0.88, -0.70)	<0.0001
Week 12	361	7.13(0.928)	-1.13(0.040)	(-1.21, -1.05)	354	8.00(0.826)	-0.28(0.041)	(-0.36, -0.20)	-0.85(-0.96, -0.74)	<0.0001
Week 16	354	7.11(0.958)	-1.14(0.044)	(-1.22, -1.05)	344	7.95(0.842)	-0.32(0.044)	(-0.41, -0.24)	-0.82(-0.94, -0.69)	<0.0001
Week 20	351	7.18(0.984)	-1.05(0.046)	(-1.14, -0.96)	345	7.94(0.864)	-0.33(0.046)	(-0.42, -0.24)	-0.72(-0.85, -0.59)	<0.0001
Week 24	335	7.19(1.005)	-1.02(0.047)	(-1.11, -0.93)	336	7.90(0.851)	-0.36(0.048)	(-0.45, -0.26)	-0.66(-0.79, -0.53)	<0.0001

The least-squares (LS) mean, standard error (SE), LS mean differences, corresponding 95% confidence interval (CI) and P-value were estimated using a mixed model for repeated measures in the full-analysis set. All statistical tests were 2-sided at a significance level of 0.05, and no adjustments were made for multiplicity. SD: standard deviation.

Supplementary Table 2. Changes in Fasting Plasma Glucose (mg/dl) by Scheduled Visits in 24-Week Double-Blind Treatment Period.

	Dorzagliatin and Metformin				Placebo and Metformin				Dorzagliatin vs. Placebo	
	N	Mean (SD)	vs. Baseline		N	Mean (SD)	vs. Baseline		LS Mean Difference (95% CI)	P-value
			LS Mean (SE)	95% CI			LS Mean (SE)	95% CI		
Baseline	376	176.04(28.818)	-	-	375	178.92 (30.114)	-	-	-	-
Week 4	374	160.38(34.956)	-16.92(1.512)	(-19.80, -13.86)	374	178.38(34.902)	-0.90(1.512)	(-3.78, 2.16)	-16.02(-19.98, -11.88)	<0.0001
Week 8	368	158.76(34.236)	-18.54(1.548)	(-21.60, -15.48)	366	178.02(35.136)	-1.26(1.548)	(-4.32, 1.80)	-17.28(-21.42, -13.14)	<0.0001
Week 12	360	161.46(34.974)	-15.66(1.602)	(-18.72, -12.42)	354	173.52(34.326)	-4.50(1.602)	(-7.74, -1.44)	-10.98(-15.30, -6.84)	<0.0001
Week 16	354	160.92(35.424)	-16.02(1.674)	(-19.26, -12.78)	346	172.26(33.138)	-5.40(1.674)	(-8.64, -1.98)	-10.62(-15.12, -6.12)	<0.0001
Week 20	350	162.72(35.136)	-13.50(1.710)	(-16.92, -10.08)	345	170.10(32.058)	-7.20(1.728)	(-10.62, -3.78)	-6.48(-10.98, -1.80)	0.0068
Week 24	335	163.98(37.566)	-12.06(1.908)	(-15.84, -8.46)	335	171.72(34.956)	-5.22(1.908)	(-9.00, -1.62)	-6.84(-12.06, -1.62)	0.0091

The least-squares (LS) mean, standard error (SE), LS mean difference and corresponding 95% confidence interval (CI) and P-value were estimated using a mixed model for repeated measures in the full-analysis set. All statistical tests were 2-sided at a significance level of 0.05, and no adjustments were made for multiplicity. SD: standard deviation.

Supplementary Table 3. Changes in 2-Hour Postchallenge Glucose (mg/dl) in 24-Week Double-Blind Treatment Period.

	Dorzagliatin and Metformin				Placebo and Metformin				Dorzagliatin vs. Placebo	
	N	Mean (SD)	vs. Baseline		N	Mean (SD)	vs. Baseline		LS Mean Difference (95% CI)	P-value
			LS Mean (SE)	95% CI			LS Mean (SE)	95% CI		
Baseline	376	338.94(55.386)	-	-	374	343.08(54.180)	-	-	-	-
Week 12	359	243.36(71.658)	-99.54(3.114)	(-105.66, -93.42)	353	300.06(57.438)	-45.00(3.132)	(-51.12, -38.88)	-54.54(-62.82, -46.44)	<0.0001
Week 24	335	243.90(71.766)	-98.10(3.312)	(-104.58, -91.44)	335	291.42(56.772)	-53.46(3.312)	(-59.94, -46.98)	-44.64(-53.28, -35.82)	<0.0001

The least-squares (LS) mean, standard error (SE), LS mean differences and corresponding 95% confidence interval (CI) and P-value were estimated using a mixed model for repeated measures in the full-analysis set. All statistical tests were 2-sided at a significance level of 0.05, and no adjustments were made for multiplicity. At visits 4, 7, 10 and 15, the 2-hour postchallenge glucose level was collected by a standardized mixed-meal tolerance test. SD: standard deviation.

Supplementary Table 4. Changes in Glycated Hemoglobin (%) by Scheduled Visits in 52-Week Treatment Period.								
	Dorzagliatin and Metformin				Placebo and Metformin			
	N	Mean (SD)	Change from Baseline	P value	N	Mean (SD)	Change from Baseline	P value
Baseline	376	8.24(0.595)	-		375	8.28(0.590)	-	
Week 4	371	7.55(0.714)	-0.69(0.452)	<0.0001	374	8.14(0.674)	-0.15(0.397)	<0.0001
Week 8	365	7.24(0.822)	-1.01(0.663)	<0.0001	367	8.06(0.789)	-0.22(0.579)	<0.0001
Week 12	361	7.13(0.928)	-1.12(0.839)	<0.0001	355	8.00(0.825)	-0.27(0.663)	<0.0001
Week 16	354	7.11(0.958)	-1.14(0.911)	<0.0001	345	7.95(0.842)	-0.32(0.724)	<0.0001
Week 20	351	7.18(0.984)	-1.06(0.955)	<0.0001	346	7.94(0.863)	-0.33(0.737)	<0.0001
Week 24	335	7.19(1.005)	-1.05(0.978)	<0.0001	336	7.90(0.851)	-0.36(0.729)	<0.0001
Week 28	326	7.21(1.038)	-1.03(1.005)	<0.0001	325	7.42(0.911)	-0.85(0.809)	<0.0001
Week 34	320	7.27(1.097)	-0.96(1.056)	<0.0001	312	7.13(0.966)	-1.15(0.886)	<0.0001
Week 40	321	7.29(1.023)	-0.94(0.976)	<0.0001	296	7.16(1.053)	-1.13(0.962)	<0.0001
Week 46	305	7.38(1.031)	-0.85(0.977)	<0.0001	301	7.25(1.097)	-1.04(1.014)	<0.0001
Week 52	312	7.41(1.136)	-0.81(1.059)	<0.0001	301	7.31(1.082)	-0.96(0.971)	<0.0001

Changes from baseline values are the arithmetic means (SD). SD: standard deviation. P-values are calculated from paired-t tests comparing HbA1c between baseline and each post-baseline visit.

Supplementary Table 5. Changes in Fasting Plasma Glucose (mg/dl) by Scheduled Visits in 52-Week Treatment Period.						
	Dorzagliatin and Metformin			Placebo and Metformin		
	N	Mean (SD)	Change from Baseline	N	Mean (SD)	Change from Baseline
Baseline	376	176.04(28.818)	-	375	178.92(30.114)	-
Week 4	374	160.38(34.956)	-15.66(28.224)	374	178.38(34.902)	-0.54(31.068)
Week 8	368	158.76(34.236)	-17.46(30.744)	367	177.84(35.100)	-0.72(30.312)
Week 12	360	161.46(34.974)	-14.04(31.914)	355	173.34(34.290)	-4.14(30.312)
Week 16	354	160.92(35.424)	-14.58(34.128)	347	172.26(33.138)	-5.04(30.690)
Week 20	350	162.72(35.136)	-12.60(35.928)	346	170.10(32.022)	-7.02(31.950)
Week 24	335	163.98(37.566)	-11.52(38.556)	335	171.72(34.956)	-5.76(33.804)
Week 28	326	164.34(37.368)	-11.52(37.242)	325	154.08(35.712)	-23.40(36.072)
Week 34	321	163.80(37.422)	-12.06(38.574)	312	156.78(35.964)	-20.52(36.972)
Week 40	321	164.52(38.322)	-10.80(37.368)	296	159.12(36.900)	-18.36(38.502)
Week 46	305	163.98(36.486)	-11.16(35.784)	301	163.80(43.092)	-13.50(41.094)
Week 52	312	166.50(38.214)	-8.64(37.422)	301	162.00(39.186)	-15.30(35.784)

Changes from baseline values are the arithmetic means (SD). SD: standard deviation.

Supplementary Table 6. Changes in 2-Hour Postchallenge Glucose (mg/dl) by Scheduled Visits in 52-Week Treatment Period.						
	**Dorzagliatin and Metformin**			**Placebo and Metformin**		
	N	Mean (SD)	Change from Baseline	N	Mean (SD)	Change from Baseline
Baseline	376	338.94(55.386)	-	374	343.08(54.180)	-
Week 12	359	243.36(71.658)	-95.58(65.214)	354	299.88(57.528)	-42.30(55.548)
Week 24	335	243.90(71.766)	-95.04(72.090)	335	291.42(56.772)	-51.66(56.574)
Week 52	311	244.44(69.084)	-92.16(69.840)	300	236.34(72.396)	-104.94(72.450)

Changes from baseline values are the arithmetic means (SD). At visits 4, 7, 10 and 15, the 2-hour postchallenge glucose level was measured by a standardized mixed-meal tolerance test. SD: standard deviation

Supplementary Table 7. Adverse Events in 24-Week Double-Blind Treatment Period and Additional 28-Week Open-Label Treatment Period.

Event	Double-blind treatment period (0-24 week)				Open-label treatment period (24-52 week)			
	Dorzagliatin and Metformin (N=382)		Placebo and Metformin (N=384)		Dorzagliatin and Metformin – Dorzagliatin and Metformin (N=349)		Placebo and Metformin – Dorzagliatin and Metformin (N=343)	
	No. of Patients (%)	*No. of Events*	*No. of Patients (%)*	*No. of Events*	*No. of Patients (%)*	*No. of Events*	*No. of Patients (%)*	*No. of Events*
Any adverse event	299 (78)	926	278 (72)	826	251 (72)	643	252 (74)	790
Mild	274 (72)	895	253 (66)	799	226 (65)	607	226 (66)	754
Moderate	23 (6)	27	25 (7)	27	23 (7)	34	24 (7)	33
Severe	2 (1)	4	0	0	2 (1)	2	2 (1)	3
Drug-related adverse event #	53 (14)	82	36 (9)	52	29 (8)	43	43 (13)	65
Mild	50 (13)	77	35 (9)	51	29 (8)	43	41 (12)	61
Moderate	3 (1)	5	1 (0.3)	1	0	0	2 (1)	4
Severe	0	0	0	0	0	0	0	0
Adverse event leading to drug discontinuation¶	1 (0.3)	1	3 (1)	4	0	0	2 (1)	3
Adverse event leading to withdrawal from the study¶¶	1 (0.3)	1	2 (1)	2	0	0	2 (1)	3
Any serious adverse event	19 (5)	24	17 (4)	19	11 (3)	12	15 (4)	19
Resulting in death	1 (0.3) †	1	0	0	0	0	0	0
Other serious adverse event	18 (5)	23	17 (4)	19	11 (3)	12	15 (4	19
Serious adverse event leading to drug discontinuation	3 (1)	4	4 (1)	4	2 (1)	2	4 (1)	5
Serious adverse event leading to withdrawal from the study	3 (1)	4	3 (1)	3	2 (1)	2	1 (0.3)	1
Adverse events in ≥5% of the patients‖								
Upper respiratory tract infection	54 (14)	59	52 (14)	60	38 (11)	46	43 (13)	45
Related to the study drug§	0	0	0	0	0	0	0	0
Hyperlipidemia	52 (14)	61	33 (9)	41	39 (11)	42	51 (15)	62
Related to the study drug§	2 (1)	2	1 (0.3)	1	1 (0.3)	1	4 (1)	4
Protein urine present	30 (8)	38	30 (8)	33	29 (8)	31	26 (8)	30
Related to the study drug§	0	0	3 (1)	3	2 (1)	2	1 (0.3)	1
Hyperuricemia	37 (10)	45	14 (4)	14	19 (5)	22	24 (7)	27
Related to the study drug§	3 (1)	3	0	0	1 (0.3)	1	3 (1)	3
Hypertriglyceridemia	21 (6)	27	11 (3)	15	14 (4)	16	13 (4)	14
Related to the study drug§	7 (2)	7	2 (1)	2	3 (1)	3	5 (2)	5
Hypoglycemia‡								
Severe hypoglycemia††	0	0	0	0	0	0	0	0

Clinically significant hypoglycemia * (Blood glucose level <54 mg/dl)	3 (1)	3	0	0	1 (0.3)	2	0	0
Mild hypoglycemia (Blood glucose level ≤70.2 mg/dl)	15 (4)	29	3 (1)	4	8 (2)	11	15 (4)	27

Adverse events and serious adverse events that occurred during the 24-week double-blind treatment period and 28-week open-label treatment period among the patients in the safety population are included in the table and presented with their preferred terms in the *Medical Dictionary for Regulatory Activities* version 23.0[30]. The safety population included all randomized patients who took at least one dose of the study drugs. Events were included if the date of onset was between the first intake of the double-blind study medications and the 7th day after the last dose of the study medications.

#Possibly or very likely related to dorzagliatin as assessed by the investigators.

¶Serious adverse events that led to drug discontinuation are not included.

¶¶ Serious adverse events that led to withdrawal from the study are not included.

†During the 24-week double-blind treatment period, one patient in the dorzagliatin+metformin group died (confirmed by the external drug safety committee as sudden death). The patient had type 2 diabetes and established cardiovascular disease, and the event was considered unlikely related to dorzagliatin by both the investigator and the sponsor.

‖Adverse events in at least 5% of the patients in the dorzagliatin+metformin group or the placebo+metformin group are listed in the table.

§Adverse events in at least 5% of the patients related to dorzagliatin were defined as adverse events deemed by the investigators very

likely or probably related to dorzagliatin or placebo.

‡ Any hypoglycemia was defined as severe hypoglycemia or a blood glucose level≤70.2 mg/dl.

* Clinically significant hypoglycemia is defined as a blood glucose level <54 mg/dl as confirmed with a blood glucose meter.

†† Severe hypoglycemia is defined as hypoglycemia with severe cognitive impairment requiring external assistance for recovery.

Supplementary Table 8. Serious Adverse Events in 24-Week Double-Blind Treatment Period and Additional 28-Week Open-Label Treatment Period by the System Organ Class (SOC).

System Organ Class（SOC）	Double-blind treatment period (0-24 week)		Open-label treatment period (24-52 week)	
	**Dorzagliatin and Metformin (N=382)**	**Placebo and Metformin (N=384)**	**Dorzagliatin and Metformin – Dorzagliatin and Metformin (N=349)**	**Placebo and Metformin – Dorzagliatin and Metformin (N=343)**
	No. of Patients (%)	*No. of Patients (%)*	*No. of Patients (%)*	*No. of Patients (%)*
General disorders and administration site conditions	1 (0.3)	1 (0.3)	0	0
Musculoskeletal and connective tissue	1 (0.3)	3 (1)	1 (0.3)	0
Injury, poisoning and procedural	1 (0.3)	1 (0.3)	3 (1)	3 (1)
Nervous system disorders	3 (1)	1 (0.3)	0	4 (1)
Respiratory, thoracic and mediastinal	0	1 (0.3)	1 (0.3)	0
Cardiac disorders	2 (1)	0	1 (0.3)	1 (0.3)
Infections and infestations	5 (1)	2 (1)	1 (0.3)	1 (0.3)
Reproductive system and breast disorders	0	1 (0.3)		
Eye disorders	2 (1)	1 (0.3)	2 (1)	1 (0.3)
Ear and labyrinth disorders	0	1 (0.3)	0	0
Hepatobiliary disorders	0	1 (0.3)	0	1 (0.3)
Renal and urinary disorders	0	2 (1)	0	0
Gastrointestinal disorders	3 (1)	2 (1)	0	1 (0.3)
Neoplasms benign, malignant and unspecified (incl cysts and polyps)	2 (1)	0	0	3 (1)
Metabolism and nutrition disorders	0	0	0	1 (0.3)
Endocrine disorders	0	0	2 (1)	0
Blood and lymphatic system disorders	0	0	1 (0.3)	0

Serious adverse events that occurred during the 24-week double-blind treatment period and 28-week open-label treatment period among the patients in the safety population are included in the table and classified by the System Organ Class (SOC) in the *Medical Dictionary for Regulatory Activities* version 23.0[30].

**Supplementary Table 9. Benign, Malignant and Unspecified (Incl Cysts and Polyps) Neoplasms. Serious Adverse Events in 24-Week Double-Blind Treatment Period and Additional 28-Week Open-Label Treatment Period by the Preferred Term (PT).**				
**System Organ Class（SOC）<br>Preferred term (PT)**	**Double-blind treatment period (0-24 week)**		**Open-label treatment period (24-52 week)**	
	**Dorzagliatin and Metformin (N=382)**	**Placebo and Metformin (N=384)**	**Dorzagliatin and Metformin – Dorzagliatin and Metformin (N=349)**	**Placebo and Metformin – Dorzagliatin and Metformin (N=343)**
	No. of Patients (%)	*No. of Patients (%)*	*No. of Patients (%)*	*No. of Patients (%)*
Benign, malignant and unspecified (incl cysts and polyps) neoplasms	2 (1)	0	0	3 (1)
Rectal cancer [A]	0	0	0	1 (0.3)
Adrenal adenoma [B]	0	0	0	1 (0.3)
Clear cell renal cell carcinoma [C]	1 (0.3)	0	0	0
Benign neoplasm of the bladder [D]	1 (0.3)	0	0	0
Breast cancer metastatic [E]	0	0	0	1 (0.3)

[A]: A 58-year-old male patient experienced changes in bowel habits, strenuous defecation without obvious inducement, and tenesmus during the open-label treatment period (approximately 160 days after taking the open-label investigational drug). The patient was hospitalized and diagnosed with rectal cancer. The patient received surgical treatment during hospitalization. The SAE was considered unlikely related to the study drug. The investigator considered the SAE possibly related to diet and lifestyle.

[B]: A 61-year-old male patient underwent physical examination with enhanced computed tomography (CT) of the adrenal gland during the open-label treatment period (50 days after taking the open label investigational drug). The CT imaging results showed that the right adrenal area was occupied. The patient underwent right adrenal mass excision via laparoscopy. The patient was diagnosed with (right adrenal gland) adrenal cortical adenoma. The outcome of the SAE was recovered. The SAE was considered unlikely related to

the study drug by both the investigator and sponsor.

[C]: A 67-year-old female patient was found to have a right kidney mass 7 months before participating in the study. No treatment was given. The patient was reexamined, and an enlargement of the right kidney mass was revealed during the double-blind treatment period of the study (91 days after taking dorzagliatin plus metformin). The patient underwent radical nephrectomy of the right kidney via laparoscopy. The pathological diagnosis showed clear cell renal cell carcinoma. The outcome of the SAE was recovered. The SAE was considered certainly not related to the study drug by either the investigator or sponsor. The investigator considered the SAE related to the medical history.

[D]: A 64-year-old male patient developed reddish blood in urine without other symptoms during the double-blind treatment period (28 days after taking dorzagliatin plus metformin). The patient was hospitalized and underwent transurethral resection of the bladder lesion. The pathological result showed an inverted urothelial tumor. The patient's hematuria disappeared. The outcome of the SAE was recovered. The SAE was considered certainly not related to the study drug.

[E]: A 67-year-old female patient developed a right breast mass approximately 1.5 years before participating in the study. No treatment was given. The right breast mass was progressively enlarged. The surface skin was ulcerated, and a small amount of secretion was observed during the double-blind treatment period of the study. The patient was hospitalized during the open-label treatment period (26 days after taking the open-label investigational drug). The patient was diagnosed with right breast cancer (upper lateral region)

cT4aN1M1 lung, mediastinal lymph nodes and bone multiple metastases. The patient received chemotherapy. The outcome of the SAE recovered, and the condition was stable at discharge. The SAE was considered unlikely related to the study drug by the investigator.

This supplement contains the following items:

1. Original protocol (Version 1.0), final protocol (Version 1.1), summary of changes.
2. Statistical analysis plan (only one Version 1.0)

Clinical Study Protocol

A 24-week multi-center, randomized, double-blind, placebo-controlled, phase III study to evaluate the efficacy and safety of HMS5552 add-on to metformin with additional 28-week open-label treatment to evaluate the safety of HMS5552 add-on to metformin in type 2 diabetes mellitus subjects

Protocol Number: **HMM0302**

Version: **Version 1.0**

Date: **February 28th, 2017**

Sponsor： **Hua Medicine (Shanghai) Ltd.**

Address： **No. 275 Aidisheng Rd, Pudong New District, Shanghai**

Confidentiality Statement

The following is for reference only. The confidential information in this protocol belongs to Hua Medicine (Shanghai) Ltd. and is only provided to you as a principal investigator, coordinate investigator, and applicable Institutional Review Board/ regulatory authorities for review. It is strictly prohibited to disclose any information herein to any irrelevant third party without prior written authorization from the Sponsor, except that explanation for potential subjects to sign informed consent form is necessary.

Medical Experts from the Sponsor

Name	Title	Institution	Telephone
Yu Zhao	Sr. Medical Manager	Hua Medicine (Shanghai) Ltd	021-58869997-3274
Xinghua Hou	Medical Director	Hua Medicine (Shanghai) Ltd	021-58869997-3302
Yi Zhang	VP, Clinical Development	Hua Medicine (Shanghai) Ltd	021-58869997-3269

Principal Investigator

Name	Title	Institution	Address
Wenying Yang	Chief Physician/Director of Endocrinology Department	China-Japan Friendship Hospital	East Yinghuayuan St, Chaoyang District, Beijing

Sponsor

Company	Hua Medicine (Shanghai) Ltd.		
Address	No. 275 Aidisheng Rd, Zhangjiang High Technology Park, Shanghai	Post Code	201203
Telephone	021-58869997	Fax	021-58866110

Contract Research Organization (1)

Company	Covance Pharmaceutical Research and Development (Beijing) Co., Ltd., Shanghai Branch		
Address	Rm 602-606, Bldg A Pinzun International, 555 Langao Rd., Putuo District, Shanghai	Post Code	200333
Telephone	021-22265200	Fax	021-63403027

Data Management

Company	dMed Biopharmaceutical Company Ltd.		
Address	780 Cailun Rd, 6/F, Q, Zhangjiang Hi-Tech Park, Pudong, Shanghai	Post Code	201203
Telephone	021-50900085	Fax	021-68755155

Safety and Pharmacodynamic Analysis

Company	dMed Biopharmaceutical Company Ltd.		
Address	780 Cailun Rd, 6/F, Q, Zhangjiang Hi-Tech Park, Pudong, Shanghai	Post Code	201203
Telephone	021-50900085	Fax	021-68755155

Pharmacokinetic Analysis

Company	Clinical pharmacological Research Center, Chinese Academy of Medical Science, Peking Union Medical College Hospital		
Address	41, Damucang Lane, West District., Beijing	Post Code	100032
Telephone	010-69158364	Fax	010-69158364

Central Laboratory for Safety and Pharmacodynamic Analysis

Company	Covance Pharmaceutical Research and Development (Shanghai) Co., Ltd.		
Address	1/F, 6 Building, 151, Libing Rd, Shanghai	Post Code	201203
Telephone	021-51371111	Fax	021-51371301

Central Laboratory for Analysis of Pharmacokinetic Samples

Company	Wuxi AppTec		
Address	288 Fute Zhong Road, Pudong New District, Shanghai	Post Code	200131
Telephone	021-50464102	Fax	021-50461000

Electronic System Vendor

Company	Medidata Information Technology (Shanghai) Co., Ltd.			
Address	9/F 1788 West Nanjing Rd, Jing'an District., Shanghai		Post Code	200040
Telephone	021-22310417	Fax	N/A	

Protocol Signature Page

I agree:

- To conduct this study strictly following the protocol, Good Clinical Practice (GCP) and applicable regulatory requirements.
- To preserve all information provided by Hua Medicine (Shanghai) Ltd. in accordance with confidentiality statement. All information should also be marked as confidential when submitted to ethics committee (EC).

I have read the entire protocol and agreed with all content.

____________________________ _____________________ _______________

Sponsor Representative Name Signature Date (Year-Month-Day)

Protocol Signature Page

I have read the protocol (version 1.0) entitled as "A 24-week multi-center, randomized, double-blind, placebo-controlled, phase III study to evaluate the efficacy and safety of HMS5552 add-on to metformin with additional 28-week open-label treatment to evaluate the safety of HMS5552 add-on to metformin in type 2 adult diabetes mellitus subjects". I agree to conduct this study in accordance with the protocol.

I agree to follow the guidance of GCP and other regulations/guidelines applicable to China Food and Drug Administration (CFDA). I declare that I will be responsible for overall conduct of this study. I also agree to ensure that all assistants and colleagues involved in this study are aware of their obligations.

I agree and confirm that without prior written approval from Hua Medicine (Shanghai) Ltd, I will not use the confidential information in this protocol for purposes other than study evaluation or implementation.

I agree that the electronic signature which is in accordance with national safety standards and regulations is legally equivalent to wet signature.

Principal Investigator Name:___________________

Signature:___________________________ Date (Year-Month-Day):_________________

Study Protocol Synopsis

**Protocol Number**	HMM0302
**Study Title**	A 24-week multi-center, randomized, double-blind, placebo-controlled, phase III study to evaluate the efficacy and safety of HMS5552 add-on to metformin with additional 28-week open-label treatment to evaluate the safety of HMS5552 add-on to metformin in type 2 adult diabetes mellitus subjects
**Study Objective**	To evaluate the efficacy and safety of HMS5552 add-on to metformin after 24-week treatment, and the safety after 52-week treatment in subjects with type 2 diabetes mellitus (T2DM) whose blood glucose is still insufficiently controlled under the stable metformin treatment
**Study Design**	This study is a phase III study in subjects with T2DM whose blood glucose is still insufficiently controlled under the stable metformin treatment.<br>The overall study design is as follows. After screening, eligible subjects enter a 4-week, single-blind, placebo run-in period on the basis of diet and exercise interventions. Meanwhile, subjects are treated with metformin (Glucophage) at 1500mg/day as basic therapy during the run-in period. At the 3rd week of run-in period, the randomization eligibility is confirmed by laboratory tests at Day -5(±2 days) before randomization. Eligible subjects are randomized to HMS5552 75mg BID group or placebo group at a ratio of 1:1 at randomization visit after the eligibility is confirmed. And then they enter a 24-week double-blind treatment period during which metformin (1500 mg/day) should be maintained in both groups as basic therapy, followed by a 28-week open-label period with HMS5552 75mg BID add-on to metformin (1500 mg/day) treatment. After the 52-week treatment, all study medications should be stopped and subjects are observed for 1 week for safety evaluation. The dosage of metformin (Glucophage) should be maintained at 1500 mg/day from beginning of the run-in period to completion of open-label treatment.
**Main Inclusion/Exclusion Criteria**	**Inclusion criteria**<br>1. Male or female, aged 18–75 years old (inclusive) when informed consent is signed.<br>2. Subjects diagnosed with T2DM according to the World Health Organization diagnostic criteria (1999) at screening and treated with metformin≥ 1500mg/day constantly for at least 12 consecutive weeks in addition to dietary and exercises intervention.

3. Glycosylated hemoglobin (HbA1c) ≥ 7.5% and ≤ 10.0% at screening.

4. Body mass index (BMI)> 18.5 kg/m^2 and < 35.0 kg/m^2 at screening.

5. Willing to follow the same diet and exercise interventions throughout the study, to take medications and meals on time per study protocol requirements, and to conduct self-monitoring of blood glucose (SMBG) timely and keep records.

6. Willing to sign the written informed consent form and comply with the study protocol.

Exclusion Criteria

Subjects cannot be enrolled if any of the following criteria is met (all laboratory parameters are examined in the central laboratory).

Exceptions for Target Disease

1. Any antidiabetic therapy other than metformin within 12 weeks before screening.
2. Received insulin therapy for more than 30 days within 1 year before screening.

3. History of severe hypoglycemia (requiring external assistance for recovery) or recurrent hypoglycemia (such as experience of 3 or more times of hypoglycemia <=3.9 mmol/L or hypoglycemia symptoms within 1 month) without contributing factors within 3 months before screening.

4. Fasting C-peptide <1.0 ng/ml (0.33 nmol/L) at screening.

5. Medical history of diabetic ketoacidosis, diabetes lactic acidosis or hyperosmotic nonketotic diabetic coma.

6. Subjects clinically diagnosed with type Ⅰ diabetes mellitus, or diabetes mellitus caused by pancreatic damage or other special types of diabetes.

Medical History and Concomitant Disease

7. Severe cardio-cerebrovascular diseases defined as:

   a) Medical history of myocardial infarction, coronary angioplasty or coronary artery bypass grafting, valvular disease or repaired, clinically significant unstable arrhythmia, unstable angina, transient ischemic attack, or cerebrovascular accident within 6 months before screening.

   b) Class III or IV congestive heart failure according to New York Heart Association (NYHA) Classification.

8. Unstable or rapidly progressive kidney diseases.

	9. Active liver diseases at screening.<br><br>10. Diagnosed mental illness.<br><br>11. Hemoglobinopathies (such as sickle cell anemia, thalassemia, or sideroblastic anemia).<br><br>12. The immunocompromised subjects such as subjects who underwent organ transplantation or subjects diagnosed with human immunodeficiency virus infection (HIV).<br><br>13. Any type of malignant tumor (no matter cured or not).<br><br>14. Any endocrine system diseases related to blood glucose (such as hyperthyroidism, acromegaly, Cushing's syndrome) or immune diseases which are unstable and need medical interventions and decided by the Investigator as not being suitable for this study.<br><br>15. History of drug abuse.<br><br>16. Received oral or injected corticosteroids treatment within 1 year before screening.<br><br>17. Alcohol intake> 2 units a day, or > 14 units a week.<br><br>One unit of alcohol equivalent to 150 mL of wine, 350 mL of beer, 100 mL (<=17%) or 80 mL (>17% and <=24%) of low-alcohol liquor, or 50 mL of high-alcohol liquor (>24%).<br><br>**Physical Examination and Laboratory Results**<br><br>18. Significant abnormal liver function at screening, defined as alanine aminotransferase (ALT)> 2.5×upper limit of normal (ULN), or aspartate aminotransferase (AST)> 2.5×ULN or serum total bilirubin (TBiL)> 1.5×ULN（refer to Appendix 6）.<br><br>19. Serological evidence of hepatitis virus infection at screening: positive hepatitis virus A IgM antibody (IgM anti-HAV), or positive hepatitis B surface antigen (HBsAg) and IgM antibody to hepatitis B core antigen (IgM anti-HBc) (to exclude subjects with acute hepatitis virus B infection), or positive hepatitis C virus antibody (anti-HCV).<br><br>20. Estimated glomerular filtration rate (eGFR)< 60 mL/min/1.73m^2 at screening.<br><br>21. Triglyceride> 5.7 mmol/L at screening.<br><br>22. Anemia of any causes, which is defined as: hemoglobin < 12.0 g/dL (120 g/L) for male and hemoglobin < 11.0 g/dL (110 g/L) for female at screening.

	23. Abnormal laboratory results that may interfere with the safety evaluation, as judged by the Investigator.<br><br>24. Abnormal electrocardiogram (ECG) results that may affect the safety evaluation or need medical interventions, as judged by the Investigator.<br><br>25. Subjects with uncontrolled hypertension (systolic blood pressure ≥ 160 mmHg or diastolic blood pressure ≥ 100 mmHg at screening) to antihypertensive treatment with stable doses for at least 4 weeks at screening.<br><br>**Allergies and Adverse Drug Reactions**<br><br>26. Subjects who are potentially allergic or do not tolerate study medications, as considered by the Investigator.<br><br>**Prohibited Therapies and/or Medications**<br><br>27. Subjects who refuse to only take the antidiabetic medications specified by the study protocol.<br><br>28. Concomitant treatment with potent or moderate CYP3A4/5 inducers or inhibitors (see Section 4.4.2).<br><br>**Sex and Reproductive Status**<br><br>29. Women who are pregnant or intend to be pregnant during the study.<br><br>30. Women in breastfeeding.<br><br>31. Women with childbearing potential and refuse to take high-efficient or clinically accredited contraceptive methods throughout the study and within 1 month after the last dose of study medications.<br><br>**Other Exclusion Criteria**<br><br>1. Any disease or medical condition that may prevent the subjects from completing all study procedures considered by the Investigator.<br><br>2. Subjects who need to receive some other treatments that may potentially affect the interpretation of efficacy and safety data of the study, as decided by the Investigator.<br><br>3. Based on Investigator’s judgment, Subjects who are unable to follow the study protocol, e.g., unable to persist on dietary and exercise therapy during the study, to take medications and meals timely per study protocol, and to conduct self-monitoring of blood glucose (SMBG) and make record timely etc.<br><br>4. Subjects who participated in any other clinical trials or took any other study drug

within 30 days before screening.

Randomization Criteria (laboratory tests for randomization are conducted on Day -5 [± 2 days])

At Visit 4 (randomization visit), the following criteria will be re-evaluated to confirm eligibility. Subjects who meet the following randomization criteria are eligible to be randomized.

Inclusion Criteria

1. HbA1c ≥ 7.5% and ≤ 10.0% at pre-randomization visit.
2. Fasting blood glucose (FPG) > 7.0 mmol/L and < 13.3 mmol/L at pre-randomization visit.

Exclusion Criteria

3. History of severe hypoglycemia (requiring external assistance for recovery) or recurrent hypoglycemia (such as experience of 3 or more times of hypoglycemia <=3.9 mmol/L or hypoglycemia symptoms within 1 month) without contributing factors within 3 months in run-in period.
4. Body weight change (weight gain or loss) ≥ 10% at randomization compared with that at screening.
5. Poor adherence to treatment during run-in period (< 80% or > 120%).
6. Abnormal liver function with clinical significance occurred in run-in period, defined as ALT > 2.5×ULN, or AST > 2.5×ULN or TBiL > 1.5×ULN at pre-randomization visit (refer to Appendix 6).
7. eGFR< 60mL/min/1.73m^2 at pre-randomization visit.
8. Triglyceride> 5.7mmol/L at pre-randomization visit.
9. Anemia of any causes, defined as hemoglobin < 12.0 g/dL (120 g/L) for male, and hemoglobin < 11.0 g/dL (110 g/L) for female at pre-randomization visit.
10. Subjects with uncontrolled hypertension (systolic pressure ⩾ 160mmHg or diastolic pressure ⩾ 100 mmHg at randomization visit and with stable dose of antihypertensive treatment in run-in period.
11. Abnormal laboratory results that may affect the safety evaluation of the study, as judged by the Investigator.
12. Abnormal ECG results that may affect the safety evaluation of the study or need

	medical interventions, as judged by the Investigator.<br>13. The Investigator reconfirm the medical history and concurrent disease occurred between screening and randomization visits (refer to the exclusion criteria at screening visit).
**Study Hypothesis**	**The HbA1c reduction after 24-week treatment with HMS5552 75 mg BID combined with metformin is significantly superior to that with placebo combined with metformin in subjects with T2DM inadequately controlled with stable treatment of metformin.**
**Efficacy Endpoints**	**Primary Efficacy Endpoint**<br>In subjects with T2DM inadequately controlled with stable treatment of metformin, to compare the change of HbA1c from baseline after HMS5552 75 mg BID combined with metformin treatment at 1500 mg/day with placebo BID combined with metformin treatment at 1500 mg/day after 24-week double-blind treatment.<br>**Secondary Efficacy Endpoints**<br>To compare HMS5552 75 mg BID combined with metformin at 1500 mg/day with placebo BID combined with metformin at 1500 mg/day after 24-week double-blind treatment in subjects with T2DM inadequately controlled with stable treatment of metformin:<br>1. Change of 2-hour postprandial plasma glucose (2h-PPG) from baseline<br>2. Change of FPG from baseline<br>3. HbA1c response rate: the proportion of subjects whose HbA1c < 7.0%<br>4. Change of HbA1c from baseline at each visit, except Week 24.
**Safety Endpoints**	• Adverse events throughout the study<br>• Hypoglycemic events<br>• Physical examination<br>• Vital signs<br>• 12-lead ECG<br>• Clinical laboratory examinations (hematology, biochemistry and urinalysis)

**Investigational Products**	**Basic drug**<br>Metformin (Glucophage). 1500 mg/day, taken according to doctor's advice. The administration method is kept consistent throughout the study.<br>**Study drug**<br>HMS5552 tablets, 75 mg BID, one tablet each time, prefer to take before meals.<br>**Control drug**<br>Placebo tablets, BID, one tablet each time, prefer to take before meals.
**Usage and dosage of Study Medications**	**Run-in period**<br>Basic drug + control drug, continue to take for 4 weeks<br>**Double-blind treatment period**<br>Basic drug + study drug or control drug, continue to take for 24 weeks<br>**Open-label treatment period**<br>Basic drug + study drug, continue to take for 28 weeks<br>**Safety follow-up period**<br>None
**Statistical Considerations**	**Sample size**<br>1. This study plans to recruit 750 subjects. Considering 20% drop-out rate, no more than 938 subjects will be recruited. The subjects are randomized at a ratio of 1:1 to treatment group (HMS5552 75 mg BID + metformin 1500 mg/day) and control group (placebo BID + metformin 1500 mg/day), i.e. no more than 469 subjects and 469 subjects are randomized to treatment group and control group.<br>2. Assessment: Based on the planned sample size and the randomization ratio (750 subjects, treatment group: control group = 1:1), the study has 95.4% power to detect the efficacy in treatment group is superior to the control group with the assumption of type I error (alpha) at 0.05 (two sided), if the within-group standard deviation for primary efficacy endpoint (change of HbA1c from baseline after 24-week treatment) is 1.5% and the between-group difference (treatment group-control group) is -0.4%.

	**Statistical analysis**<br><br>The primary endpoint of this study is the change of HbA1c from baseline to double-blind treatment completion (Week 24). Comparison between treatment and control group will be made at a significant level of 0.05 (two-sided). The mixed model with repeated measurements is mainly used for this primary endpoint analysis. Subjects included in the analysis should have baseline measurement of the primary efficacy endpoint and at least one post treatment measurement during double-blind treatment period. The factors of the model include groups, visits, the interaction between groups and visit, sites, disease duration and baseline HbA1c value. An unstructured covariance structure is preferred for the primary analysis. The Statistical Analysis Plan prepared separately provides the details for other covariance structures in case the preferred covariance model does not converge. The primary analysis provides point estimation and 95% confidence interval for the difference in the change of HbA1c from baseline between groups (treatment group-control group).
**Population Pharmacokinetic/ Pharmacodynamic Analysis (PopPK/PD)**	The PopPK/PD model for HMS5552 is updated by using the NONMEM/PIRANA/R software based on the current HMS5552 PopPK/PD model and PK/PD data in Chinese subjects with T2DM after HMS5552 treatment. The quantitative relationship between HMS5552 concentration and HbA1c level is established. Systemic quantitative analysis is conducted on the influences of various factors on HMS5552 PK/PD characteristics, e.g., demographics, T2DM duration, concomitant medications etc..

Study Flow Chart

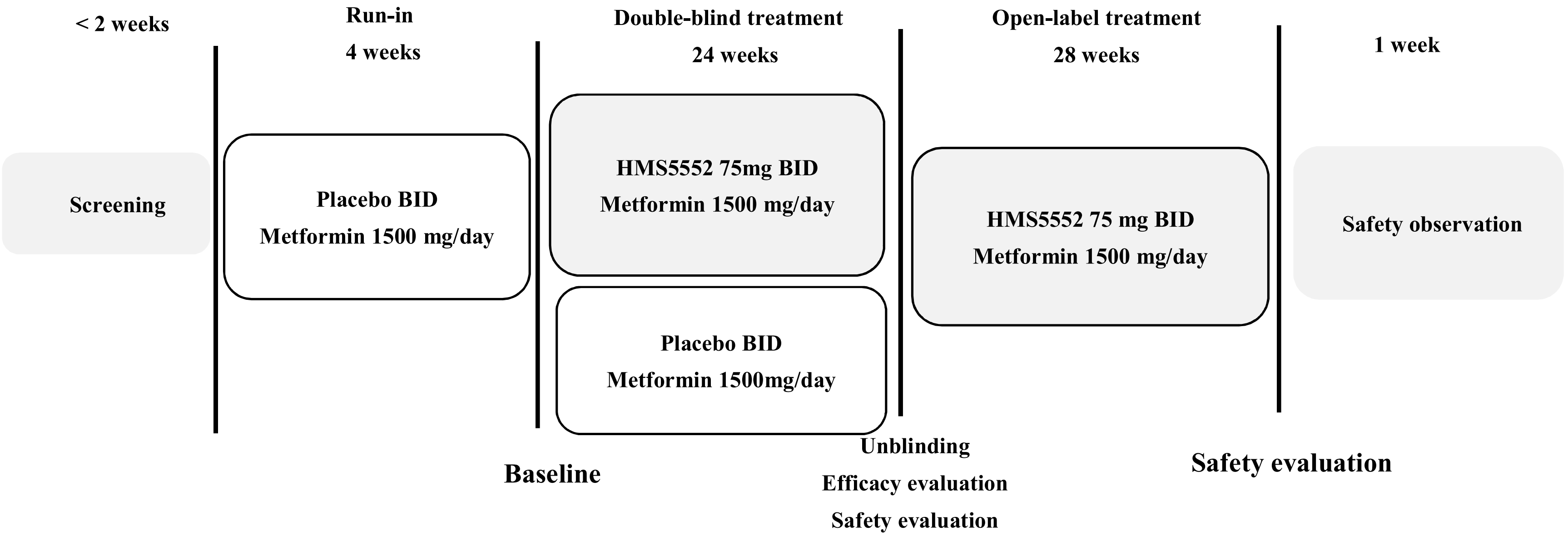

Schedule of Assessments

	Screening	Single blind run-in period[1]		Randomization	Double-blind treatment period						Open-label treatment period					Safety observation
Visit number	Visit 1	Visit 2	Visit 3	Visit 4	Visit 5	Visit 6	Visit 7	Visit 8	Visit 9	Visit 10[2]	Visit 11	Visit 12	Visit 13	Visit 14	Visit 15[3]	Visit 16[4]
Visit time	Week -6 to -4	Week -4	Day-5 ± 2 days	Day 1	Week 4 ± 4 days	Week 8 ± 4 days	Week 12 ± 4 days	Week 16 ± 4 days	Week 20 ± 4 days	Week 24 ± 4 days	Week 28 ± 4 days	Week 34 ± 6 days	Week 40 ± 6 days	Week 46 ± 6 days	Week 52 ± 6 days	Week 53 ± 2 days
**Study Procedure**																
Informed consent	X															
Demographics	X															
Medical history and prior medications	X			X												
Inclusion and exclusion criteria	X			X												
Concomitant medications	X	X	X	X	X	X	X	X	X	X	X	X	X	X	X	X
Randomization				X												
Adverse events		X	X	X	X	X	X	X	X	X	X	X	X	X	X	X
Physical examination[5]	X			X	X	X	X	X	X	X	X	X	X	X	X	
Vital signs [6]	X			X	X	X	X	X	X	X	X	X	X	X	X	
12-Lead ECG	X			X	X	X	X	X	X	X	X	X	X	X	X	
**Laboratory Tests (in central laboratory)**																
HbA1c	X		X	X	X	X	X	X	X	X	X	X	X	X	X	
FPG	X		X	X	X	X	X	X	X	X	X	X	X	X	X	
Hematology[7]	X		X	X	X	X	X	X	X	X	X	X	X	X	X	
Urinalysis[8]	X		X	X	X	X	X	X	X	X	X	X	X	X	X	
Blood biochemistry[9]	X		X	X	X	X	X	X	X	X	X	X	X	X	X	
Virology test[10]	X															
Pregnant test[11]	X	X	X	X	X	X	X	X	X	X	X	X	X	X	X	
MMTT[12]				X			X			X					X	
Biomarker[13]	X			X			X			X					X	
PK sampling[14]					X		X			X					X	
DNA sample[15]				X												
**Instructions / Suggestions**																
Health education[16]	X	X	X	X	X	X	X	X	X	X	X	X	X	X	X	
Glucometer distribution		X														
SMBG check[17]			X	X	X	X	X	X	X	X	X	X	X	X	X	
Subject diary		X		X	X	X	X	X	X	X	X	X	X	X		
**Study Medication**																
Drug distribution		X		X	X	X	X	X	X	X	X	X	X	X		
Review of drug usage and recycling[18]				X	X	X	X	X	X	X	X	X	X	X	X	

ECG: electrocardiogram. FPG: fasting plasma glucose. HbA1c: glycosylated hemoglobin. MMTT: mixed-meal tolerance test. SMBG: self-monitoring of blood glucose.

Note: a. All laboratory sampling must be collected under fasting before dosing (except for MMTT), suggested time: 7:00 to 10:00 a.m. b. MMTT is suggested to start between 7:00 and 9:00 a.m. c. The schedule of visit window is based on normal working time. In case of the National Day, Spring Festival and other holidays, the Investigator can arrange for administration of the study medications accordingly and reschedule the next visit to a time closest to the visit scheduled per study protocol. Causes for early/late visit will be recorded on eCRF and although it is not considered as protocol deviation in this particular case.

1. Single-blind placebo run-in period lasts from Day -28 days to Day -1 (inclusive).
2. The double-blind treatment period (Visit 10) ends when subjects complete all the 24-week treatment. If early withdrawal occurs in the double-blind treatment period, a termination visit needs to be conducted for the patient within 7 days (the 7th day included) after the last dose of study medications. In addition, a telephone follow-up for safety is conducted within 1 week after the last dose of study medications. If administration of the study medications has been stopped by the subjects for ≥ 7 days before early withdrawal, a termination visit needs to be completed as much as possible. The tests in the termination visit are the same as Visit 15.
3. The open-label treatment period (Visit 15) ends when subjects finish all the 52-week treatment. If early withdrawal occurs in the open-label treatment period, a termination visit needs to be completed within 7 days (the seventh day included) after the last dose of study medications. In addition, a telephone follow-up for safety is conducted within 1 week after the last dose of study medications. If administration of the study medications has been stopped by the subjects for ≥ 7 days before early withdrawal, a termination visit needs to be completed as much as possible. The tests in the termination visit are the same as Visit 15. If the study medications are already stopped at the termination visit, the MMTT can be cancelled.
4. All study medications, including metformin, are stopped in the safety observation period. Visit 16 is conducted by telephone at which adverse events and concomitant medications occurring in the safety observation period are recorded. If there is any adverse event that has occurred during the treatment period and is still ongoing, the investigator should arrange the follow-up visit based on the need of adverse event during the observation period and carry out corresponding examinations.
5. Physical examinations: general appearance, head and neck, chest (including heart and lungs), abdomen, limbs, etc.
6. Vital signs: for Visit 1, blood pressure, pulse rate, breath rate, body temperature (axillary temperature), height and weight. For other visits, all parameters except height are checked.
7. Hematology: hemoglobin, hematocrit, erythrocytes count, leukocytes count, neutrophils, basophils, eosinophils, monocytes, lymphocytes, and platelet count.
8. Urinalysis: pH, specific gravity, protein, glucose, erythrocytes, leukocytes, and ketone bodies.
9. Blood biochemistry: plasma glucose, total bilirubin, alanine aminotransferase, aspartate aminotransferase, gamma-glutamic acid, alkaline phosphatase, albumin, total protein, lactate dehydrogenase, urea nitrogen, creatinine, uric acid, sodium, potassium, chlorine, calcium, creatine kinase, creatine kinase isoenzyme, total cholesterol, high-density lipoprotein cholesterol, low-density lipoprotein cholesterol, and triglyceride.
10. Virology test: hepatitis A virus IgM antibody, hepatitis B surface antigen (HBsAg), hepatitis B surface antibody (anti-HBs), IgM antibody to hepatitis B core antigen (IgM anti-HBc), hepatitis C virus antibody (anti-HCV), human immunodeficiency virus (HIV) antibodies.

11. Pregnancy test (only for females with childbearing potential): From Visit 1 to Visit 15, blood pregnancy test must be performed in the central laboratory. During the study, if any amenorrhea or irregular menstruation is reported for any female subject at any time, a blood pregnancy test should be performed in the central laboratory immediately to exclude or validate pregnancy. Drug administration is stopped immediately once a positive result is shown.
12. MMTT: The blood samples for evaluating plasma glucose, insulin and C-peptide are collected before and 120 minutes after the instant noodles standard meal being taken, which is provided by the Sponsor. The standard meal should be taken before drug administration at Visit 4. At Visits 7, 10 and 15, the standard meal is taken after drug administration.
13. Biomarker: including fasting insulin and C-peptide. At Visit 1, only C-peptide is tested and evaluated as one of the exclusion criteria.
14. Population pharmacokinetics: At Visit 5, blood samples are collected before drug administration. At Visits 7, 10 and 15, blood sampling schedule is the same as MMTT. The time of blood sampling and the time for the closest investigational drug administration before blood sampling are recorded in detail.
15. DNA sampling. At Visit 4, DNA samples are collected (optional).
16. Diabetes education and health/exercise guidance: The Investigator fully assesses the life and disease status for each subject. If any inappropriate lifestyle is identified (e.g. eating or drinking too much in holidays) for any subject, the diabetes education and health/exercise guidance should be given to him/her timely and repeatedly.
17. SMBG examination: For each subject, fingertip blood glucose under fasting or postprandial is required to be monitored at least twice a week at home. If suspicious hypoglycemia or hyperglycemia events occur, additional blood glucose tests should be conducted and the results are recorded in subject diaries. At each visit, the Investigator should review the subject diaries carefully then make suggestions on drug administration and blood glucose monitoring for next stage.
18. Review of drug usage and recycling: The Investigator should review the drug compliance record from Visit 2 to Visit 4 (run-in period) and from Visit 4 to Visit 15 (treatment period). Queries should be made if any problem is found. At Visit 4, drug compliance is evaluated for the run-in period. Guidance should be given if any subject does not follow the drug administration specified in the study protocol.

Contents

Protocol Signature Page **4**
Protocol Signature Page **5**
Study Protocol Synopsis **6**
Study Flow Chart **14**
List of Abbreviations **22**
1 Background **24**
1.1 INTRODUCTION OF DISEASE 24
1.2 DRUG BACKGROUND 24
1.3 REFERENCES FOR THE PRECLINICAL STUDIES AND PRELIMINARY CLINICAL STUDIES OF HMS5552 ON TYPE 2 DIABETES MELLITUS 26
1.4 THE RATIONALE OF THE STUDY 27
1.5 DOSE SELECTION, BENEFIT/RISK ASSESSMENT, AND ETHICAL REVIEW 27
2 Study Objectives **28**
2.1 THE PRIMARY OBJECTIVE 28
2.2 THE SECONDARY OBJECTIVES 28
3 Study Design **28**
3.1 STUDY ENDPOINTS 28
3.1.1 Primary Efficacy Endpoint *28*
3.1.2 Secondary Efficacy Endpoints *28*
3.1.3 Safety Assessment *29*
3.2 STUDY DESIGN AND RATIONALE 29
3.3 SUBJECT ENROLLMENT, RANDOMIZATION, AND TREATMENT 30
3.3.1 Randomization Method *30*
3.3.2 Run-in Period *30*
3.3.3 Double-Blind Treatment Period *31*
3.3.4 Procedures for incorrect enrollment, randomization, or drug administration *32*
3.3.5 Blinding *32*
3.4 PREPARATION FOR THE STUDY 32
3.5 STUDY PROCEDURES AND STAGES 33
3.5.1 Visit 1: Screening (Day -42 to -29) *33*
3.5.2 Visit 2: Single-Blind Placebo Run-in Period (Day -28) *34*
3.5.3 Visit 3: Pre-Randomization Visit (Day -5 ± 2) *35*
3.5.4 Visit 4: Randomization Visit (Day 1) *35*
3.5.5 Visit 5: Treatment Visit (Day 28 ± 4) *37*
3.5.6 Visit 6: Treatment Visit (Day 56 ± 4) *38*
3.5.7 Visit 7: Treatment Visit (Day 84 ± 4) *39*
3.5.8 Visit 8: Treatment Visit (Day 112 ± 4) *40*
3.5.9 Visit 9: Treatment Visit (Day 140 ± 4) *40*
3.5.10 Visit 10: Termination Visit for Double-blind Treatment (Day 168 ± 4) *40*
3.5.11 Visit 11: Open-Label Treatment Visit (Day 196 ± 4) *41*

3.5.12 Visit 12: Open-Label Treatment Visit (Day 238 ± 6) *41*
3.5.13 Visit 13: Open-Label Treatment Visit (Day 280 ± 6) *41*
3.5.14 Visit 14: Open-Label Treatment Visit (Day 322 ± 6) *41*
3.5.15 Visit 15: Termination Visit for End of Open-Label Treatment (Day 364 ± 6) *42*
3.5.16 Visit 16: Follow-up Visit for Safety (Day 371 ± 2) *43*
3.6 STUDY MATERIALS PROVIDED TO SUBJECTS 43
4 Study Population 43
4.1 INCLUSION CRITERIA 43
4.2 EXCLUSION CRITERIA 44
4.3 RANDOMIZATION CRITERIA (LABORATORY TESTS AND WILL BE CONDUCTED ON DAY -5 [± 2 DAYS] PRIOR TO THE RANDOMIZATION)47
4.4 CONCOMITANT MEDICATION AND POST-STUDY TREATMENT 48
4.4.1 General Instructions on Study Treatment *48*
4.4.2 Prohibited and Restricted Medications *48*
4.4.3 Contraception *49*
4.4.4 Diet Control and Exercise Intervention *49*
4.4.5 Post-Study Treatment *49*
4.5 DISCONTINUATION OF STUDY TREATMENT, WITHDRAWAL, OR TERMINATION OF STUDY 50
4.5.1 Withdrawal Due to Hypoglycemia *50*
4.5.2 Withdrawal due to Hyperglycemia *52*
4.5.3 Withdrawal due to Abnormal Liver Function *52*
4.6 PROCEDURES FOR SUBJECT WITHDRAWAL 53
4.7 LOST TO FOLLOW-UP 53
5 Study Drugs 54
5.1 STUDY MEDICATIONS 54
5.1.1 Drug Name, Strength, Formulation *54*
5.1.2 Packaging and Labelling *54*
5.1.3 Study Medication Regimen and Packaging *55*
5.2 STORAGE CONDITION 57
5.3 DRUG DISPENSING AND COUNTING 57
5.4 DRUG RETURN AND DESTRUCTION 58
5.5 DRUG COMPLIANCE 58
5.6 EXTENT OF EXPOSURE 58
6 Study Endpoints 58
7 Safety Assessments 59
7.1 ADVERSE EVENT (AE) 59
7.1.1 Serious Adverse Event (SAE) *60*
7.1.2 Persistent or Recurrent AEs *60*
7.1.3 AEs Related to Examinations and Laboratory Tests *61*
7.2 RECORDING OF AES 61
7.2.1 Terms of AEs *61*
7.2.2 Recording of AE Time and Collection Period for AE *61*
7.2.3 AE Variables *62*

7.2.4 AE Grading ... *62*
7.2.5 AE Outcome ... *63*
7.2.6 Causality Assessment ... *63*
7.3 SERIOUS ADVERSE EVENT REPORTING ... 64
7.4 HYPOGLYCEMIA ... 64
7.5 HY'S LAW ... 65
7.6 POST-STUDY AE REPORTING ... 65
7.7 LACK OF EFFICACY OR AGGRAVATION OF T2DM ... 65
7.8 LABORATORY SAFETY ASSESSMENTS ... 65
7.9 PHYSICAL EXAMINATIONS ... 66
7.10 12-LEAD ECG ... 67
7.11 VITAL SIGNS ... 67
7.12 PREGNANCY ... 68
7.13 OVERDOSE ... 68
8 Data Management ... **68**
8.1 DATA TRACEABILITY, COMPLETION, AND TRANSFER OF ECRF ... 68
8.2 DATABASE DESIGN ... 69
8.3 DATA ENTRY ... 69
8.4 DATA VALIDATION ... 69
8.5 BLINDED DATA REVIEW AND UNBLINDING ... 69
8.6 DATABASE LOCK ... 70
9 Statistical Analysis ... **70**
9.1 SAMPLE SIZE DETERMINATION ... 70
9.2 ANALYSIS POPULATION SETS ... 71
9.3 STATISTICAL METHODS ... 71
9.3.1 Demographics and Baseline Characteristics ... *71*
9.3.2 Efficacy Analysis for Primary Endpoint ... *72*
9.3.3 Efficacy Analysis for Secondary Endpoints ... *72*
9.3.4 Safety Analysis ... *73*
9.3.5 Biomarker Analysis ... *73*
9.3.6 Sensitivity Analysis ... *73*
9.3.7 Interim Analysis ... *74*
10 Population PK/PD （PopPK/PD）Analysis ... **74**
11 Trial Management ... **74**
11.1 DECLARATION ... 74
11.2 ETHICS COMMITTEE ... 74
11.3 SOURCE DOCUMENT VERIFICATION ... 75
11.4 QUALITY CONTROL AND QUALITY ASSURANCE ... 75
11.5 SUBJECT INFORMED CONSENT ... 75
11.6 PROTOCOL AMENDMENT ... 75
11.7 MONITORING ... 76
11.8 CONFIDENTIALITY AGREEMENT AND PATIENT PRIVACY ... 76
12 Publication ... **76**

13 Documents Archiving 77
14 Appendix 78
15 References 92

List of Abbreviations

ADA	American Diabetes Association
AE	adverse event
ALP	alkaline phosphatase
ALT	alanine aminotransferase
AST	aspartate aminotransferase
BID	twice a day
BMI	body mass index: weight[kg]/ (height [m])^2
BUN	blood urea nitrogen
CDISC	Clinical Data Interchange Standards Consortium
CFDA	China Food and Drug Administration
CRO	Contract Research Organization
DPP-4	dipeptidyl peptidase-4
EASD	European Association for Diabetes Study
EC	ethics committee
ECG	electrocardiography
EDC	electronic data capture
e.g.	for example
eCRF	electronic case report form
eGFR	estimated glomerular filtration rate
FAS	full analysis set
FDA	Food and Drug Administration
FPG	fasting plasma glucose
GCP	Good Clinical Practice
GK	glucokinase
GKA	glucokinase activator
GLP-1	glucagon-like peptide-1
GMP	Good Manufacturing Practices
G-6-P	glucose-6-phosphate
HbA1c	glycosylated hemoglobin
HIV	human immunodeficiency virus
HK	hexokinase
IB	investigator's brochure
ICF	Informed Consent Form
ICH	International Conference on Harmonization
IWRS	interactive web response service

i.e.	that is to say
ISF	Investigator Site File
kg	kilogram
LDH	lactate dehydrogenase
LOCF	last observation carried forward
NYHA	New York Heart Association
MAD	multiple ascending dose
MedDRA	Medical Dictionary for Regulatory Activities
mmHg	millimeter of mercury
MMRM	mixed-effect model repeated measure
MMTT	mixed-meal tolerance test
MODY-2	maturity-onset diabetes of the young type-2
nH	Hill coefficient
OR	odds ratio
PD	pharmacodynamics
pH	potential of hydrogen
PK	pharmacokinetics
PPG	post-prandial plasma glucose
PPS	per protocol set
QA	quality assurance
QC	quality control
QD	once a day
SAD	single ascending dose
SAE	serious adverse event
SMBG	self-monitoring of blood glucose
SS	safety set
SOP	Standard Operation Procedure
TBiL	total bilirubin
TG	triglyceride
T2DM	type 2 diabetes mellitus
UKPDS	United Kingdom Prospective Diabetes Study

1 Background

1.1 Introduction of Disease

Diabetes mellitus is a chronic metabolic disease characterized by hyperglycemia, and requires continuous medical treatments. Diabetes mellitus usually has the concomitant diseases of ischemic heart disease, cerebrovascular and peripheral vascular diseases, which are major causes of early death and high rate of disability, as well as the common causes for blindness and renal failure.

Type 2 diabetes mellitus (T2DM) accounts for approximate 90% to 95% of all diagnosed diabetes cases and is characterized by hyperglycemia caused by decreased insulin secretion, increased insulin resistance and hepatic glucose production [1]. With the rapid growth of China's economy, the incidence of diabetes has also increased. A national epidemiological survey in 2008 showed that the prevalence of diabetes was 9.7%. Two years later, another investigational result showed that the prevalence of diabetes in adults above 18 years old increased to 11.6%, with an absolute number of 1.14 billion. China has become the country with the largest number of patients with diabetes mellitus [2].

Usually, diet control and physical exercise are the major strategies for treating slightly elevated blood glucose in patients with T2DM. If diet control and physical exercise are still insufficient to lower blood glucose, oral antidiabetic drugs should be given. Various antidiabetic drugs have been approved for the treatment of T2DM subjects, including metformin, sulfonylureas, alpha-glucosidase inhibitors, dipeptidyl peptidase 4 (DDP-4) inhibitors, glucose-like peptide-1 (GLP-1) receptor agonists and insulin, etc. These drugs exert their effects by promoting insulin secretion, improving insulin sensitivity of peripheral tissue or other mechanisms [3].

Metformin is currently the first-line therapy listed in many international guidelines and national clinical guidelines[2][3]. However, monotherapy of many antidiabetic drug, including metformin, usually fails to achieve the target level of glycosylated hemoglobin (HbA1c). After starting a monotherapy, nearly 50% of diabetic subjects need to take a second antidiabetic drug. There is evidence that combination therapy using antidiabetic drugs with different mechanisms of action can improve glycemic control. Therefore, it is clinically urgent to develop new antidiabetic drugs that have different mechanisms of action that can be used as monotherapy or in combination with another antidiabetic drug.

1.2 Drug Background

Glucokinase (GK) is one of the four subtypes of hexokinase (HK) and catalyzes at the first step of glucose metabolism, by which glucose is phosphorylated and converted to 6-phosphate glucose (G-6-P) in glucose metabolism. The GK is mainly expressed in glucose-sensitive and metabolic active tissues including the pancreas, liver, intestine and brain. As a glucose sensor, the GK plays a key role in the glucose homeostasis in the body. Abnormal GK activity leads to glucose metabolism disorder, causing diabetes and related complications. Mutations in GK gene

can cause severe symptoms of diabetes. For example, mutations of function loss lead to adolescent onset of adult type 2 diabetes (MODY-2) and permanent neonatal diabetes mellitus. In contrast, gain of function or gene activation mutations cause hypoglycemia and hyperinsulinemia. In addition, decrease in GK activity exists in diabetic patients. For instance, further study on the mechanism of T2DM showed that decrease of pancreatic β cell function is associated with the change in GK quantity and activity. Decrease in liver GK activity may be one mechanism leading to insulin resistance[4].

In recent years, GK has become a significant target in the development of new drugs for diabetes because of its pivotal role in glucose homeostasis. The development of small molecule glucokinase activator (GKA) is very active as well[5].

Roche is the first global pharmaceutical company to bring GKAs into clinical studies[4] [5]. To date, three generations of GKAs (GK1, GK2 and GK3) have been in the clinical development stage, not only showing efficacy and safety in preclinical studies, but also showing clinical efficacy in patients with T2DM.

Based on the chemical structures of GKAs from previous three generations, that for the fourth generation of GKA RO5305552 (HMS5552) underwent relatively large optimization and the main characteristics are as follows:

1. RO5305552 exerts its multiple synergistic activity in a glucose-concentration dependent manner to decrease blood glucose level by improving pancreatic insulin secretion, regulating hepatic glucose transformation, and activating intestinal GK.

2. Activation of GK by RO5305552 reveals a significant synergistic feature in kinetics. By decreasing the $S_{0.5}$ value and increasing the V_{max} value, the affinity of GK with its substrate (glucose) is enhanced with minimum effect on the Hill coefficient (nH). Therefore, the risk of hypoglycemia in clinical is effectively decreased.

3. Compared with other GKAs, RO5305552 is advantageous for the low concentration of metabolites (all plasma steady-state concentration of metabolites in human are < 10%, and the categories of metabolites are similar to those in animals). Thus, for oral administration of RO5305552, the possibilities for toxic and side effects induced by its metabolites are low.

In summary, GKAs can positively influence several major etiological factors for patients with T2DM and are of great potential for drug development. As a GKA, RO5305552 has balanced safety and efficacy features and represents a potential and novel candidate for diabetes therapy[6].

Based on the reasons above, in 2011, Hua Medicine (Shanghai), Ltd. (hereinafter referred to as "Hua Medicine") imported the fourth generation GKA (RO5305552) from Roche. The identification number for RO5305552 in study is HMS5552, including both preclinical and clinical studies. It is expected that through the multiple activation effects of HMS5552, blood glucose control in patients with T2DM can be improved.

The HMS5552 project sponsored by Hua Medicine was approved by China Food and Drug Administration (CFDA) in 2013 (Clinical Trial Permission No.: 2013L01740-2013L01743, with 2013L01740 as a Notification of Approval Opinion). In 2015, Hua Medicine obtained supplemental Clinical Trial Permission (No.: 2015L01892-2015L01895). Currently, a total of four phase I clinical studies have been completed: single ascending dose (SAD) study in health volunteers (ClinicalTrials.gov ID: NCT01952535), multiple ascending dose (MAD) study in subjects with T2DM (ClinicalTrials.gov ID: NCT02077452), continuous 4-week treatment study in subjects with T2DM (ClinicalTrials.gov ID: NCT02386982) and drug-drug interaction between metformin and HMS5552 (USA) in subjects with T2DM (ClinicalTrials.gov ID: NCT02597400). In addition, a double-blind, placebo-controlled phase II clinical study of HMS5552 alone in subjects with T2DM (ClinicalTrials.gov ID: NCT02561338) was completed as well.

1.3 References for the Preclinical Studies and Preliminary Clinical Studies of HMS5552 on Type 2 Diabetes Mellitus

Please refer to the Investigator's Brochure (HMS5552 IB Version 3.0[6]) for more details on the preclinical and clinical studies of HMS5552.

So far, the preclinical and clinical studies for HMS5552 include: single-dose toxicity test (rats and dogs). repeated dose toxicity test (4-week and 13-week repeated dosing in both rats and dogs. 26-week repeated dosing in rats and 39-week repeated dosing in dogs). safety pharmacology test. fertility and early embryonic toxicity test, embryo-fetal developmental toxicity test. genetic toxicity test. preclinical study on pharmacokinetics (PK) and pharmacodynamics (PD) after single and multiple doses of HMS5552. Phase I clinical study on single ascending dose in 60 health volunteers[7][8]. Phase I clinical study of multiple ascending dose (MAD) in 53 subjects with T2DM[9]. Phase I clinical study of continuous 4-week treatment in 24 subjects with T2DM[10]. Phase I clinical study on the interaction between HMS5552 and metformin (USA) in 15 subjects with T2DM[11]. and a 12-week phase II study to evaluate the efficacy and safety of HMS5552 in 258 subjects with T2DM[12].

Studies have shown that the overall safety and tolerability were good in both male and female Chinese subjects after receiving HMS5552 25 mg to 200 mg, twice or once daily and by oral administration. All adverse events (AEs) reported were mild. No serious adverse events (SAEs) and severe hypoglycemia. HMS5552 exhibited dose linear PK characteristics in single dose, and also showed dose-dependent drug concentration increase after steady-state in multiple doses with no obvious accumulation and food effect, and with no sex difference. At steady state, the maximal metabolite in plasma was less than about 5% of original drug. Pharmacodynamics demonstrated the same dose-dependent decrease in fasting plasma glucose and postprandial plasma glucose after steady state in single and multiple doses. After multiple doses, the 24 hours plasma glucose decrease was associated with dosage. No obvious change of insulin and C-peptide level under fasting state was observed. Postprandial insulin and C-peptide secretion increased in a dose-dependent manner.

A phase II, multicenter, randomized, double-blind, placebo-controlled 12-week clinical study evaluated the safety, tolerability, efficacy, and population PK of HMS5552 in subjects with T2DM. The doses used in this study are 50 mg BID, 75 mg BID, 75 mg QD, and 100 mg QD. Data from the phase II study showed that, after treatment for 12 weeks, the subjects receiving 75 mg HMS5552 BID had a significant decrease (1.12%) of HbA1c compared with baseline.

1.4 The Rationale of the Study

At present, metformin is recommended as the first-line treatment for subjects with T2DM by both domestic and international guidelines. The main mechanisms of metformin to control plasma glucose include inhibiting hepatic glucose production and output, reducing glucose absorption, increasing glucose oxidation and metabolism in muscle and adipose tissue, and increasing the sensitivity of peripheral tissue to insulin. However, metformin cannot target all pathogenesis mechanisms of diabetes.

With the progression of diabetes, monotherapy is difficult to achieve good efficacy. In accordance with the results from the United Kingdom Prospective Diabetes Study (UKPDS), the monotherapy of sulfonylureas, insulin, metformin, or other traditional antidiabetic drug cannot maintain glycemic control targets in long-term.

The guidelines suggest the combination with other antidiabetic drugs if plasma glucose is controlled inadequately with metformin monotherapy at maximum dose or maximum tolerated dose[13][14][15][16].

Many studies show that combination of two drugs can bring greater benefits to diabetic patients. It's mainly reflected by the fact that the combination therapy has better efficacy compared with monotherapy. The principle is that the synergistic effect can be reached by targeting different pathogenesis mechanisms and pathways in T2DM.

1.5 Dose Selection, Benefit/Risk Assessment, and Ethical Review

Results from the phase II study showed that glucose-lowering effect was better when HMS5552 dosage reached 75 to 150 mg/day (75 mg BID). The changes of HbA1c from baseline in different groups were: 75 mg QD, -0.39%. 100 mg QD, -0.65%. 50 mg BID, -0.79%. 75 mg BID, -1.12%. Results showed that the efficacy for 50 mg BID and 75 mg BID groups were significantly better than the placebo group, and the efficacy for 75 mg BID group was significantly better than 75 mg QD and 100 mg QD groups. In general, all dosage groups showed good tolerance. No death, SAEs related to study drugs or severe AEs were reported. There were no significant differences in AE incidence across groups. No definitive dose-related AEs were reported [12].

The safety and pharmacodynamic results from the phase II clinical study showed that administration of HMS5552 BID was tolerable and had better efficacy. In the comparison between 50 mg BID and 75 mg BID drug administration, the 75 mg BID showed tolerability and better efficacy meeting clinical needs. Thus, this dosage is expected to have the maximum

benefit/risk ratio for patients. Therefore, 75 mg BID was selected for phase III studies.

In summary, based on the data from preclinical and clinical studies, the benefit/risk ratio is good for subjects who received HMS5552. HMS5552 can be used for the proposed phase III studies.

2 Study Objectives

2.1 The Primary Objective

The primary objective of this study is to assess the change of HbA1c from baseline by comparing the treatment of HMS5552 75 mg BID with placebo in combination with metformin (1500 mg/day) after 24-week treatment in subjects with T2DM whose plasma glucose was inadequately controlled by stable metformin treatment.

2.2 The Secondary Objectives

The secondary objective is to assess the change of HbA1c from baseline by comparing the treatment of HMS5552 75 mg BID with placebo in combination with metformin (1500 mg/day) after 24-week treatment, as well as after the combination of metformin 1500 mg/day with HMS5552 75 mg BID for additional 28-week open-label treatment (Week 52) in subjects with T2DM whose plasma glucose was inadequately controlled by stable metformin treatment:

1. Change of 2h-PPG from baseline at the end of double-blind treatment (Week 24)
2. Change of FPG from baseline at the end of double-blind treatment (Week 24)
3. HbA1c response rate: The proportion of subjects whose HbA1c < 7.0% at the end of double-blind treatment (Week 24)
4. Change of HbA1c from baseline at each visit except Week 24
5. Safety profiles at the end of double-blind treatment (Week 24) and study treatment (Week 52).

3 Study Design

3.1 Study Endpoints

3.1.1 Primary Efficacy Endpoint

To compare the change of HbA1c from baseline after the HMS5552 75 mg BID combined with metformin treatment with the placebo BID combined with metformin treatment after 24-week double-blind treatment in subjects with T2DM and with inadequately controlled plasma glucose by stable metformin treatment

3.1.2 Secondary Efficacy Endpoints

To compare the treatment of HMS5552 75 mg BID group with placebo group both in combination with metformin (1500 mg/day) after 24-week treatment:

1. Change of 2h-PPG from the baseline
2. Change of FPG from the baseline
3. HbA1c response rate: the proportion of subjects whose HbA1c< 7.0%
4. Change of HbA1c from baseline at each visit, except Week 24.

3.1.3 Safety Assessment

- Adverse events (AEs)
- Hypoglycemia events
- Physical examination abnormality
- Vital signs
- 12-lead electrocardiogram (ECG)
- Clinical laboratory examinations (hematology, blood biochemistry and urinalysis)

3.2 Study Design and Rationale

This is a multicenter, randomized, double-blind, placebo-controlled study.

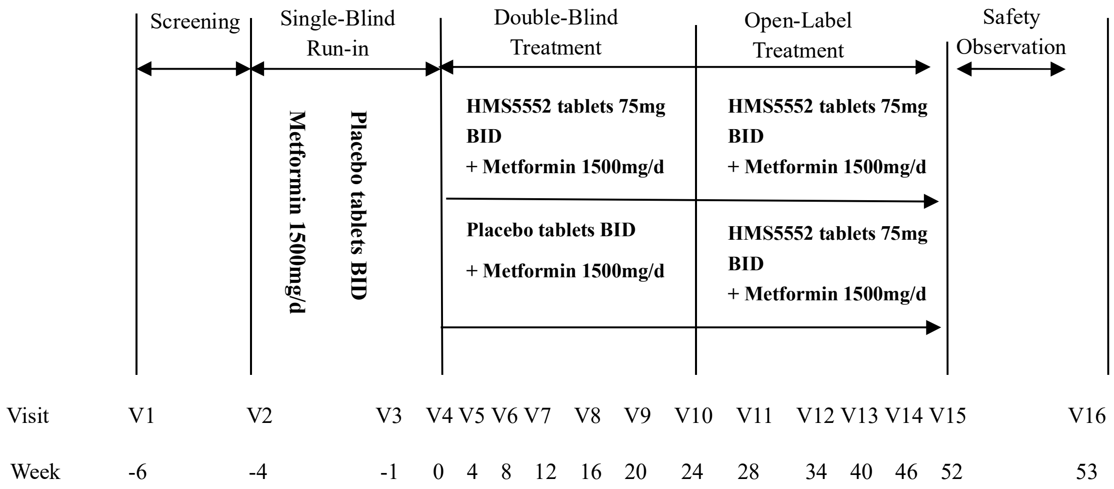

Note: See the Flow Chart of Clinical Trial for visit window details

3.3 Subject Enrollment, Randomization, and Treatment

A total of 750 subjects are planned to enroll in this study. The Investigator:

1. Obtains signed informed consent form (ICF) from potentially eligible subjects before implementing any study procedure.
2. Assigns a unique 8-digit screening number to potentially eligible subjects. The first four digits are for site number and the last four digits are for subject number at the site.
3. Confirms subjects' eligibility according to the inclusion criteria (Section 4.1), exclusion criteria (Section 4.2) and randomization criteria (Section 4.3) defined in this protocol.
4. Assigns a unique randomization number to eligible subject.

Subjects can only be randomized once in this study.

If a subject withdraws from the study, the randomization number can't be reused.

3.3.1 Randomization Method

At screening visit, a unique screening number will be assigned to each subject via the interactive web response service (IWRS) system. This number is used to identify the subject throughout the study and is not used for other subjects.

3.3.2 Run-in Period

In placebo run-in period, sites contact IWRS system to dispense study medications for run-in

period.

3.3.3 Double-Blind Treatment Period

After run-in period, subjects who are eligible for double-blind treatment will be randomized at a ratio of 1:1 to two groups by IWRS system according to the randomization factors (T2DM disease duration and HbA1c level at randomization) entered by the Investigator:

1. Treatment group：HMS5552 75 mg BID + Metformin 1500 mg/day
2. Control group：Placebo BID + Metformin 1500 mg/day

Randomization scheme will be generated from IWRS system (Balance, MedData Solution) and preserved by the IWRS vendor throughout the study. At all visits, study medications are dispensed via the IWRS system along with an identification code (package number). The identification code (package number), which should not be assigned in alphabetic or numerical order and should match the code printed on drug boxes, is provided to the IWRS vendor by drug packaging vendor. The IWRS system is available at any time, 24 hours a day and 7 days a week.

Eligible subjects are able to enter the study. The Investigator assigns each subject an identification code and informs them of the drug administration instructions and time for the first dose of study medications.

Study Period	Study Medications	Drug Name	Morning	Noon	Evening
			Dosage	Dosage	Dosage
Run-in	Basic drug	Metformin (Glucophage) 1500 mg/day	Follow doctor's advice	Follow doctor's advice	Follow doctor's advice
	Control drug	Placebo tablets	1 tablet	NA	1 tablet
Double-blind treatment	Basic drug	Metformin (Glucophage) 1500 mg/day	Follow doctor's advice	Follow doctor's advice	Follow doctor's advice
	Study drug	HMS5552 tablets 75 mg	1 tablet (75mg)	NA	1 tablet (75 mg)
	Control drug	Placebo tablets	1 tablet	NA	1 tablet
Open-label treatment	Basic drug	Metformin (Glucophage) 1500 mg/day	Follow doctor's advice	Follow doctor's advice	Follow doctor's advice
	Study drug	HMS5552 tablets 75 mg	1 tablet (75 mg)	NA	1 tablet (75 mg)

Note: refer to Section 5.1.3 for details about drug administration and package

All study medications (including metformin) are used after Visit 15. From Visit 15 to Visit 16, no antidiabetic drugs can be used. The Investigator can give subjects routine medical treatment after completion of Visit 16. Under special medical condition, subjects can receive other antidiabetic treatment in advance or immediately at end of Visit 15 for safety consideration. The reason should be recorded on electronic case report form (eCRF).

3.3.4 Procedures for incorrect enrollment, randomization, or drug administration

Under any circumstance, noneligible or meeting any of the exclusion criteria subjects should not be enrolled or receive the study treatment.

If noneligible subjects are enrolled or randomized to study by mistake, the subjects will not be withdrawn from study automatically by the Investigator. The decision will be made following a fully discussion among the Investigator, Medidata Information Technology (Shanghai) Co., Ltd. (randomization service vendor) and the Sponsor.

If subjects take the wrong drug because of dispensing error, the Investigator should notify the randomization service vendor immediately. The Investigator should not correct the wrong drug or discontinue the subjects by themselves. A final decision is made after fully discussion with Medidata Information Technology (Shanghai) Co., Ltd., and the Sponsor.

3.3.5 Blinding

Double-blind method is used in the study. Placebo tablets are designed with the size, color, smell and appearance same as the active drug tablets. A unique identification number (drug box serial number) is provided by the study medication packing provider and marked on the label of drug box. By central randomization, the randomization codes are generated by IWRS system based on randomization factors (T2DM disease duration ≤ 3 years or > 3 years. and HbA1c level ≤ 8.5% or > 8.5% at randomization) and the block size. Then, the randomization codes along with the corresponding drug box numbers are provided to subjects who meet the randomization criteria at each visit.

During the study, unauthorized people, including Hua Medicine project members or representatives, site staffs, CRO staffs, the data managers and statisticians do not have access to randomization codes.

3.4 Preparation for the Study

If any of the following requirements is not met, the visit schedule should be re-arranged:

- Subject must be in fasting status (for at least 8 hours) before going to regular scheduled visits (except Visit 2).
- Any high caloric and high fatty diets are prohibited on the day before the scheduled visit.

- Tobacco, alcohol, and caffeine are prohibited within 8 hours before the scheduled visit.
- Study medications need to be taken one day before the scheduled visit (except Visit 1 and Visit 2 or the terminal visit due to discontinuation of study medications by the subjects).
- On the day for the scheduled visit, the study medications for the morning cannot be taken until all the study procedures have been completed., At Visits 7, 10, and 15, the mixed-meal tolerance test (MMTT) is conducted after drug administration in the morning.

3.5 Study Procedures and Stages

For details of study evaluation procedures please refer to the Flow Chat of Clinical Trial. Each evaluation should be completed within scheduled visit/time. Self-Monitoring of Blood Glucose (SMBG) Procedure please refer to Appendix 4.

3.5.1 Visit 1: Screening (Day -42 to -29)

Subjects read the Informed consent form and the Investigator gives verbal explanation about the study requirements and procedures. The subjects need to sign and date the ICF before taking part in the study. On the day of screening visit, subjects must keep fasting status before sample collection (fasting at least 8 hours, suggest no food or soft drink, except water, from 22:00 pm the day before screening).

Study procedures at Visit 1 include (all laboratory tests are conducted in the central laboratory, and all samples are collected in fasting status):

- Informed consent
- Demographics (sex, ethnicity, age, and childbearing potential)
- Medical history, current diseases, and prior medications
- Concomitant medical conditions and medications
- Subjects who are diagnosed with T2DM at screening, and have been continuously treated with metformin≥ 1500 mg/day and have been receiving diet and exercise interventions for at least 12 weeks
- Vital signs (blood pressure, pulse rate, breath rate, body temperature, body weight and height)
- Physical examination: general appearance, head and neck, chest (including heart and lung), abdomen, limbs etc.

- Clinical laboratory tests

  Assessment of diabetes status: HbA1c and FPG.

  Hematology: hemoglobin concentration, hematocrit, erythrocytes, leukocytes, neutrophils, basophils, eosinophils, monocytes, lymphocytes, and platelets.

  Urinalysis: pH, specific gravity, protein, glucose, erythrocytes, leukocytes, and ketone bodies.

  Blood biochemistry: total bilirubin, ALT, AST, gamma-glutamyl transferase, alkaline phosphatase, albumin, total protein, lactate dehydrogenase, urea nitrogen, creatinine, uric acid, sodium, potassium, chloride, calcium, creatine kinase, creatine kinase isoenzyme, total cholesterol, high-density lipoprotein cholesterol, low-density lipoprotein cholesterol, and triglyceride.

  Virology test: A virus IgM antibody (IgM anti-HAV), hepatitis B surface antigen (HBsAg), hepatitis B surface antibody (anti-HBs), IgM antibody to hepatitis B core antigen (IgM anti-HBc), hepatitis C virus antibody (anti-HCV), human immunodeficiency virus (HIV) antibody.

- Biomarker: C-peptide only, evaluated as one exclusion criterion.
- 12-lead ECG.
- Blood pregnancy test (only applicable to females with childbearing potential).
- The inclusion and exclusion criteria are reviewed.

3.5.2 Visit 2: Single-Blind Placebo Run-in Period (Day -28)

The following procedures are completed at Visit 2 (laboratory tests are conducted in the central laboratory):

- Blood pregnancy test (only applicable to females with childbearing potential).
- AEs and concomitant medications are recorded.
- Glucometers and test strips are dispensed. Instructions on SMBG and hyperglycemia and hypoglycemia management are provided (refer to Appendix 4 and Appendix 8 for details).
- Subject diaries are dispensed. Instructions on recording of blood glucose, drug administration and AEs are provided.
- Diabetes education, instructions on diet and exercise are provided.

- Study medications for run-in period are dispensed.

3.5.3 Visit 3: Pre-Randomization Visit (Day -5 ± 2)

The following procedures are completed at Visit 3 (all laboratory tests are conducted in the central laboratory, and all samples are collected in fasting status):

- Confirm that subjects have been in the placebo run-in period for about 3 weeks.
- Clinical laboratory tests:

  Assessment of diabetes status: HbA1c and FPG.

  Hematology: hemoglobin concentration, hematocrit, erythrocytes, leukocytes, neutrophils, basophils, eosinophils, monocytes, lymphocytes, and platelets.

  Urinalysis: pH, specific gravity, protein, glucose, erythrocytes, leukocytes, and ketone bodies.

  Blood biochemistry: total bilirubin, ALT, AST, gamma-glutamyl transferase, alkaline phosphatase, albumin, total protein, lactate dehydrogenase, urea nitrogen, creatinine, uric acid, sodium, potassium, chloride, calcium, creatine kinase, creatine kinase isoenzyme, total cholesterol, high-density lipoprotein cholesterol, low-density lipoprotein cholesterol, and triglyceride.

- Blood pregnancy test (only applicable to females with childbearing potential).
- SMBG status: review SMBG records and provide instructions on SMBG for next stage.
- AEs and concomitant medications are recorded.
- Diabetes education, instructions on diet and exercise.

3.5.4 Visit 4: Randomization Visit (Day 1)

The following procedures are completed at Visit 4 (all laboratory tests are conducted in the central laboratory, samples are collected when subjects are in fasting status):

- Vital signs (blood pressure, pulse rate, breath rate, body temperature, and body weight). Subjects who meet the fourth exclusion criterion are excluded, i.e., body weight change (weight gain or loss) at randomization ≥ 10% compared with that at screening.
- Physical examination: general appearance, head and neck, chest (heart and lungs), abdomen, limbs etc.

- Review study medication usage and recycling. Subject diaries are reviewed. The drug administration records in run-in period are reviewed and confirmed. Instructions are given to subjects who do not take study medications per protocol. Drug compliance in the run-in period is calculated. Subjects who meet the fifth randomization exclusion criterion are excluded, i.e., poor drug compliance during run-in period (< 80% or > 120%).

- 12-lead ECG.

- SMBG status: review SMBG record and provide instructions on SMBG for next stage.

- Reconfirm medical history and previous medications.

- The randomization inclusion and exclusion criteria are reviewed (laboratory test results and from Visit 3 are used as a reference).

- Randomization.

- Baseline laboratory tests (samples are suggested to be collected between 7:00 and 10:00 am) (baseline value)

  Assessment of diabetes status: HbA1c and FPG.

  Hematology: hemoglobin concentration, hematocrit, erythrocytes, leukocytes, neutrophils, basophils, eosinophils, monocytes, lymphocytes, and platelets.

  Urinalysis: pH, specific gravity, protein, glucose, erythrocytes, leukocytes, and ketone bodies.

  Blood biochemistry: total bilirubin, ALT, AST, gamma-glutamyl transferase, alkaline phosphatase, albumin, total protein, lactate dehydrogenase, urea nitrogen, creatinine, uric acid, sodium, potassium, chloride, calcium, creatine kinase, creatine kinase isoenzyme, total cholesterol, high-density lipoprotein cholesterol, low-density lipoprotein cholesterol, and triglyceride.

- Blood pregnancy test (only applicable to females with childbearing potential).

- MMTT: the test must be conducted before taking study medications. Refer to Appendix 3 for MMTT procedures.

- Whole blood samples are collected from some subjects for DNA test.

- Biomarkers.

- AEs and concomitant medications.

- A new subject diary is dispensed.
- Diabetes education, instructions on diet and exercise are provided.
- Study medications are dispensed.

3.5.5 Visit 5: Treatment Visit (Day 28 ± 4)

The following procedures are completed at Visit 5 (all laboratory tests are conducted in the central laboratory, and all samples are collected in fasting status):

- Vital signs (blood pressure, pulse rate, breath rate, body temperature, and body weight).
- Physical examination: general appearance, head and neck, chest (heart and lungs), abdomen, limbs etc..
- Laboratory tests (samples are suggested to be collected between 7:00 and 10:00 am)

  Assessment of diabetes status: HbA1c and FPG.

  Hematology: hemoglobin concentration, hematocrit, erythrocytes, leukocytes, neutrophils, basophils, eosinophils, monocytes, lymphocytes, and platelets.

  Urinalysis: pH, specific gravity, protein, glucose, erythrocytes, leukocytes, and ketone bodies.

  Blood biochemistry: total bilirubin, ALT, AST, gamma-glutamyl transferase, alkaline phosphatase, albumin, total protein, lactate dehydrogenase, urea nitrogen, creatinine, uric acid, sodium, potassium, chloride, calcium, creatine kinase, creatine kinase isoenzyme, total cholesterol, high-density lipoprotein cholesterol, low-density lipoprotein cholesterol, and triglyceride.
- Blood pregnancy test (only applicable to females with childbearing potential).
- Blood sampling for population PK.
- 12-lead ECG.
- SMBG status: review SMBG records and provide instructions on SMBG for next stage.
- Review study medication usage and recycling. The drug administration record is reviewed and confirmed. The remaining medications and used drug packages since last visit are recycled. Instructions are given to subjects who did not take study medications per protocol.

- AEs and concomitant medications.
- A new subject diary is dispensed.
- Diabetes education, instructions on diet and exercise are provided.
- Study medications are dispensed.

3.5.6 Visit 6: Treatment Visit (Day 56 ± 4)

The following procedures are completed at Visit 6 (all laboratory tests are conducted in the central laboratory, and all samples are collected in fasting status):

- Vital signs (blood pressure, pulse rate, breath rate, body temperature, and body weight).
- Physical examination: general appearance, head and neck, chest (heart and lungs), abdomen, limbs etc..
- Laboratory tests (samples are suggested to be collected between 7:00 and 10:00 am)

  Assessment of diabetes status: HbA1c and FPG.

  Hematology: hemoglobin concentration, hematocrit, erythrocytes, leukocytes, neutrophils, basophils, eosinophils, monocytes, lymphocytes, and platelets.

  Urinalysis: pH, specific gravity, protein, glucose, erythrocytes, leukocytes, and ketone bodies.

  Blood biochemistry: total bilirubin, ALT, AST, gamma-glutamyl transferase, alkaline phosphatase, albumin, total protein, lactate dehydrogenase, urea nitrogen, creatinine, uric acid, sodium, potassium, chloride, calcium, creatine kinase, creatine kinase isoenzyme, total cholesterol, high-density lipoprotein cholesterol, low-density lipoprotein cholesterol, and triglyceride.

- Blood pregnancy test (only applicable to females with childbearing potential).
- 12-lead ECG.
- SMBG status: review SMBG record and provide instructions on SMBG for next stage.
- Review study medication usage and recycling. The drug administration record is reviewed and confirmed. The remaining medications and used drug packages since last visit are recycled. Instructions are given to subjects who did not take study medications per protocol.

- AEs and concomitant medications.
- A new subject diary is dispensed.
- Diabetes education, instructions on diet and exercise are provided.
- Study medications are dispensed.

3.5.7 Visit 7: Treatment Visit (Day 84 ± 4)

The following procedures are completed at Visit 7 (all laboratory tests are conducted in the central laboratory and samples are collected in fasting status):

- Vital signs (blood pressure, pulse rate, breath rate, body temperature, and body weight).
- Physical examination: general appearance, head and neck, chest (heart and lungs), abdomen, limbs etc..
- Laboratory tests (samples are suggested to be collected between 7:00 and 10:00 am)

  Assessment of diabetes status: HbA1c and FPG.

  Hematology: hemoglobin concentration, hematocrit, erythrocytes, leukocytes, neutrophils, basophils, eosinophils, monocytes, lymphocytes, and platelets.

  Urinalysis: pH, specific gravity, protein, glucose, erythrocytes, leukocytes, and ketone bodies.

  Blood biochemistry: total bilirubin, ALT, AST, gamma-glutamyl transferase, alkaline phosphatase, albumin, total protein, lactate dehydrogenase, urea nitrogen, creatinine, uric acid, sodium, potassium, chloride, calcium, creatine kinase, creatine kinase isoenzyme, total cholesterol, high-density lipoprotein cholesterol, low-density lipoprotein cholesterol, and triglyceride.
- Blood pregnancy test (only applicable to females with childbearing potential).
- MMTT: the test must be conducted after blood sampling for HbA1c and other laboratory tests are completed, and after taking study medications. Refer to Appendix 3 for MMTT procedures.
- Blood sampling for population PK.
- Biomarkers.
- 12-lead ECG.

- SMBG status: review SMBG records and provide instructions on SMBG for next stage.
- Review study medication usage and recycling. The drug administration record is reviewed and confirmed. The remaining medications and used drug packages since last visit are recycled. Instructions are given to subjects who did not take study medications per protocol.
- AEs and concomitant medications.
- A new subject diary is dispensed.
- Diabetes education, instructions on diet and exercise are provided.
- Study medications are dispensed.

3.5.8 Visit 8: Treatment Visit (Day 112 ± 4)

Procedures that are completed at Visit 8 please see Visit 6.

3.5.9 Visit 9: Treatment Visit (Day 140 ± 4)

Procedures that are completed at Visit 9 please see Visit 6.

3.5.10 Visit 10: Termination Visit for Double-blind Treatment (Day 168 ± 4)

The following procedures are completed at Visit 10 (all laboratory tests are conducted in the central laboratory and samples are collected in fasting status):

- Vital signs (blood pressure, pulse rate, breath rate, body temperature, and body weight).
- Physical examination: general appearance, head and neck, chest (heart and lungs), abdomen, limbs etc..
- Laboratory test at end of double-blind treatment (samples are suggested to be collected between 7:00 and 10:00 am)

  Assessment of diabetes status: HbA1c and FPG.

  Hematology: hemoglobin concentration, hematocrit, erythrocytes, leukocytes, neutrophils, basophils, eosinophils, monocytes, lymphocytes, and platelets.

  Urinalysis: pH, specific gravity, protein, glucose, erythrocytes, leukocytes, and ketone bodies.

Blood biochemistry: total bilirubin, ALT, AST, gamma-glutamyl transferase, alkaline phosphatase, albumin, total protein, lactate dehydrogenase, urea nitrogen, creatinine, uric acid, sodium, potassium, chloride, calcium, creatine kinase, creatine kinase isoenzyme, total cholesterol, high-density lipoprotein cholesterol, low-density lipoprotein cholesterol, and triglyceride.

- Blood pregnancy test (only applicable to females with childbearing potential).
- Blood sampling for population PK.
- Biomarkers.
- MMTT: the test must be conducted after blood sampling for HbA1c and other laboratory tests are completed, and after taking study medications. Refer to Appendix 3 for MMTT procedures.
- 12-lead ECG.
- Review SMBG records.
- Review study medication usage and recycling. The drug administration record is reviewed and confirmed. The remaining medications and used drug packages since last visit are recycled. Instructions are given to subjects who did not take study medications per protocol.
- AEs and concomitant medications.
- A new subject diary is dispensed.
- Diabetes education, instructions on diet and exercise are provided.
- Study medications are dispensed.

3.5.11 Visit 11: Open-Label Treatment Visit (Day 196 ± 4)

Procedures that are completed at Visit 11 please see Visit 6.

3.5.12 Visit 12: Open-Label Treatment Visit (Day 238 ± 6)

Procedures that are completed at Visit 12 please see Visit 6.

3.5.13 Visit 13: Open-Label Treatment Visit (Day 280 ± 6)

Procedures that are completed at Visit 13 please see Visit 6.

3.5.14 Visit 14: Open-Label Treatment Visit (Day 322 ± 6)

Procedures that are completed at Visit 14 please see Visit 6.

3.5.15 Visit 15: Termination Visit for End of Open-Label Treatment (Day 364 ± 6)

The following procedures are completed at Visit 15 (all laboratory tests are conducted in the central laboratory and samples are collected in fasting status):

- Vital signs (blood pressure, pulse rate, breath rate, body temperature and body weight).
- Physical examination: general appearance, head and neck, chest (heart and lungs), abdomen, limbs etc..
- Laboratory tests at end of study (samples are suggested to be collected between 7:00 and 10:00 am)

  Assessment of diabetes status: HbA1c and FPG.

  Hematology: hemoglobin concentration, hematocrit, erythrocytes, leukocytes, neutrophils, basophils, eosinophils, monocytes, lymphocytes, and platelets.

  Urinalysis: pH, specific gravity, protein, glucose, erythrocytes, leukocytes, and ketone bodies.

  Blood biochemistry: total bilirubin, ALT, AST, gamma-glutamyl transferase, alkaline phosphatase, albumin, total protein, lactate dehydrogenase, urea nitrogen, creatinine, uric acid, sodium, potassium, chloride, calcium, creatine kinase, creatine kinase isoenzyme, total cholesterol, high-density lipoprotein cholesterol, low-density lipoprotein cholesterol, and triglyceride.
- Blood pregnancy test (only applicable to females with childbearing potential).
- Blood sampling for population PK.
- Biomarkers.
- MMTT: the test must be conducted after blood sampling for HbA1c and other laboratory tests is completed, and after taking study medications. Refer to Appendix 3 for MMTT procedures.
- 12-lead ECG.
- Review SMBG records.
- Review study medication usage and recycling. The drug administration record is reviewed and confirmed. The remaining medications and used drug packages since last visit are recycled.

- AEs and concomitant medications.
- Diabetes education, instructions on diet and exercise are provided.
- Subjects are reminded that all study medications (including metformin) are stopped after Visit 15. No antidiabetic drugs are allowed between Visits 15 and 16. Under special medical conditions, other antidiabetic treatment must be given immediately after Visit 15. In this case, for safety considerations, routine treatment can be started at an early stage but the reason should be recorded on eCRF.

3.5.16 Visit 16: Follow-up Visit for Safety (Day 371 ± 2)

The following procedures are completed at Visit 16:

- Existing AEs and possible new AEs after end of treatment via phone-call.
- Concomitant medications via phone-call.
- Between Visits 15 and 16, subjects can contact the Investigator if there is any discomfort or hyperglycemia event. Depending on disease status, the Investigator decides if Visit 16 will be conducted in advance by phone. For safety considerations, routine antidiabetic treatment can be provided to subjects earlier, which needs to be recorded on eCRF. Routine medical care is given after Visit 16.

3.6 Study Materials Provided to Subjects

The following materials are provided to study sites by Hua Medicine:

- Blood glucose meter: a portable blood glucose meter is provided to each subject at Visit 2.
- Matched glucose test strips.
- Matched blood sampling needles.
- Subject diaries for recording daily drug usage, blood glucose value and any discomfort.
- Health care education materials.

4 Study Population

4.1 Inclusion Criteria

Subjects must meet all following inclusion criteria to enter the study (all laboratory parameters are tested in the central laboratory):

1. Male or female, aged 18~75 years old (inclusive) when they sign ICF.

2. Subjects diagnosed as T2DM in accordance to the WHO diagnostic criterion published in 1999 at screening period and treated with stable metformin ≥ 1500 mg/day constantly for at least 12 consecutive weeks in addition to dietary and exercises intervention.

3. 7.5% ≤ HbA1c ≤ 10.0% at screening.

4. 18.5 kg/m^2 < BMI < 35.0 kg/m^2 at screening.

5. Willing to follow the consistent diet and exercise interventions from the run-in period throughout the whole study. willing to take medication and meals on time per study protocol requirements. and to take timely SMBG and recording.

6. Willing to sign the written ICF and comply with the study protocol.

4.2 Exclusion Criteria

Exclusion Criteria

The subject cannot be enrolled with any of the following criteria (all laboratory parameters will be tested in the central laboratory).

Exceptions for Target Disease

1. Any antidiabetic therapy other than metformin within 12 weeks before screening.

2. Insulin therapy more than 30 days within 1 year before screening.

3. History of severe hypoglycemia (requiring external assistance for recovery) or recurrent hypoglycemia (such as experience of 3 or more times of hypoglycemia <=3.9 mmol/L or hypoglycemia symptoms within 1 month) without contributing factors within 3 months before screening.

4. Fasting C-peptide < 1.0 ng/ml (0.33 nmol/L) at screening visit.

5. Medical history of diabetic ketoacidosis, diabetes lactic acidosis or hyperosmotic nonketotic diabetic coma.

6. Subjects clinically diagnosed as type Ⅰ diabetes mellitus, or diabetes mellitus caused by pancreatic damage or other specific types of diabetes.

Medical History and Concomitant Disease

7. Have severe cardio-cerebrovascular medical history defined as:

a) Within 6 months prior to screening, subjects had medical history of myocardial infarction, coronary angioplasty or bypass graft(s) surgery, valvular heart disease or valve repair, and clinically significant unstable arrhythmia, unstable angina pectoris, transient ischemic attack, or cerebrovascular accident.

b) Congestive heart failure defined as New York Heart Association (NYHA) stage III and IV.

8. Medical history of unstable or rapidly progressive kidney disease.

9. Active liver diseases at screening.

10. Medical history of diagnosed mental disease.

11. Medical history of hemoglobinopathies (sickle cell anemia or thalassemia, and sideroblastic anemia).

12. The immunocompromised individuals such as subjects who had undergone organ transplants or diagnosed as human immunodeficiency virus (HIV) infection.

13. Subjects with any type of malignant tumor (regardless of cured or not).

14. Based on Investigator's judgment, any unstable or medical intervention-needed endocrine system diseases related to blood glucose (such as hyperthyroidism, acromegaly syndrome, Cushing's syndrome) or immune system disease that are improper for enrollment.

15. Medical history of drug abuse.

16. Subjects who received oral or injected corticosteroids treatment within 1 year at screening.

17. Alcohol intake> 2 units a day, or> 14 units a week.

    One unit of alcohol equivalent to 150 mL of wine, 350 mL of beer, 100 mL (<=17%) or 80 mL(>17% and <=24%) of low-alcohol liquor, or 50 mL of high-alcohol liquor (>24%).

Physical Examination and Laboratory Results

18. Significant abnormal liver function results at screening, defined as ALT > 2.5×ULN, or AST > 2.5×ULN or TBiL > 1.5×ULN (refer to Appendix 6).

19. Have serological evidence of infectious liver disease at screening defined as: positive IgM anti-HAV, or positive HBsAg and IgM anti-HBc (to exclude subjects with acute hepatitis virus B infection), or positive anti-HCV.

20. Estimated glomerular filtration rate (eGFR) < 60 mL/min/1.73m2 at screening.

21. Triglyceride (TG) > 5.7 mmol/L at screening.

22. Anemia (defined as hemoglobin < 12.0 g/dL (120 g/L) for men and hemoglobin < 11.0 g/dL (110 g/L) for women) of any causes at screening.

23. Based on Investigator's judgment, any abnormal laboratory parameters that may affect the safety evaluation.

24. Based on Investigator's judgment, any abnormal ECG that may affect the safety evaluation.

25. Subjects with uncontrolled hypertension (systolic blood pressure ⩾ 160 mmHg or diastolic blood pressure ⩾ 100 mmHg at screening) to antihypertensive treatment with stable doses for at least 4 weeks in run-in period.

Allergies and Adverse Drug Reactions

26. Based on Investigator's judgment, subjects may be allergic or intolerant to Investigational drug.

Prohibited Therapies and/or Medications

27. Subjects who refuse to take antidiabetic medication only required in this study.

28. Concomitant treatment with a potent or moderate CYP3A4/5 inhibitor or inducers (refer to Section 4.4.2).

Sex and Reproductive Status

29. Women who are pregnant or intend to be pregnant during study.

30. Women in breastfeeding.

31. Women who have childbearing potential and refuse to use a high-efficient and accredited contraceptive method through study startup to 1 month after the last medication with study drug.

Other Exclusion Criteria

32. Based on Investigator's judgment, any accompanied conditions or diseases may interfere with subjects to complete the whole study or to participate all processes of the study.

33. Based on Investigator's judgment, subjects need other therapy which may potentially affect the interpretation of the efficacy and safety data of the study.

34. Based on Investigator's judgment, subjects who are unable to comply the requirements specified in the protocol, for instance: fail to keep the dietary and exercise control during the study, fail to take required medicine and meal timely per protocol requirements. and fail to conduct SMBG and make record timely etc.

35. Subjects who participated in any other clinical trials or took any other study drug within 30 days before screening.

4.3 Randomization Criteria (Laboratory tests and will be conducted on Day -5 [± 2 days] prior to the randomization)

Before randomization of Visit 4 (randomization visit), subjects are re-evaluated for eligibility by following parameters. Those who meet randomization criteria are eligible to enter the randomization period.

Inclusion Criteria

1. HbA1c ⩾ 7.5% and ⩽ 10.0% at pre-randomization visit.
2. Fasting blood glucose> 7.0 mmol/L and < 13.3 mmol/L at pre-randomization visit.

Exclusion Criteria

3. History of severe hypoglycemia (requiring external assistance for recovery) or recurrent hypoglycemia (such as experience of 3 or more times of hypoglycemia <=3.9 mmol/L or hypoglycemia symptoms within 1 month) without contributing factors within 3 months in run-in period.
4. Body weight changes (weight gain or loss) ≥ 10% at randomization compared with that at screening.
5. Poor drug compliance (< 80% or > 120%) during run-in period.
6. Significant abnormal liver function in run-in period, defined as ALT > 2.5×ULN, or AST > 2.5×ULN or TBiL > 1.5×ULN at pre-randomization visit (refer to Appendix 6).
7. Estimated glomerular filtration rate< 60mL/min/1.73m^2 at pre-randomization visit.
8. Triglyceride> 5.7mmol/L at pre-randomization visit.
9. Anemia (defined as hemoglobin < 12.0 g/dL (120 g/L) for men and hemoglobin < 11.0 g/dL (110 g/L) for women) of any causes at pre-randomization visit.
10. Subjects with uncontrolled hypertension (systolic pressure ⩾ 160mmHg or diastolic pressure ⩾ 100 mmHg at randomization visit and with stable dose of

antihypertensive treatment in run-in period.

11. Based on Investigator's judgment, any abnormal laboratory parameters that may affect the safety evaluation.

12. Based on Investigator's judgment, any abnormal ECG that needs medical intervention or may affect the safety evaluation.

13. Additionally, the Investigator should re-confirm the medical history and concomitant disease from screening to randomization visit (refer to exclusion criteria at screening).

4.4 Concomitant Medication and Post-study Treatment

4.4.1 General Instructions on Study Treatment

Investigator can use other medications if they are critical for the safety and health of subject. All medication taken must be recorded in eCRF.

From the ICF signed date to one week after treatment completion (Visit 16), any concomitant medications (study medications and other medications) or the changes on dosages of previous medications must be documented in the corresponding eCRF section. Reasons for giving the medications, administration dates (start and stop dates), and dosage information (dose, route, frequency) should also be documented.

4.4.2 Prohibited and Restricted Medications

Other antidiabetic drugs, except the study medications, are not allowed to be used throughout the study from the run-in period. If hyperglycemia happens and meets the withdraw criteria, the subject will be treated as withdrawal. This does not apply to those medication used for AE treatment or concomitant medications which have been used stably before the study.

Unless emergency, subjects are not allowed to take any new medications which have not been used at screening, or adjust current dosage of any study medication or concomitant medications without permission from the Investigator. In case of any emergency when there is any clinical indication needing additional medications or dosage change of concurrent medications, the subjects must inform the Investigator within reasonable timeframe before concomitant medications are started. The information, including drug name, dosage, route of administration, treatment purpose and administration dates are confirmed and recorded by the Investigator.

The following medications are strictly prohibited unless for special medical conditions

- Any antidiabetic drug (including traditional Chinese medicine) other than the study medications (starting from the run-in period)
- Hypoglycemic healthcare product

- Oral or injectable glucocorticoid
- Any known potent or moderate liver enzyme CYP450 3A4/5 inducers/inhibitors, including but not limited to:

  Potent liver enzyme inducers: barbitals (especially phenobarbital), carbamazepine, diphenylhydantoin sodium, rifampin, dexamethasone, etc.

  Potent liver enzyme inhibitors: ketoconazole, itraconazole, voriconazole, posaconazole, clarithromycin, indinavir, ritonavir, saquinavir, telithromycin, Boceprevir, nelfinavir, Telaprevir, conivaptan, nefazodone.
- Any unauthorized drugs (i.e., investigational drugs not marketed yet).

4.4.3 Contraception

From screening to 1 month after the last dose of study medications, female subjects with childbearing potential must use high-efficiency, medically approved contraception methods (e.g. barrier type intrauterine device combined with spermicides, IUD implanted at least 3 months before study, oral contraceptives and preventive measures). The same method is recommended to be used throughout the study.

See Section 7.12 in the protocol for procedures for handling pregnancy.

4.4.4 Diet Control and Exercise Intervention

Instructions are given to subjects on nutrition and physical exercise, how to identify the signs and symptoms of hypoglycemia and hyperglycemia, and how to use blood glucose meter (refer to Appendix 4). The Investigator should ask subjects to keep fasting for at least 8 hours before each visit to study sites (from Visit 3 to Visit 15). Each site is suggested to give subjects instructions on nutrition and diet and ask them to follow the standard diet guideline for diabetes patients (refer to Appendix 8).

4.4.5 Post-Study Treatment

After 52-week treatment is completed, all the investigational drugs (HMS5552 tablets and metformin) are stopped and subjects proceed to 1-week safety observation period. In the safety observation period, in principle, no antidiabetic drugs can be used until the Visit 16 telephone follow-up is completed. Routine treatment can be initiated after Visit 16.

Subjects can contact the Investigator if any hyperglycemia or discomfort occurs between Visits 15 and 16. Depending on disease status, the Investigator decides whether to conduct the telephone follow-up in advance. Under special medical conditions, subjects can take routine antidiabetic treatment in advance for safety consideration. The reasons need to be recorded on eCRF.

4.5 Discontinuation of Study Treatment, Withdrawal, or Termination of Study

During the study, the Investigator should make every effort to encourage subjects to complete the entire study.

Subjects have the right to withdraw from the study treatment for any reason and at any time. The Investigator has the right to require subjects to discontinue the study treatment or withdraw from the study.

In addition, the reasons for discontinuation of study treatment or withdrawal from the study include but not limited to:

- Withdrawal of informed consent form by subjects
- AEs
- Hyperglycemia
- Hypoglycemia
- Pregnancy
- Subjects do not comply with study treatment or scheduled visits (e.g., drug administration or visits not following Investigator's instructions)
- Major protocol deviations and high risks to subjects as considered by the Investigator
- Lost to follow-up
- Others.

Subject withdrawal occurring before randomization for any reason (e.g., not meeting the inclusion criteria) is considered as "screening failure".

The Sponsor can terminate the study or certain study site based on the overall consideration for the study.

Specific early withdrawal criteria due to hypoglycemia, hyperglycemia, and abnormal liver function have been defined to ensure subject safety. See Sections 4.5.1 to 4.5.3.

Subjects who expect to re-enter the study after discontinuation of study treatment is allowed if the interval is ≤ 2 weeks since discontinuation, which will be decided by the Investigator after consultation with the Sponsor.

4.5.1 Withdrawal Due to Hypoglycemia

4.5.1.1 Definition of Hypoglycemia

Hypoglycemic events are collected and classified as follows according to the China guidelines for the prevention and treatment of T2DM (2013)[3]. The Clinical Trials Guidelines of Diabetes Treatment Drug and Biological Products (2012)[16], and a joint statement made by the American Diabetes Association (ADA) and the European Association for the Study of Diabetes (EASD) recently published in November 2016 for identifying and reporting hypoglycemic events [17]:

1. Fingertip or venous plasma glucose value **≥ 3.0 mmol/L, ≤ 3.9 mmol/L**, with or without symptoms of hypoglycemia.
2. Fingertip or venous plasma glucose value **< 3.0 mmol/L**, with or without symptoms of hypoglycemia.
3. **Severe hypoglycemia**: assistance from others is needed, usually with disturbance of consciousness, and neurological symptoms improve significantly or disappear after correction of hypoglycemia.

Hypoglycemia symptoms may include shakiness, hot flashes, vertigo, weakness, cold limbs, anxiety, dizziness, hunger, palpitations, and blurred vision, etc.

1. In addition, based on The Clinical Trials Guidelines of Diabetes Treatment Drug and Biological Products (2012), the following possible hypoglycemic events are also collected but will not be used for hypoglycemia assessment:

Possible symptomatic hypoglycemia: presenting with symptoms of hypoglycemia, but without plasma glucose test results.

Relative hypoglycemia: presenting with hypoglycemia symptoms, but with plasma glucose > 3.9 mmol/L (fingertip or venous).

4.5.1.2 Rules for Withdrawal due to Hypoglycemia

2. If severe hypoglycemia occurs (help from others is needed, usually with disturbance of consciousness, and neurological symptoms improve significantly or disappear after correction of hypoglycemia) and no other reason (e.g., physical exercise increase or meals skipping) except for the study medications can be found, the study medications are discontinued and the subjects withdraw from the study.
3. If fingertip or venous plasma glucose value is < 3.0 mmol/L and the result occurs twice at two consecutive visits without other reasons (e.g., physical exercise increase or meals skipping) except for the study medications, the study medications are discontinued and the subjects withdraw from the study.

Subjects should be informed to contact study sites in following conditions:

Any hypoglycemia that needs help (i.e., severe hypoglycemia), or any occurrence of hypoglycemia with fingertip or venous plasma glucose value ≤ 3.9 mmol/L.

Notes: As noted before, subjects record all symptoms related to hypoglycemia and fingertip plasma glucose values in subject diaries. The Investigator should review each hypoglycemia event and carefully inquire about the associated symptoms, treatment, and duration etc. Hypoglycemia documented in subject diaries needs to be confirmed by reviewing glucose meter records. All hypoglycemic events confirmed by the Investigator must be recorded on the hypoglycemia review form in eCRF.

4.5.2 Withdrawal due to Hyperglycemia

Between Visits 2 and 15, subjects should carry out SMBG according to SMBG guidance (refer to Appendix 4 for procedures of SMBG). Subjects should contact the Investigator timely if plasma glucose exceed the value specified in SMBG guidance. Depending on the fingertip plasma glucose values, diet and exercise condition, the Investigator determines whether the subjects should return to study sites in advance for laboratory tests.

If a subject has a FPG value reaching the following criteria twice (at least once tested in the central laboratory) without any apparent causes at two consecutive visits, the Investigator will evaluate whether the subject will withdraw from the study with the reason recorded as hyperglycemia based on subject's actual situation.

1. Day 1–before Week 12: venous FPG (central laboratory) ≥ 13.3 mmol/L
2. Week 12–before Week 24: venous FPG (central laboratory) ≥ 11.1 mmol/L
3. Week 24–Week 52: venous FPG (central laboratory) ≥ 11.1 mmol/L.

If a subject is confirmed to withdraw from the study due to hyperglycemia, the subject must complete all the laboratory tests same with those required at Visit 15 (open-label treatment) before withdrawal (the information on the "Unplanned Study Visits" page in eCRF needs to be completed if the visit doesn't overlap with the planned study visits).

The Investigator must ensure no other causes can be identified to explain poor glycemic control except for lack of efficacy (e.g., acute inflammations/infections, concomitant treatments, etc.). If the disease aggravation is unexpected progression, it should be reported as an AE.

4.5.3 Withdrawal due to Abnormal Liver Function

Depending on the subject's condition, study treatment can be discontinued if any of the following criteria is met (no need to wait for the retest results):

- ALT or AST > 8×ULN

- ALT or AST > 5×ULN and lasts for ≥ 14 days
- ALT or AST > 3×ULN and TBiL > 2×ULN
- ALT or AST > 3×ULN with liver damage symptoms (e.g., fatigue, nausea, vomiting, right upper abdominal pain or tenderness, fever, rash) or eosinophilia increase.

Active intervention or treatment needs to be given to subjects. The causes for abnormal liver function need to be identified. Consultation with hepatologists will be conducted if needed. Subjects with abnormal liver function are closely followed up until the liver function restore or the results return to baseline level. The termination visit should be scheduled as soon as possible within 3 days after discontinuation of study treatment. AEs relevant to abnormal liver function need to be recorded in time. SAEs which meet the criteria should be reported immediately.

4.6 Procedures for Subject Withdrawal

All subjects who withdraw from the study are kept under medical monitoring. The reasons for withdrawal need to be confirmed by contacting the subject or the relatives by phone or email. If withdrawal occurs during the treatment period, the efficacy and safety observations and assessments (same as Visit 15) at the termination visit need to be completed as much as possible within 7 days (including the seventh day) after the last dose of study medications. Safety assessment via phone within 1 week after the last dose of study medications also needs to be completed. If the study treatment has been discontinued by subjects for more than 7 days before the withdrawal, the assessments (same as Visit 15) at termination visit is strongly recommended to be completed. If study treatment has been discontinued at the termination visit, the MMTT can be cancelled.

For subjects who withdraw early from the study, a comprehensive evaluation is conducted to find the reasons for early withdrawal. Withdrawal reasons should be recorded in detail. For example, the Investigator should ask about the reasons for inform consent withdrawal, rather than just to record the withdrawal.

If the early withdrawal from study is due to AEs or abnormal laboratory results, the details should be recorded on eCRF. AEs are followed up (see Section 7.2). Subject diaries, all remaining medications and used drug packages should be returned.

4.7 Lost to Follow-up

All reasonable efforts must be made to contact subjects to confirm and record their current situation. Actions include contacting the authorized personnel (e.g. spouses, children or other relatives). Lost to follow-up is defined as, as shown in records, failure to reach subjects via phone calls, faxes or emails at least 3 times within 1 month (each contact is on different calendar days). All attempts should be recorded in subjects' medical records. If death is confirmed, the study sites acquire the date and cause for the death in a way permitted locally. If a subject is still lost to follow up after all efforts, the last known survival date should be decided by the

Investigator and recorded in subjects' medical records.

5 Study Drugs

5.1 Study Medications

5.1.1 Drug Name, Strength, Formulation

Investigational Drug

HMS5552 tablets. formulation: light yellow, double convex, round, film-coated tablet. specification: 75mg/tablet. Batch No.: BXE17001 (Double-blind treatment period), BXE17001 or BXE18001 (open-label treatment period). Administration route: oral. Drug supplier: Hua Medicine (Shanghai) Ltd.

Control Drug

Placebo tablets, no active drug ingredient, but have the same appearance, odor, and color etc. with HMS5552 tablets. Batch No.: BXK17001. Administration route: oral. Drug supplier: Hua Medicine (Shanghai) Ltd.

Basic Drug: Metformin (Glucophage)

Formulation: tablet. specification: 500mg/tablet. Administration route: oral. Batch No: refer to original package. Manufacturer: Merck Serono Co. Ltd.

5.1.2 Packaging and Labelling

All study medications are packed and labeled in compliance with GMP requirements.

The packing method used for 4-week visit period is as follows:

- The packing details for basic drug (Glucophage, metformin hydrochloride tablets): the original package from the provider is kept, i.e., each small box contains 2 packs with 10 tablets for each (total 20 tablets). For the second packaging, the box in medium size containing 5 small boxes is prepared for 4-week (28 days) dosage (totally 5 small boxes. each small box contains 2 packs with 10 tablets for each. Thus, a total of 20 tablets in small box and 100 tablets in medium box). Of the 100 tablets, 84 tablets are for 4-week dosage (28 days, 3 tablets/day) and 16 tablets are for backup.

- The single-blind placebo run-in period lasts for 4 weeks, in which the study medications are administered BID. For each subject, all the study medications (control drug) for run-in period are packed together as one package (dosage for 4 weeks). Each package consists of 8 packs and each pack contains 8 tablets, 64 tablets in total. Of the 64 tablets, 56 tablets are used within 4 weeks (28 days), and 8 tablets are for backup.

- After randomization, study medications (investigational drug and control drug) are packed separately with a unique packing number (study drug box serial number). Each package contains the dosage for 4-week treatment. Each package consists of 8 packs and each pack contains 8 tablets, 64 tablets in total. Of the 64 tablets, 56 tablets are used within 4 weeks (28 days), and 8 tablets are for backup.

Package for 6-week visit period is as follows:

- The packing details for basic drug (Glucophage, metformin hydrochloride tablets): the original package from the provider is kept, i.e., each small box contains 2 packs with 10 tablets for each (total 20 tablets). For the second packaging, the box in medium size containing 8 small boxes is prepared for 6-week (42 days) dosage (totally 8 small boxes. each small box contains 2 packs with 10 tablets for each. Thus, totally 20 tablets in small box and 160 tablets in medium box). Of the 160 tablets, 126 tablets are for 6-week dosage (42 days, 3 tablets/day) and 34 tablets are for backup.

- Each package contains the study medications for 6-week treatment. Each package consists of 12 packs and each pack contains 8 tablets, 96 tablets in total. Of the 64 tablets, 84 tablets are used within 6 weeks (42 days), and 12 tablets are for backup.

Study medications are sent to each study site separately and the Investigator at each site is responsible for drug storage.

Drug label should include at least information below:

- Sponsor name
- Study medications/formulation, administration route, and the quantity of dosage unit
- Drug storage conditions
- Study number
- Drug box serial number
- Use instructions
- Investigator’s name (add to the label when dispensing study medications, if applicable)
- Expiry date (term of validity)
- “For clinical study use only”
- “Keep out of reach of children”.

5.1.3 Study Medication Regimen and Packaging

Subjects from each group orally take the following medications all at once, BID. The medications are suggested to take at a draught before breakfast and supper (If the Investigator considers metformin should be administered 3 times a day, another dose is suggested to be taken in lunch time). The time for drug administration is suggested to be consistent as much as possible throughout the study.

Single-blind run-in period

Placebo tablets

First dose Placebo tablet×1, taken at a draught before breakfast

Second dose Placebo tablet×1, taken at a draught before supper

Metformin is taken following doctor's advice

Double-blind treatment period

Group A HMS5552 tablets, 75mg BID group

First dose HMS5552 tablet 75mg×1, taken at a draught before breakfast

Second dose HMS5552 tablet 75mg×1, taken at a draught before supper

Metformin is taken following doctor's advice

Group B Placebo tablets, placebo BID control group

First dose Placebo tablet×1, taken at a draught before breakfast

Second dose Placebo tablet×1, taken at a draught before supper.

Metformin is taken following doctor's advice

Open-label treatment period

Group A HMS5552 tablets, 75mg BID group

First dose HMS5552 tablet 75mg×1, taken at a draught before breakfast

Second dose HMS5552 tablet 75mg×1, taken at a draught before supper

Metformin is taken following doctor's advice

Group B HMS5552 tablets, 75mg BID group

First dose HMS5552 tablet 75mg×1, taken at a draught before breakfast

Second dose HMS5552 tablet 75mg×1, taken at a draught before supper.

Metformin is taken following doctor's advice

All drug storage information (empty packs, partially used or unused drugs) should be recorded and monitored regularly by clinical research associate (CRA). All unused medications must be returned to the Investigator at each visit. The Investigator returns all received medications to the Sponsor after study closure.

5.2 Storage Condition

HMS5552 is recommended to be stored under room temperature (10–30°C), avoiding high humidity and strong light.

The basic drug metformin (Glucophage) is recommended to be stored in sealed condition. Details are provided in drug instructions.

The Investigator should pay adequate attention to ensuring the study medications to be securely stored in locked cabinets with limited access or in other fully secured locations.

5.3 Drug Dispensing and Counting

The Investigator and/or study sites are responsible for establishing a system to manage the study (including study medications) and ensure that:

- The Investigator or designated staff can correctly receive the study medications from Hua Medicine (Shanghai) Ltd.
- Receiving and dispensing of study medications is recorded in the drug inventory. The Investigator is required to ensure the correct records to demonstrate the receiving and disposition of the study medications (Hua Medication Ltd. or designated organizations provide copies of listings for shipping and delivery of study medications). The following information is recorded: the person to whom the drug is dispensed, the quantity, the date of drug dispensing, and any unused drugs returned to the Investigator. The information is provided as supplementary information to the records on eCRF. After study completion, drug inventory and dispensing log should be checked and confirmed, and any discrepancy should be clarified. The dispensing and return of study medications requires to be confirmed by signature, ideally by the Investigator or his designees.
- Study medications must be handled and stored safely and appropriately.
- Storage of study medications (empty packs, partially used or unused medications) should be recorded and monitored by the CRAs.
- The study medications are only dispensed to subjects taking part in the study.
- Only the Investigator, Sub-Investigator or their designees are authorized to dispense the study medications. The study medications can only be used in this study per study

protocol. Without the permission from Hua Medicine (Shanghai) Ltd, the study medications should not be used for other purposes.

5.4 Drug Return and Destruction

At each visit, all unused study medications and used packages for that visit must be returned to the Investigator. The study staffs count all returned medications and empty packs. The drug compliance is determined by comparing the number of returned tablets with the number of dispensed tablets. The results must be documented on corresponding eCRF pages.

All returned medications and empty packs are kept by the Investigator, and returned to designated places or other companies designated by Hua Medicine for destruction. Hua Medicine (Shanghai) Ltd. or designated organizations provide a receipt (or relevant form) to document drug return.

5.5 Drug Compliance

For subjects who are followed up in the study, “planned dosage” stands for the planned total dose that should be taken in certain periods (e.g. run-in or treatment periods). If early withdrawal from the study occurs, the planned dosage stands for the total dose that should be taken from beginning of that period to the last day of administration. Actual dosage means: dispensed quantity - returned quantity - missed quantity (must be verified and consistent with the record in subject diaries. If not, details of actual drug usage should be checked and recorded).

The following formula is applied to calculate the percentage of drug compliance:

$$\text{Percentage of Drug Compliance} = \frac{Actual\ \ Dosage}{Planned\ \ Dosage} \times 100\%$$

Drug compliance lower than 80% or greater than 120% suggests a poor drug compliance.

5.6 Extent of Exposure

Extent of exposure is summarized based on the drug compliance results.

6 Study Endpoints

The methods for assessment and documentation of study endpoints and time for assessment are as follows. The efficacy parameters include HbA1c, PPG and FPG.

Follow up visits are scheduled at Weeks 4, 8, 12, 16, 20, 24, 28, 34, 40, 46, 52 after study treatment starts. Efficacy parameters, e.g. HbA1c and FPG, are tested at each visit (refer to the Flow Chart of Clinical Study for details on assessments). The visit window for Weeks 4, 8, 12, 16, 20, 24 and 28 is defined as ± 4 days of visiting day. whereas the visit window for Weeks 34, 40, 46, and 52 is defined as ± 6 days of visiting day. and visit window of ± 2 days for Week 53

phone visit is suggested. The schedule of visit window is based on normal working time. In case of the National Day, Spring Festival and other holidays, the Investigator can arrange for administration of the study medications accordingly and reschedule the next visit to a time closest to the visit scheduled per study protocol. Causes for early/late visit are recorded on eCRF and this is not considered as a protocol deviation.

Study visits are recommended to be arranged between 7:00 and 10:00 a.m. High calorie and high fat diet is not permitted the day before the study visit. Subjects should stay in fasting status for at least 8 hours from 22: 00 on the previous day until sampling for the visit is completed, without intake of food/drink (water is permitted). If a subject is not prohibited from high calorie and high fat diets or not fasted, the sampling for the subject is suggested to be done in the morning next to the scheduled visit. The Investigator should emphasis the importance of prohibition on high calorie and high fat diets and sampling in fasting status.

The Investigator should inform the subjects not taking study medications in the morning of study visit. The study medications should be taken after sampling in fasting status. Then the MMTT is conducted. The Investigator should also remind subjects of returning the remaining medications and used drug packages on the day of study visit.

Procedures of MMTT:

The MMTT is a standardized method of measuring PPG level. In this study, MMTT is conducted to assess the impact of HMS5552 on PPG, insulin, and C-peptide. (Note: in this study, all meals for MMTT are referred to instant noodles standard meal produced by the COFCO provided by the Sponsor. The usage method and cautions are described in Appendix 3).

If a subject withdraws from the study early, and already has discontinued study medications at the termination visit, the MMTT is not needed (in other cases, the procedures are consistent with Visit 15).

7 Safety Assessments

7.1 Adverse Event (AE)

AE refers to any untoward medical occurrence, including exacerbation of a pre-existing condition, in a patient or a subject in a clinical study who has been administered with an investigational drug, no matter its causal relationship with the investigational drug. An AE can therefore be any unfavorable and unintended disease (e.g., acute appendicitis), symptom (e.g., nausea, chest pain), sign (e.g., tachycardia, hepatomegaly), or abnormal laboratory finding (e.g., laboratory tests, ECG). In a clinical study, an AE can be any undesirable medical condition occurring at any time after the ICF has been signed, even if no investigational drug has been given yet.

AEs include serious and non-serious AEs.

7.1.1 Serious Adverse Event (SAE)

SAE is defined as an AE occurring in any period of the study (i.e. run-in, treatment, follow-up) that meets one or more of the following criteria:

1. Resulting in death
2. Immediately life-threatening

The term "life-threatening" refers to an event in which the subject is at immediate risk of direct death at the time of the event. It does not include the situation that might lead to death if it were more severe. For example, drug-induced hepatitis resolved without hepatic failure cannot be considered as life-threatening though it might be fatal.

3. Requiring hospitalization or prolonged hospitalization

Hospitalization or prolonged hospitalization due to elective surgery, routine clinical procedures, social reasons or self-convenience do not need to be recorded as an AE. If the events meet AE criteria e.g., aggravation from previous condition, they should be reported as “serious” or “non-serious” AEs based on routine criteria.

4. Resulting in persistent or significant disability/incapacity
5. Congenital anomaly/birth defect
6. Important medical event: to preclude one of the outcomes listed as above, medical and scientific judgment should be made to assess whether other situations should be considered as serious, such as important medical events that may not be immediately life-threatening or result in death or hospitalization but may jeopardize the subject or may require medical intervention. Examples of such events include allergic bronchospasm requiring intensive treatment in an emergency room or at home. blood dyscrasias or convulsions that do not result in hospitalization. drug dependency or drug abuse, etc.

7.1.2 Persistent or Recurrent AEs

Persistent AEs are events that continuously exist during the assessment period without resolution. These events should be recorded only once on eCRF. The initial severity of this event should be recorded, and the highest severity should be updated once the event is aggravated. If the event becomes an SAE, corresponding update should be made on eCRF to reflect this situation.

Recurrent AEs are events that are recovered during the assessment period, but reoccur afterwards. Each recurrence must be recorded separately on the AE page of eCRF.

7.1.3 AEs Related to Examinations and Laboratory Tests

Abnormal results from examinations and laboratory tests (such as hematology, blood biochemistry, urinalysis, ECG, physical examination, vital signs, etc.) at screening are regarded as pre-existing condition before the ICF is signed and do not need to be recorded as an AE. Clinically significant abnormality at screening can be recorded as relevant medical history according to the Investigator's judgement. The Investigator is responsible for assessing all abnormal results of examinations and laboratory tests for clinical significance. Any abnormality meeting the following criteria is considered as clinically significant and should be reported as an AE:

- The abnormality that suggests a disease and/or organ toxicity that is new or has worsened from baseline.
- The abnormality that needs the investigational product dosage and usage to be adjusted, e.g., dose change, drug discontinuation, etc.
- The abnormality that requires additional active intervention, e.g. increase or modification of the concomitant medications, close observation, more frequent follow-up assessments, or further diagnostic examination, etc.

If the laboratory abnormality of clinical significance is a manifestation of disease (e.g. alkaline phosphatase and total bilirubin increase with cholecystitis), then only the diagnosis (i.e., cholecystitis) will be recorded as an AE. If laboratory abnormality of clinical significance is not a manifestation of disease, then the abnormality will be recorded as an AE. Appropriate description will be used to record if the test results are lower or higher than normal range (e.g. "serum potassium increase" rather than "abnormal serum potassium"). If the result meets diagnostic criteria, the clinical diagnosis will be recorded as an AE. For instance, when serum potassium increases to 7.0 mmol/L, it can be recorded as "hyperpotassemia".

7.2 Recording of AEs

7.2.1 Terms of AEs

All AEs reported by subjects or found by the Investigator are recorded and described on eCRF in accurate and standard medical terminology. The diagnosis is recommended to be used as the term for the AE. If no diagnosis, the symptoms, signs, or laboratory abnormality can be reported as the term of the AE. When a diagnosis is determined afterwards, the record can be updated accordingly.

7.2.2 Recording of AE Time and Collection Period for AE

The Investigator should try to collect the start and end dates of each AE. Correct records of AE start time are helpful in assessing the temporal relationship between AE and drug administration.

Usually, the AE start date should be the date of symptom onset. If the AE is aggravated from the baseline medical conditions, the AE start date is the date of condition aggravated. If the AE is newly happened in the study and progressed to SAE, the SAE start date should still be the AE start date.

In this study, AEs are collected from the first dose of study medication in run-in period to the last visit.

The SAEs are collected from the first informed consent form is signed to the last visit.

7.2.3 AE Variables

The following variables are collected for each AE:

- Term (verbatim)
- Start and end dates/time
- Severity
- Serious adverse event or not
- The SAE criteria
- Action taken for study medication
- Outcome
- Assessment made by the Investigator on the causal relationship to study medication
- Hospitalization date (if applicable)
- Discharge date (if applicable)
- Cause of death (if applicable)
- Date of death (if applicable)
- Autopsy (if applicable).

7.2.4 AE Grading

AEs are graded by severity as mild, moderate and severe:

- **Mild**: appearance of signs or symptoms, but can be easily tolerated.
- **Moderate**: obvious discomfort interfering normal daily activities.

- **Severe**: incapacitating, and can't perform normal daily activities.

7.2.5 AE Outcome

The outcomes are described as follows:

- Recovered/resolved: the subject returns to baseline state.
- Recovering/resolving: the event hasn't been resolved completely, but the subject is improving.
- Not recovered/not resolved: the event is ongoing, e.g., irreversible congenital anomaly.
- Recovered/resolved with sequelae: only if the subject suffers from lifetime sequelae, e.g., blindness caused by diabetes, and the hemiplegia after a stroke.
- Fatal: the death date is the date for event ending.
- Unknown: the Investigator can't obtain the outcomes of the AE, e.g. the subject is lost to follow-up.

If an AE is assessed as "recovered/resolved", "recovered/resolved with sequelae", or "fatal", the AE ending date must be recorded.

The Investigator must follow up all AEs. When the study is completed, any AEs that are unresolved or don't have outcomes should be followed up by the Investigator until the events are resolved or considered stable, which don't have to be recorded further on eCRF (e.g., after database lock). The follow-up information on SAEs that occurs after database lock needs to be reported to Clinical Drug Safety department of the Sponsor. The contact information is:

Email: drugsafety@huamedicine.com. Fax: 021-58863272. Mobile: +86 156 1839 6573.

7.2.6 Causality Assessment

The Investigator should assess the causal relationship between the AE and the study medications through medical and scientific judgement. The following criteria can be referred to:

1. Related. The AE occurrence follows a reasonable temporal relationship with administration of the study medication. The AE is consistent with the known profile of the study medication. The event is improved when the dose of study medication is decreased or stopped. The event reoccurs when the study treatment is restarted. The AE can't be explained by subjects' medical condition or alternative treatment.
2. Possibly related. The AE occurrence follows a reasonable temporal relationship with administration of the study medication. The AE is consistent with the known profile of

the study medication. The AE alleviates or becomes not obvious after reducing the dosage or stopping the study medication. The subjects' medical condition or alternative treatment may lead to the event.

3. Unlikely related. The temporal relationship between the AE occurrence and administration of the study medication is not clear. The AE may not be consistent with the known profile of the study medication. The subjects' medical condition or alternative treatments may lead to the event.

4. Not related. There is no reasonable temporal relationship between the AE occurrence and administration of the study medication. The AE is not consistent with the known profile of the study medication. The subjects' medical condition or alternative treatments may lead to the event. The AE improves or disappears when the disease condition improves or alternative treatment is stopped.

7.3 Serious Adverse Event Reporting

Once a SAE occurs in the study, the Investigator must immediately take appropriate measures for treatment and report the SAE to the relevant drug administration departments, health administration departments, ethic committees and the Sponsor within 24 hours after awareness. The Investigator needs to ensure the SAE information be recorded on eCRF timely.

When completing the SAE report form (CFDA version), the Investigator must also collect the requested SAE information requested by the Sponsor. Refer to the Investigator Site File for the details of SAE reporting.

7.4 Hypoglycemia

The Investigator must correctly instruct subjects to conduct the SMBG (refer to Appendix 4 procedures for SMBG) to identify and manage hypoglycemia. Subjects should measure their fasting and postprandial fingertip blood glucose at least twice a week, and record in subject diaries. Any symptoms of hypoglycemia should be recorded in subject diaries. The subjects should be instructed to measure blood glucose when hypoglycemia symptoms appear, and also to carry carbohydrates that can be easily taken for hypoglycemia management.

Hypoglycemia event should be recorded on the hypoglycemia evaluation page of eCRF. Detailed information listed below must be recorded for each hypoglycemia occurrence: start date and time, end date and time, blood glucose values, symptom description, action taken, severe hypoglycemia or not, resolved or not, precipitating factors, causal relationship with the study medication, etc.

Refer to Section 4.5.1.1 of the protocol for the hypoglycemia assessment and classification criteria.

7.5 Hy's Law

The Hy's Law is used in this study to monitor any possible drug-induced liver injury. The Hy's Law[18] usually means that AST or ALT> 3×ULN and TBiL> 2×ULN, non-biliary increase (usually alkaline phosphatase < 2×ULN) without any other diseases for explanation of the increase. If ALT or AST is > 3×ULN, and TBiL is > 2×ULN without any other previous diseases for explanation of the increase, no matter whether alkaline phosphatase (ALP) increases or not, the Investigator must inform the subject of the central laboratory reports within 48 hours after awareness and ask the subject to return to the study site for central laboratory re-evaluation (if necessary, re-examinations at local laboratories can be taken). The Investigator conducts re-evaluation and closely monitors or follows-up with the subjects, or stops the study medications according to the Section 4.5.3 Subject withdrawal due to abnormal liver function and Appendix 6 Measures to be Taken for Abnormal Liver Functions in the protocol. The Investigator evaluates the etiology and conducts every essential examination to rule out drug-induced liver injury. If the Investigator confirms the event meets Hy's Law, the event needs to be reported as an SAE.

7.6 Post-study AE Reporting

At any time after the study ends, if the Investigator is informed of any AE which is considered possibly related to the study medication, the Investigator needs to promptly report the event to the Clinical Drug Safety department of the Sponsor. The follow-up information of the SAEs after database lock also needs to be reported to the Clinical Drug Safety department of the Sponsor. The contact information is: Email: drugsafety@huamedicine.com. Fax: 021-58863272. Mobile: 156 1839 6573.

7.7 Lack of Efficacy or Aggravation of T2DM

The events that are apparently consistent with expected progression of T2DM (e.g., hyperglycemia) do not need to be recorded as AEs. These data is used for efficacy assessment (parameters for glycemic control).

The protocol defines the withdrawal criteria relevant to poor glycemic control/hyperglycemia (see Section 4.5.2 withdrawal criteria due to hyperglycemia and Appendix 4 SMBG Procedures). The Investigator must warrant no other causes for poor glycemic control except for lack of efficacy (e.g. concurrent acute inflammations/infections, and concomitant treatments, etc.). If the disease aggravation is unexpected, it should be reported as an AE. The Investigator is suggested to give the subjects relevant examinations to find out the reason for disease aggravation and record the reason on eCRF.

7.8 Laboratory Safety Assessments

Blood and urine samples are collected for laboratory assessments. The collecting date and time are recorded on the laboratory tests application form. The samples are processed in the central laboratory and the results are reported to study sites within 72 hours.

All subjects are suggested to go to each visit in fasting state between 7:00 am and 10:00 am. The subjects should be informed to avoid heavy physical activities and intake of high fat and high calorie food one day before the visit. Food and beverage except for water are prohibited within 8 hours before the visit. Also, the subjects are informed of not taking the study medications by themselves in the morning of the visit day. Other permitted medications can only be administered with water.

All samples are collected by authorized study staffs, and processed per manuals in the central laboratory. The latest laboratory reference range is provided to study sites during the study. The laboratory results are compared with the reference range values. Results exceeding the normal range are marked. The Investigator evaluates the abnormal results and decides whether they are clinically significant or not.

See Section 7.1.3 for how to record and report AEs relevant to laboratory tests.

The following laboratory safety parameters are measured:

- Hematology: hemoglobin, hematocrit, erythrocytes count, leukocytes count, neutrophils, basophils, eosinophils, monocytes, lymphocytes, and platelet count
- Urinalysis: pH, specific gravity, protein, glucose, erythrocytes, leukocytes, and ketone bodies
- Blood biochemistry: glucose, total bilirubin, ALT, AST, gamma-glutamyl transferase, alkaline phosphatase, albumin, total protein, lactate dehydrogenase, urea nitrogen, creatinine, uric acid, sodium, potassium, chloride, calcium, creatine kinase, creatine kinase isoenzyme, total cholesterol, high-density lipoprotein cholesterol, low-density lipoprotein cholesterol, and triglyceride.

All methods, instruments, and quality control requirements for laboratory tests are described in detail in the manual of central laboratory.

7.9 Physical Examinations

Full physical examination is conducted at each visit except Visit 2, Visit 3 and Visit 16. The physical examination includes a check on: general appearance, head and neck, chest (heart and lung), abdomen, and limbs.

Any finding from physical examination at subsequent study visits is compared with the results at screening visit and is evaluated for clinical significance. See Section 7.1.3 AEs Related to Examinations and Laboratory Tests for how to record and report AEs related to examinations.

7.10 12-lead ECG

A 12-lead ECG examination is conducted at each visit except Visit 2, Visit 3 and Visit 16. The Investigator evaluates the ECG results and record the results on eCRF as “normal” or “abnormal with/without clinical significance”. See Section 7.1.3 AEs Related to Examinations and Laboratory Tests for how to record and report AEs related to laboratory examinations.

7.11 Vital Signs

Vital signs (blood pressure, pulse rate, respiratory rate and body temperature) are measured according to study protocol. Height and body weight are included as part of vital sign assessments.

The subjects should rest for at least 5 minutes before vital sign assessments. Throughout the study, all efforts are made to measure pulse rate, respiratory rate and blood pressure from the same arm. The arm used for measurement is documented in the original records.

Pulse rate

Palpation on the radial artery is suggested to last for at least 30 seconds to calculate the number of beats per minute, which is recorded on eCRF. If necessary, palpation is on bilateral radial artery simultaneously to check the symmetry. It is recommended to be marked on eCRF if there is any asymmetry.

Body temperature

A thermometer is placed at deep axillary region of the subjects. The subjects are told to hold the thermometer tightly and read the temperature 10 minutes later. Be noted that there should not be heating or cooling objects at the axillary region and sweat at the axillary region should be swiped.

Respiratory rate

The subjects are observed for breathing rhythm and breathing frequency per minute is recorded.

Blood pressure

A mercury sphygmomanometer is used for measuring blood pressure. The subject should be in a quiet place with his feet flat on the floor back against the chair. Cigarettes and coffee is prohibited and bladder is emptied within 30 minutes before the assessment. The subject’s upper arm is exposed and stretched slightly with the elbow at the same height with heart. The cuff is closely and evenly wrapped around upper arm with its lower edge at about 2.5 cm above the antecubital fossa and the center of cuff at the surface of brachial artery. After touching the brachial artery, the examiner puts the stethoscope’s bell over the brachial artery for auscultation. Then the cuff is inflated while auscultation. After the sound of brachial artery pulse disappears,

the cuff continues to be inflated until the reading values increase for additional 30 mmHg. Then the cuff is deflated gradually (2–6 mmHg/s). Eyes are kept at the same level with the mercury gauge while the mercury column is descending, and blood pressure is read according to auscultation. Blood pressure is measured at least twice with two-minute interval and the average of the two measurements is calculated. If the difference of the two measurements in systolic or diastolic blood pressure is larger than 5 mmHg, a third measurement is done after 2 minutes and the average of 3 measurements is calculated.

Height and Weight

Shoes and thick clothes should be taken off while height and body weight are being measured. The brand and model of the scales used for body weight measurement in each site should be kept the same throughout the study. BMI=Body Weight (kg)/Height (m)^2.

7.12 Pregnancy

The subject who becomes pregnant while receiving the study medication must immediately discontinue the study medication. Pregnancy itself is not considered as an AE, but it must be reported on the pregnancy form and submitted to the Sponsor as special safety information. The reporting time limit is the same as SAE reporting. All pregnancies must be followed up until the pregnancy outcome is obtained (e.g. spontaneous abortion, selective termination, ectopic pregnancy, full term birth, congenital anomaly). In addition, if a male subject's partner is pregnant during the study, it is also reported to the Sponsor and followed up until pregnancy completion.

Congenital anomaly, birth defects and spontaneous abortion should be reported as SAEs. Artificial abortion without complications do not need to be recorded as AEs. The pregnancy outcomes after database lock should be reported to the clinical drug safety department of the Sponsor. The contact information is: email: drugsafety@huamedicine.com, fax: 021-58863272, mobile: 156 1839 6573. Pregnancy report form refer to ISF.

7.13 Overdose

Overdose is defined as the situation when a subject is given excessive study medication, or the subject himself /herself takes excessive study medication intentionally or accidentally and beyond the dosage defined in study protocol. Overdose is recorded on the medication dosing page of eCRF. If overdose is associated with any AE, it is documented on AE page of eCRF as well. If overdose is associated with any SAE, it needs to be reported according to the procedures in Section 7.3 of this protocol.

8 Data Management

8.1 Data Traceability, Completion, and Transfer of eCRF

Data management of the study is conducted by the data management department of dMed Biopharmaceutical Company Ltd. to assure data integrity, completeness, privacy, and traceability.

Data entry by site Investigator or the delegates must ensure the integrity and accuracy. And any correction and/or addition should be instructed to site personnel. Any correction must comply with the eCRF completion instructions.

8.2 Database Design

Database is designed by the data management department of dMed Biopharmaceutical Company Ltd. per requirements of the “Code of Federal Regulations Title 21, Part 11 (21 CRF Part 11)”. Database should be able to manage the traceability of system login, data entry, data update, and data deletion. Database design should follow the CDISC standards.

8.3 Data Entry

The Investigators and delegates are responsible for entering study data in eCRF. Required EDC training must be provided and completed prior to study initiation to ensure mastery of the EDC system and eCRF layout. Data entered into EDC must be validated to ensure data completeness and accuracy.

8.4 Data Validation

Data Validation includes programmed edit check per study design and manual review. Any discrepancy occurred during data validation must be clarified by Investigator and corrected timely.

8.5 Blinded Data Review and Unblinding

The study should be unblinded at the completion of the double-blind period for all subjects. Suggested by ICH E9 Biostatistical Principles for Clinical Trials, blinded data review should be conducted before unblinding to achieve the following two purposes:

- Determine population set during blinded data review
- Review blinded study data

It should be assured that the database is locked before unblinding.

Emergency Unblinding

Emergency unblinding is only applicable to emergency situations. The randomization code can only be unblinded under emergency medical conditions (e.g., treatment can only be given when the actual study treatment group of this subject is known). The investigator can discuss the

potential unblinding case with the Sponsor and use the IWRS system (Medidata's Balance) to unblind the randomization code, which tells the actual treatment group that subject is randomly assigned to. If randomization code is unblinded, the time, date, and reason of unblinding must be recorded in the system, and signed off by the individual who performs the unblinding. The Sponsor and Medidata staffs shall be notified immediately.

If any unexpected SAE occurs or any suspicious causality between study drug and SAE, the case should be reported immediately to regulatory agencies. Hua Medicine reserves the right to unblind the randomization code or not for such cases.

Monitor must check the blinding status of any subject at each scheduled visit to assure the blind status of each subject is intact, and/or record is complete for each emergency unblinding case.

8.6 Database Lock

Two stages are planned for database lock in this study.

The first database lock is scheduled at the completion of the double-blind treatment for all subjects. After the blinded data review meeting, the principle Investigator, Sponsor, and biostatistician must sign off all related documents before the database lock (DBL). DBL is limited to the double-blind treatment data, but not the data in the open-label treatment period. No update is permitted to locked data in principle. Errors found after DBL should be evaluated to determine whether database unlock is necessary for data correction. Documentation must be recorded if database unlock is required.

The second database lock is scheduled at the completion of the open-label treatment. A regular data review meeting is required before the final DBL. The Investigator, Sponsor, and biostatistician should sign off relevant documents before DBL. In principle, no update is permitted to locked data. Errors found after database lock should be evaluated to determine whether database unlock is necessary for data correction. Documentation must be recorded if database unlock is required.

9 Statistical Analysis

9.1 Sample Size Determination

This study plans to recruit 750 subjects. Considering possible drop-out of the subjects, a 20% increase of the total subjects, but no more than 938 subjects, will be enrolled. These subjects are randomized at a ratio of 1:1 into the treatment group (HMS5552 75mg BID + stable dose of metformin) or control group (placebo BID + stable dose of metformin), i.e. no more than 469 subjects and 469 subjects are randomized to the treatment group and control group.

Based on the planned sample size and the randomization ratio (750 subjects, treatment group: control group = 1:1, the study has 95.4% power to detect the treatment group is superior to the placebo group with the assumption of type I error (alpha) at 0.05 (two-sided), within-group

standard deviation for the primary efficacy endpoint as 1.5% and the treatment difference (treatment group vs control group) as -0.4%.

9.2 Analysis Population Sets

Full analysis set (FAS): Based on the intention-to-treat (ITT) analysis principle, the FAS consists of all randomized subjects who receive at least one study dose and have at least one measure of the primary efficacy endpoint post randomization.

Per-protocol set (PPS): The PPS is a subset of FAS consisting of all subjects who have no major protocol deviations. The subjects in PPS are discussed and determined in the blinded review meeting prior to the database lock.

Safety set (SS): The SS comprises of all randomized subjects who receive at least one study dose.

The FAS is considered as the primary efficacy analysis, whereas safety assessment is performed on SS.

9.3 Statistical Methods

After all randomized subjects complete the 24-week double-blind period, the first stage database lock is performed. The study is unblinded for statistical analysis. When all subjects completed the 28-week open-label treatment period, the second database lock and statistical analysis is performed. The details of statistical analysis methods and description are provided in the separate Statistical Analysis Plan. The biostatisticians should formulate the Statistical Analysis Plan according to the clinical study protocol, should record in details the changes that deviate from the planned analysis in the protocol, and refine the final document before database lock and unblinding.

Statistical analysis will be performed using the statistical analysis software SAS version 9.2 or above. The significant level for between-group comparison is set as 0.05 (two-sided).

9.3.1 Demographics and Baseline Characteristics

The demographics is summarized for all randomized subjects (double-blind period) and those who received open-label treatment (open-label period). Summary tables or figures are used to describe the baseline characteristics between the two treatment groups. The robustness of randomization is justified by the descriptive statistics, but no statistical testing is performed on baseline characteristics.

The number and percentage of subjects who are screened, randomized (double-blind period), and received open-label treatment (open-label period) is summarized separately by study period. The primary reasons of screen failure, randomization failure and withdrawal from the study is

summarized by treatment group.

For continuous variables, the descriptive statistics such as sample size, mean, standard deviation (SD), median, and range (minimum-maximum) are provided. For categorical variables, the number and percentage of subjects for each category are calculated.

The baseline characteristics are summarized for all subjects who are randomized (completed Visit 4 in double-blind period) and receive open-label treatment (completed Visit 10 in open-label period). Both the FAS and PPS populations are used for summary of baseline characteristics.

9.3.2 Efficacy Analysis for Primary Endpoint

The primary efficacy endpoint is the change of HbA1c from baseline at the end of double -blind period (at Week 24). The mixed model for repeated measures (MMRM) is used for the primary endpoint analysis. The model includes groups, visits, the interaction between groups and visit, sites, disease duration and baseline HbA1c value. Treatment difference of the mean HbA1c change from baseline at each visit is estimated by MMRM. An unstructured variance-covariance matrix is preferred in the primary model.

The adjusted least-squares mean estimate and 95% confidence interval (CI) of treatment difference (treatment group-placebo group) in the primary efficacy endpoint is calculated. The following hypotheses are tested:

- Null hypothesis: at the end of double-blind treatment (Week 24), the HbA1c reduction from baseline in the two groups has no difference.
- Alternative hypothesis: at the end of double-blind treatment (Week 24), the HbA1c reduction from baseline in the two groups has significant difference.

The significant level of the between-group comparison is 0.05 (two-sided). If p value is smaller than 0.05, it is considered that the between-group difference is statistically significant. If the upper limit of the 95% CI is smaller than 0, it is considered that HMS5552 is superior to the placebo in treatment effect.

9.3.3 Efficacy Analysis for Secondary Endpoints

The secondary efficacy endpoints include changes from baseline at the end of double-blind period (at Week 24) of 2h-PPG, and FPG. The same analysis method for the primary efficacy endpoint is used, i.e. MMRM. The adjusted least-squares mean difference between HMS5552 and placebo, and its 95% CI is estimated by MMRM.

HbA1c response rate is calculated based on the data imputed by last observation carried forward (LOCF). The HbA1c response rate, i.e. number and percentage of subjects whose HbA1c is less than 7.0% at the end of double-blind treatment period (at Week 24) are summarized. The HbA1c

response rate and 95% CI for each group are estimated using the logistic regression method.

The measurement and changes of HbA1c, FPG and 2h-PPG from the end of double-blind period (Week 24) to the end of study (Week 52) are summarized by two groups: one group is for subjects who are randomized into HMS5552 treatment group, and the other group is for subjects who are randomized into placebo group while switched to HMS5552 open-label treatment after double blind period (Week 24). The measurements at the end of double-blind period (Week 24) are used as baseline for the summary of the measurements and change from baseline at each visit in the open-label period.

9.3.4 Safety Analysis

Descriptive analysis is performed on SS. Safety data are summarized and evaluated for the following two stages: 1) Stage 1: when the subjects completed the double-blind period, and 2) Stage 2: when the whole study is completed.

All AEs are coded using the MedDRA dictionary. The incidences and severity of AEs, and SAEs, etc. are summarized and compared between two treatment groups. The detailed description of the treatment emergent SAE and study discontinuation due to AE is listed.

The incidence of hypoglycemic events is compared between treatment groups. The incidence ratio and 95% CI of each treatment group, as well as the incidence difference between treatment groups and its 95% CI are calculated.

The change from baseline of laboratory tests, physical examinations, vital signs, and 12-lead ECG measurements at each visit are summarized. Each index before and after treatment of normal/abnormal changes is also summarized.

9.3.5 Biomarker Analysis

This section is not applicable.

9.3.6 Sensitivity Analysis

Sensitivity analysis is applicable to the primary efficacy endpoint.

The LOCF approach is performed to impute missing primary efficacy endpoint. For subjects with early withdrawal, the last available post-treatment measurement is carried forward. The analysis of covariance (ANCOVA) model is used for analyzing the imputed data. The ANCOVA model includes baseline HbA1c value, disease duration, sites and groups as covariate, with or without the interactions between center and group. If p value of the between-group comparison is smaller than 0.05, it is considered that the between-group difference is statistically significant. If the upper limit of the 95% CI is smaller than 0, it is considered that HMS5552 is superior to placebo in treatment effect.

9.3.7 Interim Analysis

No interim analysis is planned during the double-blind period. Statistical analysis is planned for two stages: 1) Stage 1 is for double-blind period, and 2) Stage 2 is for open-label period. After the completion of the double-blind period, the eligible subjects will enter the open-label period. No alpha sparing or independent data monitoring committee is required in this study.

10 Population PK/PD （PopPK/PD）Analysis

The PopPK/PD model for HMS5552 in subjects with T2DM is updated by using the NONMEM/PIRANA/R software based on the current HMS5552 PopPK/PD model and PK/PD data in Chinese subjects with T2DM after HMS5552 treatment. The quantitative relationship between HMS5552 concentration and HbA1c level is established. Systemic quantitative analysis is conducted on the influences of various factors on HMS5552 PK/PD characteristics, e.g., demographic characteristics, disease course, and concomitant medications. The specific methods and information for analysis of PopPK/PD model are elaborated in the population PK/PD analysis plan.

11 Trial Management

11.1 Declaration

This study is conducted in full compliance with the Declaration of Helsinki, China Good Clinical Practice (GCP), China clinical study laws and regulations.

11.2 Ethics Committee

In accordance with China regulatory requirements, all study related documents are provided to ethics committee (EC) by the Investigator.

Before the study drugs are shipped to the Investigator, the copy of EC approval and the list of required documents must be provided to the Sponsor. The documents approved by EC should include a list of EC members who have taken part in the discussion for document approval.

Upon receipt of the EC approval, the Sponsor/CRO should submit the study related documents to the relevant drug evaluation authority for record.

Approval of the study from EC and drug administration department must be obtained before the study starts.

Protocol amendments need to be submitted to EC for approval. The health bureau is also notified per local requirements.

During the study, the Investigator should notify EC of any SAEs or unexpected AEs that are

related to the safety of clinical study and may affect patient safety and study implementation.

The possible benefits and risks for subjects are fully described in the ICF.

11.3 Source Document Verification

The Investigator must handle all data obtained from clinical study properly to protect the rights and privacy for the subjects participating in the study. The Investigator and the medical institution which he/she works in allow the Sponsor to monitor or audit the study. Health administration and drug administration departments have the right to inspect the study and review data/source documents.

11.4 Quality Control and Quality Assurance

This study can be audited by Sponsor or Sponsored authorized independent personnel for quality assurance, or by drug approval authority. The quality assurance monitor or inspector from drug approval authority can review all study related information and data, including but not limited to medical records, study documents and letters, study required facilities and equipment, study samples and materials, etc.

The audit on clinical trial by Sponsor is a part of quality assurance, which is different from the routine clinical trials monitoring, also different from the quality control and management from a Sponsor's functional department. The objectives of this audit by Sponsor are: to evaluate the implementation of clinical trial, and the compliance with the study protocol, SOP, related law and regulations from Drug supervision and management authorities.

11.5 Subject Informed Consent

The Investigator is responsible for explaining to each subject about the study objectives, study methods, benefits, and potential risks. ICF signed by subjects must be obtained before any study related procedures. The informed consent should be presented both orally and in writing. The ICF also must be dated and signed by subjects. For subjects who cannot sign by themselves for any reason, the ICF must be signed by their parents, legal guardians or legal representatives. The signed ICF is kept by the subjects.

By signing the ICF, subjects/patients must agree to allow the Sponsor, regulatory authorities, auditors and/or monitors to verify the related original data obtained from the study. All the auditing personnel must follow the confidentiality agreement.

11.6 Protocol Amendment

After the protocol is finalized, any protocol amendments must be documented in details and the records for protocol changes need to be signed at least by the Investigator and the Sponsor. The version numbers and dates need to be indicated.

All protocol amendments must be submitted to EC. Written approval must be obtained from EC for significant changes. Local regulatory authorities should also be notified with renewed profiles. All documents should be submitted to the Sponsor. All the procedures should be completed before the updated profile takes effect.

11.7 Monitoring

On-site monitoring is performed by the CRAs from the Sponsor or Sponsor authorized CROs (the CROs' SOPs should be followed). The monitoring visits should be conducted regularly throughout the study.

Monitors authorized by the Sponsor are permitted to review the source data relevant to the study and will verify the data on eCRFs and in other study documents per SOP and GCP requirements, ensuring data integrity, accuracy and consistency with the source documents.

All source documents, data, records, laboratory data/information, eCRF and medical test results must be accessible to monitors, inspectors and the drug administration departments for review at any time.

11.8 Confidentiality Agreement and Patient Privacy

The Investigator must keep the information related to the Sponsor and investigational drug confidential, which is provided or disclosed due to the cooperation in the study. The information can only be used with authorization from the Sponsor.

The commitment to confidentiality is independent, valid, and persistent.

The Investigator should ensure that the confidential information is not disclosed to third parties and is only used within the agreed scope. The confidential information may be obtained from Hua Medicine and its products, or provided or disclosed due to the contractual relationship.

The clinical study staffs must protect subjects' privacy. Only the subject number can be used for subject's identification in all documents submitted to the Sponsor. Subjects' names and hospitalization IDs cannot be used. The Investigator must properly preserve the relevant documents related to subjects' names, addresses and the screening IDs/randomization IDs. All subjects' information should be securely preserved by the Investigator in accordance with GCP regulations.

12 Publication

As the Sponsor, Hua Medicine (Shanghai) Ltd. has the exclusive ownership for the publications in this study. Authors and publications reflect the collaboration among multiple investigators, study sites and staffs from Hua Medicine (Shanghai) Ltd. Authors should be determined before preparing the manuscript. Unless approved by Hua Medicine (Shanghai) Ltd., any person is not allowed to have any publication before the final clinical study report is completed. Hua

Medicine (Shanghai) Ltd. has the right to make final decision on all manuscripts and publications.

13 Documents Archiving

In accordance with relevant regulations, the Investigator should maintain all the medical source documents properly. All study documents should be kept for at least 5 years after the drug is marketed. The Sponsor is responsible for informing the Investigator/study sites when the study documents do not need to be kept any more.

14 Appendix

Appendix 1 Diagnosis of Diabetes Mellitus

(From China Guideline for Type II Diabetes prevention and treatment, Version 2013, CDS[11])

Clinical diagnosis for diabetes mellitus should be based on the blood test results of venous plasma glucose rather than capillary blood glucose. If not specified, the blood glucose mentioned in this article refers to the venous plasma glucose.

The normal range of blood glucose and the diagnosis cutoff point for glucose metabolism abnormality are mainly confirmed by the relationship between the value of blood glucose and the risk of specific chronic diabetic complications (diabetic retinopathy) and diabetes mellitus occurrence. Currently, the commonly used diagnostic criteria and classification are the WHO standards (1999) and the ADA (American Diabetes Association) standards (2003).

This guideline adopts WHO Standards (1999) for diabetes diagnosis, classification of glucose metabolism state, and the classification system of diabetes mellitus (Table 1 and 2). Fasting Plasma Glucose (FPG) or the venous plasma glucose value 2 hours after 75g Oral Glucose Tolerance Test (OGTT) alone can be used for epidemiological investigation or population screening. However, based on the data in our country, the missed diagnosis rate is high if only FPG is tested. Ideally, both FPG and venous plasma glucose 2 hours after OGTT need to be tested. Blood glucose at other time points after OGTT are not used as diagnostic criteria.

The population with impaired glucose regulation is suggested to take the OGTT to reduce the missed diagnosis rate.

Table 1 Classification of Glucose Metabolism State (WHO 1999)

Classification of Glucose Metabolism	Venous Plasma Glucose (mmol/L)	
	Fasting Plasma Glucose	2 hours Postload Plasma Glucose
Normal Plasma Glucose	< 6.1	< 7.8
Impaired Fasting Glucose (IFG)	6.1 ~ < 7.0	< 7.8
Impaired Glucose Tolerance (IGT)	< 7.0	7.8 ~ < 11.1
Diabetes Mellitus	≥ 7.0	≥ 7.0

Note: both IFG and IGT are called as impaired glucose regulation, also known as prediabetic state.

Table 2 Diagnostic Criteria for Diabetes Mellitus

Diagnostic Criteria	Venous Plasma Glucose (mmol/L)
(1) Typical diabetic symptoms (polydipsia, polyuria, polyphagia, and weight loss), with random plasma glucose test	≥ 11.1
Or, *with*	
(2) Fasting plasma glucose test	≥ 7.0
Or, *with*	
(3) Blood glucose test 2 hours postload glucose<br>People without diabetic symptoms needs re-test on different day	≥ 11.1

Note: The fasting status means no calories intake for at least 8 hours. random plasma glucose means plasma glucose tested at any time regardless of the last meal time and can't be used for IFG and IGT diagnosis.

Appendix 2 New York Heart Association (NYHA) Classification

NYHA Classification:

I. Cardiac patients with no limitation in daily activity. ordinary physical activity will not lead to excessive fatigue, palpitations, dyspnea or angina.

II. Cardiac patients with slight limitation in physical activity. Asymptomatic at rest, ordinary physical activity will cause excessive fatigue, palpitations, dyspnea or angina.

III. Cardiac patients with obviously limitation in physical activity. Asymptomatic at rest, while less-than-ordinary activity cause excessive fatigue, palpitations, dyspnea or angina.

IV. Cardiac patients who cannot stand for any physical activity. Symptoms of heart failure occurs even at rest, which is aggravated when undertaking physical activity.

Appendix 3 Procedure of Mixed Meal Tolerance Test (MMTT)

The MMTT is a standardized method to determine postprandial blood glucose levels. In this study, MMTT is used for assessing how HMS5552 affects postprandial blood glucose, insulin and C-peptide. (Note: All meals used for MMTT in this study are the instant noodles standard meal provided by Sponsor).

Subjects are requested to avoid any heavy physical activities and high fat and high calorie food on the night prior before MMTT visit.

On the day of MMTT visit, subjects should be in fasting status (suggest no food and beverage intake from 22:00 at previous night until blood sampling is completed. water is allowed and fasting status should be kept for at least 8 hours).

Totally four MMTTs will be conducted for the whole study, including one at Visit 4 before study drug administration, and once each at Visits 7, 10 and 15 after study drug administration.

Before the instant noodles standard meal, blood samples need to be collected at the timepoint 0 and then the instant noodles standard meal starts to be taken (timing starts from the first chewing of the noodles and the meal is finished within 10 minutes). Blood samples are collected at 30 minutes (±3 minutes are allowed), 120 minutes (±5 minutes are allowed) after the instant noodles standard meal. When blood samples are being taken, food, water, and strenuous exercise is not allowed until all samples for MMTT are collected. (Blood sampling at 30 minutes applies to the subjects who enroll after the Version1.1 profiles take effect)

If subjects withdraw from the study early and already discontinued study medications on the termination visit, then MMTT for the termination visit can be cancelled (other tests are the same as Visit 15).

Procedures for Taking the Instant Noodles Standard Meal

1. Adding seasoning: Take out the seasoning bag and add the seasoning to the noodles.

2. Cooking: Add approximately 550 ml boiling water (suggest to use immediately once the water is boiling) to the noodles. Cover the lid and start timing.

3. Stirring: After 2 to 2.5 minutes, open the lid and stir the noodles. Cover the lid again.

4. Noodles ready: Use chopsticks to stir the noodles well when they are cooked for 10 to 15 minutes.

5. Eating: Eat all the noodles within 10 minutes. The soup can be finished or left according to subjects' personal preference.

Appendix 4 Procedures for SMBG

At Visit 2, blood glucose meters with matching blood collection needles and test strips are provided to all subjects. Subjects are instructed for SMBG. Subjects monitor fingertip blood glucose levels (fingertip blood glucose tests for FPG or PPG are required to be conducted at least twice a week. the frequency is increased when hypoglycemia is suspected. All test time and results are recorded in subject diaries) at appropriate frequency decided by the Investigator (based on the assessment on the risks of subjects' blood glucose concentration).

Throughout the entire study (Visit 2 to Visit 16), to give subjects safety guidance and assess the withdrawn criteria timely, the Investigator should ask subjects to contact research centers immediately if the results of fingertip plasma glucose test meet the following criteria:

1. Hypoglycemia. Visit 2 (-Week 4) to Visit 15 (Week 52), at any time, fingertip plasma glucose value ≤ 3.9 mmol/L.

2. Hyperglycemia.

   Day 1 to before Week 12, fasting plasma glucose (central laboratory) ≥ 13.3 mmol/L.

   Week 12 to before Week 24, fasting plasma glucose (central laboratory) ≥ 11.1 mmol/L.

   Week 24 to Week 52, fasting plasma glucose (central laboratory) ≥ 11.1 mmol/L.

Blood glucose results are recorded by subjects in subject diaries. At each visit after Visit 2, subjects are required to take blood glucose meters and subject diaries to research centers for Investigator review.

Appendix 5 Estimated Glomerular Filtration Rate (eGFR) Measurement

Calculation of eGFR with MDRD equation[19]

1. $eGFR = 175 \times [S_{cr}]^{-1.154} \times [Age\ (Year)]^{-0.203} \times [\times 0.742 \text{ if female}]$

   Cr unit: mg/dL, Age unit: year

2. $eGFR = 175 \times [S_{cr} \times 0.01131]^{-1.154} \times [Age\ (Year)]^{-0.203} \times [\times 0.742 \text{ if female}]$

   Cr unit: µmol/L, Age unit: year

Appendix 6 Rules for Managing Abnormal Liver Functions

The Investigator should be highly alert with increased liver biochemistry values during the study, and should ensure subjects' safety and assess the cause of abnormal liver function timely.

For abnormal liver biochemistry values occurring at different stages of the study, the below actions are suggested.

Screening and Run-in Periods

1. Subjects with active liver disease are screen failures.
2. Subjects with ALT or AST > 2.5×ULN or total TBiL > 1.5×ULN are screen failures.
3. If a subject has ALT or AST values higher than the upper limit of normal range but are ≤ 2.5×ULN, or TBiL higher than the upper limit of normal range but are ≤ 1.5×ULN, the Investigator is suggested to find out the cause for abnormal liver function parameters (the Checklist for Collecting Abnormal Liver Function Information in the Investigator's folder can be used). Subjects can continue the subsequent study only when the Investigator can ensure the abnormal liver function will not affect the safety assessment or subjects' completion of the entire study or study procedures.

Treatment Period

From Visit 4 to Visit 15 in this study

1. The Investigator can stop study medications based on subjects' actual condition if any of the following criteria is met (no need to wait for the retest results):
   - ALT or AST > 8 × ULN
   - ALT or AST > 5×ULN and lasts for ≥ 14 days
   - ALT or AST > 3×ULN and TBiL > 2×ULN
   - ALT or AST > 3×ULN with liver damage symptoms (e.g., fatigue, nausea, vomiting, right upper abdominal pain or tenderness, fever, rash) or eosinophilia increase.

The Investigator should immediately provide active intervention or treatment to subjects and identify the cause. If necessary, consultation with hematologists is conducted. The Investigator should closely follow-up with the subjects until the liver function is recovered or returns to baseline status. The Investigator should record the relevant AEs or SAEs timely.

2. If subjects have ALT or AST> 3×ULN but do not meet withdrawal criteria, the Investigator must ask the subjects to come back to the research center and retake the liver function tests at the central laboratory within 48 hours after receiving the test report (local laboratory tests can

be done when necessary). The Investigator should record the relevant AE or SAE timely, and find out the cause for abnormal liver functions, and give necessary interventions. The Investigator should closely follow-up with the subjects until the liver function is recovered or returns to baseline status. The subjects can continue to receive treatment according to the visit schedule in the study protocol if the Investigator considers appropriate.

3. If subjects have ALT or AST higher than the upper limit of normal range but is≤ 3×ULN, the Investigator evaluates the clinical significance of the abnormal values (see Section 7.1.3 Adverse Events Related to the Medical and Clinical Laboratory Examinations). If any AE criterion is met, an AE should be recorded. The Investigator needs to find out the cause for abnormal liver functions and give necessary interventions. The Investigator should closely follow-up with the subjects until the liver function is recovered or returns to baseline status. The subjects can continue to receive treatment according to the visit schedule in the study protocol if the Investigator considers appropriate.

Safety Observation Period

All study drugs are stopped in the safety observation period. All new occurrence of clinically significant abnormal liver function at the last visit should be reported as an AE (refer to Section 7). The Investigator should give necessary interventions and follow-up with the subjects until the liver function is recovered or returns to baseline status.

Clarify the cause of abnormal liver function (for reference)

The Investigator figures out causes of abnormal liver function based on subjects' actual medical situation or clinical experience. The Investigator can also refer to the following items for clarification (e.g. inquiry, physical examination or laboratory tests).

Medical history and related risk factors: e.g. short-term drinking history, drug abuse, acute drug overdose, blood transfusion history, unclean seafood intake, occupational toxic contact, exposure to jaundice or other high-risk population, familial liver disease history, etc..

Physical examination: if any hepatomegaly, jaundice and other symptoms/signs occur.

Concomitant medications: if any prescription or non-prescription drugs or herbs or health care products been used and may lead to liver injury before the occurrence of liver injury, e.g. acetaminophen, paracetamol, carbamazepine, chlorpromazine, chlorpropamide, steroid contraception drugs, erythromycin ethylsuccinate, non-urethane, halothane, ketoconazole, monoamine oxidase, nonsteroidal anti-inflammatory drugs, phenindione, phenylbutazone, phenytoin, sulfonamides and sodium valproate, etc..

Hematological examination results: e.g. prothrombin time (PT), hepatitis A, B, C, D, E virus related tests, anti-cytomegalovirus antibodies, EB virus antibodies, anti-smooth muscle antibodies, anti-mitochondria antibodies, anti-neutrophil cytoplasmic antibodies, etc.

Other tests: e.g. abdominal ultrasound, CT or MRI if needed.

If necessary, hepatologists are consulted.

Appendix 7 Recommendations for Managing Hypoglycemia

(for reference)

1. When suspected hypoglycemia symptoms appear (e.g. palpitation, anxiety, sweating, hunger etc, or altered mental status, cognitive impairment, convulsion, coma, etc.), blood glucose level should be measured immediately for a concise diagnosis. If blood glucose is difficult to measure, treatment for hypoglycemia is given temporarily.

2. Treatment for new occurrence of hypoglycemia

1) Subject with clear consciousness. Take glucose or sugary food as soon as possible (suggest taking 15 to 20 grams of sugary food orally, glucose is preferred).
   Caution: to avoid a delay in the sugar absorption, don’t eat any foods immediately after taking sugars described above.
2) Subject with no consciousness or coma. Call 120 and send the subject to hospital immediately. Don’t put food, liquid, or hand into the subject’s mouth. Keep the subject’s airway open. Recommended treatment: intravenous injection of 20 to 40 mL 50% glucose solution or intramuscular injection of 0.5 to 1.0 mg glucagon.

3. After the treatments mentioned above, blood glucose level is monitored every 15 minutes.

1) If plasma glucose level is still ≤ 3.9 mmol/L, oral intake of sugary food or glucose injection will be done once again.
2) If plasma glucose level is > 3.9 mmol/L but the time to next meal is more than 1 hour, food consists of starch or protein will be given after 10 to 15 minutes.
3) If plasma glucose level is still ≤ 3.0 mmol/L, intravenous injection of 60 mL 50% glucose solution will be given.

4. Once hypoglycemia has been corrected

1) Find out the cause for hypoglycemia and adjust the drug administration. For subjects with impaired consciousness, plasma glucose level in short-term does not need to be strictly controlled.
2) Pay attention to cardiovascular diseases induced by hypoglycemia.
3) Frequent SMBG is suggested.
4) Increase diabetic education and carry diabetes emergency cards.

If hypoglycemia has not been corrected,

1) Intravenous injection of 5 or 10% glucose, or combined with glucocorticoid.
2) Monitor blood glucose for at least for 24 to 48 hours after recovery of consciousness.

Appendix 8 Medical Nutrition Therapy for T2DM

(Chinese Guideline for the Prevention and Treatment of T2DM (2013 Edition), Chinese Diabetes Society[11])

Medical nutrition therapy is a special intervention approach in treating the nutritional problems in patients with diabetes mellitus, including personalized nutrition assessment, nutrition diagnosis, relevant nutritional intervention plan to be implemented and monitored in a certain period of time. Medical nutrition therapy is important in the prevention, treatment, self-management, and education for diabetes mellitus and relevant complications. By modifying nutrient structure, medical nutrition therapy is beneficial in blood glucose control and helps to maintain optimal body weight and prevent malnutrition.

General principles for nutrition therapy

Subjects with diabetes mellitus and prediabetes need to receive personalized medical nutrient treatment under the guidance of a registered dietitian who is familiar with diabetes treatment or an integrated management team (including diabetes educators). After nutrition condition of the patient is assessed, a reasonable target should be set for controlling the total energy intake, maintaining appropriate and balanced distribution of various nutrients, and achieving metabolic control goal. Personal food preferences are satisfied as much as possible. Moderate body weight loss, which can be maintained by physical exercises and behavior changes, is recommended for overweight or obese patients.

Objectives for medical nutrition therapy

To maintain reasonable body weight: the goal for overweight or obese patients is to achieve 5% to 10% weight loss within 3 to 6 months. Subjects of marasmus should restore and maintain optimal body weight through well-designed nutrition programs. With balanced diet, to achieve and maintain optimal blood glucose level and decrease HbA1c level. To reduce the risk factors for cardiovascular diseases, including the management of dyslipidemia and high blood pressure. To reduce insulin resistance and the load for islet beta cells.

Nutrients

Fat

The energy from fat should be no more than 30% of the total dietary energy. Intake of saturated fatty acids should be less than 7% of the total dietary energy. Intake of trans fatty acids should be reduced as much as possible. Monounsaturated fatty acids are good source of dietary fat and should account for 10 to 20% of total fat intake. Intake of polyunsaturated fatty acids should be less than 10% of total energy intake. Intake of food rich in n-3 rich fatty acids should be properly increased. The dietary cholesterol intake should be less than 300 mg/d.

Carbohydrate

Carbohydrate should account for 50 to 60% of the total dietary energy. The quantitation, assessment and measurement for dietary carbohydrates are key steps in blood glucose control. Foods of low glycemic index are beneficial to blood glucose control. Reasonable intake of sugar alcohol and non-nutritive sweeter is safe. However, fructose produced from excessive sucrose decomposition or adding excessive fructose can lead to increase triglyceride synthesis and body fat accumulation. Everyday meals need to be taken regularly to keep carbohydrate intake balanced every day.

Protein

For individual diabetes patient with normal renal function, protein intake is suggested to account for 10 to 15% of the total energy. The proportion of high-quality protein should be over 50%. Subjects with presence of proteinuria should restrict their protein intake to 0.8g/kg•day. Subjects with decreased glomerular filtration rate (GFR) should take low protein diet with the recommended protein intake of 0.6g/kg•day. To prevent protein malnutrition, compound alpha-keto acid can be used. Simple protein intake is unlikely to cause hyperglycemia but it may increase insulin secretion.

Drinking

Alcohol is not recommended for patients with diabetes mellitus. If subjects drink alcohol, the energy from alcohol should be calculated. Daily alcohol consumption for females shouldn't be more than 15 g. Daily alcohol consumption for males shouldn't be more than 25g (15g alcohol is equivalent to 450 ml beer, or 150 ml wine, or 50 ml low alcohol liquor). Alcohol drinking should be no more than twice a week. Attention should be paid to alcohol-induced hypoglycemia. Alcohol drinking in fasting status should be avoided. Individuals with risks for T2DM should restrict intake of sugary drinks.

Dietary fiber

Beans, fiber-rich cereals (≥ 5g fiber per serving), fruits, vegetables, and whole grains are good sources of dietary fiber. Increasing fiber intake is good for health. Diabetes patients are suggested to have recommended dietary fiber intake, i.e. 14g/1000 kcal per day.

Salt

Daily salt intake should be restricted to less than 6g/day. Patients with hypertension should further restrict their salt intake and food rich in salt, e.g. monosodium glutamate, soy sauce, processed food such as salted food, etc..

Micronutrient

Diabetic subjects are prone to deficiency of vitamin B, vitamin C, vitamin D and many micronutrients such as chromium, zinc, selenium, magnesium, iron, manganese. Micronutrient could be supplemented based on the nutritional assessment results. Patients with long drug history of metformin should be prevented from vitamin B12 deficiency.

Deficiency

Long-term intake of large quantities of antioxidants, such as vitamin E, vitamin C and carotene, is not suggested because their long-term safety remains to be verified.

Dietary pattern

Different dietary patterns, such as the Mediterranean diet, vegetarian diet, low carbohydrate diet, low fat and low energy diet, or high protein diet, are all beneficial to body weight control in short term. However, this should be done under the guidance of qualified healthcare professionals, and meanwhile, change of blood lipids and renal functions is monitored.

Exercise therapy for T2DM

Physical exercise plays an important role in the integrative management of patients with T2DM. Regular physical exercise can improve insulin sensitivity and glucose control, reduce cardiovascular risks, reduce body weight, and increase feeling of happiness. Physical exercise is also proved to be effective in primary prevention for the population with high risk of diabetes. Epidemiological studies show that regular physical exercise for more than 8 weeks helps to reduce the HbA1c by 0.66% in subjects with T2DM, and the mortality rate significantly reduced in subjects who adhere to regular physical exercise for 12 to 14 years.

When patients with T2DM are doing physical exercise, the following rules need to be followed. Exercise therapy should be carried out under the guidance of physician. Necessary assessments need to be conducted before physical exercise, especially the medical assessment on cardiopulmonary function and motor function (such as exercise stress test, etc.). Subjects are not permitted to do physical exercise in the following situations: fasting plasma glucose > 16.7 mmol/L. recurrent hypoglycemia or blood glucose fluctuations. diabetic ketoacidosis and other acute metabolic complications. complicated acute infection. proliferative retinopathy. severe nephropathy. severe cardiovascular and cerebrovascular diseases (unstable angina, severe arrhythmia, transient cerebral ischemic attack). Normal activities can be resumed gradually after the disease status becomes stable. Adult diabetic subjects need to do aerobic exercise of moderate intensity (50 to 70% of maximum heart rate, a little strenuous, heart rate and breathing rate increase without shortness) for at least 150 minutes every week (e.g. 30 minutes per day, 5 days per week).

Studies have shown that even short-time (such as 10 minutes, totally 30 minutes/day) physical exercise is beneficial. Moderate intensity activities include fast walking, tai chi, cycling, table tennis, badminton, and golf. Vigorous intensity activities include dancing, aerobics, jogging, swimming, and cycling to uphill. Subjects are suggested to have resistance training twice a week to improve their muscular strength and endurance, if they do not have any contraindications. The resistance exercise should be mild or moderate. The combination of resistance exercise and aerobic exercise can improve the metabolism more greatly. Exercise plan should be adapted to subjects' age, disease status and physical endurance and assessed and adjusted regularly. Keeping exercise diaries is helpful to improve the exercise compliance.

Developing healthy habits and active lifestyle, such as increasing daily physical activities and

reducing sedentary time, to integrate beneficial sports into daily life. To prevent hypoglycemia, subjects should be monitored for blood glucose before and after physical exercise. Diet and treatment is suggested to be adjusted temporarily after strenuous exercise.

Smoking Cessation

Smoking is harmful to health and is associated with higher risks of cancer, diabetic macrovascular diseases, diabetic microangiopathy, and early death. Studies have shown that for newly diagnosed T2DM patients, smoking cessation is helpful in improving metabolic parameters and decreasing blood pressure and albuminuria. All diabetic subjects are suggested to stop smoking or using other tobacco products. Subjects are evaluated for smoking and the nicotine-dependence status. Short consultation, smoking cessation hotline, and necessary medications are provided to subjects for smoking cessation.

15 References

[1] Yang W, Lu J, Weng J, et al., Prevalence of diabetes among men and women in China. The New England Journal of Medicine 2010；362(12):1090-1101.

[2] Jiamin Chi, Practice of Diabetology, Version 4, Jul2015.

[3] Chinese guidelines for the prevention and treatment of type 2 diabetes mellitus (2013 Edition), Chinese Diabetes Society. Chinese Journal of Diabetes 2014,7:447-498.

[4] Matschinsky FM. Assessing the potential of glucokinase activators in diabetes therapy. Nature Reviews Drug Discovery 2009, 8(5):399-416.

[5] Matschinsky FM, Zelent B, Zoliba N, et al., Glucokinase activators for diabetes therapy, Diabetes Care, 2011 (34), S236-S243.

[6] HMS5552 Investigator's Brochure, Hua Medicine (Shanghai) Ltd., 2017.03, version 3.0.

[7] Phase Ia clinical study report of single ascending dose of HMS5552 (a randomized, double-blind, placebo-controlled safety, tolerability, pharmacokinetics and pharmacodynamic study of administrating single ascending dose of HMS5552 in healthy adult volunteers, serial number: HMM0101), Hua Medicine (Shanghai) Ltd., 2014.05.

[8] Hongrong Xu, et al., Safety, tolerability, pharmacokinetics, and pharmacodynamics of novel GK activator HMS5552: results from a first-in-human single ascending dose study. *Drug Design, Development and Therapy*.09May2016.10:1619-26.

[9] Phase Ib clinical study report of multiple ascending doses of HMS5552 (a randomized, double-blind, placebo-controlled study to assess the safety, tolerability, pharmacokinetics, and pharmacodynamics of multiple ascending doses of HMS5552 in adult subjects with type 2 diabetes mellitus, serial number: HMM0102), Hua Medicine (Shanghai) Ltd., Nov2014

[10] Phase Ic clinical study report of HMS5552 (a single-center, randomized, open-label, 24-week treatment study to investigate safety, tolerability, pharmacokinetics and pharmacodynamics in type 2 diabetes mellitus (T2DM) subjects, serial number: HMM0103), Hua Medicine (Shanghai) Ltd., Mar2016

[11] Phase Id clinical study report of HMS5552 (a phase 1, open-label, sequential, multiple-dose, drug-drug interaction study of GK activator HMS5552 and Metformin in subjects with Type 2 Diabetes Mellitus (T2DM), Serial number: HMM0104), Hua Medicine (Shanghai) Ltd., May2016

[12] Phase II clinical study report of HMS5552 (a multi-center, randomized, double-blind, placebo-controlled, 12-week phase II study to evaluate the safety, tolerability, efficacy and population PK of HMS5552 in type 2 diabetic adult subjects, serial number: HMM0201), Hua Medicine

(Shanghai) Ltd., Mar2017

[13] Garber AJ, Abrahamson MJ,Barzilay JI,et al. American Association of Clinical Endocrinologists's comprehensive diabetes management algorithm 2013 consensus statement—executive summary. Endocr Pract, 2013, 19:536-557.

[14] American Diabetes Association. Standards of medical care in diabetes-2014.Diabetes Care,2014,37(Suppl 1): S14-S80.

[15] International Diabetes Federation. Global guideline for type 2 diabetes, 2012.

[16] Guidelines for clinical trials of drug and biologics for the treatment of diabetes, China Food and Drug Administration 15May2012 No [2012] 122

[17] Glucose concentrations of less than 3.0 mmol/L (54 mg/dL) should be reported in clinical trials: A joint position statement of the American Diabetes Association and the European Association for the Study of Diabetes. Diabetes Care 21Nov2016. [Epub ahead of print] PMID: 27872155.

[18] FDA guidance for industry drug-induced liver injury: premarketing clinical evaluation. July 2009 https://www.fda.gov/downloads/Drugs/GuidanceComplianceRegulatoryInformation/Guidances/UCM174090.pdf

[19] Miller WG. Estimating glomerular filtration rate. Clin Chem Lab Med 2009.47(9):1017-9.

Clinical Study Protocol

A 24-week multi-center, randomized, double-blind, placebo-controlled, phase III study to evaluate the efficacy and safety of HMS5552 add-on to metformin with additional 28-week open-label treatment to evaluate the safety of HMS5552 add-on to metformin in type 2 diabetes mellitus subjects

Protocol Number: **HMM0302**

Version: **Version 1.1**

Date: **March 2nd, 2018**

Sponsor： **Hua Medicine (Shanghai) Ltd.**

Address： **No. 275 Aidisheng Rd, Pudong New District, Shanghai**

Confidentiality Statement

The following is for reference only. The confidential information in this protocol belongs to Hua Medicine (Shanghai) Ltd. and is only provided to you as a principal investigator, coordinate investigator, and applicable Institutional Review Board/ regulatory authorities for review. It is strictly prohibited to disclose any information herein to any irrelevant third party without prior written authorization from the Sponsor, except that explanation for potential subjects to sign informed consent form is necessary.

Medical Experts from the Sponsor

Name	Title	Institution	Telephone
Yu Zhao	Sr. Medical Manager	Hua Medicine (Shanghai) Ltd	021-58869997-3274
Xinghua Hou	Medical Director	Hua Medicine (Shanghai) Ltd	021-58869997-3302
Yi Zhang	VP, Clinical Development	Hua Medicine (Shanghai) Ltd	021-58869997-3269

Principal Investigator

Name	Title	Institution	Address
Wenying Yang	Chief Physician/Director of Endocrinology Department	China-Japan Friendship Hospital	East Yinghuayuan St, Chaoyang District, Beijing

Sponsor

Company	Hua Medicine (Shanghai) Ltd.		
Address	No. 275 Aidisheng Rd, Zhangjiang High Technology Park, Shanghai	Post Code	201203
Telephone	021-58869997	Fax	021-58866110

Contract Research Organization (1)

Company	Covance Pharmaceutical Research and Development (Beijing) Co., Ltd., Shanghai Branch		
Address	Rm 602-606, Bldg A Pinzun International, 555 Langao Rd., Putuo District, Shanghai	Post Code	200333
Telephone	021-22265200	Fax	021-63403027

Contract Research Organization (2)

Company	Wuxi Clinical		
Address	701, Block A, SOHO Fuxing Plaza, No. 299 Danshui Road, Jingan District, Shanghai, China	Post Code	200025
Telephone	021-23158000	Fax	021-53061029

Data Management

Company	dMed Biopharmaceutical Company Ltd.		
Address	780 Cailun Rd, 6/F, Q, Zhangjiang Hi-Tech Park, Pudong, Shanghai	Post Code	201203
Telephone	021-50900085	Fax	021-68755155

Safety and Pharmacodynamic Analysis

Company	dMed Biopharmaceutical Company Ltd.		
Address	780 Cailun Rd, 6/F, Q, Zhangjiang Hi-Tech Park, Pudong, Shanghai	Post Code	201203
Telephone	021-50900085	Fax	021-68755155

Pharmacokinetic Analysis

Company	Clinical pharmacological Research Center, Chinese Academy of Medical Science, Peking Union Medical College Hospital		
Address	41, Damucang Lane, West District., Beijing	Post Code	100032
Telephone	010-69158364	Fax	010-69158364

Central Laboratory for Safety and Pharmacodynamic Analysis

Company	Covance Pharmaceutical Research and Development (Shanghai) Co., Ltd.		
Address	1/F, 6 Building, 151, Libing Rd, Shanghai	Post Code	201203
Telephone	021-51371111	Fax	021-51371301

Central Laboratory for Analysis of Pharmacokinetic Samples

Company	Wuxi AppTec		
Address	288 Fute Zhong Road, Pudong New District, Shanghai	Post Code	200131
Telephone	021-50464102	Fax	021-50461000

Electronic System Vendor

Company	Medidata Information Technology (Shanghai) Co., Ltd.		
Address	9/F 1788 West Nanjing Rd, Jing'an District., Shanghai	Post Code	200040
Telephone	021-22310417	Fax	N/A

Protocol Signature Page

I agree:

- To conduct this study strictly following the protocol, Good Clinical Practice (GCP) and applicable regulatory requirements.
- To preserve all information provided by Hua Medicine (Shanghai) Ltd. in accordance with confidentiality statement. All information should also be marked as confidential when submitted to ethics committee (EC).

I have read the entire protocol and agreed with all content.

____________________________ ______________________ ________________

Sponsor Representative Name Signature Date (Year-Month-Day)

Protocol Signature Page

I have read the protocol (version 1.1) entitled as "A 24-week multi-center, randomized, double-blind, placebo-controlled, phase III study to evaluate the efficacy and safety of HMS5552 add-on to metformin with additional 28-week open-label treatment to evaluate the safety of HMS5552 add-on to metformin in type 2 adult diabetes mellitus subjects". I agree to conduct this study in accordance with the protocol.

I agree to follow the guidance of GCP and other regulations/guidelines applicable to China Food and Drug Administration (CFDA). I declare that I will be responsible for overall conduct of this study. I also agree to ensure that all assistants and colleagues involved in this study are aware of their obligations.

I agree and confirm that without prior written approval from Hua Medicine (Shanghai) Ltd, I will not use the confidential information in this protocol for purposes other than study evaluation or implementation.

I agree that the electronic signature which is in accordance with national safety standards and regulations is legally equivalent to wet signature.

Principal Investigator Name:____________________

Signature:____________________________ Date (Year-Month-Day):_________________

Study Protocol Synopsis

**Protocol Number**	HMM0302
**Study Title**	A 24-week multi-center, randomized, double-blind, placebo-controlled, phase III study to evaluate the efficacy and safety of HMS5552 add-on to metformin with additional 28-week open-label treatment to evaluate the safety of HMS5552 add-on to metformin in type 2 adult diabetes mellitus subjects
**Study Objective**	To evaluate the efficacy and safety of HMS5552 add-on to metformin after 24-week treatment, and the safety after 52-week treatment in subjects with type 2 diabetes mellitus (T2DM) whose blood glucose is still inadequately controlled under the stable metformin treatment
**Study Design**	This study is a phase III study in subjects with T2DM whose blood glucose is still insufficiently controlled under the stable metformin treatment.<br>The overall study design is as follows. After screening, eligible subjects enter a 4-week, single-blind, placebo run-in period on the basis of diet and exercise interventions. Meanwhile, subjects are treated with metformin (Glucophage) at 1500mg/day as basic therapy during the run-in period. At the 3rd week of run-in period, the randomization eligibility is confirmed by laboratory tests at Day -5(±2 days) before randomization. Eligible subjects are randomized to HMS5552 75mg BID group or placebo group at a ratio of 1:1 at randomization visit after the eligibility is confirmed. And then they enter a 24-week double-blind treatment period during which metformin (1500 mg/day) should be maintained in both groups as basic therapy, followed by a 28-week open-label period with HMS5552 75mg BID add-on to metformin (1500 mg/day) treatment. After the 52-week treatment, all study medications should be stopped and subjects are observed for 1 week for safety evaluation. The dosage of metformin (Glucophage) should be maintained at 1500 mg/day from beginning of the run-in period to completion of open-label treatment.
**Main Inclusion/Exclusion Criteria**	**Inclusion criteria**<br>1. Male or female, aged 18–75 years old (inclusive) when informed consent is signed.<br>2. Subjects diagnosed with T2DM according to the World Health Organization diagnostic criteria (1999) at screening and treated with metformin≥ 1500mg/day constantly for at least 12 consecutive weeks in addition to dietary and exercises intervention.

	3. Glycosylated hemoglobin (HbA1c) ≥ 7.5% and ≤ 10.0% at screening.<br>4. Body mass index (BMI)> 18.5 kg/m^2 and < 35.0 kg/m^2 at screening.<br>5. Willing to follow the same diet and exercise interventions throughout the study, to take medications and meals on time per study protocol requirements, and to conduct self-monitoring of blood glucose (SMBG) timely and keep records.<br>6. Willing to sign the written informed consent form and comply with the study protocol.<br>**Exclusion Criteria**<br>Subjects cannot be enrolled if any of the following criteria is met (all laboratory parameters are examined in the central laboratory).<br>**Exceptions for Target Disease**<br>1. Any antidiabetic therapy other than metformin within 12 weeks before screening.<br>2. Received insulin therapy for more than 30 days within 1 year before screening.<br>3. History of severe hypoglycemia (requiring external assistance for recovery) or recurrent hypoglycemia (such as experience of 3 or more times of hypoglycemia <=3.9 mmol/L or hypoglycemia symptoms within 1 month) without contributing factors within 3 months before screening.<br>4. Fasting C-peptide <0.81ng/ml (0.27 nmol/L) at screening.<br>5. Medical history of diabetic ketoacidosis, diabetes lactic acidosis or hyperosmotic nonketotic diabetic coma.<br>6. Subjects clinically diagnosed with type 1 diabetes mellitus, or diabetes mellitus caused by pancreatic damage or other special types of diabetes.<br>**Medical History and Concomitant Disease**<br>7. Severe cardio-cerebrovascular diseases defined as:<br>a) Medical history of myocardial infarction, coronary angioplasty or coronary artery bypass grafting, valvular disease or repaired, clinically significant unstable arrhythmia, unstable angina, transient ischemic attack, or cerebrovascular accident within 6 months before screening.<br>b) Class III or IV congestive heart failure according to New York Heart Association (NYHA) Classification.<br>8. Unstable or rapidly progressive kidney diseases.

9. Active liver diseases at screening.

10. Diagnosed mental illness.

11. Hemoglobinopathies (such as sickle cell anemia, thalassemia, or sideroblastic anemia).

12. The immunocompromised subjects such as subjects who underwent organ transplantation or subjects diagnosed with human immunodeficiency virus infection (HIV).

13. Any type of malignant tumor (no matter cured or not).

14. Any endocrine system diseases related to blood glucose (such as hyperthyroidism, acromegaly, Cushing's syndrome) or immune diseases which are unstable and need medical interventions and decided by the Investigator as not being suitable for this study.

15. History of rug abuse.

16. Received corticosteroids treatment (except for short-term external use) within 1 year before screening.

17. Alcohol intake> 2 units a day, or > 14 units a week.

    One unit of alcohol equivalent to 150 mL of wine, 350 mL of beer, 100 mL (<=17%) or 80 mL (>17% and <=24%) of low-alcohol liquor, or 50 mL of high-alcohol liquor (>24%).

Physical Examination and Laboratory Results

18. Significant abnormal liver function at screening, defined as alanine aminotransferase (ALT)> 2.5×upper limit of normal (ULN), or aspartate aminotransferase (AST)> 2.5×ULN or serum total bilirubin (TBiL)> 1.5×ULN（refer to Appendix 6）.

19. Serological evidence of hepatitis virus infection at screening: positive hepatitis virus A IgM antibody (IgM anti-HAV), or positive hepatitis B surface antigen (HBsAg) and IgM antibody to hepatitis B core antigen (IgM anti-HBc) (to exclude subjects with acute hepatitis virus B infection), or positive hepatitis C virus antibody (anti-HCV).

20. Estimated glomerular filtration rate (eGFR)< 60 mL/min/1.73m^2 at screening.

21. Triglyceride> 5.7 mmol/L at screening.

	22. Anemia of any causes, which is defined as: hemoglobin < 12.0 g/dL (120 g/L) for male and hemoglobin < 11.0 g/dL (110 g/L) for female at screening.<br><br>23. Abnormal laboratory results that may interfere with the safety evaluation, as judged by the Investigator.<br><br>24. Abnormal electrocardiogram (ECG) results that may affect the safety evaluation or need medical interventions, as judged by the Investigator（except for stable coronary heart disease）.<br><br>25. Systolic blood pressure ≥ 160 mmHg or diastolic blood pressure ≥ 100 mmHg at screening or who added/changed antihypertensive drugs or adjusted dose within 4 weeks before screening.<br><br>**Allergies and Adverse Drug Reactions**<br><br>26. Subjects who are potentially allergic or do not tolerate study medications, as considered by the Investigator.<br><br>**Prohibited Therapies and/or Medications**<br><br>27. Subjects who refuse to only take the antidiabetic medications specified by the study protocol.<br><br>28. Concomitant treatment with potent or moderate CYP3A4 inducers or inhibitors (see Section 4.4.2).<br><br>**Sex and Reproductive Status**<br><br>29. Women who are pregnant or intend to be pregnant during the study.<br><br>30. Women in breastfeeding.<br><br>31. Women with childbearing potential and refuse to take high-efficient or clinically accredited contraceptive methods throughout the study and within 1 month after the last dose of study medications.<br><br>**Other Exclusion Criteria**<br><br>1. Any disease or medical condition that may prevent the subjects from completing all study procedures considered by the Investigator.<br><br>2. Subjects who need to receive some other treatments that may potentially affect the interpretation of efficacy and safety data of the sutdy, as decided by the Investigator.<br><br>3. Based on Investigator's judgment, Subjects who are unable to follow the study protocol, e.g., unable to persist on dietary and exercise therapy during the study,

	to take medications and meals timely per study protocol, and to conduct self-monitoring of blood glucose (SMBG) and make record timely etc.<br><br>4. Subjects who participated in any clinical trials or took any study drug within 30 days before screening.<br><br>**Randomization Criteria (laboratory tests for randomization and 12-lead ECG are conducted on Day -5 [± 2 days])**<br><br>At Visit 4 (randomization visit), the following criteria will be re-evaluated to confirm eligibility. Subjects who meet the following randomization criteria are eligible to be randomized.<br><br>**Inclusion Criteria**<br><br>1. HbA1c ≥ 7.5% and ≤ 10.0% at pre-randomization visit.<br><br>2. Fasting blood glucose (FPG) > 7.0 mmol/L and < 13.3 mmol/L at pre-randomization visit.<br><br>**Exclusion Criteria**<br><br>3. History of severe hypoglycemia (requiring external assistance for recovery) or recurrent hypoglycemia (such as experience of 3 or more times of hypoglycemia <=3.9 mmol/L or hypoglycemia symptoms within 1 month) without contributing factors within 3 months in run-in period.<br><br>4. Body weight change (weight gain or loss) ≥ 10% at randomization compared with that at screening.<br><br>5. Poor adherence to treatment during run-in period (< 80% or > 120%).<br><br>6. Abnormal liver function with clinical significance occurred in run-in period, defined as ALT > 2.5×ULN, or AST > 2.5×ULN or TBiL > 1.5×ULN at pre-randomization visit (refer to Appendix 6).<br><br>7. eGFR< 60mL/min/1.73m^2 at pre-randomization visit.<br><br>8. Triglyceride> 5.7mmol/L at pre-randomization visit.<br><br>9. Anemia of any causes, defined as hemoglobin < 12.0 g/dL (120 g/L) for male, and hemoglobin < 11.0 g/dL (110 g/L) for female at pre-randomization visit.<br><br>10. Systolic blood pressure ⩾ 160 mmHg or diastolic blood pressure ⩾ 100 mmHg at randomization visit, or who added/changed the antihypertensive drugs or adjusted dose within run-in period.<br><br>11. Abnormal laboratory results that may affect the safety evaluation of the study, as

	judged by the Investigator.<br>12. Abnormal ECG results that may affect the safety evaluation of the study or need medical interventions, as judged by the Investigator (except for stable coronary heart disease).<br>13. The Investigator reconfirm the medical history and concurrent disease occurred between screening and randomization visits (refer to the exclusion criteria at screening visit).
**Study Hypothesis**	**The HbA1c reduction after 24-week treatment with HMS5552 75 mg BID combined with metformin is significantly superior to that with placebo combined with metformin in subjects with T2DM inadequately controlled with stable treatment of metformin.**
**Efficacy Endpoints**	**Primary Efficacy Endpoint**<br>In subjects with T2DM inadequately controlled with stable treatment of metformin, to compare the change of HbA1c from baseline after HMS5552 75 mg BID combined with metformin treatment at 1500 mg/day with placebo BID combined with metformin treatment at 1500 mg/day after 24-week double-blind treatment.<br>**Secondary Efficacy Endpoints**<br>To compare HMS5552 75 mg BID combined with metformin at 1500 mg/day with placebo BID combined with metformin at 1500 mg/day after 24-week double-blind treatment in subjects with T2DM inadequately controlled with stable treatment of metformin:<br>1. Change of 2-hour postprandial plasma glucose (2h-PPG) from baseline<br>2. Change of FPG from baseline<br>3. HbA1c response rate: the proportion of subjects whose HbA1c < 7.0%<br>4. Change of HbA1c from baseline at each visit, except Week 24.
**Safety Endpoints**	• Adverse events throughout the study<br>• Hypoglycemic events<br>• Physical examination<br>• Vital signs

	• 12-lead ECG<br>• Clinical laboratory examinations (hematology, biochemistry and urinalysis)
**Investigational Products**	**Basic drug**<br>Metformin (Glucophage). 1500 mg/day, taken according to doctor's advice. The administration method is kept consistent throughout the study.<br>**Study drug**<br>HMS5552 tablets, 75 mg BID, one tablet each time, prefer to take before meals.<br>**Control drug**<br>Placebo tablets, BID, one tablet each time, prefer to take before meals.
**Usage and dosage of Study Medications**	**Run-in period**<br>Basic drug + control drug, continue to take for 4 weeks<br>**Double-blind treatment period**<br>Basic drug + study drug or control drug, continue to take for 24 weeks<br>**Open-label treatment period**<br>Basic drug + study drug, continue to take for 28 weeks<br>**Safety follow-up period**<br>None
**Statistical Considerations**	**Sample size**<br>1. This study plans to recruit 750 subjects. Considering 20% drop-out rate, no more than 938 subjects will be recruited. The subjects are randomized at a ratio of 1:1 to treatment group (HMS5552 75 mg BID + metformin 1500 mg/day) and control group (placebo BID + metformin 1500 mg/day), i.e. no more than 469 subjects and 469 subjects are randomized to treatment group and control group.<br>2. Assessment: Based on the planned sample size and the randomization ratio (750 subjects, treatment group: control group = 1:1), the study has 95.4% power to detect the efficacy in treatment group is superior to the control group with the assumption of type I error (alpha) at 0.05 (two sided), if the within-group standard deviation for primary efficacy endpoint (change of HbA1c from baseline after

	24-week treatment) is 1.5% and the between-group difference (treatment group-control group) is -0.4%.<br><br>**Statistical analysis**<br><br>The primary endpoint of this study is the change of HbA1c from baseline to double-blind treatment completion (Week 24). Comparison between treatment and control group will be made at a significant level of 0.05 (two-sided). The mixed model with repeated measurements is mainly used for this primary endpoint analysis. Subjects included in the analysis should have baseline measurement of the primary efficacy endpoint and at least one post treatment measurement during double-blind treatment period. The factors of the model include groups, visits, the interaction between groups and visit, sites, disease duration and baseline HbA1c value. An unstructured covariance structure is preferred for the primary analysis. The Statistical Analysis Plan prepared separately provides the details for other covariance structures in case the preferred covariance model does not converge. The primary analysis provides point estimation and 95% confidence interval for the difference in the change of HbA1c from baseline between groups (treatment group-control group).
**Population Pharmacokinetic/ Pharmacodynamic Analysis (PopPK/PD)**	The PopPK/PD model for HMS5552 is updated by using the NONMEM/PIRANA/R software based on the current HMS5552 PopPK/PD model and PK/PD data in Chinese subjects with T2DM after HMS5552 treatment. The quantitative relationship between HMS5552 concentration and HbA1c level is established. Systemic quantitative analysis is conducted on the influences of various factors on HMS5552 PK/PD characteristics, e.g., demographics, T2DM duration, concomitant medications etc..

Study Flow Chart

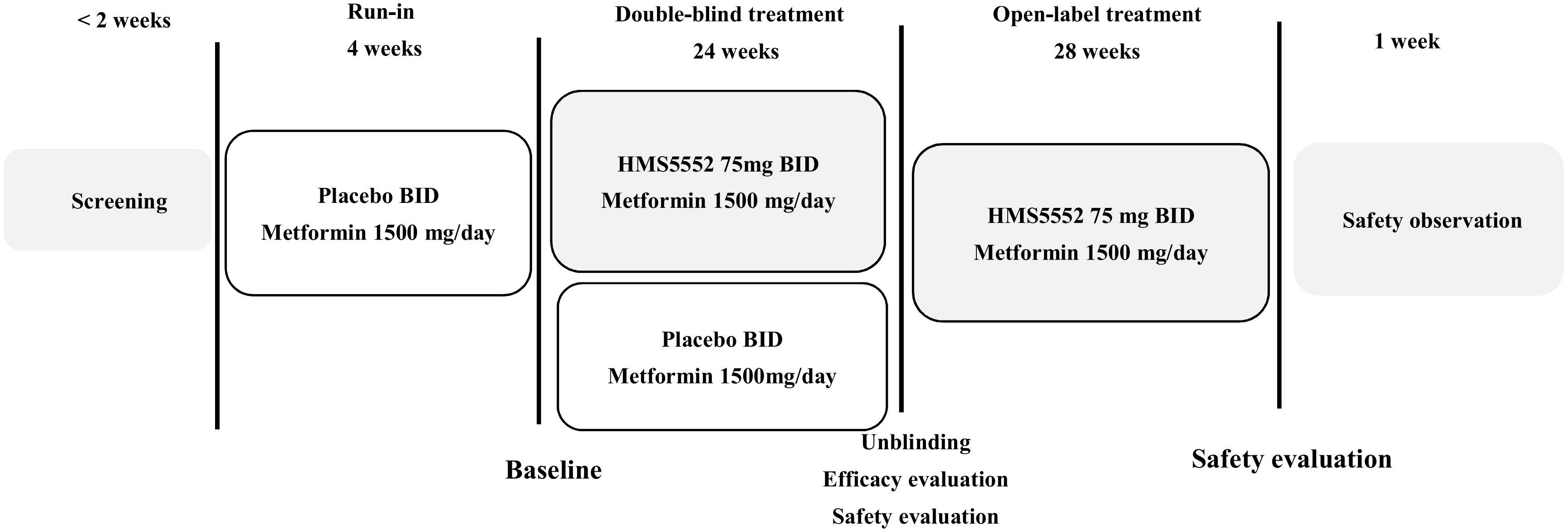

Schedule of Assessments

	Screening	Single blind run-in period[1]		Randomization	Double-blind treatment period						Open-label treatment period					Safety observation
Visit number	Visit 1	Visit 2	Visit 3	Visit 4	Visit 5	Visit 6	Visit 7	Visit 8	Visit 9	Visit 10[2]	Visit 11	Visit 12	Visit 13	Visit 14	Visit 15[3]	Visit 16[4]
Visit time	Week -6 to -4	Week -4±3 days	Day-5±2 days	Day 1	Week 4 ± 4 days	Week 8 ± 4 days	Week 12 ± 4 days	Week 16 ± 4 days	Week 20 ± 4 days	Week 24 ± 4 days	Week 28 ± 4 days	Week 34 ± 6 days	Week 40 ± 6 days	Week 46 ± 6 days	Week 52 ± 6 days	Week 53 ± 2 days
**Study Procedure**																
Informed consent	X															
Demographics	X															
Medical history and prior medications	X			X												
Inclusion and exclusion criteria	X			X												
Concomitant medications	X	X	X	X	X	X	X	X	X	X	X	X	X	X	X	X
Randomization				X												
Adverse events		X	X	X	X	X	X	X	X	X	X	X	X	X	X	X
Physical examination[5]	X			X	X	X	X	X	X	X	X	X	X	X	X	
Vital signs [6]	X			X	X	X	X	X	X	X	X	X	X	X	X	
12-Lead ECG	X		X	X	X	X	X	X	X	X	X	X	X	X	X	
**Laboratory Tests (in central laboratory)**																
HbA1c	X		X	X	X	X	X	X	X	X	X	X	X	X	X	
FPG	X		X	X	X	X	X	X	X	X	X	X	X	X	X	
Hematology[7]	X		X	X	X	X	X	X	X	X	X	X	X	X	X	
Urinalysis[8]	X		X	X	X	X	X	X	X	X	X	X	X	X	X	
Blood biochemistry[9]	X		X	X	X	X	X	X	X	X	X	X	X	X	X	
Virology test[10]	X															
Pregnant test[11]	X	X	X	X	X	X	X	X	X	X	X	X	X	X	X	
MMTT[12]				X			X			X					X	
Biomarker[13]	X			X			X			X					X	
PK sampling[14]					X		X			X					X	
DNA sample[15]				X												
**Instructions / Suggestions**																
Health education[16]	X	X	X	X	X	X	X	X	X	X	X	X	X	X	X	
Glucometer distribution		X														
SMBG check[17]			X	X	X	X	X	X	X	X	X	X	X	X	X	
Subject diary		X		X	X	X	X	X	X	X	X	X	X	X		
**Study Medication**																
Drug distribution		X		X	X	X	X	X	X	X	X	X	X	X		
Review of drug usage and recycling[18]				X	X	X	X	X	X	X	X	X	X	X	X	

ECG: electrocardiogram. FPG: fasting plasma glucose. HbA1c: glycosylated hemoglobin. MMTT: mixed-meal tolerance test. SMBG: self-monitoring of blood glucose.

Note: a. All laboratory sampling must be collected under fasting before dosing (except for MMTT 30-min and 120-min), suggested time: 7:00 to 10:00 a.m. b. MMTT is suggested to start between 7:00 and 9:00 a.m. c. The schedule of visit window is based on normal working time. In case of the National Day, Spring Festival and other holidays, the Investigator can arrange for administration of the study medications accordingly and reschedule the next visit to a time closest to the visit scheduled per study protocol. Causes for early/late visit will be recorded on eCRF and although it is not considered as protocol deviation in this particular case.

1. Single-blind placebo run-in period lasts from Day -28±3 days to Day -1 (inclusive).
2. The double-blind treatment period (Visit 10) ends when subjects complete all the 24-week treatment. If early withdrawal occurs in the double-blind treatment period, a termination visit needs to be conducted for the patient within 7 days (the 7th day included) after the last dose of study medications. In addition, a telephone follow-up for safety is conducted within 1 week after the last dose of study medications. If administration of the study medications has been stopped by the subjects for ≥ 7 days before early withdrawal, a termination visit needs to be completed as much as possible. The tests in the termination visit are the same as Visit 15.
3. The open-label treatment period (Visit 15) ends when subjects finish all the 52-week treatment. If early withdrawal occurs in the open-label treatment period, a termination visit needs to be completed within 7 days (the seventh day included) after the last dose of study medications. In addition, a telephone follow-up for safety is conducted within 1 week after the last dose of study medications. If administration of the study medications has been stopped by the subjects for ≥ 7 days before early withdrawal, a termination visit needs to be completed as much as possible. The tests in the termination visit are the same as Visit 15. If the study medications are already stopped at the termination visit, the MMTT can be cancelled.
4. All study medications, including metformin, are stopped in the safety observation period. Visit 16 is conducted by telephone at which adverse events and concomitant medications occurring in the safety observation period are recorded. If there is any adverse event that has occurred during the treatment period and is still ongoing, the investigator should arrange the follow-up visit based on the need of adverse event during the observation period and carry out corresponding examinations.
5. Physical examinations: general appearance, head and neck, chest (including heart and lungs), abdomen, limbs, etc.
6. Vital signs: for Visit 1, blood pressure, pulse rate, breath rate, body temperature (axillary temperature), height and weight. For other visits, all parameters except height are checked.
7. Hematology: hemoglobin, hematocrit, erythrocytes count, leukocytes count, neutrophils, basophils, eosinophils, monocytes, lymphocytes, and platelet count.
8. Urinalysis: pH, specific gravity, protein, glucose, erythrocytes, leukocytes, and ketone bodies.
9. Blood biochemistry: plasma glucose, total bilirubin, alanine aminotransferase, aspartate aminotransferase, gamma-glutamic acid, alkaline phosphatase, albumin, total protein, lactate dehydrogenase, urea nitrogen, creatinine, uric acid, sodium, potassium, chlorine, calcium, creatine kinase, creatine kinase isoenzyme, total cholesterol, high-density lipoprotein cholesterol, low-density lipoprotein cholesterol, and triglyceride.
10. Virology test: hepatitis A virus IgM antibody, hepatitis B surface antigen (HBsAg), hepatitis B surface antibody (anti-HBs), IgM antibody to hepatitis B core antigen (IgM anti-HBc), hepatitis C virus antibody (anti-HCV), human immunodeficiency virus (HIV) antibodies.

11. Pregnancy test (only for females with childbearing potential): From Visit 1 to Visit 15, blood pregnancy test must be performed in the central laboratory. During the study, if any amenorrhea or irregular menstruation is reported for any female subject at any time, a blood pregnancy test should be performed in the central laboratory immediately to exclude or validate pregnancy. Drug administration is stopped immediately once a positive result is shown.
12. MMTT: The blood samples for evaluating plasma glucose, insulin and C-peptide are collected 30 minutes (±3 minutes) and 120 minutes (±5 minutes) after the instant noodles standard meal being taken, which is provided by the Sponsor. The standard meal should be taken before drug administration at Visit 4. At Visits 7, 10 and 15, the standard meal is taken after drug administration. (blood sampling at 30 minutes after the standard meal is applicable to subjects enrolled after the protocol v1.1 taking effect).
13. Biomarker: including fasting insulin and C-peptide. At Visit 1, only C-peptide is tested and evaluated as one of the exclusion criteria.
14. Population pharmacokinetics: At Visit 5, blood samples are collected before drug administration. At Visits 7, 10 and 15, blood sampling schedule is the same as MMTT. The time of blood sampling and the time (before the scheduled visit) for the closest investigational drug (HMS5552/Placebo) administration before blood sampling are recorded in detail.
15. DNA sampling. At Visit 4, DNA samples are collected (optional).
16. Diabetes education and health/exercise guidance: The Investigator fully assesses the life and disease status for each subject. If any inappropriate lifestyle is identified (e.g. eating or drinking too much in holidays) for any subject, the diabetes education and health/exercise guidance should be given to him/her timely and repeatedly.
17. SMBG examination: For each subject, fingertip blood glucose under fasting or postprandial is required to be monitored at least twice a week at home. If suspicious hypoglycemia or hyperglycemia events occur, additional blood glucose tests should be conducted and the results are recorded in subject diaries. At each visit, the Investigator should review the subject diaries carefully then make suggestions on drug administration and blood glucose monitoring for next stage.
18. Review of drug usage and recycling: The Investigator should review the drug compliance record from Visit 2 to Visit 4 (run-in period) and from Visit 4 to Visit 15 (treatment period). Queries should be made if any problem is found. At Visit 4, drug compliance is evaluated for the run-in period. Guidance should be given if any subject does not follow the drug administration specified in the study protocol.

Contents

Protocol Signature Page **4**
Protocol Signature Page **5**
Study Protocol Synopsis **6**
Study Flow Chart **14**
List of Abbreviations **22**
1 Background **24**
1.1 INTRODUCTION OF DISEASE 24
1.2 DRUG BACKGROUND 24
1.3 REFERENCES FOR THE PRECLINICAL STUDIES AND PRELIMINARY CLINICAL STUDIES OF HMS5552 ON TYPE 2 DIABETES MELLITUS 26
1.4 THE RATIONALE OF THE STUDY 27
1.5 DOSE SELECTION, BENEFIT/RISK ASSESSMENT, AND ETHICAL REVIEW 27
2 Study Objectives **28**
2.1 THE PRIMARY OBJECTIVE 28
2.2 THE SECONDARY OBJECTIVES 28
3 Study Design **28**
3.1 STUDY ENDPOINTS 28
3.1.1 Primary Efficacy Endpoint *28*
3.1.2 Secondary Efficacy Endpoints *28*
3.1.3 Safety Assessment *29*
3.2 STUDY DESIGN AND RATIONALE 29
3.3 SUBJECT ENROLLMENT, RANDOMIZATION, AND TREATMENT 30
3.3.1 Randomization Method *30*
3.3.2 Run-in Period *30*
3.3.3 Double-Blind Treatment Period *31*
3.3.4 Procedures for incorrect enrollment, randomization, or drug administration *32*
3.3.5 Blinding *32*
3.4 PREPARATION FOR THE STUDY 32
3.5 STUDY PROCEDURES AND STAGES 33
3.5.1 Visit 1: Screening (Day -42 to -29) *33*
3.5.2 Visit 2: Single-Blind Placebo Run-in Period (Day -28±3 days) *34*
3.5.3 Visit 3: Pre-randomization Visit (Day -5 ± 2) *35*
3.5.4 Visit 4: Randomization Visit (Day 1) *35*
3.5.5 Visit 5: Treatment Visit (Day 28 ± 4) *37*
3.5.6 Visit 6: Treatment Visit (Day 56 ± 4) *38*
3.5.7 Visit 7: Treatment Visit (Day 84 ± 4) *39*
3.5.8 Visit 8: Treatment Visit (Day 112 ± 4) *40*
3.5.9 Visit 9: Treatment Visit (Day 140 ± 4) *40*
3.5.10 Visit 10: Termination Visit for Double-blind Treatment (Day 168 ± 4) *40*
3.5.11 Visit 11: Open-Label Treatment Visit (Day 196 ± 4) *41*

3.5.12 Visit 12: Open-Label Treatment Visit (Day 238 ± 6) *41*
3.5.13 Visit 13: Open-Label Treatment Visit (Day 280 ± 6) *42*
3.5.14 Visit 14: Open-Label Treatment Visit (Day 322 ± 6) *42*
3.5.15 Visit 15: Termination Visit for End of Open-Label Treatment (Day 364 ± 6) *42*
3.5.16 Visit 16: Follow-up Visit for Safety (Day 371 ± 2) *43*
3.6 STUDY MATERIALS PROVIDED TO SUBJECTS 43
4 Study Population 44
4.1 INCLUSION CRITERIA 44
4.2 EXCLUSION CRITERIA 44
4.3 RANDOMIZATION CRITERIA (LABORATORY TESTS AND 12-LEAD ECG WILL BE CONDUCTED ON DAY -5 [± 2 DAYS] PRIOR TO THE RANDOMIZATION) 47
4.4 CONCOMITANT MEDICATION AND POST-STUDY TREATMENT 48
4.4.1 General Instructions on Study Treatment *48*
4.4.2 Prohibited and Restricted Medications *48*
4.4.3 Contraception *49*
4.4.4 Diet Control and Exercise Intervention *49*
4.4.5 Post-Study Treatment *50*
4.5 DISCONTINUATION OF STUDY TREATMENT, WITHDRAWAL, OR TERMINATION OF STUDY 50
4.5.1 Withdrawal Due to Hypoglycemia *51*
4.5.2 Withdrawal due to Hyperglycemia *52*
4.5.3 Withdrawal due to Abnormal Liver Function *53*
4.6 PROCEDURES FOR SUBJECT WITHDRAWAL 53
4.7 LOST TO FOLLOW-UP 54
5 Study Drugs 54
5.1 STUDY MEDICATIONS 54
5.1.1 Drug Name, Strength, Formulation *54*
5.1.2 Packaging and Labelling *54*
5.1.3 Study Medication Regimen and Packaging *56*
5.2 STORAGE CONDITION 57
5.3 DRUG DISPENSING AND COUNTING 57
5.4 DRUG RETURN AND DESTRUCTION 58
5.5 DRUG COMPLIANCE 58
5.6 EXTENT OF EXPOSURE 59
6 Study Endpoints 59
7 Safety Assessments 59
7.1 ADVERSE EVENT (AE) 60
7.1.1 Serious Adverse Event (SAE) *60*
7.1.2 Persistent or Recurrent AEs *61*
7.1.3 AEs Related to Examinations and Laboratory Tests *61*
7.2 RECORDING OF AEs 61
7.2.1 Terms of AEs *61*
7.2.2 Recording of AE Time and Collection Period for AE *62*
7.2.3 AE Variables *62*

7.2.4 AE Grading ... *63*
7.2.5 AE Outcome ... *63*
7.2.6 Causality Assessment ... *63*
7.3 SERIOUS ADVERSE EVENT REPORTING ... 64
7.4 HYPOGLYCEMIA ... 64
7.5 HY'S LAW ... 65
7.6 POST-STUDY AE REPORTING ... 65
7.7 LACK OF EFFICACY OR AGGRAVATION OF T2DM ... 65
7.8 LABORATORY SAFETY ASSESSMENTS ... 66
7.9 PHYSICAL EXAMINATIONS ... 66
7.10 12-LEAD ECG ... 67
7.11 VITAL SIGNS ... 67
7.12 PREGNANCY ... 68
7.13 OVERDOSE ... 68
8 Data Management ... **69**
8.1 DATA TRACEABILITY, COMPLETION, AND TRANSFER OF ECRF ... 69
8.2 DATABASE DESIGN ... 69
8.3 DATA ENTRY ... 69
8.4 DATA VALIDATION ... 69
8.5 BLINDED DATA REVIEW AND UNBLINDING ... 69
8.6 DATABASE LOCK ... 70
9 Statistical Analysis ... **70**
9.1 SAMPLE SIZE DETERMINATION ... 70
9.2 ANALYSIS POPULATION SETS ... 71
9.3 STATISTICAL METHODS ... 71
9.3.1 Demographics and Baseline Characteristics ... *71*
9.3.2 Efficacy Analysis for Primary Endpoint ... *72*
9.3.3 Efficacy Analysis for Secondary Endpoints ... *72*
9.3.4 Safety Analysis ... *73*
9.3.5 Biomarker Analysis ... *73*
9.3.6 Sensitivity Analysis ... *73*
9.3.7 Interim Analysis ... *74*
10 Population PK/PD （PopPK/PD）Analysis ... **74**
11 Trial Management ... **74**
11.1 DECLARATION ... 74
11.2 ETHICS COMMITTEE ... 74
11.3 SOURCE DOCUMENT VERIFICATION ... 75
11.4 QUALITY CONTROL AND QUALITY ASSURANCE ... 75
11.5 SUBJECT INFORMED CONSENT ... 75
11.6 PROTOCOL AMENDMENT ... 76
11.7 MONITORING ... 76
11.8 CONFIDENTIALITY AGREEMENT AND PATIENT PRIVACY ... 76
12 Publication ... **77**

13 Documents Archiving **77**
14 Appendix **78**
15 References **92**

List of Abbreviations

ADA	American Diabetes Association
AE	adverse event
ALP	alkaline phosphatase
ALT	alanine aminotransferase
AST	aspartate aminotransferase
BID	twice a day
BMI	body mass index: weight[kg]/ (height [m])^2
BUN	blood urea nitrogen
CDISC	Clinical Data Interchange Standards Consortium
CFDA	China Food and Drug Administration
CRO	Contract Research Organization
DPP-4	dipeptidyl peptidase-4
EASD	European Association for Diabetes Study
EC	ethics committee
ECG	electrocardiography
EDC	electronic data capture
e.g.	for example
eCRF	electronic case report form
eGFR	estimated glomerular filtration rate
FAS	full analysis set
FDA	Food and Drug Administration
FPG	fasting plasma glucose
GCP	Good Clinical Practice
GK	glucokinase
GKA	glucokinase activator
GLP-1	glucagon-like peptide-1
GMP	Good Manufacturing Practices
G-6-P	glucose-6-phosphate
HbA1c	glycosylated hemoglobin
HIV	human immunodeficiency virus
HK	hexokinase
IB	investigator's brochure
ICF	Informed Consent Form
ICH	International Conference on Harmonization
IWRS	interactive web response service

i.e.	that is to say
ISF	Investigator Site File
kg	kilogram
LDH	lactate dehydrogenase
LOCF	last observation carried forward
NYHA	New York Heart Association
MAD	multiple ascending dose
MedDRA	Medical Dictionary for Regulatory Activities
mmHg	millimeter of mercury
MMRM	mixed-effect model repeated measure
MMTT	mixed-meal tolerance test
MODY-2	maturity-onset diabetes of the young type-2
nH	Hill coefficient
OR	odds ratio
PD	pharmacodynamics
pH	potential of hydrogen
PK	pharmacokinetics
PPG	post-prandial plasma glucose
PPS	per protocol set
QA	quality assurance
QC	quality control
QD	once a day
SAD	single ascending dose
SAE	serious adverse event
SMBG	self-monitoring of blood glucose
SS	safety set
SOP	Standard Operation Procedure
TBiL	total bilirubin
TG	triglyceride
T2DM	type 2 diabetes mellitus
UKPDS	United Kingdom Prospective Diabetes Study

1 Background

1.1 Introduction of Disease

Diabetes mellitus is a chronic metabolic disease characterized by hyperglycemia, and requires continuous medical treatments. Diabetes mellitus usually has the concomitant diseases of ischemic heart disease, cerebrovascular and peripheral vascular diseases, which are major causes of early death and high rate of disability, as well as the common causes for blindness and renal failure.

Type 2 diabetes mellitus (T2DM) accounts for approximate 90% to 95% of all diagnosed diabetes cases and is characterized by hyperglycemia caused by decreased insulin secretion, increased insulin resistance and hepatic glucose output [1]. With the rapid growth of China's economy, the incidence of diabetes has also increased. A national epidemiological survey in 2008 showed that the prevalence of diabetes was 9.7%. Two years later, another investigational result showed that the prevalence of diabetes in adults above 18 years old increased to 11.6%, with an absolute number of 114 million. China has become the country with the largest number of patients with diabetes mellitus [2].

Usually, diet control and physical exercise are the major strategies for treating slightly elevated blood glucose in patients with T2DM. If diet control and physical exercise are still insufficient to lower blood glucose, oral antidiabetic drugs should be given. Various antidiabetic drugs have been approved for the treatment of T2DM subjects, including metformin, sulfonylureas, alpha-glucosidase inhibitors, dipeptidyl peptidase 4 (DDP-4) inhibitors, glucose-like peptide-1 (GLP-1) receptor agonists and insulin, etc. These drugs exert their effects by promoting insulin secretion, improving insulin sensitivity of peripheral tissue or other mechanisms [3].

Metformin is currently the first-line therapy listed in many international guidelines and national clinical guidelines[2][3]. However, monotherapy of many antidiabetic drug, including metformin, usually fails to achieve the target level of glycosylated hemoglobin (HbA1c). After starting a monotherapy, nearly 50% of diabetic subjects need to take a second antidiabetic drug. There is evidence that combination therapy using antidiabetic drugs with different mechanisms of action can improve glycemic control. Therefore, it is clinically urgent to develop new antidiabetic drugs that have different mechanisms of action that can be used as monotherapy or in combination with another antidiabetic drug.

1.2 Drug Background

Glucokinase (GK) is one of the four subtypes of hexokinase (HK) and catalyzes at the first step of glucose metabolism, by which glucose is phosphorylated and converted to 6-phosphate glucose (G-6-P) in glucose metabolism. The GK is mainly expressed in glucose-sensitive and metabolic active tissues including the pancreas, liver, intestine and brain. As a glucose sensor, the GK plays a key role in the glucose homeostasis in the body. Abnormal GK activity leads to glucose metabolism disorder, causing diabetes and related complications. Mutations in GK gene

can cause severe symptoms of diabetes. For example, mutations of function loss lead to adolescent onset of adult type 2 diabetes (MODY-2) and permanent neonatal diabetes mellitus. In contrast, gain of function or gene activation mutations cause hypoglycemia and hyperinsulinemia. In addition, decrease in GK activity exists in diabetic patients. For instance, further study on the mechanism of T2DM showed that decrease of pancreatic β cell function is associated with the change in GK quantity and activity. Decrease in liver GK activity may be one mechanism leading to insulin resistance[4].

In recent years, GK has become a significant target in the development of new drugs for diabetes because of its pivotal role in glucose homeostasis. The development of small molecule glucokinase activator (GKA) is very active as well[5].

Roche is the first global pharmaceutical company to bring GKAs into clinical studies[4] [5]. To date, three generations of GKAs (GK1, GK2 and GK3) have been in the clinical development stage, not only showing efficacy and safety in preclinical studies, but also showing clinical efficacy in patients with T2DM.

Based on the chemical structures of GKAs from previous three generations, that for the fourth generation of GKA RO5305552 (HMS5552) underwent relatively large optimization and the main characteristics are as follows:

1. RO5305552 exerts its multiple synergistic activity in a glucose-concentration dependent manner to decrease blood glucose level by improving pancreatic insulin secretion, regulating hepatic glucose transformation, and activating intestinal GK.

2. Activation of GK by RO5305552 reveals a significant synergistic feature in kinetics. By decreasing the $S_{0.5}$ value and increasing the V_{max} value, the affinity of GK with its substrate (glucose) is enhanced with minimum effect on the Hill coefficient (nH). Therefore, the risk of hypoglycemia in clinical is effectively decreased.

3. Compared with other GKAs, RO5305552 is advantageous for the low concentration of metabolites (all plasma steady-state concentration of metabolites in human are < 10%, and the categories of metabolites are similar to those in animals). Thus, for oral administration of RO5305552, the possibilities for toxic and side effects induced by its metabolites are low.

In summary, GKAs can positively influence several major etiological factors for patients with T2DM and are of great potential for drug development. As a GKA, RO5305552 has balanced safety and efficacy features and represents a potential and novel candidate for diabetes therapy[6].

Based on the reasons above, in 2011, Hua Medicine (Shanghai), Ltd. (hereinafter referred to as "Hua Medicine") imported the fourth generation GKA (RO5305552) from Roche. The identification number for RO5305552 in study is HMS5552, including both preclinical and clinical studies. It is expected that through the multiple activation effects of HMS5552, blood glucose control in patients with T2DM can be improved.

The HMS5552 project sponsored by Hua Medicine was approved by China Food and Drug Administration (CFDA) in 2013 (Clinical Trial Permission No.: 2013L01740-2013L01743, with 2013L01740 as a Notification of Approval Opinion). In 2015, Hua Medicine obtained supplemental Clinical Trial Permission (No.: 2015L01892-2015L01895). Currently, a total of four phase I clinical studies have been completed: single ascending dose (SAD) study in health volunteers (ClinicalTrials.gov ID: NCT01952535), multiple ascending dose (MAD) study in subjects with T2DM (ClinicalTrials.gov ID: NCT02077452), continuous 4-week treatment study in subjects with T2DM (ClinicalTrials.gov ID: NCT02386982) and drug-drug interaction between metformin and HMS5552 (USA) in subjects with T2DM (ClinicalTrials.gov ID: NCT02597400). In addition, a double-blind, placebo-controlled phase II clinical study of HMS5552 alone in subjects with T2DM (ClinicalTrials.gov ID: NCT02561338) was completed as well.

1.3 References for the Preclinical Studies and Preliminary Clinical Studies of HMS5552 on Type 2 Diabetes Mellitus

Please refer to the Investigator's Brochure (HMS5552 IB Version 3.0[6]) for more details on the preclinical and clinical studies of HMS5552.

So far, the preclinical and clinical studies for HMS5552 include: single-dose toxicity test (rats and dogs). repeated dose toxicity test (4-week and 13-week repeated dosing in both rats and dogs. 26-week repeated dosing in rats and 39-week repeated dosing in dogs). safety pharmacology test. fertility and early embryonic toxicity test, embryo-fetal developmental toxicity test. genetic toxicity test. preclinical study on pharmacokinetics (PK) and pharmacodynamics (PD) after single and multiple doses of HMS5552. Phase I clinical study on single ascending dose in 60 health volunteers[7][8]. Phase I clinical study of multiple ascending dose (MAD) in 53 subjects with T2DM[9]. Phase I clinical study of continuous 4-week treatment in 24 subjects with T2DM[10]. Phase I clinical study on the interaction between HMS5552 and metformin (USA) in 15 subjects with T2DM[11]. and a 12-week phase II study to evaluate the efficacy and safety of HMS5552 in 258 subjects with T2DM[12].

Studies have shown that the overall safety and tolerability were good in both male and female Chinese subjects after receiving HMS5552 25 mg to 200 mg, twice or once daily and by oral administration. All adverse events (AEs) reported were mild. No serious adverse events (SAEs) and severe hypoglycemia. HMS5552 exhibited dose linear PK characteristics in single dose, and also showed dose-dependent drug concentration increase after steady-state in multiple doses with no obvious accumulation and food effect, and with no sex difference. At steady state, the maximal metabolite in plasma was less than about 5% of original drug. Pharmacodynamics demonstrated the same dose-dependent decrease in fasting plasma glucose and postprandial plasma glucose after steady state in single and multiple doses. After multiple doses, the 24 hours plasma glucose decrease was associated with dosage. No obvious change of insulin and C-peptide level under fasting state was observed. Postprandial insulin and C-peptide secretion increased in a dose-dependent manner.

A phase II, multicenter, randomized, double-blind, placebo-controlled 12-week clinical study evaluated the safety, tolerability, efficacy, and population PK of HMS5552 in subjects with T2DM. The doses used in this study are 50 mg BID, 75 mg BID, 75 mg QD, and 100 mg QD. Data from the phase II study showed that, after treatment for 12 weeks, the subjects receiving 75 mg HMS5552 BID had a significant decrease (1.12%) of HbA1c compared with baseline.

1.4 The Rationale of the Study

At present, metformin is recommended as the first-line treatment for subjects with T2DM by both domestic and international guidelines. The main mechanisms of metformin to control plasma glucose include inhibiting hepatic glucose production and output, reducing glucose absorption, increasing glucose oxidation and metabolism in muscle and adipose tissue, and increasing the sensitivity of peripheral tissue to insulin. However, metformin cannot target all pathogenesis mechanisms of diabetes.

With the progression of diabetes, monotherapy is difficult to achieve good efficacy. In accordance with the results from the United Kingdom Prospective Diabetes Study (UKPDS), the monotherapy of sulfonylureas, insulin, metformin, or other traditional antidiabetic drug cannot maintain glycemic control targets in long-term.

The guidelines suggest the combination with other antidiabetic drugs if plasma glucose is controlled inadequately with metformin monotherapy at maximum dose or maximum tolerated dose[13][14][15][16].

Many studies show that combination of two drugs can bring greater benefits to diabetic patients. It’s mainly reflected by the fact that the combination therapy has better efficacy compared with monotherapy. The principle is that the synergistic effect can be reached by targeting different pathogenesis mechanisms and pathways in T2DM.

1.5 Dose Selection, Benefit/Risk Assessment, and Ethical Review

Results from the phase II study showed that glucose-lowering effect was better when HMS5552 dosage reached 75 to 150 mg/day (75 mg BID). The changes of HbA1c from baseline in different groups were: 75 mg QD, -0.39%. 100 mg QD, -0.65%. 50 mg BID, -0.79%. 75 mg BID, -1.12%. Results showed that the efficacy for 50 mg BID and 75 mg BID groups were significantly better than the placebo group, and the efficacy for 75 mg BID group was significantly better than 75 mg QD and 100 mg QD groups. In general, all dosage groups showed good tolerance. No death, SAEs related to study drugs or severe AEs were reported. There were no significant differences in AE incidence across groups. No definitive dose-related AEs were reported [12].

The safety and pharmacodynamic results from the phase II clinical study showed that administration of HMS5552 BID was tolerable and had better efficacy. In the comparison between 50 mg BID and 75 mg BID drug administration, the 75 mg BID showed tolerability and better efficacy meeting clinical needs. Thus, this dosage is expected to have the maximum

benefit/risk ratio for patients. Therefore, 75 mg BID was selected for phase III studies.

In summary, based on the data from preclinical and clinical studies, the benefit/risk ratio is good for subjects who received HMS5552. HMS5552 can be used for the proposed phase III studies.

2 Study Objectives

2.1 The Primary Objective

The primary objective of this study is to assess the change of HbA1c from baseline by comparing the treatment of HMS5552 75 mg BID with placebo in combination with metformin (1500 mg/day) after 24-week treatment in subjects with T2DM whose plasma glucose was inadequately controlled by stable metformin treatment.

2.2 The Secondary Objectives

The secondary objective is to assess the change of HbA1c from baseline by comparing the treatment of HMS5552 75 mg BID with placebo in combination with metformin (1500 mg/day) after 24-week treatment, as well as after the combination of metformin 1500 mg/day with HMS5552 75 mg BID for additional 28-week open-label treatment (Week 52) in subjects with T2DM whose plasma glucose was inadequately controlled by stable metformin treatment:

1. Change of 2h-PPG from baseline at the end of double-blind treatment (Week 24)
2. Change of FPG from baseline at the end of double-blind treatment (Week 24)
3. HbA1c response rate: The proportion of subjects whose HbA1c < 7.0% at the end of double-blind treatment (Week 24)
4. Change of HbA1c from baseline at each visit except Week 24
5. Safety profiles at the end of double-blind treatment (Week 24) and study treatment (Week 52).

3 Study Design

3.1 Study Endpoints

3.1.1 Primary Efficacy Endpoint

To compare the change of HbA1c from baseline after the HMS5552 75 mg BID combined with metformin treatment with the placebo BID combined with metformin treatment after 24-week double-blind treatment in subjects with T2DM and with inadequately controlled plasma glucose by stable metformin treatment

3.1.2 Secondary Efficacy Endpoints

To compare the treatment of HMS5552 75 mg BID group with placebo group both in combination with metformin (1500 mg/day) after 24-week treatment:

1. Change of 2h-PPG from the baseline
2. Change of FPG from the baseline
3. HbA1c response rate: the proportion of subjects whose HbA1c< 7.0%
4. Change of HbA1c from baseline at each visit, except Week 24.

3.1.3 Safety Assessment

- Adverse events (AEs)
- Hypoglycemia events
- Physical examination abnormality
- Vital signs
- 12-lead electrocardiogram (ECG)
- Clinical laboratory examinations (hematology, blood biochemistry and urinalysis)

3.2 Study Design and Rationale

This is a multicenter, randomized, double-blind, placebo-controlled study.

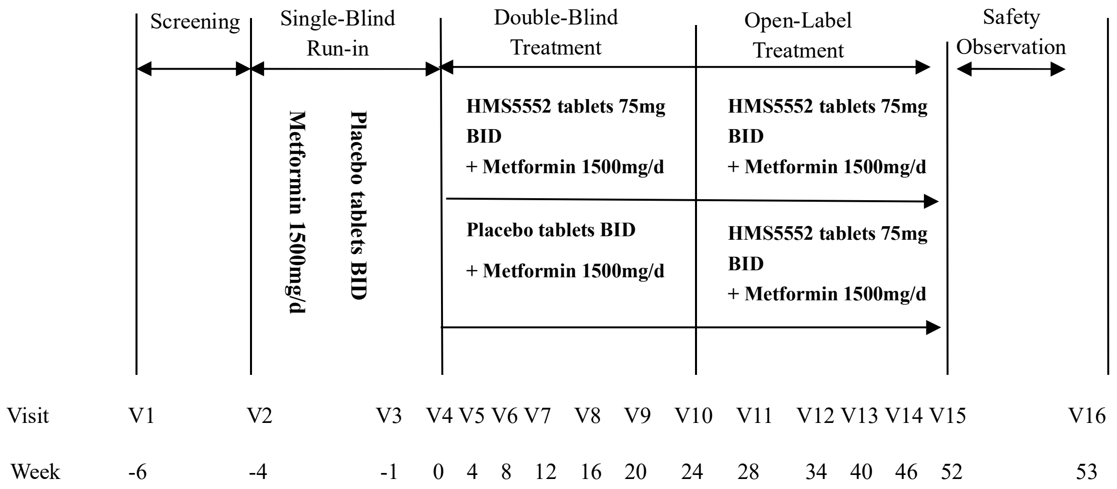

Note: See the Flow Chart of Clinical Trial for visit window details

3.3 Subject Enrollment, Randomization, and Treatment

A total of 750 subjects are planned to enroll in this study. The Investigator:

1. Obtains signed informed consent form (ICF) from potentially eligible subjects before implementing any study procedure.
2. Assigns a unique 8-digit screening number to potentially eligible subjects. The first four digits are for site number and the last four digits are for subject number at the site.
3. Confirms subjects' eligibility according to the inclusion criteria (Section 4.1), exclusion criteria (Section 4.2) and randomization criteria (Section 4.3) defined in this protocol.
4. Assigns a unique randomization number to eligible subject.

Each subject can only be randomized once in this study.

If a subject withdraws from the study, the randomization number can't be reused.

3.3.1 Randomization Method

At screening visit, A unique screening number will be assigned to each subject via the interactive web response service (IWRS) system. This number is used to identify the subject throughout the study and is not used for other subjects.

3.3.2 Run-in Period

In placebo run-in period, sites contact IWRS system to dispense study medications for run-in

period.

3.3.3 Double-Blind Treatment Period

After run-in period, subjects who are eligible for double-blind treatment will be randomized at a ratio of 1:1 to two groups by IWRS system according to the randomization factors (T2DM disease duration and HbA1c level at randomization) entered by the Investigator:

1. Treatment group：HMS5552 75 mg BID + Metformin 1500 mg/day
2. Control group：Placebo BID + Metformin 1500 mg/day

Randomization scheme will be generated from IWRS system (Balance, MedData Solution) and preserved by the IWRS vendor throughout the study. At all visits, study medications are dispensed via the IWRS system along with an identification code (package number). The identification code (package number), which should not be assigned in alphabetic or numerical order and should match the code printed on drug boxes, is provided to the IWRS vendor by drug packaging vendor. The IWRS system is available at any time, 24 hours a day and 7 days a week.

Eligible subjects are able to enter the study. The Investigator assigns each subject an identification code and informs them of the drug administration instructions and time for the first dose of study medications.

Study Period	Study Medications	Drug Name	Morning Dosage	Noon Dosage	Evening Dosage
Run-in	Basic drug	Metformin (Glucophage) 1500 mg/day	Follow doctor's advice	Follow doctor's advice	Follow doctor's advice
	Control drug	Placebo tablets	1 tablet	NA	1 tablet
Double-blind treatment	Basic drug	Metformin (Glucophage) 1500 mg/day	Follow doctor's advice	Follow doctor's advice	Follow doctor's advice
	Study drug	HMS5552 tablets 75 mg	1 tablet (75mg)	NA	1 tablet (75 mg)
	Control drug	Placebo tablets	1 tablet	NA	1 tablet
Open-label treatment	Basic drug	Metformin (Glucophage) 1500 mg/day	Follow doctor's advice	Follow doctor's advice	Follow doctor's advice
	Study drug	HMS5552 tablets 75 mg	1 tablet (75 mg)	NA	1 tablet (75 mg)

Note: refer to Section 5.1.3 for details about drug administration and package

All study medications (including metformin) are used after Visit 15. From Visit 15 to Visit 16, no antidiabetic drugs can be used. The Investigator can give subjects routine medical treatment after completion of Visit 16. Under special medical condition, subjects can receive other antidiabetic treatment in advance or immediately at end of Visit 15 for safety consideration. The reason should be recorded on electronic case report form (eCRF).

3.3.4 Procedures for incorrect enrollment, randomization, or drug administration

Under any circumstance, noneligible or meeting any of the exclusion criteria subjects should not be enrolled or receive the study treatment.

If noneligible subjects are enrolled or randomized to study by mistake, the subjects will not be withdrawn from study automatically by the Investigator. The decision will be made following a fully discussion among the Investigator, Medidata Information Technology (Shanghai) Co., Ltd. (randomization service vendor) and the Sponsor.

If subjects take the wrong drug because of dispensing error, the Investigator should notify the randomization service vendor immediately. The Investigator should not correct the wrong drug or discontinue the subjects by themselves. A final decision is made after fully discussion with Medidata Information Technology (Shanghai) Co., Ltd., and the Sponsor.

3.3.5 Blinding

Double-blind method is used in the study. Placebo tablets are designed with the size, color, smell and appearance same as the active drug tablets. A unique identification number (drug box serial number) is provided by the study medication packing provider and marked on the label of drug box. By central randomization, the randomization codes are generated by IWRS system based on randomization factors (T2DM disease duration ≤ 3 years or > 3 years. and HbA1c level ≤ 8.5% or > 8.5% at randomization) and the block size. Then, the randomization codes along with the corresponding drug box numbers are provided to subjects who meet the randomization criteria at each visit.

During the study, unauthorized people, including Hua Medicine project members or representatives, site staffs, CRO staffs, the data managers and statisticians do not have access to randomization codes.

3.4 Preparation for the Study

If any of the following requirements is not met, the visit schedule should be re-arranged:

- Subject must be in fasting status (for at least 8 hours) before going to regular scheduled visits.
- Any high caloric and high fatty diets are prohibited on the day before the scheduled visit.

- Tobacco, alcohol, and caffeine are prohibited within 8 hours before the scheduled visit.
- Study medications need to be taken one day before the scheduled visit (except Visit 1 and Visit 2 or the terminal visit due to discontinuation of study medications by the subjects).
- On the day for the scheduled visit, the study medications for the morning cannot be taken until all the study procedures have been completed., At Visits 7, 10, and 15, the mixed-meal tolerance test (MMTT) is conducted after drug administration in the morning.

3.5 Study Procedures and Stages

For details of study evaluation procedures please refer to the Flow Chat of Clinical Trial. Each evaluation should be completed within scheduled visit/time. Self-Monitoring of Blood Glucose (SMBG) Procedure please refer to Appendix 4.

3.5.1 Visit 1: Screening (Day -42 to -29)

Subjects read the Informed consent form and the Investigator gives verbal explanation about the study requirements and procedures. The subjects need to sign and date the ICF before taking part in the study. On the day of screening visit, subjects must keep fasting status before sample collection (fasting at least 8 hours, suggest no food or soft drink, except water, from 22:00 pm the day before screening).

Study procedures at Visit 1 include (all laboratory tests are conducted in the central laboratory, and all blood samples are collected in fasting status):

- Informed consent
- Demographics (sex, ethnicity, age, and childbearing potential)
- Medical history, current diseases, and prior medications
- Concomitant medical conditions and medications
- Subjects who are diagnosed with T2DM at screening, and have been continuously treated with metformin≥ 1500 mg/day and have been receiving diet and exercise interventions for at least 12 weeks
- Vital signs (blood pressure, pulse rate, breath rate, body temperature, body weight and height)
- Physical examination: general appearance, head and neck, chest (including heart and lung), abdomen, limbs etc.

- Clinical laboratory tests

  Assessment of diabetes status: HbA1c and FPG.

  Hematology: hemoglobin concentration, hematocrit, erythrocytes, leukocytes, neutrophils, basophils, eosinophils, monocytes, lymphocytes, and platelets.

  Urinalysis: pH, specific gravity, protein, glucose, erythrocytes, leukocytes, and ketone bodies.

  Blood biochemistry: total bilirubin, ALT, AST, gamma-glutamyl transferase, alkaline phosphatase, albumin, total protein, lactate dehydrogenase, urea nitrogen, creatinine, uric acid, sodium, potassium, chloride, calcium, creatine kinase, creatine kinase isoenzyme, total cholesterol, high-density lipoprotein cholesterol, low-density lipoprotein cholesterol, and triglyceride.

  Virology test: A virus IgM antibody (IgM anti-HAV), hepatitis B surface antigen (HBsAg), hepatitis B surface antibody (anti-HBs), IgM antibody to hepatitis B core antigen (IgM anti-HBc), hepatitis C virus antibody (anti-HCV), human immunodeficiency virus (HIV) antibody.

- Biomarker: C-peptide only, evaluated as one exclusion criterion.
- 12-lead ECG.
- Blood pregnancy test (only applicable to females with childbearing potential).
- The inclusion and exclusion criteria are reviewed.

3.5.2 Visit 2: Single-Blind Placebo Run-in Period (Day -28±3 days)

The following procedures are completed at Visit 2 (laboratory tests are conducted in the central laboratory):

- Blood pregnancy test (only applicable to females with childbearing potential).
- AEs and concomitant medications are recorded.
- Glucometers and test strips are dispensed. Instructions on SMBG and hyperglycemia and hypoglycemia management are provided (refer to Appendix 4 and Appendix 8 for details).
- Subject diaries are dispensed. Instructions on recording of blood glucose, drug administration and AEs are provided.
- Diabetes education, instructions on diet and exercise are provided.

- Study medications for run-in period are dispensed.

3.5.3 Visit 3: Pre-randomization Visit (Day -5 ± 2)

The following procedures are completed at Visit 3 (all laboratory tests are conducted in the central laboratory, and all blood samples are collected in fasting status):

- Confirm that subjects have been in the placebo run-in period for about 3 weeks.
- Clinical laboratory tests:

  Assessment of diabetes status: HbA1c and FPG.

  Hematology: hemoglobin concentration, hematocrit, erythrocytes, leukocytes, neutrophils, basophils, eosinophils, monocytes, lymphocytes, and platelets.

  Urinalysis: pH, specific gravity, protein, glucose, erythrocytes, leukocytes, and ketone bodies.

  Blood biochemistry: total bilirubin, ALT, AST, gamma-glutamyl transferase, alkaline phosphatase, albumin, total protein, lactate dehydrogenase, urea nitrogen, creatinine, uric acid, sodium, potassium, chloride, calcium, creatine kinase, creatine kinase isoenzyme, total cholesterol, high-density lipoprotein cholesterol, low-density lipoprotein cholesterol, and triglyceride.

- Blood pregnancy test (only applicable to females with childbearing potential).
- 12-lead ECG.
- SMBG status: review SMBG records and provide instructions on SMBG for next stage.
- AEs and concomitant medications are recorded.
- Diabetes education, instructions on diet and exercise.

3.5.4 Visit 4: Randomization Visit (Day 1)

The following procedures are completed at Visit 4 (all laboratory tests are conducted in the central laboratory and blood samples are collected when subjects are in fasting status, except 30-min and 120-min MMTT samples):

- Vital signs (blood pressure, pulse rate, breath rate, body temperature, and body weight). Subjects who meet the fourth exclusion criterion are excluded, i.e., body weight change (weight gain or loss) at randomization ≥ 10% compared with that at screening.

- Physical examination: general appearance, head and neck, chest (heart and lungs), abdomen, limbs etc.

- Review study medication usage and recycling. Subject diaries are reviewed. The drug administration records in run-in period are reviewed and confirmed. Instructions are given to subjects who do not take study medications per protocol. Drug compliance in the run-in period is calculated. Subjects who meet the fifth randomization exclusion criterion are excluded, i.e., poor drug compliance during run-in period (< 80% or > 120%).

- 12-lead ECG.

- SMBG status: review SMBG record and provide instructions on SMBG for next stage.

- Reconfirm medical history and previous medications.

- The randomization inclusion and exclusion criteria are reviewed (laboratory test results and 12-lead ECG from Visit 3 are used as a reference).

- Randomization.

- Baseline laboratory tests (samples are suggested to be collected between 7:00 and 10:00 am) (baseline value)

  Assessment of diabetes status: HbA1c and FPG.

  Hematology: hemoglobin concentration, hematocrit, erythrocytes, leukocytes, neutrophils, basophils, eosinophils, monocytes, lymphocytes, and platelets.

  Urinalysis: pH, specific gravity, protein, glucose, erythrocytes, leukocytes, and ketone bodies.

  Blood biochemistry: total bilirubin, ALT, AST, gamma-glutamyl transferase, alkaline phosphatase, albumin, total protein, lactate dehydrogenase, urea nitrogen, creatinine, uric acid, sodium, potassium, chloride, calcium, creatine kinase, creatine kinase isoenzyme, total cholesterol, high-density lipoprotein cholesterol, low-density lipoprotein cholesterol, and triglyceride.

- Blood pregnancy test (only applicable to females with childbearing potential).

- MMTT: the test must be conducted before taking study medications. Refer to Appendix 3 for MMTT procedures.

- Whole blood samples are collected from some subjects for DNA test (only for subjects who sign the informed consent form for DNA sampling).

- Biomarkers.
- AEs and concomitant medications.
- A new subject diary is dispensed.
- Diabetes education, instructions on diet and exercise are provided.
- Study medications are dispensed.

3.5.5 Visit 5: Treatment Visit (Day 28 ± 4)

The following procedures are completed at Visit 5 (all laboratory tests are conducted in the central laboratory, and all blood samples are collected in fasting status):

- Vital signs (blood pressure, pulse rate, breath rate, body temperature, and body weight).
- Physical examination: general appearance, head and neck, chest (heart and lungs), abdomen, limbs etc..
- Laboratory tests (samples are suggested to be collected between 7:00 and 10:00 am)

  Assessment of diabetes status: HbA1c and FPG.

  Hematology: hemoglobin concentration, hematocrit, erythrocytes, leukocytes, neutrophils, basophils, eosinophils, monocytes, lymphocytes, and platelets.

  Urinalysis: pH, specific gravity, protein, glucose, erythrocytes, leukocytes, and ketone bodies.

  Blood biochemistry: total bilirubin, ALT, AST, gamma-glutamyl transferase, alkaline phosphatase, albumin, total protein, lactate dehydrogenase, urea nitrogen, creatinine, uric acid, sodium, potassium, chloride, calcium, creatine kinase, creatine kinase isoenzyme, total cholesterol, high-density lipoprotein cholesterol, low-density lipoprotein cholesterol, and triglyceride.

- Blood pregnancy test (only applicable to females with childbearing potential).
- Blood sampling for population PK (before subjects taking the study medications).
- 12-lead ECG.
- SMBG status: review SMBG records and provide instructions on SMBG for next stage.

- Review study medication usage and recycling. The drug administration record is reviewed and confirmed. The remaining medications and used drug packages since last visit are recycled. Instructions are given to subjects who did not take study medications per protocol.
- AEs and concomitant medications.
- A new subject diary is dispensed.
- Diabetes education, instructions on diet and exercise are provided.
- Study medications are dispensed.

3.5.6 Visit 6: Treatment Visit (Day 56 ± 4)

The following procedures are completed at Visit 6 (all laboratory tests are conducted in the central laboratory, and all blood samples are collected in fasting status):

- Vital signs (blood pressure, pulse rate, breath rate, body temperature, and body weight).
- Physical examination: general appearance, head and neck, chest (heart and lungs), abdomen, limbs etc..
- Laboratory tests (samples are suggested to be collected between 7:00 and 10:00 am)

  Assessment of diabetes status: HbA1c and FPG.

  Hematology: hemoglobin concentration, hematocrit, erythrocytes, leukocytes, neutrophils, basophils, eosinophils, monocytes, lymphocytes, and platelets.

  Urinalysis: pH, specific gravity, protein, glucose, erythrocytes, leukocytes, and ketone bodies.

  Blood biochemistry: total bilirubin, ALT, AST, gamma-glutamyl transferase, alkaline phosphatase, albumin, total protein, lactate dehydrogenase, urea nitrogen, creatinine, uric acid, sodium, potassium, chloride, calcium, creatine kinase, creatine kinase isoenzyme, total cholesterol, high-density lipoprotein cholesterol, low-density lipoprotein cholesterol, and triglyceride.
- Blood pregnancy test (only applicable to females with childbearing potential).
- 12-lead ECG.
- SMBG status: review SMBG record and provide instructions on SMBG for next stage.

- Review study medication usage and recycling. The drug administration record is reviewed and confirmed. The remaining medications and used drug packages since last visit are recycled. Instructions are given to subjects who did not take study medications per protocol.
- AEs and concomitant medications.
- A new subject diary is dispensed.
- Diabetes education, instructions on diet and exercise are provided.
- Study medications are dispensed.

3.5.7 Visit 7: Treatment Visit (Day 84 ± 4)

The following procedures are completed at Visit 7 (all laboratory tests are conducted in the central laboratory and blood samples are collected in fasting status, except 30-min and 120-min MMTT samples):

- Vital signs (blood pressure, pulse rate, breath rate, body temperature, and body weight).
- Physical examination: general appearance, head and neck, chest (heart and lungs), abdomen, limbs etc..
- Laboratory tests (samples are suggested to be collected between 7:00 and 10:00 am)

  Assessment of diabetes status: HbA1c and FPG.

  Hematology: hemoglobin concentration, hematocrit, erythrocytes, leukocytes, neutrophils, basophils, eosinophils, monocytes, lymphocytes, and platelets.

  Urinalysis: pH, specific gravity, protein, glucose, erythrocytes, leukocytes, and ketone bodies.

  Blood biochemistry: total bilirubin, ALT, AST, gamma-glutamyl transferase, alkaline phosphatase, albumin, total protein, lactate dehydrogenase, urea nitrogen, creatinine, uric acid, sodium, potassium, chloride, calcium, creatine kinase, creatine kinase isoenzyme, total cholesterol, high-density lipoprotein cholesterol, low-density lipoprotein cholesterol, and triglyceride.
- Blood pregnancy test (only applicable to females with childbearing potential).
- MMTT: the test must be conducted after blood sampling for HbA1c and other laboratory tests are completed, and after taking study medications. Refer to Appendix 3 for MMTT procedures.

- Blood sampling for population PK (before taking study medications, 120min MMTT).
- Biomarkers.
- 12-lead ECG.
- SMBG status: review SMBG records and provide instructions on SMBG for next stage.
- Review study medication usage and recycling. The drug administration record is reviewed and confirmed. The remaining medications and used drug packages since last visit are recycled. Instructions are given to subjects who did not take study medications per protocol.
- AEs and concomitant medications.
- A new subject diary is dispensed.
- Diabetes education, instructions on diet and exercise are provided.
- Study medications are dispensed.

3.5.8 Visit 8: Treatment Visit (Day 112 ± 4)

Procedures that are completed at Visit 8 please see Visit 6.

3.5.9 Visit 9: Treatment Visit (Day 140 ± 4)

Procedures that are completed at Visit 9 please see Visit 6.

3.5.10 Visit 10: Termination Visit for Double-blind Treatment (Day 168 ± 4)

The following procedures are completed at Visit 10 (all laboratory tests are conducted in the central laboratory and blood samples are collected in fasting status, except 30min and 120min MMTT samples):

- Vital signs (blood pressure, pulse rate, breath rate, body temperature, and body weight).
- Physical examination: general appearance, head and neck, chest (heart and lungs), abdomen, limbs etc..
- Laboratory test at end of double-blind treatment (samples are suggested to be collected between 7:00 and 10:00 am)

Assessment of diabetes status: HbA1c and FPG.

Hematology: hemoglobin concentration, hematocrit, erythrocytes, leukocytes, neutrophils, basophils, eosinophils, monocytes, lymphocytes, and platelets.

Urinalysis: pH, specific gravity, protein, glucose, erythrocytes, leukocytes, and ketone bodies.

Blood biochemistry: total bilirubin, ALT, AST, gamma-glutamyl transferase, alkaline phosphatase, albumin, total protein, lactate dehydrogenase, urea nitrogen, creatinine, uric acid, sodium, potassium, chloride, calcium, creatine kinase, creatine kinase isoenzyme, total cholesterol, high-density lipoprotein cholesterol, low-density lipoprotein cholesterol, and triglyceride.

- Blood pregnancy test (only applicable to females with childbearing potential).
- Blood sampling for population PK (before taking study medications, MMTT 120min).
- Biomarkers.
- MMTT: the test must be conducted after blood sampling for HbA1c and other laboratory tests are completed, and after taking study medications. Refer to Appendix 3 for MMTT procedures.
- 12-lead ECG.
- Review SMBG records.
- Review study medication usage and recycling. The drug administration record is reviewed and confirmed. The remaining medications and used drug packages since last visit are recycled. Instructions are given to subjects who did not take study medications per protocol.
- AEs and concomitant medications.
- A new subject diary is dispensed.
- Diabetes education, instructions on diet and exercise are provided.
- Study medications are dispensed.

3.5.11 Visit 11: Open-Label Treatment Visit (Day 196 ± 4)

Procedures that are completed at Visit 11 please see Visit 6.

3.5.12 Visit 12: Open-Label Treatment Visit (Day 238 ± 6)

Procedures that are completed at Visit 12 please see Visit 6.

3.5.13 Visit 13: Open-Label Treatment Visit (Day 280 ± 6)

Procedures that are completed at Visit 13 please see Visit 6.

3.5.14 Visit 14: Open-Label Treatment Visit (Day 322 ± 6)

Procedures that are completed at Visit 14 please see Visit 6.

3.5.15 Visit 15: Termination Visit for End of Open-Label Treatment (Day 364 ± 6)

The following procedures are completed at Visit 15 (all laboratory tests are conducted in the central laboratory and blood samples are collected in fasting status, except 30-min and 120-min MMTT samples):

- Vital signs (blood pressure, pulse rate, breath rate, body temperature and body weight).
- Physical examination: general appearance, head and neck, chest (heart and lungs), abdomen, limbs etc..
- Laboratory tests at end of study (samples are suggested to be collected between 7:00 and 10:00 am)

  Assessment of diabetes status: HbA1c and FPG.

  Hematology: hemoglobin concentration, hematocrit, erythrocytes, leukocytes, neutrophils, basophils, eosinophils, monocytes, lymphocytes, and platelets.

  Urinalysis: pH, specific gravity, protein, glucose, erythrocytes, leukocytes, and ketone bodies.

  Blood biochemistry: total bilirubin, ALT, AST, gamma-glutamyl transferase, alkaline phosphatase, albumin, total protein, lactate dehydrogenase, urea nitrogen, creatinine, uric acid, sodium, potassium, chloride, calcium, creatine kinase, creatine kinase isoenzyme, total cholesterol, high-density lipoprotein cholesterol, low-density lipoprotein cholesterol, and triglyceride.
- Blood pregnancy test (only applicable to females with childbearing potential).
- Blood sampling for population PK (before taking study medications, 120-min MMTT).
- Biomarkers.

- MMTT: the test must be conducted after blood sampling for HbA1c and other laboratory tests is completed, and after taking study medications. Refer to Appendix 3 for MMTT procedures.
- 12-lead ECG.
- Review SMBG records.
- Review study medication usage and recycling. The drug administration record is reviewed and confirmed. The remaining medications and used drug packages since last visit are recycled.
- AEs and concomitant medications.
- Diabetes education, instructions on diet and exercise are provided.
- Subjects are reminded that all study medications (including metformin) are stopped after Visit 15. No antidiabetic drugs are allowed between Visits 15 and 16. Under special medical conditions, other antidiabetic treatment must be given immediately after Visit 15. In this case, for safety considerations, routine treatment can be started at an early stage but the reason should be recorded on eCRF.

3.5.16 Visit 16: Follow-up Visit for Safety (Day 371 ± 2)

The following procedures are completed at Visit 16:

- Existing AEs and possible new AEs after end of treatment via phone-call.
- Concomitant medications via phone-call.
- Between Visits 15 and 16, subjects can contact the Investigator if there is any discomfort or hyperglycemia event. Depending on disease status, the Investigator decides if Visit 16 will be conducted in advance by phone. For safety considerations, routine antidiabetic treatment can be provided to subjects earlier, which needs to be recorded on eCRF. Routine medical care is given after Visit 16.

3.6 Study Materials Provided to Subjects

The following materials are provided to study sites by Hua Medicine:

- Blood glucose meter: a portable blood glucose meter is provided to each subject at Visit 2.
- Matched glucose test strips.
- Matched blood sampling needles.

- Subject diaries for recording daily drug usage, blood glucose value and any discomfort.
- Health care education materials.

4 Study Population

4.1 Inclusion Criteria

Subjects must meet all following inclusion criteria to enter the study (all laboratory parameters are tested in the central laboratory):

1. Male or female, aged 18~75 years old (inclusive) when they sign ICF.
2. Subjects diagnosed as T2DM in accordance to the WHO diagnostic criterion published in 1999 at screening period and treated with stable metformin ≥ 1500 mg/day constantly for at least 12 consecutive weeks in addition to dietary and exercises intervention.
3. 7.5% ≤ HbA1c ≤ 10.0% at screening.
4. 18.5 kg/m^2 < BMI < 35.0 kg/m^2 at screening.
5. Willing to follow the consistent diet and exercise interventions from the run-in period throughout the whole study. willing to take medication and meals on time per study protocol requirements. and to take timely SMBG and recording.
6. Willing to sign the written ICF and comply with the study protocol.

4.2 Exclusion Criteria

Exclusion Criteria

The subject cannot be enrolled with any of the following criteria (all laboratory parameters will be tested in the central laboratory).

Exceptions for Target Disease

1. Any antidiabetic therapy other than metformin within 12 weeks before screening.
2. Insulin therapy more than 30 days within 1 year before screening.
3. History of severe hypoglycemia (requiring external assistance for recovery) or recurrent hypoglycemia (such as experience of 3 or more times of hypoglycemia <=3.9 mmol/L or hypoglycemia symptoms within 1 month) without contributing factors within 3 months before screening.

4. Fasting C-peptide <0.81ng/ml (0.27 nmol/L) at screening visit.
5. Medical history of diabetic ketoacidosis, diabetes lactic acidosis or hyperosmotic nonketotic diabetic coma.
6. Subjects clinically diagnosed as type 1 diabetes mellitus, or diabetes mellitus caused by pancreatic damage or other specific types of diabetes.

Medical History and Concomitant Disease

7. Have severe cardio-cerebrovascular medical history defined as:
   a) Within 6 months prior to screening, subjects had medical history of myocardial infarction, coronary angioplasty or bypass graft(s) surgery, valvular heart disease or valve repair, and clinically significant unstable arrhythmia, unstable angina pectoris, transient ischemic attack, or cerebrovascular accident.
   b) Congestive heart failure defined as New York Heart Association (NYHA) stage III and IV.
8. Medical history of unstable or rapidly progressive kidney disease.
9. Active liver diseases at screening.
10. Medical history of diagnosed mental disease.
11. Medical history of hemoglobinopathies (sickle cell anemia or thalassemia, and sideroblastic anemia).
12. The immunocompromised individuals such as subjects who had undergone organ transplants or diagnosed as human immunodeficiency virus (HIV) infection.
13. Subjects with any type of malignant tumor (regardless of cured or not).
14. Based on Investigator's judgment, any unstable or medical intervention-needed endocrine system diseases related to blood glucose (such as hyperthyroidism, acromegaly syndrome, Cushing's syndrome) or immune system disease that are improper for enrollment.
15. Medical history of drug abuse.
16. Subjects who received corticosteroids treatment (except for short-term external use) within 1 year at screening.
17. Alcohol intake> 2 units a day, or> 14 units a week.

    One unit of alcohol equivalent to 150 mL of wine, 350 mL of beer, 100 mL (<=17%)

or 80 mL(>17% and <=24%) of low-alcohol liquor, or 50 mL of high-alcohol liquor (>24%).

Physical Examination and Laboratory Results

18. Significant abnormal liver function results at screening, defined as ALT > 2.5×ULN, or AST > 2.5×ULN or TBiL > 1.5×ULN (refer to Appendix 6).

19. Have serological evidence of infectious liver disease at screening defined as: positive IgM anti-HAV, or positive HBsAg and IgM anti-HBc (to exclude subjects with acute hepatitis virus B infection), or positive anti-HCV.

20. Estimated glomerular filtration rate (eGFR) < 60 mL/min/1.73m2 at screening.

21. Triglyceride (TG) > 5.7 mmol/L at screening.

22. Anemia (defined as hemoglobin < 12.0 g/dL (120 g/L) for men and hemoglobin < 11.0 g/dL (110 g/L) for women) of any causes at screening.

23. Based on Investigator's judgment, any abnormal laboratory parameters that may affect the safety evaluation.

24. Based on Investigator's judgment, any abnormal ECG that may affect the safety evaluation (except for stable coronary heart disease).

25. Systolic blood pressure ≥ 160 mmHg or diastolic blood pressure ≥ 100 mmHg at screening, or who added/changed antihypertensive drugs or adjusted dose within 4 weeks before screening.

Allergies and Adverse Drug Reactions

26. Based on Investigator's judgment, subjects may be allergic or intolerant to Investigational drug.

Prohibited Therapies and/or Medications

27. Subjects who refuse to take antidiabetic medication only required in this study.

28. Concomitant treatment with a potent or moderate CYP3A4 inhibitor or inducers (refer to Section 4.4.2).

Sex and Reproductive Status

29. Women who are pregnant or intend to be pregnant during study.

30. Women in breastfeeding.

31. Women who have childbearing potential and refuse to use a high-efficient and accredited contraceptive method through study startup to 1 month after the last medication with study drug.

Other Exclusion Criteria

32. Based on Investigator's judgment, any accompanied conditions or diseases may interfere with subjects to complete the whole study or to participate all processes of the study.

33. Based on Investigator's judgment, subjects need other therapy which may potentially affect the interpretation of the efficacy and safety data of the study.

34. Based on Investigator's judgment, subjects who are unable to comply the requirements specified in the protocol, for instance: fail to keep the dietary and exercise control during the study, fail to take required medicine and meal timely per protocol requirements. and fail to conduct SMBG and make record timely etc.

35. Subjects who participated in any clinical trials or took any study drug within 30 days before screening.

4.3 Randomization Criteria (Laboratory tests and 12-lead ECG will be conducted on Day -5 [± 2 days] prior to the randomization)

Before randomization of Visit 4 (randomization visit), subjects are re-evaluated for eligibility by following parameters. Those who meet randomization criteria are eligible to enter the randomization period.

Inclusion Criteria

1. HbA1c ⩾ 7.5% and ⩽ 10.0% at pre-randomization visit.

2. Fasting blood glucose> 7.0 mmol/L and < 13.3 mmol/L at pre-randomization visit.

Exclusion Criteria

3. History of severe hypoglycemia (requiring external assistance for recovery) or recurrent hypoglycemia (such as experience of 3 or more times of hypoglycemia <=3.9 mmol/L or hypoglycemia symptoms within 1 month) without contributing factors within 3 months in run-in period.

4. Body weight changes (weight gain or loss) ≥ 10% at randomization compared with that at screening.

5. Poor drug compliance (< 80% or > 120%) during run-in period.

6. Significant abnormal liver function in run-in period, defined as ALT > 2.5×ULN, or AST > 2.5×ULN or TBiL > 1.5×ULN at pre-randomization visit (refer to Appendix 6).

7. Estimated glomerular filtration rate< 60mL/min/1.73m^2 at pre-randomization visit.

8. Triglyceride> 5.7mmol/L at pre-randomization visit.

9. Anemia (defined as hemoglobin < 12.0 g/dL (120 g/L) for men and hemoglobin < 11.0 g/dL (110 g/L) for women) of any causes at pre-randomization visit.

10. Systolic blood pressure ⩾ 160 mmHg or diastolic blood pressure ⩾ 100 mmHg at randomization visit, or who added/changed the antihypertensive drugs or adjusted dose within run-in period.

11. Based on Investigator's judgment, any abnormal laboratory parameters that may affect the safety evaluation.

12. Based on Investigator's judgment, any abnormal ECG that needs medical intervention or may affect the safety evaluation (except for stable coronary heart disease).

13. Additionally, the Investigator should re-confirm the medical history and concomitant disease from screening to randomization visit (refer to exclusion criteria at screening).

4.4 Concomitant Medication and Post-study Treatment

4.4.1 General Instructions on Study Treatment

Investigator can use other medications if they are critical for the safety and health of subject. All medication taken must be recorded in eCRF.

From the ICF signed date to one week after treatment completion (Visit 16), any concomitant medications (study medications and other medications) or the changes on dosages of previous medications must be documented in the corresponding eCRF section. Reasons for giving the medications, administration dates (start and stop dates), and dosage information (dose, route, frequency) should also be documented.

4.4.2 Prohibited and Restricted Medications

Other antidiabetic drugs, except the study medications, are not allowed to be used throughout the study from the run-in period. If hyperglycemia happens and meets the withdraw criteria, the subject will be treated as withdrawal. This does not apply to those medication used for AE treatment or concomitant medications which have been used stably before the study.

Unless emergency, subjects are not allowed to take any new medications which have not been used at screening, or adjust current dosage of any study medication or concomitant medications without permission from the Investigator. In case of any emergency when there is any clinical indication needing additional medications or dosage change of concurrent medications, the subjects must inform the Investigator within reasonable timeframe before concomitant medications are started. The information, including drug name, dosage, route of administration, treatment purpose and administration dates are confirmed and recorded by the Investigator.

The following medications are strictly prohibited unless for special medical conditions

- Any antidiabetic drug (including traditional Chinese medicine) other than the study medications (starting from the run-in period)
- Hypoglycemic healthcare product
- Oral or injectable or inhaled glucocorticoid
- Any known potent or moderate liver enzyme CYP450 3A4 inducers/inhibitors, including but not limited to:

  Potent liver enzyme inducers: barbitals (especially phenobarbital), carbamazepine, diphenylhydantoin sodium, rifampin, dexamethasone, etc.

  Potent liver enzyme inhibitors: ketoconazole, itraconazole, voriconazole, posaconazole, clarithromycin, indinavir, ritonavir, saquinavir, telithromycin, Boceprevir, nelfinavir, Telaprevir, conivaptan, nefazodone.
- Any unauthorized drugs (i.e., investigational drugs not marketed yet).

4.4.3 Contraception

From screening to 1 month after the last dose of study medications, female subjects with childbearing potential must use high-efficiency, medically approved contraception methods (e.g. barrier type intrauterine device combined with spermicides, IUD implanted at least 3 months before study, oral contraceptives and preventive measures). The same method is recommended to be used throughout the study.

See Section 7.12 in the protocol for procedures for handling pregnancy.

4.4.4 Diet Control and Exercise Intervention

Instructions are given to subjects on nutrition and physical exercise, how to identify the signs and symptoms of hypoglycemia and hyperglycemia, and how to use blood glucose meter (refer to Appendix 4). The Investigator should ask subjects to keep fasting for at least 8 hours before each visit to study sites (from Visit 3 to Visit 15). Each site is suggested to give subjects instructions on nutrition and diet and ask them to follow the standard diet guideline for diabetes

patients (refer to Appendix 8).

4.4.5 Post-Study Treatment

After 52-week treatment is completed, all the investigational drugs (HMS5552 tablets and metformin) are stopped and subjects proceed to 1-week safety observation period. In the safety observation period, in principle, no antidiabetic drugs can be used until the Visit 16 telephone follow-up is completed. Routine treatment can be initiated after Visit 16.

Subjects can contact the Investigator if any hyperglycemia or discomfort occurs between Visits 15 and 16. Depending on disease status, the Investigator decides whether to conduct the telephone follow-up in advance. Under special medical conditions, subjects can take routine antidiabetic treatment in advance for safety consideration. The reasons need to be recorded on eCRF.

4.5 Discontinuation of Study Treatment, Withdrawal, or Termination of Study

During the study, the Investigator should make every effort to encourage subjects to complete the entire study.

Subjects have the right to withdraw from the study treatment for any reason and at any time. The Investigator has the right to require subjects to discontinue the study treatment or withdraw from the study.

In addition, the reasons for discontinuation of study treatment or withdrawal from the study include but not limited to:

- Withdrawal of informed consent form by subjects
- AEs
- Hyperglycemia
- Hypoglycemia
- Pregnancy
- Subjects do not comply with study treatment or scheduled visits (e.g., drug administration or visits not following Investigator's instructions)
- Major protocol deviations and high risks to subjects as considered by the Investigator
- Lost to follow-up
- Others.

Subject withdrawal occurring before randomization for any reason (e.g., not meeting the inclusion criteria) is considered as “screening failure”.

The Sponsor can terminate the study or certain study site based on the overall consideration for the study.

Specific early withdrawal criteria due to hypoglycemia, hyperglycemia, and abnormal liver function have been defined to ensure subject safety. See Sections 4.5.1 to 4.5.3.

Subjects who expect to re-enter the study after discontinuation of study treatment is allowed if the interval is ≤ 2 weeks since discontinuation, which will be decided by the Investigator after consultation with the Sponsor.

4.5.1 Withdrawal Due to Hypoglycemia

4.5.1.1 Definition of Hypoglycemia

Hypoglycemic events are collected and classified as follows according to the China guidelines for the prevention and treatment of T2DM (2013)[3]. The Clinical Trials Guidelines of Diabetes Treatment Drug and Biological Products (2012)[16], and a joint statement made by the American Diabetes Association (ADA) and the European Association for the Study of Diabetes (EASD) recently published in November 2016 for identifying and reporting hypoglycemic events [17]:

1. Fingertip or venous plasma glucose value **≥ 3.0 mmol/L, ≤ 3.9 mmol/L**, with or without symptoms of hypoglycemia.
2. Fingertip or venous plasma glucose value **< 3.0 mmol/L**, with or without symptoms of hypoglycemia.
3. **Severe hypoglycemia**: assistance from others is needed, usually with disturbance of consciousness, and neurological symptoms improve significantly or disappear after correction of hypoglycemia.

Hypoglycemia symptoms may include shakiness, hot flashes, vertigo, weakness, cold limbs, anxiety, dizziness, hunger, palpitations, and blurred vision, etc.

1. In addition, based on The Clinical Trials Guidelines of Diabetes Treatment Drug and Biological Products (2012), the following possible hypoglycemic events are also collected but will not be used for hypoglycemia assessment:

Possible symptomatic hypoglycemia: presenting with symptoms of hypoglycemia, but without plasma glucose test results.

Relative hypoglycemia: presenting with hypoglycemia symptoms, but with plasma glucose > 3.9 mmol/L (fingertip or venous).

4.5.1.2 Rules for Withdrawal due to Hypoglycemia

2. If severe hypoglycemia occurs (help from others is needed, usually with disturbance of consciousness, and neurological symptoms improve significantly or disappear after correction of hypoglycemia) and no other reason (e.g., physical exercise increase or meals skipping) except for the study medications can be found, the study medications are discontinued and the subjects withdraw from the study.

3. If fingertip or venous plasma glucose value is < 3.0 mmol/L and the result occurs twice at two consecutive visits without other reasons (e.g., physical exercise increase or meals skipping) except for the study medications, the study medications are discontinued and the subjects withdraw from the study.

Subjects should be informed to contact study sites in following conditions:

Any hypoglycemia that needs help (i.e., severe hypoglycemia), or any occurrence of hypoglycemia with fingertip or venous plasma glucose value ≤ 3.9 mmol/L.

Notes: As noted before, subjects record all symptoms related to hypoglycemia and fingertip plasma glucose values in subject diaries. The Investigator should review each hypoglycemia event and carefully inquire about the associated symptoms, treatment, and duration etc. Hypoglycemia documented in subject diaries needs to be confirmed by reviewing glucose meter records. All hypoglycemic events confirmed by the Investigator must be recorded on the hypoglycemia review form in eCRF.

4.5.2 Withdrawal due to Hyperglycemia

Between Visits 2 and 15, subjects should carry out SMBG according to SMBG guidance (refer to Appendix 4 for procedures of SMBG). Subjects should contact the Investigator timely if plasma glucose exceed the value specified in SMBG guidance. Depending on the fingertip plasma glucose values, diet and exercise condition, the Investigator determines whether the subjects should return to study sites in advance for laboratory tests.

If a subject has a FPG value reaching the following criteria twice (at least once tested in the central laboratory) without any apparent causes at two consecutive visits, the Investigator will evaluate whether the subject will withdraw from the study with the reason recorded as hyperglycemia based on subject's actual situation.

1. Day 1–before Week 12: venous FPG (central laboratory) ≥ 13.3 mmol/L
2. Week 12–before Week 24: venous FPG (central laboratory) ≥ 11.1 mmol/L
3. Week 24–Week 52: venous FPG (central laboratory) ≥ 11.1 mmol/L.

If a subject is confirmed to withdraw from the study due to hyperglycemia, the subject must

complete termination visit before withdrawal (refer to Section 4.6). Then, the study medications can be discontinued and routine diagnosis and treatment can be started.

The Investigator must ensure no other causes can be identified to explain poor glycemic control except for lack of efficacy (e.g., acute inflammations/infections, concomitant treatments, etc.). If the disease aggravation is unexpected progression, it should be reported as an AE.

4.5.3 Withdrawal due to Abnormal Liver Function

Depending on the subject's condition, study treatment can be discontinued if any of the following criteria is met (no need to wait for the retest results):

- ALT or AST > 8×ULN
- ALT or AST > 5×ULN and lasts for ≥ 14 days
- ALT or AST > 3×ULN and TBiL > 2×ULN
- ALT or AST > 3×ULN with liver damage symptoms (e.g., fatigue, nausea, vomiting, right upper abdominal pain or tenderness, fever, rash) or eosinophilia increase.

Active intervention or treatment needs to be given to subjects. The causes for abnormal liver function need to be identified. Consultation with hepatologists will be conducted if needed. Subjects with abnormal liver function are closely followed up until the liver function restore or the results return to baseline level. The termination visit should be scheduled as soon as possible within 3 days after discontinuation of study treatment. AEs relevant to abnormal liver function need to be recorded in time. SAEs which meet the criteria should be reported immediately.

4.6 Procedures for Subject Withdrawal

All subjects who withdraw from the study are kept under medical monitoring. The reasons for withdrawal need to be confirmed by contacting the subject or the relatives by phone or email. If withdrawal occurs during the treatment period, the efficacy and safety observations and assessments (same as Visit 15) at the termination visit need to be completed as much as possible within 7 days (including the seventh day) after the last dose of study medications. Safety assessment via phone within 1 week after the last dose of study medications also needs to be completed. If the study treatment has been discontinued by subjects for more than 7 days before the withdrawal, the assessments (same as Visit 15) at termination visit is strongly recommended to be completed. If study treatment has been discontinued at the termination visit, the MMTT can be cancelled.

For subjects who withdraw early from the study, a comprehensive evaluation is conducted to find the reasons for early withdrawal. Withdrawal reasons should be recorded in detail. For example, the Investigator should ask about the reasons for inform consent withdrawal, rather than just to record the withdrawal.

If the early withdrawal from study is due to AEs or abnormal laboratory results, the details should be recorded on eCRF. AEs are recorded and followed up (see Section 7.2). Subject diaries, all remaining medications and used drug packages should be returned.

4.7 Lost to Follow-up

All reasonable efforts must be made to contact subjects to confirm and record their current situation. Actions include contacting the authorized personnel (e.g. spouses, children or other relatives). Lost to follow-up is defined as, as shown in records, failure to reach subjects via phone calls, faxes or emails at least 3 times within 1 month (each contact is on different calendar days). All attempts should be recorded in subjects' medical records. If death is confirmed, the study sites acquire the date and cause for the death in a way permitted locally. If a subject is still lost to follow up after all efforts, the last known survival date should be decided by the Investigator and recorded in subjects' medical records.

5 Study Drugs

5.1 Study Medications

5.1.1 Drug Name, Strength, Formulation

Investigational Drug

HMS5552 tablets. formulation: light yellow, double convex, round, film-coated tablet. specification: 75mg/tablet. Batch No.: BXE17001 (Double-blind treatment period), BXE17001 or BXE18001 (open-label treatment period). Administration route: oral. Drug supplier: Hua Medicine (Shanghai) Ltd.

Control Drug

Placebo tablets, no active drug ingredient, but have the same appearance, odor, and color etc. with HMS5552 tablets. Batch No.: BXK17001. Administration route: oral. Drug supplier: Hua Medicine (Shanghai) Ltd.

Basic Drug: Metformin (Glucophage)

Formulation: tablet. specification: 500mg/tablet. Administration route: oral. Batch No: refer to original package. Manufacturer: Sino-American Shanghai Squibb Pharmaceuticals Ltd.

5.1.2 Packaging and Labelling

All study medications are packed and labeled in compliance with GMP requirements.

The packing method used for 4-week visit period is as follows:

- The packing details for basic drug (Glucophage, metformin hydrochloride tablets): the

original package from the provider is kept, i.e., each small box contains 2 packs with 10 tablets for each (total 20 tablets). For the second packaging, the box in medium size containing 5 small boxes is prepared for 4-week (28 days) dosage (totally 5 small boxes. each small box contains 2 packs with 10 tablets for each. Thus, a total of 20 tablets in small box and 100 tablets in medium box). Of the 100 tablets, 84 tablets are for 4-week dosage (28 days, 3 tablets/day) and 16 tablets are for backup.

- The single-blind placebo run-in period lasts for 4 weeks, in which the study medications are administered BID. For each subject, all the study medications (control drug) for run-in period are packed together as one package (dosage for 4 weeks). Each package consists of 8 packs and each pack contains 8 tablets, 64 tablets in total. Of the 64 tablets, 56 tablets are used within 4 weeks (28 days), and 8 tablets are for backup.

- After randomization, study medications (investigational drug and control drug) are packed separately with a unique packing number (study drug box serial number). Each package contains the dosage for 4-week treatment. Each package consists of 8 packs and each pack contains 8 tablets, 64 tablets in total. Of the 64 tablets, 56 tablets are used within 4 weeks (28 days), and 8 tablets are for backup.

Package for 6-week visit period is as follows:

- The packing details for basic drug (Glucophage, metformin hydrochloride tablets): the original package from the provider is kept, i.e., each small box contains 2 packs with 10 tablets for each (total 20 tablets). For the second packaging, the box in medium size containing 8 small boxes is prepared for 6-week (42 days) dosage (totally 8 small boxes. each small box contains 2 packs with 10 tablets for each. Thus, totally 20 tablets in small box and 160 tablets in medium box). Of the 160 tablets, 126 tablets are for 6-week dosage (42 days, 3 tablets/day) and 34 tablets are for backup.

- Each package contains the study medications for 6-week treatment. Each package consists of 12 packs and each pack contains 8 tablets, 96 tablets in total. Of the 64 tablets, 84 tablets are used within 6 weeks (42 days), and 12 tablets are for backup.

Study medications are sent to each study site separately and the Investigator at each site is responsible for drug storage.

Drug label should include at least information below:

- Sponsor name
- Study medications/formulation, administration route, and the quantity of dosage unit
- Drug storage conditions
- Study number

- Drug box serial number
- Use instructions
- Investigator's name (add to the label when dispensing study medications, if applicable)
- Expiry date (term of validity)
- "For clinical study use only"
- "Keep out of reach of children".

5.1.3 Study Medication Regimen and Packaging

Subjects from each group orally take the following medications all at once, BID. The medications are suggested to take at a draught before breakfast and supper (If the Investigator considers metformin should be administered 3 times a day, another dose is suggested to be taken in lunch time). The time for drug administration is suggested to be consistent as much as possible throughout the study.

Single-blind run-in period

Placebo tablets

First dose Placebo tablet×1, taken at a draught before breakfast

Second dose Placebo tablet×1, taken at a draught before supper

Metformin is taken following doctor's advice

Double-blind treatment period

Group A HMS5552 tablets, 75mg BID group

First dose HMS5552 tablet 75mg×1, taken at a draught before breakfast

Second dose HMS5552 tablet 75mg×1, taken at a draught before supper

Metformin is taken following doctor's advice

Group B Placebo tablets, placebo BID control group

First dose Placebo tablet×1, taken at a draught before breakfast

Second dose Placebo tablet×1, taken at a draught before supper.

Metformin is taken following doctor's advice

Open-label treatment period

Group A HMS5552 tablets, 75mg BID group

First dose HMS5552 tablet 75mg×1, taken at a draught before breakfast

Second dose HMS5552 tablet 75mg×1, taken at a draught before supper

Metformin is taken following doctor's advice

Group B HMS5552 tablets, 75mg BID group

First dose HMS5552 tablet 75mg×1, taken at a draught before breakfast

Second dose HMS5552 tablet 75mg×1, taken at a draught before supper.

Metformin is taken following doctor's advice

All drug storage information (empty packs, partially used or unused drugs) should be recorded and monitored regularly by clinical research associate (CRA). All unused medications must be returned to the Investigator at each visit. The Investigator returns all received medications to the Sponsor after study closure.

5.2 Storage Condition

HMS5552 is recommended to be stored under room temperature (10–30°C), avoiding high humidity and strong light.

The basic drug metformin (Glucophage) is recommended to be stored in sealed condition. Details are provided in drug instructions.

The Investigator should pay adequate attention to ensuring the study medications to be securely stored in locked cabinets with limited access or in other fully secured locations.

5.3 Drug Dispensing and Counting

The Investigator and/or study sites are responsible for establishing a system to manage the study (including study medications) and ensure that:

- The Investigator or designated staff can correctly receive the study medications from Hua Medicine (Shanghai) Ltd.
- Receiving and dispensing of study medications is recorded in the drug inventory. The Investigator is required to ensure the correct records to demonstrate the receiving and disposition of the study medications (Hua Medication Ltd. or designated organizations provide copies of listings for shipping and delivery of study medications). The following information is recorded: the person to whom the drug is dispensed, the quantity, the date

of drug dispensing, and any unused drugs returned to the Investigator. The information is provided as supplementary information to the records on eCRF. After study completion, drug inventory and dispensing log should be checked and confirmed, and any discrepancy should be clarified. The dispensing and return of study medications requires to be confirmed by signature, ideally by the Investigator or his designees.

- Study medications must be handled and stored safely and appropriately.
- Storage of study medications (empty packs, partially used or unused medications) should be recorded and monitored by the CRAs.
- The study medications are only dispensed to subjects taking part in the study.
- Only the Investigator, Sub-Investigator or their designees are authorized to dispense the study medications. The study medications can only be used in this study per study protocol. Without the permission from Hua Medicine (Shanghai) Ltd, the study medications should not be used for other purposes.

5.4 Drug Return and Destruction

At each visit, all unused study medications and used packages for that visit must be returned to the Investigator. The study staffs count all returned medications and empty packs. The drug compliance is determined by comparing the number of returned tablets with the number of dispensed tablets. The results must be documented on corresponding eCRF pages.

All returned medications and empty packs are kept by the Investigator, and returned to designated places or other companies designated by Hua Medicine for destruction. Hua Medicine (Shanghai) Ltd. or designated organizations provide a receipt (or relevant form) to document drug return.

5.5 Drug Compliance

For subjects who are followed up in the study, “planned dosage” stands for the planned total dose that should be taken in certain periods (e.g. run-in or treatment periods). If early withdrawal from the study occurs, the planned dosage stands for the total dose that should be taken from beginning of that period to the last day of administration. Actual dosage means: dispensed quantity - returned quantity - missed quantity (must be verified and consistent with the record in subject diaries. If not, details of actual drug usage should be checked and recorded).

The following formula is applied to calculate the percentage of drug compliance:

$$\text{Percentage of Drug Compliance} = \frac{Actual\ \ Dosage}{Planned\ \ Dosage} \times 100\%$$

Drug compliance lower than 80% or greater than 120% suggests a poor drug compliance.

5.6 Extent of Exposure

Extent of exposure is summarized based on the drug compliance results.

6 Study Endpoints

The methods for assessment and documentation of study endpoints and time for assessment are as follows. The efficacy parameters include HbA1c, PPG and FPG.

Follow up visits are scheduled at Weeks 4, 8, 12, 16, 20, 24, 28, 34, 40, 46, 52 after study treatment starts. Efficacy parameters, e.g. HbA1c and FPG, are tested at each visit (refer to the Flow Chart of Clinical Study for details on assessments). The visit window for Weeks 4, 8, 12, 16, 20, 24 and 28 is defined as ± 4 days of visiting day. whereas the visit window for Weeks 34, 40, 46, and 52 is defined as ± 6 days of visiting day. and visit window of ± 2 days for Week 53 phone visit is suggested. The schedule of visit window is based on normal working time. In case of the National Day, Spring Festival and other holidays, the Investigator can arrange for administration of the study medications accordingly and reschedule the next visit to a time closest to the visit scheduled per study protocol. Causes for early/late visit are recorded on eCRF and this is not considered as a protocol deviation.

Study visits are recommended to be arranged between 7:00 and 10:00 a.m. High calorie and high fat diet is not permitted the day before the study visit. Subjects should stay in fasting status for at least 8 hours from 22: 00 on the previous day until sampling for the visit is completed, without intake of food/drink (water is permitted). If a subject is not prohibited from high calorie and high fat diets or not fasted, the sampling for the subject is suggested to be done in the morning next to the scheduled visit. The Investigator should emphasis the importance of prohibition on high calorie and high fat diets and sampling in fasting status.

The Investigator should inform the subjects not taking study medications in the morning of study visit. The study medications should be taken after sampling in fasting status. Then the MMTT is conducted. The Investigator should also remind subjects of returning the remaining medications and used drug packages on the day of study visit.

Procedures of MMTT:

The MMTT is a standardized method of measuring PPG level. In this study, MMTT is conducted to assess the impact of HMS5552 on PPG, insulin, and C-peptide. (Note: in this study, all meals for MMTT are referred to instant noodles standard meal provided by the Sponsor. The usage method and cautions are described in Appendix 3).

If a subject withdraws from the study early, and already has discontinued study medications at the termination visit, the MMTT is not needed (in other cases, the procedures are consistent with Visit 15).

7 Safety Assessments

7.1 Adverse Event (AE)

AE refers to any untoward medical occurrence, including exacerbation of a pre-existing condition, in a patient or a subject in a clinical study who has been administered with an investigational drug, no matter its causal relationship with the investigational drug. An AE can therefore be any unfavorable and unintended disease (e.g., acute appendicitis), symptom (e.g., nausea, chest pain), sign (e.g., tachycardia, hepatomegaly), or abnormal laboratory finding (e.g., laboratory tests, ECG).

AEs include serious and non-serious AEs.

7.1.1 Serious Adverse Event (SAE)

SAE is defined as an AE occurring in any period of the study (i.e. run-in, treatment, follow-up) that meets one or more of the following criteria:

1. Resulting in death
2. Immediately life-threatening

The term "life-threatening" refers to an event in which the subject is at immediate risk of direct death at the time of the event. It does not include the situation that might lead to death if it were more severe. For example, drug-induced hepatitis resolved without hepatic failure cannot be considered as life-threatening though it might be fatal.

3. Requiring hospitalization or prolonged hospitalization

Hospitalization or prolonged hospitalization due to elective surgery, routine clinical procedures, social reasons or self-convenience do not need to be recorded as an AE. If the events meet AE criteria e.g., aggravation from previous condition, they should be reported as “serious” or “non-serious” AEs based on routine criteria.

4. Resulting in persistent or significant disability/incapacity
5. Congenital anomaly/birth defect
6. Important medical event: to preclude one of the outcomes listed as above, medical and scientific judgment should be made to assess whether other situations should be considered as serious, such as important medical events that may not be immediately life-threatening or result in death or hospitalization but may jeopardize the subject or may require medical intervention. Examples of such events include allergic bronchospasm requiring intensive treatment in an emergency room or at home. blood dyscrasias or convulsions that do not result in hospitalization. drug dependency or drug abuse, etc.

7.1.2 Persistent or Recurrent AEs

Persistent AEs are events that continuously exist during the assessment period without resolution. These events should be recorded only once on eCRF. The initial severity of this event should be recorded, and the highest severity should be updated once the event is aggravated. If the event becomes an SAE, corresponding update should be made on eCRF to reflect this situation.

Recurrent AEs are events that are recovered during the assessment period, but reoccur afterwards. Each recurrence must be recorded separately on the AE page of eCRF.

7.1.3 AEs Related to Examinations and Laboratory Tests

Abnormal results from examinations and laboratory tests (such as hematology, blood biochemistry, urinalysis, ECG, physical examination, vital signs, etc.) at screening are regarded as pre-existing condition before the ICF is signed and do not need to be recorded as an AE. Clinically significant abnormality at screening can be recorded as relevant medical history according to the Investigator's judgement. The Investigator is responsible for assessing all abnormal results of examinations and laboratory tests for clinical significance. Any abnormality meeting the following criteria is considered as clinically significant and should be reported as an AE:

- The abnormality that suggests a disease and/or organ toxicity that is new or has worsened from baseline.
- The abnormality that needs the investigational product dosage and usage to be adjusted, e.g., dose change, drug discontinuation, etc.
- The abnormality that requires additional active intervention, e.g. increase or modification of the concomitant medications, close observation, more frequent follow-up assessments, or further diagnostic examination, etc.

If the laboratory abnormality of clinical significance is a manifestation of disease (e.g. alkaline phosphatase and total bilirubin increase with cholecystitis), then only the diagnosis (i.e., cholecystitis) will be recorded as an AE. If laboratory abnormality of clinical significance is not a manifestation of disease, then the abnormality will be recorded as an AE. Appropriate description will be used to record if the test results are lower or higher than normal range (e.g. "serum potassium increase" rather than "abnormal serum potassium"). If the result meets diagnostic criteria, the clinical diagnosis will be recorded as an AE. For instance, when serum potassium increases to 7.0 mmol/L, it can be recorded as "hyperpotassemia".

7.2 Recording of AEs

7.2.1 Terms of AEs

All AEs reported by subjects or found by the Investigator are recorded and described on eCRF in accurate and standard medical terminology. The diagnosis is recommended to be used as the term for the AE. If no diagnosis, the symptoms, signs, or laboratory abnormality can be reported as the term of the AE. When a diagnosis is determined afterwards, the record can be updated accordingly.

7.2.2 Recording of AE Time and Collection Period for AE

The Investigator should try to collect the start and end dates of each AE. Correct records of AE start time are helpful in assessing the temporal relationship between AE and drug administration. Usually, the AE start date should be the date of symptom onset. If the AE is aggravated from the baseline medical conditions, the AE start date is the date of condition aggravated. If the AE is newly happened in the study and progressed to SAE, the SAE start date should still be the AE start date.

In this study, AEs (except for SAEs) are collected from the first dose of study medication in run-in period to the last visit.

The SAEs are collected from the first informed consent form is signed to the last visit.

7.2.3 AE Variables

The following variables are collected for each AE:

- Term (verbatim)
- Start and end dates/time
- Severity
- Serious adverse event or not
- The SAE criteria
- Action taken for study medication
- Outcome
- Assessment made by the Investigator on the causal relationship to study medication
- Hospitalization date (if applicable)
- Discharge date (if applicable)
- Cause of death (if applicable)
- Date of death (if applicable)

- Autopsy (if applicable).

7.2.4 AE Grading

AEs are graded by severity as mild, moderate and severe:

- **Mild**: appearance of signs or symptoms, but can be easily tolerated.
- **Moderate**: obvious discomfort interfering normal daily activities.
- **Severe**: incapacitating, and can't perform normal daily activities.

7.2.5 AE Outcome

The outcomes are described as follows:

- Recovered/resolved: the subject returns to baseline state.
- Recovering/resolving: the event hasn't been resolved completely, but the subject is improving.
- Not recovered/not resolved: the event is ongoing, e.g., irreversible congenital anomaly.
- Recovered/resolved with sequelae: only if the subject suffers from lifetime sequelae, e.g., blindness caused by diabetes, and the hemiplegia after a stroke.
- Fatal: the death date is the date for event ending.
- Unknown: the Investigator can't obtain the outcomes of the AE, e.g. the subject is lost to follow-up.

If an AE is assessed as "recovered/resolved", "recovered/resolved with sequelae", or "fatal", the AE ending date must be recorded.

The Investigator must follow up all AEs. When the study is completed, any AEs that are unresolved or don't have outcomes should be followed up by the Investigator until the events are resolved or considered stable, which don't have to be recorded further on eCRF (e.g., after database lock). The follow-up information on SAEs that occurs after database lock needs to be reported to Clinical Drug Safety department of the Sponsor. The contact information is:

Email: drugsafety@huamedicine.com. Fax: 021-58863272. Mobile: +86 156 1839 6573.

7.2.6 Causality Assessment

The Investigator should assess the causal relationship between the AE and the study medications

through medical and scientific judgement. The following criteria can be referred to:

1. Related. The AE occurrence follows a reasonable temporal relationship with administration of the study medication. The AE is consistent with the known profile of the study medication. The event is improved when the dose of study medication is decreased or stopped. The event reoccurs when the study treatment is restarted. The AE can't be explained by subjects' medical condition or alternative treatment.

2. Possibly related. The AE occurrence follows a reasonable temporal relationship with administration of the study medication. The AE is consistent with the known profile of the study medication. The AE alleviates or becomes not obvious after reducing the dosage or stopping the study medication. The subjects' medical condition or alternative treatment may lead to the event.

3. Unlikely related. The temporal relationship between the AE occurrence and administration of the study medication is not clear. The AE may not be consistent with the known profile of the study medication. The subjects' medical condition or alternative treatments may lead to the event.

4. Not related. There is no reasonable temporal relationship between the AE occurrence and administration of the study medication. The AE is not consistent with the known profile of the study medication. The subjects' medical condition or alternative treatments may lead to the event. The AE improves or disappears when the disease condition improves or alternative treatment is stopped.

7.3 Serious Adverse Event Reporting

Once a SAE occurs in the study, the Investigator must immediately take appropriate measures for treatment and report the SAE to the relevant drug administration departments, health administration departments, ethic committees and the Sponsor within 24 hours after awareness. The Investigator needs to ensure the SAE information be recorded on eCRF timely.

When completing the SAE report form (CFDA version), the Investigator must also collect the requested SAE information requested by the Sponsor. Refer to the Investigator Site File for the details of SAE reporting.

7.4 Hypoglycemia

The Investigator must correctly instruct subjects to conduct the SMBG (refer to Appendix 4 procedures for SMBG) to identify and manage hypoglycemia. Subjects should measure their fasting and postprandial fingertip blood glucose at least twice a week, and record in subject diaries. Any symptoms of hypoglycemia should be recorded in subject diaries. The subjects should be instructed to measure blood glucose when hypoglycemia symptoms appear, and also to carry carbohydrates that can be easily taken for hypoglycemia management.

Hypoglycemia event should be recorded on the hypoglycemia evaluation page of eCRF. Detailed information listed below must be recorded for each hypoglycemia occurrence: start date and time, end date and time, blood glucose values, symptom description, action taken, severe hypoglycemia or not, resolved or not, precipitating factors, causal relationship with the study medication, etc.

Refer to Section 4.5.1.1 of the protocol for the hypoglycemia assessment and classification criteria.

7.5 Hy's Law

The Hy's Law is used in this study to monitor any possible drug-induced liver injury. The Hy's Law[18] usually means that AST or ALT> 3×ULN and TBiL> 2×ULN, non-biliary increase (usually alkaline phosphatase < 2×ULN) without any other diseases for explanation of the increase. If ALT or AST is > 3×ULN, and TBiL is > 2×ULN without any other previous diseases for explanation of the increase, no matter whether alkaline phosphatase (ALP) increases or not, the Investigator must inform the subject of the central laboratory reports within 48 hours after awareness and ask the subject to return to the study site for central laboratory re-evaluation (if necessary, re-examinations at local laboratories can be taken). The Investigator conducts re-evaluation and closely monitors or follows-up with the subjects, or stops the study medications according to the Section 4.5.3 Subject withdrawal due to abnormal liver function and Appendix 6 Measures to be Taken for Abnormal Liver Functions in the protocol. The Investigator evaluates the etiology and conducts every essential examination to rule out drug-induced liver injury. If the Investigator confirms the event meets Hy's Law, the event needs to be reported as an SAE.

7.6 Post-study AE Reporting

At any time after the study ends, if the Investigator is informed of any AE which is considered possibly related to the study medication, the Investigator needs to promptly report the event to the Clinical Drug Safety department of the Sponsor. The follow-up information of the SAEs after database lock also needs to be reported to the Clinical Drug Safety department of the Sponsor. The contact information is: Email: drugsafety@huamedicine.com. Fax: 021-58863272. Mobile: 156 1839 6573.

7.7 Lack of Efficacy or Aggravation of T2DM

The events that are apparently consistent with expected progression of T2DM (e.g., hyperglycemia) do not need to be recorded as AEs. These data is used for efficacy assessment (parameters for glycemic control).

The protocol defines the withdrawal criteria relevant to poor glycemic control/hyperglycemia (see Section 4.5.2 withdrawal criteria due to hyperglycemia and Appendix 4 SMBG Procedures). The Investigator must warrant no other causes for poor glycemic control except for lack of

efficacy (e.g. concurrent acute inflammations/infections, and concomitant treatments, etc.). If the disease aggravation is unexpected, it should be reported as an AE. The Investigator is suggested to give the subjects relevant examinations to find out the reason for disease aggravation and record the reason on eCRF.

7.8 Laboratory Safety Assessments

Blood and urine samples are collected for laboratory assessments. The collecting date and time are recorded on the laboratory tests application form. The samples are processed in the central laboratory and the results are reported to study sites within 72 hours.

All subjects are suggested to go to each visit in fasting state between 7:00 am and 10:00 am. The subjects should be informed to avoid heavy physical activities and intake of high fat and high calorie food one day before the visit. Food and beverage except for water are prohibited within 8 hours before the visit. Also, the subjects are informed of not taking the study medications by themselves in the morning of the visit day. Other permitted medications can only be administered with water.

All samples are collected by authorized study staffs, and processed per manuals in the central laboratory. The latest laboratory reference range is provided to study sites during the study. The laboratory results are compared with the reference range values. Results exceeding the normal range are marked. The Investigator evaluates the abnormal results and decides whether they are clinically significant or not.

See Section 7.1.3 for how to record and report AEs relevant to laboratory tests.

The following laboratory safety parameters are measured:

- Hematology: hemoglobin, hematocrit, erythrocytes count, leukocytes count, neutrophils, basophils, eosinophils, monocytes, lymphocytes, and platelet count
- Urinalysis: pH, specific gravity, protein, glucose, erythrocytes, leukocytes, and ketone bodies
- Blood biochemistry: glucose, total bilirubin, ALT, AST, gamma-glutamyl transferase, alkaline phosphatase, albumin, total protein, lactate dehydrogenase, urea nitrogen, creatinine, uric acid, sodium, potassium, chloride, calcium, creatine kinase, creatine kinase isoenzyme, total cholesterol, high-density lipoprotein cholesterol, low-density lipoprotein cholesterol, and triglyceride.

All methods, instruments, and quality control requirements for laboratory tests are described in detail in the manual of central laboratory.

7.9 Physical Examinations

Full physical examination is conducted at each visit except Visit 2, Visit 3 and Visit 16. The physical examination includes a check on: general appearance, head and neck, chest (heart and lung), abdomen, and limbs.

Any finding from physical examination at subsequent study visits is compared with the results at screening visit and is evaluated for clinical significance. See Section 7.1.3 AEs Related to Examinations and Laboratory Tests for how to record and report AEs related to examinations.

7.10 12-lead ECG

A 12-lead ECG examination is conducted at each visit except Visit 2 and Visit 16. The Investigator evaluates the ECG results and record the results on eCRF as “normal” or “abnormal with/without clinical significance”. See Section 7.1.3 AEs Related to Examinations and Laboratory Tests for how to record and report AEs related to laboratory examinations.

7.11 Vital Signs

Vital signs (blood pressure, pulse rate, respiratory rate and body temperature) are measured according to study protocol. Height and body weight are included as part of vital sign assessments.

The subjects should rest for at least 5 minutes before vital sign assessments. Throughout the study, all efforts are made to measure pulse rate and blood pressure from the same arm. The arm used for measurement is documented in the original records.

Pulse rate

Palpation on the radial artery is suggested to last for at least 30 seconds to calculate the number of beats per minute, which is recorded on eCRF. If necessary, palpation is on bilateral radial artery simultaneously to check the symmetry. It is recommended to be marked on eCRF if there is any asymmetry.

Body temperature

A thermometer is placed at deep axillary region of the subjects. The subjects are told to hold the thermometer tightly and read the temperature 10 minutes later. Be noted that there should not be heating or cooling objects at the axillary region and sweat at the axillary region should be swiped.

Respiratory rate

The subjects are observed for breathing rhythm and breathing frequency per minute is recorded.

Blood pressure

A mercury sphygmomanometer is used for measuring blood pressure. The subject should be in

a quiet place with his feet flat on the floor back against the chair. Cigarettes and coffee is prohibited and bladder is emptied within 30 minutes before the assessment. The subject's upper arm is exposed and stretched slightly with the elbow at the same height with heart. The cuff is closely and evenly wrapped around upper arm with its lower edge at about 2.5 cm above the antecubital fossa and the center of cuff at the surface of brachial artery. After touching the brachial artery, the examiner puts the stethoscope's bell over the brachial artery for auscultation. Then the cuff is inflated while auscultation. After the sound of brachial artery pulse disappears, the cuff continues to be inflated until the reading values increase for additional 30 mmHg. Then the cuff is deflated gradually (2–6 mmHg/s). Eyes are kept at the same level with the mercury gauge while the mercury column is descending, and blood pressure is read according to auscultation. Blood pressure is measured at least twice with two-minute interval and the average of the two measurements is calculated. If the difference of the two measurements in systolic or diastolic blood pressure is larger than 5 mmHg, a third measurement is done after 2 minutes and the average of 3 measurements is calculated.

Height and Weight

Shoes and thick clothes should be taken off while height and body weight are being measured. The brand and model of the scales used for body weight measurement in each site should be kept the same throughout the study. BMI=Body Weight (kg)/Height $(m)^2$.

7.12 Pregnancy

The subject who becomes pregnant while receiving the study medication must immediately discontinue the study medication. Pregnancy itself is not considered as an AE, but it must be reported on the pregnancy form and submitted to the Sponsor as special safety information. The reporting time limit is the same as SAE reporting. All pregnancies must be followed up until the pregnancy outcome is obtained (e.g. spontaneous abortion, selective termination, ectopic pregnancy, full term birth, congenital anomaly). In addition, if a male subject's partner is pregnant during the study, it is also reported to the Sponsor and followed up until pregnancy completion.

Congenital anomaly, birth defects and spontaneous abortion should be reported as SAEs. Artificial abortion without complications do not need to be recorded as AEs. The pregnancy outcomes after database lock should be reported to the clinical drug safety department of the Sponsor. The contact information is: email: drugsafety@huamedicine.com, fax: 021-58863272, mobile: 156 1839 6573. Pregnancy report form refer to ISF.

7.13 Overdose

Overdose is defined as the situation when a subject is given excessive study medication, or the subject himself /herself takes excessive study medication intentionally or accidentally and beyond the dosage defined in study protocol. Overdose is recorded on the medication dosing page of eCRF. If overdose is associated with any AE, it is documented on AE page of eCRF as

well. If overdose is associated with any SAE, it needs to be reported according to the procedures in Section 7.3 of this protocol.

8 Data Management

8.1 Data Traceability, Completion, and Transfer of eCRF

Data management of the study is conducted by the data management department of dMed Biopharmaceutical Company Ltd. to assure data integrity, completeness, privacy, and traceability.

Data entry by site Investigator or the delegates must ensure the integrity and accuracy. And any correction and/or addition should be instructed to site personnel. Any correction must comply with the eCRF completion instructions.

8.2 Database Design

Database is designed by the data management department of dMed Biopharmaceutical Company Ltd. per requirements of the “Code of Federal Regulations Title 21, Part 11 (21 CRF Part 11)”. Database should be able to manage the traceability of system login, data entry, data update, and data deletion. Database design should follow the CDISC standards.

8.3 Data Entry

The Investigators and delegates are responsible for entering study data in eCRF. Required EDC training must be provided and completed prior to study initiation to ensure mastery of the EDC system and eCRF layout. Data entered into EDC must be validated to ensure data completeness and accuracy.

8.4 Data Validation

Data Validation includes programmed edit check per study design and manual review. Any discrepancy occurred during data validation must be clarified by Investigator and corrected timely.

8.5 Blinded Data Review and Unblinding

The study should be unblinded at the completion of the double-blind period for all subjects. Suggested by ICH E9 Biostatistical Principles for Clinical Trials, blinded data review should be conducted before unblinding to achieve the following two purposes:

- Determine population set during blinded data review
- Review blinded study data

It should be assured that the database is locked before unblinding.

Emergency Unblinding

Emergency unblinding is only applicable to emergency situations. The randomization code can only be unblinded under emergency medical conditions (e.g., treatment can only be given when the actual study treatment group of this subject is known). The investigator can discuss the potential unblinding case with the Sponsor and use the IWRS system (Medidata's Balance) to unblind the randomization code, which tells the actual treatment group that subject is randomly assigned to. If randomization code is unblinded, the time, date, and reason of unblinding must be recorded in the system, and signed off by the individual who performs the unblinding. The Sponsor and Medidata staffs shall be notified immediately.

If any unexpected SAE occurs or any suspicious causality between study drug and SAE, the case should be reported immediately to regulatory agencies. Hua Medicine reserves the right to unblind the randomization code or not for such cases.

Monitor must check the blinding status of any subject at each scheduled visit to assure the blind status of each subject is intact, and/or record is complete for each emergency unblinding case.

8.6 Database Lock

Two stages are planned for database lock in this study.

The first database lock is scheduled at the completion of the double-blind treatment for all subjects. After the blinded data review meeting, the principle Investigator, Sponsor, and biostatistician must sign off all related documents before the database lock (DBL). DBL is limited to the double-blind treatment data, but not the data in the open-label treatment period. No update is permitted to locked data in principle. Errors found after DBL should be evaluated to determine whether database unlock is necessary for data correction. Documentation must be recorded if database unlock is required.

The second database lock is scheduled at the completion of the open-label treatment. A regular data review meeting is required before the final DBL. The Investigator, Sponsor, and biostatistician should sign off relevant documents before DBL. In principle, no update is permitted to locked data. Errors found after database lock should be evaluated to determine whether database unlock is necessary for data correction. Documentation must be recorded if database unlock is required.

9 Statistical Analysis

9.1 Sample Size Determination

This study plans to recruit 750 subjects. Considering possible drop-out of the subjects, a 20% increase of the total subjects, but no more than 938 subjects, will be enrolled. These subjects

are randomized at a ratio of 1:1 into the treatment group (HMS5552 75mg BID + stable dose of metformin) or control group (placebo BID + stable dose of metformin), i.e. no more than 469 subjects and 469 subjects are randomized to the treatment group and control group.

Based on the planned sample size and the randomization ratio (750 subjects, treatment group: control group = 1:1, the study has 95.4% power to detect the treatment group is superior to the placebo group with the assumption of type I error (alpha) at 0.05 (two-sided), within-group standard deviation for the primary efficacy endpoint as 1.5% and the treatment difference (treatment group vs control group) as -0.4%.

9.2 Analysis Population Sets

Full analysis set (FAS): Based on the intention-to-treat (ITT) analysis principle, the FAS consists of all randomized subjects who receive at least one study dose and have at least one measure of the primary efficacy endpoint post randomization.

Per-protocol set (PPS): The PPS is a subset of FAS consisting of all subjects who have no major protocol deviations. The subjects in PPS are discussed and determined in the blinded review meeting prior to the database lock.

Safety set (SS): The SS comprises of all randomized subjects who receive at least one study dose.

The FAS is considered as the primary efficacy analysis, whereas safety assessment is performed on SS.

9.3 Statistical Methods

After all randomized subjects complete the 24-week double-blind period, the first stage database lock is performed. The study is unblinded for statistical analysis. When all subjects completed the 28-week open-label treatment period, the second database lock and statistical analysis is performed. The details of statistical analysis methods and description are provided in the separate Statistical Analysis Plan. The biostatisticians should formulate the Statistical Analysis Plan according to the clinical study protocol, should record in details the changes that deviate from the planned analysis in the protocol, and refine the final document before database lock and unblinding.

Statistical analysis will be performed using the statistical analysis software SAS version 9.2 or above. The significant level for between-group comparison is set as 0.05 (two-sided).

9.3.1 Demographics and Baseline Characteristics

The demographics is summarized for all randomized subjects (double-blind period) and those who received open-label treatment (open-label period). Summary tables or figures are used to

describe the baseline characteristics between the two treatment groups. The robustness of randomization is justified by the descriptive statistics, but no statistical testing is performed on baseline characteristics.

The number and percentage of subjects who are screened, randomized (double-blind period), and received open-label treatment (open-label period) is summarized separately by study period. The primary reasons of screen failure, randomization failure and withdrawal from the study is summarized by treatment group.

For continuous variables, the descriptive statistics such as sample size, mean, standard deviation (SD), median, and range (minimum-maximum) are provided. For categorical variables, the number and percentage of subjects for each category are calculated.

The baseline characteristics are summarized for all subjects who are randomized (completed Visit 4 in double-blind period) and receive open-label treatment (completed Visit 10 in open-label period). Both the FAS and PPS populations are used for summary of baseline characteristics.

9.3.2 Efficacy Analysis for Primary Endpoint

The primary efficacy endpoint is the change of HbA1c from baseline at the end of double -blind period (at Week 24). The mixed model for repeated measures (MMRM) is used for the primary endpoint analysis. The model includes groups, visits, the interaction between groups and visit, sites, disease duration and baseline HbA1c value. Treatment difference of the mean HbA1c change from baseline at each visit is estimated by MMRM. An unstructured variance-covariance matrix is preferred in the primary model.

The adjusted least-squares mean estimate and 95% confidence interval (CI) of treatment difference (treatment group-placebo group) in the primary efficacy endpoint is calculated. The following hypotheses are tested:

- Null hypothesis: at the end of double-blind treatment (Week 24), the HbA1c reduction from baseline in the two groups has no difference.
- Alternative hypothesis: at the end of double-blind treatment (Week 24), the HbA1c reduction from baseline in the two groups has significant difference.

The significant level of the between-group comparison is 0.05 (two-sided). If p value is smaller than 0.05, it is considered that the between-group difference is statistically significant. If the upper limit of the 95% CI is smaller than 0, it is considered that HMS5552 is superior to the placebo in treatment effect.

9.3.3 Efficacy Analysis for Secondary Endpoints

The secondary efficacy endpoints include changes from baseline at the end of double-blind

period (at Week 24) of 2h-PPG, and FPG. The same analysis method for the primary efficacy endpoint is used, i.e. MMRM. The adjusted least-squares mean difference between HMS5552 and placebo, and its 95% CI is estimated by MMRM.

HbA1c response rate is calculated based on the data imputed by last observation carried forward (LOCF). The HbA1c response rate, i.e. number and percentage of subjects whose HbA1c is less than 7.0% at the end of double-blind treatment period (at Week 24) are summarized. The HbA1c response rate and 95% CI for each group are estimated using the logistic regression method.

The measurement and changes of HbA1c, FPG and 2h-PPG from the end of double-blind period (Week 24) to the end of study (Week 52) are summarized by two groups: one group is for subjects who are randomized into HMS5552 treatment group, and the other group is for subjects who are randomized into placebo group while switched to HMS5552 open-label treatment after double blind period (Week 24). The measurements at the end of double-blind period (Week 24) are used as baseline for the summary of the measurements and change from baseline at each visit in the open-label period.

9.3.4 Safety Analysis

Descriptive analysis is performed on SS. Safety data are summarized and evaluated for the following two stages: 1) Stage 1: when the subjects completed the double-blind period, and 2) Stage 2: when the whole study is completed.

All AEs are coded using the MedDRA dictionary. The incidences and severity of AEs, and SAEs, etc. are summarized and compared between two treatment groups. The detailed description of the treatment emergent SAE and study discontinuation due to AE is listed.

The incidence of hypoglycemic events is compared between treatment groups. The incidence ratio and 95% CI of each treatment group, as well as the incidence difference between treatment groups and its 95% CI are calculated.

The change from baseline of laboratory tests, physical examinations, vital signs, and 12-lead ECG measurements at each visit are summarized. Each index before and after treatment of normal/abnormal changes is also summarized.

9.3.5 Biomarker Analysis

This section is not applicable.

9.3.6 Sensitivity Analysis

Sensitivity analysis is applicable to the primary efficacy endpoint.

The LOCF approach is performed to impute missing primary efficacy endpoint. For subjects with early withdrawal, the last available post-treatment measurement is carried forward. The

analysis of covariance (ANCOVA) model is used for analyzing the imputed data. The ANCOVA model includes baseline HbA1c value, disease duration, sites and groups as covariate, with or without the interactions between center and group. If p value of the between-group comparison is smaller than 0.05, it is considered that the between-group difference is statistically significant. If the upper limit of the 95% CI is smaller than 0, it is considered that HMS5552 is superior to placebo in treatment effect.

9.3.7 Interim Analysis

No interim analysis is planned during the double-blind period. Statistical analysis is planned for two stages: 1) Stage 1 is for double-blind period, and 2) Stage 2 is for open-label period. After the completion of the double-blind period, the eligible subjects will enter the open-label period. No alpha sparing or independent data monitoring committee is required in this study.

10 Population PK/PD （PopPK/PD）Analysis

The PopPK/PD model for HMS5552 in subjects with T2DM is updated by using the NONMEM/PIRANA/R software based on the current HMS5552 PopPK/PD model and PK/PD data in Chinese subjects with T2DM after HMS5552 treatment. The quantitative relationship between HMS5552 concentration and HbA1c level is established. Systemic quantitative analysis is conducted on the influences of various factors on HMS5552 PK/PD characteristics, e.g., demographic characteristics, disease course, and concomitant medications. The specific methods and information for analysis of PopPK/PD model are elaborated in the population PK/PD analysis plan.

11 Trial Management

11.1 Declaration

This study is conducted in full compliance with the Declaration of Helsinki, China Good Clinical Practice (GCP), China clinical study laws and regulations.

11.2 Ethics Committee

In accordance with China regulatory requirements, all study related documents are provided to ethics committee (EC) by the Investigator.

Before the study drugs are shipped to the Investigator, the copy of EC approval and the list of required documents must be provided to the Sponsor. The documents approved by EC should include a list of EC members who have taken part in the discussion for document approval.

Upon receipt of the EC approval, the Sponsor/CRO should submit the study related documents to the relevant drug evaluation authority for record.

Approval of the study from EC and drug administration department must be obtained before the study starts.

Protocol amendments need to be submitted to EC for approval. The health bureau is also notified per local requirements.

During the study, the Investigator should notify EC of any SAEs or unexpected AEs that are related to the safety of clinical study and may affect patient safety and study implementation.

The possible benefits and risks for subjects are fully described in the ICF.

11.3 Source Document Verification

The Investigator must handle all data obtained from clinical study properly to protect the rights and privacy for the subjects participating in the study. The Investigator and the medical institution which he/she works in allow the Sponsor to monitor or audit the study. Health administration and drug administration departments have the right to inspect the study and review data/source documents.

11.4 Quality Control and Quality Assurance

This study can be audited by Sponsor or Sponsored authorized independent personnel for quality assurance, or by drug approval authority. The quality assurance monitor or inspector from drug approval authority can review all study related information and data, including but not limited to medical records, study documents and letters, study required facilities and equipment, study samples and materials, etc.

The audit on clinical trial by Sponsor is a part of quality assurance, which is different from the routine clinical trials monitoring, also different from the quality control and management from a Sponsor's functional department. The objectives of this audit by Sponsor are: to evaluate the implementation of clinical trial, and the compliance with the study protocol, SOP, related law and regulations from Drug supervision and management authorities.

11.5 Subject Informed Consent

The Investigator is responsible for explaining to each subject about the study objectives, study methods, benefits, and potential risks. ICF signed by subjects must be obtained before any study related procedures. The informed consent should be presented both orally and in writing. The ICF also must be dated and signed by subjects. For subjects who cannot sign by themselves for any reason, the ICF must be signed by their parents, legal guardians or legal representatives. The signed ICF is kept by the subjects.

By signing the ICF, subjects/patients must agree to allow the Sponsor, regulatory authorities, auditors and/or monitors to verify the related original data obtained from the study. All the

auditing personnel must follow the confidentiality agreement.

11.6 Protocol Amendment

After the protocol is finalized, any protocol amendments must be documented in details and the records for protocol changes need to be signed at least by the Investigator and the Sponsor. The version numbers and dates need to be indicated.

All protocol amendments must be submitted to EC. Written approval must be obtained from EC for significant changes. Local regulatory authorities should also be notified with renewed profiles. All documents should be submitted to the Sponsor. All the procedures should be completed and follow the previous edition before the updated profile takes effect.

11.7 Monitoring

On-site monitoring is performed by the CRAs from the Sponsor or Sponsor authorized CROs (the CROs' SOPs, the Sponsor's SOPs or the written agreement agreed by both parties should be followed). The monitoring visits should be conducted regularly throughout the study.

Monitors authorized by the Sponsor are permitted to review the source data relevant to the study and will verify the data on eCRFs and in other study documents per SOP and GCP requirements, ensuring data integrity, accuracy and consistency with the source documents.

All source documents, data, records, laboratory data/information, eCRF and medical test results must be accessible to monitors, inspectors and the drug administration departments for review at any time.

11.8 Confidentiality Agreement and Patient Privacy

The Investigator must keep the information related to the Sponsor and investigational drug confidential, which is provided or disclosed due to the cooperation in the study. The information can only be used with authorization from the Sponsor.

The commitment to confidentiality is independent, valid, and persistent.

The Investigator should ensure that the confidential information is not disclosed to third parties and is only used within the agreed scope. The confidential information may be obtained from Hua Medicine and its products, or provided or disclosed due to the contractual relationship.

The clinical study staffs must protect subjects' privacy. Only the subject number can be used for subject's identification in all documents submitted to the Sponsor. Subjects' names and hospitalization IDs cannot be used. The Investigator must properly preserve the relevant documents related to subjects' names, addresses and the screening IDs/randomization IDs. All subjects' information should be securely preserved by the Investigator in accordance with GCP regulations.

12 Publication

As the Sponsor, Hua Medicine (Shanghai) Ltd. has the exclusive ownership for the publications in this study. Authors and publications reflect the collaboration among multiple investigators, study sites and staffs from Hua Medicine (Shanghai) Ltd. Authors should be determined before preparing the manuscript. Unless approved by Hua Medicine (Shanghai) Ltd., any person is not allowed to have any publication before the final clinical study report is completed. Hua Medicine (Shanghai) Ltd. has the right to make final decision on all manuscripts and publications.

13 Documents Archiving

In accordance with relevant regulations, the Investigator should maintain all the medical source documents properly. All study documents should be kept for at least 5 years after the drug is marketed. The Sponsor is responsible for informing the Investigator/study sites when the study documents do not need to be kept any more.

14 Appendix

Appendix 1 Diagnosis of Diabetes Mellitus

(From China Guideline for Type II Diabetes prevention and treatment, Version 2013, CDS[11])

Clinical diagnosis for diabetes mellitus should be based on the blood test results of venous plasma glucose rather than capillary blood glucose. If not specified, the blood glucose mentioned in this article refers to the venous plasma glucose.

The normal range of blood glucose and the diagnosis cutoff point for glucose metabolism abnormality are mainly confirmed by the relationship between the value of blood glucose and the risk of specific chronic diabetic complications (diabetic retinopathy) and diabetes mellitus occurrence. Currently, the commonly used diagnostic criteria and classification are the WHO standards (1999) and the ADA (American Diabetes Association) standards (2003).

This guideline adopts WHO Standards (1999) for diabetes diagnosis, classification of glucose metabolism state, and the classification system of diabetes mellitus (Table 1 and 2). Fasting Plasma Glucose (FPG) or the venous plasma glucose value 2 hours after 75g Oral Glucose Tolerance Test (OGTT) alone can be used for epidemiological investigation or population screening. However, based on the data in our country, the missed diagnosis rate is high if only FPG is tested. Ideally, both FPG and venous plasma glucose 2 hours after OGTT need to be tested. Blood glucose at other time points after OGTT are not used as diagnostic criteria.

The population with impaired glucose regulation is suggested to take the OGTT to reduce the missed diagnosis rate.

Table 1 Classification of Glucose Metabolism State (WHO 1999)

Classification of Glucose Metabolism	Venous Plasma Glucose (mmol/L)	
	Fasting Plasma Glucose	2 hours Postload Plasma Glucose
Normal Plasma Glucose	< 6.1	< 7.8
Impaired Fasting Glucose (IFG)	6.1 ~ < 7.0	< 7.8
Impaired Glucose Tolerance (IGT)	< 7.0	7.8 ~ < 11.1
Diabetes Mellitus	≥ 7.0	≥ 11.1

Note: both IFG and IGT are called as impaired glucose regulation, also known as prediabetic state.

Table 2 Diagnostic Criteria for Diabetes Mellitus

Diagnostic Criteria	Venous Plasma Glucose (mmol/L)
(1) Typical diabetic symptoms (polydipsia, polyuria, polyphagia, and weight loss), with random plasma glucose test	≥ 11.1
Or, *with*	
(2) Fasting plasma glucose test	≥ 7.0
Or, *with*	
(3) Blood glucose test 2 hours postload glucose<br>People without diabetic symptoms needs re-test on different day	≥ 11.1

Note: The fasting status means no calories intake for at least 8 hours. random plasma glucose means plasma glucose tested at any time regardless of the last meal time and can’t be used for IFG and IGT diagnosis.

Appendix 2 New York Heart Association (NYHA) Classification

NYHA Classification:

I. Cardiac patients with no limitation in daily activity. ordinary physical activity will not lead to excessive fatigue, palpitations, dyspnea or angina.

II. Cardiac patients with slight limitation in physical activity. Asymptomatic at rest, ordinary physical activity will cause excessive fatigue, palpitations, dyspnea or angina.

III. Cardiac patients with obviously limitation in physical activity. Asymptomatic at rest, while less-than-ordinary activity cause excessive fatigue, palpitations, dyspnea or angina.

IV. Cardiac patients who cannot stand for any physical activity. Symptoms of heart failure occurs even at rest, which is aggravated when undertaking physical activity.

Appendix 3 Procedure of Mixed Meal Tolerance Test (MMTT)

The MMTT is a standardized method to determine postprandial blood glucose levels. In this study, MMTT is used for assessing how HMS5552 affects postprandial blood glucose, insulin and C-peptide. (Note: All meals used for MMTT in this study are the instant noodles standard meal provided by Sponsor).

Subjects are requested to avoid any heavy physical activities and high fat and high calorie food on the night prior before MMTT visit. Drug administration for the basic drug metformin is kept consistent throughout the study.

On the day of MMTT visit, subjects should be in fasting status (suggest no food and beverage intake from 22:00 at previous night until blood sampling is completed. water is allowed and fasting status should be kept for at least 8 hours).

Totally four MMTTs will be conducted for the whole study, including one at Visit 4 before study drug administration, and once each at Visits 7, 10 and 15 after study drug administration (except 0-min MMTT).

Before the instant noodles standard meal, blood samples need to be collected at the timepoint 0 and then the instant noodles standard meal starts to be taken (timing starts from the first chewing of the noodles and the meal is finished within 10 minutes). Blood samples are collected at 30 minutes (±3 minutes are allowed), 120 minutes (±5 minutes are allowed) after the instant noodles standard meal. When blood samples are being taken, food, water, and strenuous exercise is not allowed until all samples for MMTT are collected. (Blood sampling at 30 minutes applies to the subjects who enroll after the Version1.1 profiles take effect)

If subjects withdraw from the study early and already discontinued study medications on the termination visit, then MMTT for the termination visit can be cancelled (other tests are the same as Visit 15).

Procedures for Taking the Instant Noodles Standard Meal

1. Adding seasoning: Take out the seasoning bag and add the seasoning to the noodles.

2. Cooking: Add approximately 550 ml boiling water (suggest to use immediately once the water is boiling) to the noodles. Cover the lid and start timing.

3. Stirring: After 2 to 2.5 minutes, open the lid and stir the noodles. Cover the lid again.

4. Noodles ready: Use chopsticks to stir the noodles well when they are cooked for 10 to 15 minutes.

5. Eating: Eat all the noodles within 10 minutes. The soup can be finished or left according to subjects' personal preference.

Appendix 4 Procedures for SMBG

At Visit 2, blood glucose meters with matching blood collection needles and test strips are provided to all subjects. Subjects are instructed for SMBG. Subjects monitor fingertip blood glucose levels (fingertip blood glucose tests for FPG or PPG are required to be conducted at least twice a week. the frequency is increased when hypoglycemia is suspected. All test time and results are recorded in subject diaries) at appropriate frequency decided by the Investigator (based on the assessment on the risks of subjects' blood glucose concentration).

Throughout the entire study (Visit 2 to Visit 16), to give subjects safety guidance and assess the withdrawn criteria timely, the Investigator should ask subjects to contact research centers immediately if the results of fingertip plasma glucose test meet the following criteria:

1. Hypoglycemia. Visit 2 (-Week 4) to Visit 15 (Week 52), at any time, fingertip plasma glucose value ≤ 3.9 mmol/L.

2. Hyperglycemia.

   Day 1 to before Week 12, fasting plasma glucose (central laboratory) ≥ 13.3 mmol/L.

   Week 12 to before Week 24, fasting plasma glucose (central laboratory) ≥ 11.1 mmol/L.

   Week 24 to Week 52, fasting plasma glucose (central laboratory) ≥ 11.1 mmol/L.

Blood glucose results are recorded by subjects in subject diaries. At each visit after Visit 2, subjects are required to take blood glucose meters and subject diaries to research centers for Investigator review.

Appendix 5 Estimated Glomerular Filtration Rate (eGFR) Measurement

Calculation of eGFR with MDRD equation[19]

1. $eGFR = 175 \times [S_{cr}]^{-1.154} \times [Age\ (Year)]^{-0.203} \times [\times 0.742 \text{ if female}]$

   Cr unit: mg/dL, Age unit: year

2. $eGFR = 175 \times [S_{cr} \times 0.01131]^{-1.154} \times [Age\ (Year)\ ]^{-0.203} \times [\times 0.742 \text{ if female}]$

   Cr unit: µmol/L, Age unit: year

Appendix 6 Rules for Managing Abnormal Liver Functions

The Investigator should be highly alert with increased liver biochemistry values during the study, and should ensure subjects' safety and assess the cause of abnormal liver function timely.

For abnormal liver biochemistry values occurring at different stages of the study, the below actions are suggested.

Screening and Run-in Periods

1. Subjects with active liver disease are screen failures.
2. Subjects with ALT or AST > 2.5×ULN or total TBiL > 1.5×ULN are screen failures.
3. If a subject has ALT or AST values higher than the upper limit of normal range but are ≤ 2.5×ULN, or TBiL higher than the upper limit of normal range but are ≤ 1.5×ULN, the Investigator is suggested to find out the cause for abnormal liver function parameters (the Checklist for Collecting Abnormal Liver Function Information in the Investigator's folder can be used). Subjects can continue the subsequent study only when the Investigator can ensure the abnormal liver function will not affect the safety assessment or subjects' completion of the entire study or study procedures.

Treatment Period

From Visit 4 to Visit 15 in this study

1. The Investigator can stop study medications based on subjects' actual condition if any of the following criteria is met (no need to wait for the retest results):
   - ALT or AST > 8 × ULN
   - ALT or AST > 5×ULN and lasts for ≥ 14 days
   - ALT or AST > 3×ULN and TBiL > 2×ULN
   - ALT or AST > 3×ULN with liver damage symptoms (e.g., fatigue, nausea, vomiting, right upper abdominal pain or tenderness, fever, rash) or eosinophilia increase.

The Investigator should immediately provide active intervention or treatment to subjects and identify the cause. If necessary, consultation with hematologists is conducted. The Investigator should closely follow-up with the subjects until the liver function is recovered or returns to baseline status. The Investigator should record the relevant AEs or SAEs timely.

2. If subjects have ALT or AST> 3×ULN but do not meet withdrawal criteria, the Investigator must ask the subjects to come back to the research center and retake the liver function tests at the central laboratory within 48 hours after receiving the test report (local laboratory tests can

be done when necessary). The Investigator should record the relevant AE or SAE timely, and find out the cause for abnormal liver functions, and give necessary interventions. The Investigator should closely follow-up with the subjects until the liver function is recovered or returns to baseline status. The subjects can continue to receive treatment according to the visit schedule in the study protocol if the Investigator considers appropriate.

3. If subjects have ALT or AST higher than the upper limit of normal range but is≤ 3×ULN, the Investigator evaluates the clinical significance of the abnormal values (see Section 7.1.3 Adverse Events Related to the Medical and Clinical Laboratory Examinations). If any AE criterion is met, an AE should be recorded. The Investigator needs to find out the cause for abnormal liver functions and give necessary interventions. The Investigator should closely follow-up with the subjects until the liver function is recovered or returns to baseline status. The subjects can continue to receive treatment according to the visit schedule in the study protocol if the Investigator considers appropriate.

Safety Observation Period

All study drugs are stopped in the safety observation period. All new occurrence of clinically significant abnormal liver function at the last visit should be reported as an AE (refer to Section 7). The Investigator should give necessary interventions and follow-up with the subjects until the liver function is recovered or returns to baseline status.

Clarify the cause of abnormal liver function (for reference)

The Investigator figures out causes of abnormal liver function based on subjects' actual medical situation or clinical experience. The Investigator can also refer to the following items for clarification (e.g. inquiry, physical examination or laboratory tests).

Medical history and related risk factors: e.g. short-term drinking history, drug abuse, acute drug overdose, blood transfusion history, unclean seafood intake, occupational toxic contact, exposure to jaundice or other high-risk population, familial liver disease history, etc..

Physical examination: if any hepatomegaly, jaundice and other symptoms/signs occur.

Concomitant medications: if any prescription or non-prescription drugs or herbs or health care products been used and may lead to liver injury before the occurrence of liver injury, e.g. acetaminophen, paracetamol, carbamazepine, chlorpromazine, chlorpropamide, steroid contraception drugs, erythromycin ethylsuccinate, non-urethane, halothane, ketoconazole, monoamine oxidase, nonsteroidal anti-inflammatory drugs, phenindione, phenylbutazone, phenytoin, sulfonamides and sodium valproate, etc..

Hematological examination results: e.g. prothrombin time (PT), hepatitis A, B, C, D, E virus related tests, anti-cytomegalovirus antibodies, EB virus antibodies, anti-smooth muscle antibodies, anti-mitochondria antibodies, anti-neutrophil cytoplasmic antibodies, etc.

Other tests: e.g. abdominal ultrasound, CT or MRI if needed.

If necessary, hepatologists are consulted.

Appendix 7 Recommendations for Managing Hypoglycemia

(for reference)

1. When suspected hypoglycemia symptoms appear (e.g. palpitation, anxiety, sweating, hunger etc, or altered mental status, cognitive impairment, convulsion, coma, etc.), blood glucose level should be measured immediately for a concise diagnosis. If blood glucose is difficult to measure, treatment for hypoglycemia is given temporarily.

2. Treatment for new occurrence of hypoglycemia

1) Subject with clear consciousness. Take glucose or sugary food as soon as possible (suggest taking 15 to 20 grams of sugary food orally, glucose is preferred).
   Caution: to avoid a delay in the sugar absorption, don't eat any foods immediately after taking sugars described above.
2) Subject with no consciousness or coma. Call 120 and send the subject to hospital immediately. Don't put food, liquid, or hand into the subject's mouth. Keep the subject's airway open. Recommended treatment: intravenous injection of 20 to 40 mL 50% glucose solution or intramuscular injection of 0.5 to 1.0 mg glucagon.

3. After the treatments mentioned above, blood glucose level is monitored every 15 minutes.

1) If plasma glucose level is still ≤ 3.9 mmol/L, oral intake of sugary food or glucose injection will be done once again.
2) If plasma glucose level is > 3.9 mmol/L but the time to next meal is more than 1 hour, food consists of starch or protein will be given after 10 to 15 minutes.
3) If plasma glucose level is still ≤ 3.0 mmol/L, intravenous injection of 60 mL 50% glucose solution will be given.

4. Once hypoglycemia has been corrected

1) Find out the cause for hypoglycemia and adjust the drug administration. For subjects with impaired consciousness, plasma glucose level in short-term does not need to be strictly controlled.
2) Pay attention to cardiovascular diseases induced by hypoglycemia.
3) Frequent SMBG is suggested.
4) Increase diabetic education and carry diabetes emergency cards.

If hypoglycemia has not been corrected,

1) Intravenous injection of 5 or 10% glucose, or combined with glucocorticoid.
2) Monitor blood glucose for at least for 24 to 48 hours after recovery of consciousness.

Appendix 8 Medical Nutrition Therapy for T2DM

(Chinese Guideline for the Prevention and Treatment of T2DM (2013 Edition), Chinese Diabetes Society[11])

Medical nutrition therapy is a special intervention approach in treating the nutritional problems in patients with diabetes mellitus, including personalized nutrition assessment, nutrition diagnosis, relevant nutritional intervention plan to be implemented and monitored in a certain period of time. Medical nutrition therapy is important in the prevention, treatment, self-management, and education for diabetes mellitus and relevant complications. By modifying nutrient structure, medical nutrition therapy is beneficial in blood glucose control and helps to maintain optimal body weight and prevent malnutrition.

General principles for nutrition therapy

Subjects with diabetes mellitus and prediabetes need to receive personalized medical nutrient treatment under the guidance of a registered dietitian who is familiar with diabetes treatment or an integrated management team (including diabetes educators). After nutrition condition of the patient is assessed, a reasonable target should be set for controlling the total energy intake, maintaining appropriate and balanced distribution of various nutrients, and achieving metabolic control goal. Personal food preferences are satisfied as much as possible. Moderate body weight loss, which can be maintained by physical exercises and behavior changes, is recommended for overweight or obese patients.

Objectives for medical nutrition therapy

To maintain reasonable body weight: the goal for overweight or obese patients is to achieve 5% to 10% weight loss within 3 to 6 months. Subjects of marasmus should restore and maintain optimal body weight through well-designed nutrition programs. With balanced diet, to achieve and maintain optimal blood glucose level and decrease HbA1c level. To reduce the risk factors for cardiovascular diseases, including the management of dyslipidemia and high blood pressure. To reduce insulin resistance and the load for islet beta cells.

Nutrients

Fat

The energy from fat should be no more than 30% of the total dietary energy. Intake of saturated fatty acids should be less than 7% of the total dietary energy. Intake of trans fatty acids should be reduced as much as possible. Monounsaturated fatty acids are good source of dietary fat and should account for 10 to 20% of total fat intake. Intake of polyunsaturated fatty acids should be less than 10% of total energy intake. Intake of food rich in n-3 rich fatty acids should be properly increased. The dietary cholesterol intake should be less than 300 mg/d.

Carbohydrate

Carbohydrate should account for 50 to 60% of the total dietary energy. The quantitation, assessment and measurement for dietary carbohydrates are key steps in blood glucose control. Foods of low glycemic index are beneficial to blood glucose control. Reasonable intake of sugar alcohol and non-nutritive sweeter is safe. However, fructose produced from excessive sucrose decomposition or adding excessive fructose can lead to increase triglyceride synthesis and body fat accumulation. Everyday meals need to be taken regularly to keep carbohydrate intake balanced every day.

Protein

For individual diabetes patient with normal renal function, protein intake is suggested to account for 10 to 15% of the total energy. The proportion of high-quality protein should be over 50%. Subjects with presence of proteinuria should restrict their protein intake to 0.8g/kg•day. Subjects with decreased glomerular filtration rate (GFR) should take low protein diet with the recommended protein intake of 0.6g/kg•day. To prevent protein malnutrition, compound alpha-keto acid can be used. Simple protein intake is unlikely to cause hyperglycemia but it may increase insulin secretion.

Drinking

Alcohol is not recommended for patients with diabetes mellitus. If subjects drink alcohol, the energy from alcohol should be calculated. Daily alcohol consumption for females shouldn't be more than 15 g. Daily alcohol consumption for males shouldn't be more than 25g (15g alcohol is equivalent to 450 ml beer, or 150 ml wine, or 50 ml low alcohol liquor). Alcohol drinking should be no more than twice a week. Attention should be paid to alcohol-induced hypoglycemia. Alcohol drinking in fasting status should be avoided. Individuals with risks for T2DM should restrict intake of sugary drinks.

Dietary fiber

Beans, fiber-rich cereals (≥ 5g fiber per serving), fruits, vegetables, and whole grains are good sources of dietary fiber. Increasing fiber intake is good for health. Diabetes patients are suggested to have recommended dietary fiber intake, i.e. 14g/1000 kcal per day.

Salt

Daily salt intake should be restricted to less than 6g/day. Patients with hypertension should further restrict their salt intake and food rich in salt, e.g., monosodium glutamate, soy sauce, processed food such as salted food, etc.

Micronutrient

Diabetic subjects are prone to deficiency of vitamin B, vitamin C, vitamin D and many micronutrients such as chromium, zinc, selenium, magnesium, iron, manganese. Micronutrient could be supplemented based on the nutritional assessment results. Patients with long drug history of metformin should be prevented from vitamin B12 deficiency.

Deficiency

Long-term intake of large quantities of antioxidants, such as vitamin E, vitamin C and carotene, is not suggested because their long-term safety remains to be verified.

Dietary pattern

Different dietary patterns, such as the Mediterranean diet, vegetarian diet, low carbohydrate diet, low fat and low energy diet, or high protein diet, are all beneficial to body weight control in short term. However, this should be done under the guidance of qualified healthcare professionals, and meanwhile, change of blood lipids and renal functions is monitored.

Exercise therapy for T2DM

Physical exercise plays an important role in the integrative management of patients with T2DM. Regular physical exercise can improve insulin sensitivity and glucose control, reduce cardiovascular risks, reduce body weight, and increase feeling of happiness. Physical exercise is also proved to be effective in primary prevention for the population with high risk of diabetes. Epidemiological studies show that regular physical exercise for more than 8 weeks helps to reduce the HbA1c by 0.66% in subjects with T2DM, and the mortality rate significantly reduced in subjects who adhere to regular physical exercise for 12 to 14 years.

When patients with T2DM are doing physical exercise, the following rules need to be followed. Exercise therapy should be carried out under the guidance of physician. Necessary assessments need to be conducted before physical exercise, especially the medical assessment on cardiopulmonary function and motor function (such as exercise stress test, etc.). Subjects are not permitted to do physical exercise in the following situations: fasting plasma glucose > 16.7 mmol/L. recurrent hypoglycemia or blood glucose fluctuations. diabetic ketoacidosis and other acute metabolic complications. complicated acute infection. proliferative retinopathy. severe nephropathy. severe cardiovascular and cerebrovascular diseases (unstable angina, severe arrhythmia, transient cerebral ischemic attack). Normal activities can be resumed gradually after the disease status becomes stable. Adult diabetic subjects need to do aerobic exercise of moderate intensity (50 to 70% of maximum heart rate, a little strenuous, heart rate and breathing rate increase without shortness) for at least 150 minutes every week (e.g. 30 minutes per day, 5 days per week).

Studies have shown that even short-time (such as 10 minutes, totally 30 minutes/day) physical exercise is beneficial. Moderate intensity activities include fast walking, tai chi, cycling, table tennis, badminton, and golf. Vigorous intensity activities include dancing, aerobics, jogging, swimming, and cycling to uphill. Subjects are suggested to have resistance training twice a week to improve their muscular strength and endurance, if they do not have any contraindications. The resistance exercise should be mild or moderate. The combination of resistance exercise and aerobic exercise can improve the metabolism more greatly. Exercise plan should be adapted to subjects' age, disease status and physical endurance and assessed and adjusted regularly. Keeping exercise diaries is helpful to improve the exercise compliance.

Developing healthy habits and active lifestyle, such as increasing daily physical activities and

reducing sedentary time, to integrate beneficial sports into daily life. To prevent hypoglycemia, subjects should be monitored for blood glucose before and after physical exercise. Diet and treatment is suggested to be adjusted temporarily after strenuous exercise.

Smoking Cessation

Smoking is harmful to health and is associated with higher risks of cancer, diabetic macrovascular diseases, diabetic microangiopathy, and early death. Studies have shown that for newly diagnosed T2DM patients, smoking cessation is helpful in improving metabolic parameters and decreasing blood pressure and albuminuria. All diabetic subjects are suggested to stop smoking or using other tobacco products. Subjects are evaluated for smoking and the nicotine-dependence status. Short consultation, smoking cessation hotline, and necessary medications are provided to subjects for smoking cessation.

15 References

[1] Yang W, Lu J, Weng J, et al., Prevalence of diabetes among men and women in China. The New England Journal of Medicine 2010；362(12):1090-1101.

[2] Jiamin Chi, Practice of Diabetology, Version 4, Jul2015.

[3] Chinese guidelines for the prevention and treatment of type 2 diabetes mellitus (2013 Edition), Chinese Diabetes Society. Chinese Journal of Diabetes 2014,7:447-498.

[4] Matschinsky FM. Assessing the potential of glucokinase activators in diabetes therapy. Nature Reviews Drug Discovery 2009, 8(5):399-416.

[5] Matschinsky FM, Zelent B, Zoliba N, et al., Glucokinase activators for diabetes therapy, Diabetes Care, 2011 (34), S236-S243.

[6] HMS5552 Investigator's Brochure, Hua Medicine (Shanghai) Ltd., 2017.03, version 3.0.

[7] Phase Ia clinical study report of single ascending dose of HMS5552 (a randomized, double-blind, placebo-controlled safety, tolerability, pharmacokinetics and pharmacodynamic study of administrating single ascending dose of HMS5552 in healthy adult volunteers, serial number: HMM0101), Hua Medicine (Shanghai) Ltd., 2014.05.

[8] Hongrong Xu, et al., Safety, tolerability, pharmacokinetics, and pharmacodynamics of novel GK activator HMS5552: results from a first-in-human single ascending dose study. *Drug Design, Development and Therapy*.09May2016.10:1619-26.

[9] Phase Ib clinical study report of multiple ascending doses of HMS5552 (a randomized, double-blind, placebo-controlled study to assess the safety, tolerability, pharmacokinetics, and pharmacodynamics of multiple ascending doses of HMS5552 in adult subjects with type 2 diabetes mellitus, serial number: HMM0102), Hua Medicine (Shanghai) Ltd., Nov2014

[10] Phase Ic clinical study report of HMS5552 (a single-center, randomized, open-label, 24-week treatment study to investigate safety, tolerability, pharmacokinetics and pharmacodynamics in type 2 diabetes mellitus (T2DM) subjects, serial number: HMM0103), Hua Medicine (Shanghai) Ltd., Mar2016

[11] Phase Id clinical study report of HMS5552 (a phase 1, open-label, sequential, multiple-dose, drug-drug interaction study of GK activator HMS5552 and Metformin in subjects with Type 2 Diabetes Mellitus (T2DM), Serial number: HMM0104), Hua Medicine (Shanghai) Ltd., May2016

[12] Phase II clinical study report of HMS5552 (a multi-center, randomized, double-blind, placebo-controlled, 12-week phase II study to evaluate the safety, tolerability, efficacy and population PK of HMS5552 in type 2 diabetic adult subjects, serial number: HMM0201), Hua Medicine

(Shanghai) Ltd., Mar2017

[13] Garber AJ, Abrahamson MJ,Barzilay JI,et al. American Association of Clinical Endocrinologists's comprehensive diabetes management algorithm 2013 consensus statement—executive summary. Endocr Pract, 2013, 19:536-557.

[14] American Diabetes Association. Standards of medical care in diabetes-2014.Diabetes Care,2014,37(Suppl 1): S14-S80.

[15] International Diabetes Federation. Global guideline for type 2 diabetes, 2012.

[16] Guidelines for clinical trials of drug and biologics for the treatment of diabetes, China Food and Drug Administration 15May2012 No [2012] 122

[17] Glucose concentrations of less than 3.0 mmol/L (54 mg/dL) should be reported in clinical trials: A joint position statement of the American Diabetes Association and the European Association for the Study of Diabetes. Diabetes Care 21Nov2016. [Epub ahead of print] PMID: 27872155.

[18] FDA guidance for industry drug-induced liver injury: premarketing clinical evaluation. July 2009 https://www.fda.gov/downloads/Drugs/GuidanceComplianceRegulatoryInformation/Guidances/UCM174090.pdf

[19] Miller WG. Estimating glomerular filtration rate. Clin Chem Lab Med 2009.47(9):1017-9.

Proposal for HMM0302 Protocol Revision

Changed from Ver.1.0（2017/02/28）to Ver.1.1（2018/03/02）

1.Key protocol revisions:

No.	Original Protocol	After Amendment	Relevant Section(s)/Page(s)	Reason for Amendment
1	Fasting C-peptide <1.0 ng/ml (0.33 nmol/L) at screening.	Fasting C-peptide <0.81 ng/ml (0.27 nmol/L) at screening.	Protocol Synopsis, Main Inclusion/Exclusion Criteria, Exceptions for Target Disease 4/Page 6.<br><br>4.2 Exclusion Criteria 4/Page 44	Referred to the lower limit of normal range in central laboratory
2	Received corticosteroid treatment (oral medication or injection) within 1 year at screening.	Received corticosteroid treatment (except for short-term external use) within 1 year at screening.	Protocol Synopsis, Main Inclusion/Exclusion Criteria, Exclusion Criteria 10/Page 7.<br><br>4.2 Exclusion Criteria 10/Page 44	To clarify the exclusion criteria for corticosteroids (dealing with exclusion criteria and restricted medications)
	Oral or injectable glucocorticoid.	Oral or injectable or inhaled glucocorticoid	4.4.2 Prohibited and Restricted Medications/Page 48.	
3	Abnormal electrocardiogram (ECG) results that may affect the safety evaluation or need medical interventions, as judged by the Investigator.	Abnormal electrocardiogram (ECG) results that may affect the safety evaluation or need medical interventions, as judged by the Investigator (except for stable coronary heart disease).	Protocol Synopsis, Main Inclusion/Exclusion Criteria, Exclusion Criteria 7/Page 8.<br><br>Protocol Synopsis, Randomization Criteria 12/ Page 10.<br><br>4.2 Exclusion Criteria, Physical Examination and Laboratory Results 7/Page 45.<br><br>4.3 Randomization Criteria, Exclusion Criteria 10/Page 47.	To clarify the exclusion criterion for ECG abnormalities

4	Subjects with uncontrolled hypertension (systolic blood pressure ⩾ 160 mmHg or diastolic blood pressure ⩾ 100 mmHg at screening) to antihypertensive treatment with stable doses for at least 4 weeks at screening.	Systolic blood pressure ≥ 160 mmHg or diastolic blood pressure ≥ 100 mmHg at screening, or who added/changed antihypertensive drugs or adjusted dose within 4 weeks before screening.	Protocol Synopsis, Main Inclusion/Exclusion Criteria, Physical Examination and Laboratory Results 8/Page 8.<br><br>4.2 Exclusion Criteria, Physical Examination and Laboratory Results 8/Page 45.	To clarify the exclusion criteria for hypertension
	Subjects with uncontrolled hypertension (systolic blood pressure ⩾ 160 mmHg or diastolic blood pressure ⩾ 100 mmHg at screening) to antihypertensive treatment with stable doses for at least 4 weeks in run-in period.	Systolic blood pressure ≥ 160 mmHg or diastolic blood pressure ≥ 100 mmHg) at randomization visit, or who added/changed the antihypertensive drugs or adjusted dose within run-in period.	Protocol Synopsis, Main Inclusion/Exclusion Criteria, Randomization Criteria 19/Page 9.<br><br>4.3 Randomization Criteria, Exclusion Criteria 8/Page 47.	
5	Randomization Criteria (laboratory tests for randomization are conducted on Day -5 [± 2 days] before randomization)	Randomization Criteria (laboratory tests for randomization and 12-lead ECG are conducted on Day -5 [± 2 days] before randomization)	Protocol Synopsis, Main Inclusion/Exclusion Criteria, Randomization Criteria/Page 9.<br><br>Contents, 4.3 Randomization Criteria /Page 18.<br><br>4.3 Randomization Criteria /Page 46.	As some study sites are unable to report ECG results on the same day, ECG was added to Visit 3 as a pre-randomization criterion to ensure smooth conduct of the study
	Blank（12-Lead ECG，Visit 3）	Adding *"X"*（12-Lead ECG，Visit 3）	Schedule of Assessments, Visit 3/Page 14.	
	No 12-Lead ECG at Visit 3	Adding "12-Lead ECG" at Visit 3	3.5.3 Visit 3/Page 34.	
	The randomization inclusion and exclusion criteria are reviewed (laboratory test results from Visit 3 are used as a reference);	The randomization inclusion and exclusion criteria are reviewed (laboratory test results and 12-lead	3.5.4 Visit 4: Randomization Visit (Day 1)/Page 35.	

		ECG from Visit 3 are used as a reference);		
	A 12-lead ECG examination is conducted at each visit except Visit 2, Visit 3, and Visit 16.	A 12-lead ECG examination is conducted at each visit except Visit 2, ~~Visit 3,~~ and Visit 16.	7.10 Electrocardiogram/Page 66.	
6	Week -4（Visit 2）	Week -4 ±3 Days（Visit 2）	Schedule of Assessments/Page 14.	Added a visit window period of ± 3 days for Visit 2 to match actual operational needs.
	Single-blind placebo run-in period lasts from Day -28 days to Day -1 (inclusive).	Single-blind placebo run-in period lasts from Day -28±3 days to Day -1 (inclusive).	Schedule of Assessments, Note 1/Page 15.	
	Visit 2: Single-Blind Placebo Run-in Period (Day -28)	Visit 2: Single-Blind Placebo Run-in Period (Day -28 ±3 days)	Contents, 3.5.2 Visit 2/Page 17.<br><br>3.5.2 Visit 2/Page 33	
7	All laboratory sampling must be collected under fasting before dosing (except for MMTT)	All laboratory sampling must be collected under fasting before dosing (except for MMTT 30-min and 120-min)	Schedule of Assessments, Note a/Page 15.	To clarify the workflow.
	(all laboratory tests are conducted in the central laboratory, and all samples are collected in fasting status)	(all laboratory tests are conducted in the central laboratory, and all blood samples are collected in fasting status)	3.5.1 Visit 1: Screening /Page 32.	
	(all laboratory tests are conducted in the central laboratory, and all samples are collected in fasting status)	(all laboratory tests are conducted in the central laboratory, and all blood samples are collected in fasting status)	3.5.3 Visit 3 /Page 34.<br><br>3.5.5 Visit 5 /Page 36.<br><br>3.5.6 Visit 6 /Page 37.	
	(all laboratory tests are conducted in the central laboratory before drug administration and in fasting status)	(all laboratory tests are conducted in the central laboratory and blood samples are collected when subjects are in fasting status, except 30-min and 120-min MMTT samples)	3.5.4 Visit 4/Page 34.<br><br>3.5.7 Visit 7/Page 38.<br><br>3.5.10 Visit 10/Page 39.<br><br>3.5.15 Visit 15/Page 41.	

8	MMTT: The blood samples for evaluating plasma glucose, insulin and C-peptide are collected before and 120 minutes after the instant noodles standard meal being taken……The standard meal should be taken before drug administration at Visit 4. At Visits 7, 10 and 15, the standard meal is taken after drug administration.	MMTT: The blood samples for evaluating plasma glucose, insulin and C-peptide are collected ~~before,~~ 30 minutes (±3 minutes) and 120 minutes (±5 minutes) after the instant noodles standard meal being taken……The standard meal should be taken before drug administration at Visit 4. At Visits 7, 10 and 15, the standard meal is taken after drug administration (blood sampling at 30 minutes after the standard meal is applicable to subjects enrolled after the protocol v1.1 taking effect).	Schedule of Assessments, Note 12/Page 16.	Added the 30-minute testing point for the MMTT to optimize the study protocol.
	Totally four MMTTs will be conducted for the whole study, including one at Visit 4 before study drug administration, and once each at Visits 7, 10 and 15 after study drug administration. Before the instant noodle standard meal, blood samples need to be collected at the timepoint 0 and then the instant noodles standard meal starts to be taken (timing starts from the first chewing of the noodles and the meal is finished within 10 minutes). Blood samples are collected at 120 minutes (±5 minutes are allowed) after the instant noodles standard meal. When blood samples are being taken, food, water, and strenuous exercise is	Totally four MMTTs will be conducted for the whole study, including one at Visit 4 before study drug administration, and once each at Visits 7, 10 and 15 after study drug administration (except 0-min MMTT). Before the instant noodle standard meal, blood samples need to be collected at the timepoint 0 and then the instant noodles standard meal starts to be taken (timing starts from the first chewing of the noodles and the meal is finished within 10 minutes). Blood samples are collected at 30 minutes (±3 minutes are allowed), 120 minutes (±5 minutes are allowed) after the instant noodles standard meal. When blood samples are being taken, food, water, and strenuous exercise is not allowed until all samples for MMTT are collected.	14 Appendix, Appendix 3/Page 80.	

	not allowed until all samples for MMTT are collected.	(Blood sampling at 30 minutes applies to the subjects who enroll after the Version1.1 profile take effect).		
9	Subject must be in fasting status (for at least 8 hours) before going to regular scheduled visits (except Visit 2)	Subject must be in fasting status (for at least 8 hours) before going to regular scheduled visits ~~(except Visit 2)~~	3.4 Preparation for the Study/Page 31.	To address the problem of inconsistency in calculating compliance during the run-in period.

2. Other minor protocol revisions:

10	Version: Version 1.0<br>Date: February 28, 2017	Version: Version 1.1<br>Date: March 02, 2018	Cover.<br><br>Protocol Signature Page/Page 4.<br><br>Page Footer/Each Page.	Version changes
11	Only one Contract Research Organization company in Version 1.0	Added" Wuxi Clinical" as "Contract Research Organization (2)"	Study Participants/Page 2.	Added a new Contract Research Organization company.
12	Subjects clinically diagnosed with type I diabetes mellitus	Subjects clinically diagnosed with type 1 diabetes mellitus	Protocol Synopsis, Main Inclusion, Exclusion Criteria, Exceptions for Target Disease 6/Page 6.<br><br>4.2 Exclusion Criteria, Exceptions for Target Disease 6/Page 44.	Corrected the typos.
13	Concomitant treatment with potent or moderate CYP3A4/5 inducers or inhibitors (see Section 4.4.2).	Concomitant treatment with potent or moderate CYP3A4~~/5~~ inducers or inhibitors (see Section 4.4.2).	Protocol Synopsis, Main Inclusion, Exclusion Criteria, Prohibited Therapies and/or Medications 2/Page 8.<br><br>4.2 Exclusion Criteria, Prohibited Therapies and/or Medications 2/Page 46.<br><br>4.4.2 Prohibited and Restricted Medications/Page 48.	To keep consistent with Investigator's Brochure.
14	Subjects who took any other investigational drugs or participated in any other clinical study within 30 days before screening.	Subjects who participated in ~~any~~ other clinical trials or took ~~any~~ other study drug within 30 days before screening.	Protocol Synopsis, Main Inclusion, Exclusion Criteria, Other Exclusion Criteria 4/Page 9,<br><br>4.2 Exclusion Criteria, Other Exclusion Criteria 4/Page 46	To clarify the exclusion criteria.

15	Systemic quantitative analysis is conducted on the influences of various factors on HMS5552 PK/PD characteristics, e.g., demographics, pathology, disease course, concomitant medications.	Systemic quantitative analysis is conducted on the influences of various factors on HMS5552 PK/PD characteristics, e.g., demographics, ~~pathology,~~ disease course, concomitant medications.	Protocol Synopsis, Population Pharmacokinetic/Pharmacodynamic Analysis (PopPK/PD)/Page 12<br><br>10 Population PK/PD Analysis/Page 74.	This protocol does not involve pathological design.
16	The instant noodles standard meal produced by the COFCO and provided by the Sponsor……	The instant noodles standard meal ~~produced by the COFCO and~~ provided by the Sponsor……	Schedule of Assessments, Note 12/Page 16.<br><br>6 Study Endpoints/Page 59<br><br>14 Appendix, Appendix 3 /Page 80.	Removed the brand description of the instant noodles due to the transfer of ownership of "standard instant noodles meal"
17	The time of blood sampling and the time for the closest drug administration before blood sampling are recorded in detail.	The time of blood sampling and the time (before the scheduled visit) for the closest investigational drug (HMS5552/Placebo) administration before blood sampling are recorded in detail.	Schedule of Assessments, Note 14/Page 16	To avoid misunderstanding
18	Type 2 diabetes mellitus (T2DM) accounts for approximate 90% to 95% of all diagnosed diabetes cases and is characterized by hyperglycemia caused by decreased insulin secretion, increased insulin resistance and hepatic glucose production.	Type 2 diabetes mellitus (T2DM) accounts for approximate 90% to 95% of all diagnosed diabetes cases and is characterized by hyperglycemia caused by decreased insulin secretion, increased insulin resistance and hepatic glucose output.	1 Background, 1.1 Introduction of Disease/Page 23	More accurately
19	the prevalence of diabetes in adults above 18 years old increased to 11.6%, with an absolute number of 1.14 billion.	the prevalence of diabetes in adults above 18 years old increased to 11.6%, with an absolute number of 114 million.	1 Background, 1.1 Introduction of Disease/Page 23	Corrected the typos.
20	Subjects can only be randomized once in this study.	Each Subject can only be randomized once in this study.	3 Study Design, 3.3 Subject Enrollment, Randomization, and Treatment/Page 29	More accurately

21	Whole blood samples are collected from some subjects for DNA test.	Whole blood samples are collected from some subjects for DNA test (only for subjects who signs the informed consent form for DNA sampling).	3.5.4 Visit 4/Page 35	More accurately
22	Blood sampling for population PK	Blood sampling for population PK (before subjects taking the study medications);	3.5.5 Visit 5/Page 36	To clarify the sampling time for PK
	Blood sampling for population PK	Blood sampling for population PK (before taking study medications, MMTT 120min);	3.5.7 Visit 7/Page 39<br>3.5.10 Visit 10/Page 40<br>3.5.15 Visit 15/Page 42	
23	If a subject is confirmed to withdraw from the study due to hyperglycemia, the subject must complete all the laboratory tests same with those required at Visit 15 (open-label treatment) before withdrawal (the information on the "Unplanned Study Visits" page in eCRF needs to be completed if the visit does not overlap with the planned study visits).	If a subject is confirmed to withdraw from the study due to hyperglycemia, the subject must complete termination visit before withdrawal (refer to Section 4.6).	4.5.2 Withdrawal due to Hyperglycemia/Page 52	More accurately
24	AEs are followed up (see Section 7.2).	AEs are recorded and followed up (see Section 7.2).	4.6 Procedures for Subject Withdrawal/Page 53	More accurately
25	Manufacturer: Merck Serono Co. Ltd.	Manufacturer: Sino-American Shanghai Squibb Pharmaceuticals Ltd.	5.1.1 Drug Name, Strength, Formulation, Basic Drug: Metformin (Glucophage)/Page 54	More accurately
26	In a clinical study, an AE can be any undesirable medical condition occurring at any time after the ICF has been signed, even if no	~~In a clinical study, an AE can be any undesirable medical condition occurring at any time after the ICF has been signed, even if no~~	7.1 Adverse Event/Page 59	Removed the irrelevant content

	investigational drug has been given yet.	~~investigational drug has been given yet.~~		
27	In this study, AEs are collected from the first dose of study medication in run-in period to the last visit.	In this study, AEs (except for SAEs) are collected from the first dose of study medication in run-in period to the last visit.	7.2.2 Recording of Adverse Event Time and Collection Period for Adverse Event/Page 61	To clarify the collection of AEs (but not the SAEs).
28	Throughout the study, all efforts are made to measure pulse rate, respiratory rate and blood pressure from the same arm.	Throughout the study, all efforts are made to measure pulse rate, ~~respiratory rate~~ and blood pressure from the same arm.	7.11 Vital Signs/Page 67	Corrected the typos.
29	the CROs' SOPs should be followed	the CROs' SOPs, the Sponsor's SOPs or the written agreement agreed by both parties should be followed	11.7 Monitoring/Page 75	To optimize the workflow.
30	All the procedures should be completed before the updated profile takes effect.	All the procedures should be completed and follow the previous edition before the updated profile takes effect.	11.6 Protocol Amendment/Page 75	To clarify the implementation of the protocol.
31	2 hours Postload Plasma Glucose for Diabetes Mellitus: ≥7.0	2 hours Postload Plasma Glucose for Diabetes Mellitus: ≥~~7.0~~ 11.1	14 Appendix, Appendix 1， Table 1 /Page 77.	Corrected the typos.
32	Drug administration for the basic drug metformin is kept consistent throughout the study.	~~Drug administration for the basic drug metformin is kept consistent throughout the study.~~	14 Appendix, Appendix 3/Page 80	More accurately.

A 24-week multi-center, randomized, double-blind, placebo-controlled, Phase III study to evaluate the efficacy and safety of HMS5552 with additional 28-week open-label treatment to evaluate the safety of HMS5552 combined with Metformin in subjects with type II diabetes mellitus

Statistical Analysis Plan

**Sponsor**	Hua Medicine (Shanghai) Ltd.
**Protocol Number**	HMM0302
**Protocol Version**	1.1
**Authoring institution**	dMed Biopharmaceutical Co., Ltd.
**Document name**	Statistical Analysis Plan
**Version**	1.0
**Date**	08 JUN 2020

*

Statement: The Signature page means that all Signatories have reviewed and approved the follow chats and contents in this document.

**Author**		
**Name and Title**	**Signature**	**Date (yyyy-mm-dd)**
**Wei Zhang**<br><br>**dMed Biopharmaceutical Co., Ltd. Biostatistician**		

**CRO Approval**		
**Name and Title**	**Signature**	**Date (yyyy-mm-dd)**
**Jie Li**<br><br>**dMed Biopharmaceutical Co., Ltd. Biostatistician Director**		

**Sponsor Approval**		
**Name and Title**	**Signature**	**Date (yyyy-mm-dd)**
**Yi Zhang**<br><br>**Clinical Research and Development Department, Hua Medicine (Shanghai) Ltd**		

*

Content

LIST OF ABBREVIATIONS......6

1. INTRODUCTION......7

2. SUMMARY OF KEY PROTOCOL INFORMATION......7

2.1. CHANGES TO THE PROTOCOL DEFINED STATISTICAL ANALYSIS PLAN......7
2.2. STUDY OBJECTIVES AND ENDPOINTS......7
2.2.1. Study Objectives......7
2.2.1.1. Primary Objective......7
2.2.1.2. Secondary Objectives......8
2.2.2. Study Endpoints......8
2.2.2.1. Primary Endpoint......8
2.2.2.2. Secondary Endpoints......9
2.2.2.3. Other Efficacy Endpoints......9
2.2.2.4. Safety Endpoints......9
2.3. STUDY DESIGN......9
2.3.1. Study Design and Rationale......9
2.3.2. Study Flow Chart......11
2.4. SAMPLE SIZE DETERMINATION......15
2.5. STATISTICAL HYPOTHESES......15
2.6. RANDOMIZATION......15

3. PLANNED ANALYSES......17

3.1. DOUBLE-BLIND-PERIOD ANALYSIS – WHEN DOUBLE-BLIND TREATMENT PERIOD FINISHES......17
3.2. OPEN-LABEL-PERIOD ANALYSIS AND FULL-TREATMENT-PERIOD ANALYSIS – WHEN ENTIRE STUDY FINISHES 17

4. ANALYSIS POPULATION......18

4.1. PROTOCOL DEVIATION......19
4.2. INCORRECT TREATMENTS......19

5. GENERAL CONVENTIONS OF STATISTICAL ANALYSIS.......19

5.1. MULTICENTER STUDY......20
5.2. STRATIFICATION FACTORS......20
5.3. MULTIPLICITY......21
5.4. STUDY TREATMENT DISPLAY DESCRIPTORS......21

6. GENERAL CONVENTIONS FOR DATA HANDLING......22

6.1. PREMATURE WITHDRAW AND MISSING DATA 22
6.1.1. Imputation on Start / End Date of Adverse Events 22
6.1.2. Missing data in Start / End Date of Study Medications 23
6.1.3. Imputation on Start / End Date of Concomitant Medications 24
6.2. DERIVATION AND CONVERSION OF DATA 25
6.3. DEFINITION OF BASELINE AND CHANGE FROM BASELINE 25
6.4. MULTIPLE MEASUREMENT 26
6.5. DATA DISPLAY 26
6.6. GENERAL RULE OF END-OF-TREATMENT VISIT AND UNSCHEDULED VISIT 27
6.7. PROHIBITED THERAPIES AND/OR MEDICATIONS 27
6.8. MISSING EFFICACY DATA DUE TO CORONAVIRUS DISEASE 2019 (COVID-19) 28

7. STUDY POPULATION ANALYSES 29

7.1. DISPOSITION OF SUBJECTS 29
7.2. DEMOGRAPHIC AND BASELINE CHARACTERISTICS 29
7.3. EXPOSURE TO STUDY DRUG AND COMPLIANCE 30
7.4. PRIOR AND CONCOMITANT MEDICATION 30

8. PRIMARY STATISTICAL ANALYSES 31

8.1. PRIMARY EFFICACY ANALYSES 31
8.1.1. Sensitivity Analyses of Primary Efficacy endpoint 32
8.1.2. Supplementary Analyses of Primary Efficacy endpoint 32

9. SECONDARY STATISTICAL ANALYSES 33

9.1. SECONDARY EFFICACY ANALYSES 33
9.1.1. Change in Baseline in Postprandial Plasma Glucose (PPG) 33
9.1.2. Change from Baseline in Fasting Plasma Glucose (FPG) 33
9.1.3. Hba1c Response Rate (proportion of subjects with HbA1c< 7%) 33
9.1.4. Change from Baseline in HbA1c at Each Visit (Except Week 24) 33
9.2. OTHER EFFICACY ANALYSES 34
9.3. SAFETY ANALYSES 34
9.3.1. Exposure of Subjects 34
9.3.2. Adverse Events 34
9.3.3. Incidence Rate of Hypoglycemia Events 36
9.3.4. Death 38
9.3.5. Physical Examination 38
9.3.6. Vital Sign 38

9.3.7. 12-lead ECG 38
9.3.8. Clinical Lab Test 39
9.3.9. HCG 39

10. DATABASE LOCK 39

11. REFERENCE 40

List of Abbreviations

Abbreviations	Full
AE	Adverse Event
ANCOVA	Analysis of Covariance
ARH (1)	Heterogeneous first-order autoregressive
BDR	Blind Data Review
BID	Twice in a day
BMI	Body Mass Index
CDISC	Clinical Data Interchange Standards Consortium
CFDA	China Food and Drug Administration
CRO	Contract Research Organization
DBL	Database Lock
eCRF	electronic Case Report Form
FAS	Full Analysis Set
FPG	Fasting Plasma Glucose
HbA1c	Glycosylated Hemoglobin
HIV	Human Immunodeficiency Virus
ICH	International Conference on Harmonization
IWRS	Interactive Web Response Service
LOCF	Last Observation Carried Forward
MedDRA	Medical Dictionary for Regulatory Activities
MMRM	Mixed Model for Repeated Measure
MMTT	Mixed-meal Tolerance Test
OR	Odds Ratio
PD	Pharmacodynamics
PK	Pharmacokinetic
PPG	Post-prandial Glucose
PPS	Per protocol Set
PT	Preferred Term
SAE	Serious Adverse Event
SAP	Statistical Analysis Plan
SAR	Statistical Analysis Report
SMBG	Self-monitoring of blood glucose
SOC	System Organ Class
SS	Safety Set
T2DM	Type II Diabetes Mellitus

1. Introduction

The purpose of this document is to describe the planned analyses included in clinical study report for registration and application. This statistical analysis plan (SAP) introduces all the planned analyses and output contents of study HMM0302. This SAP is established on the basis of chapter 9 in Protocol (HMM0302).

This SAP is established on the basis of：

Document	Version (Effective Date)
HMM0302 Study Protocol	Version 1.1 (March 2nd, 2018)
HMM0302 Case Report Form	Version 6.0 (June 18th, 2019)

2. Summary of Key Protocol Information

2.1. Changes to the Protocol Defined Statistical Analysis Plan

Protocol	SAP	Reasons
Alternative hypothesis: After 24-week treatment of HMS5552 75 mg BID, the decrease of HbA1c level in subjects with T2DM is significantly different with placebo group	Alternative hypothesis: After 24-week treatment of HMS5552 75 mg BID, the decrease of HbA1c level in subjects with T2DM is different with placebo group	"significantly" was removed, which makes SAP more accurate while no conflict with protocol.
No other efficacy endpoints in protocol.	2.2.2.3. Other Efficacy Endpoints<br>• Change of HOMA2-β from baseline<br>• Change of HOMA2-IR from baseline	Exploration analyses

2.2. Study Objectives and Endpoints

2.2.1. Study Objectives

2.2.1.1. Primary Objective

The primary objective of this study is to assess the change of Glycosylated Hemoglobin (HbA1c) from baseline among subjects with Type II Diabetes Mellitus (T2DM) whose glycemic level is not fully controlled after stable treatment of Metformin by comparing

the treatment group (HMS5552 tablets 75mg BID combined with Metformin tablets 1500mg per day, oral) with the placebo group (placebo tablets BID combined with Metformin tablets 1500mg per day, oral) after 24-week double-blind treatment.

2.2.1.2. Secondary Objectives

The secondary objective is to compare the following parameters between the treatment group (HMS5552 tablets 75mg BID combined with Metformin tablets 1500mg per day, oral) and placebo group (placebo tablets BID combined with Metformin tablets 1500mg per day, oral) after 24-week double-blind treatment and additional 28-week open-label treatment (Week 52) among subjects with T2DM whose glycemic level is not fully controlled after stable treatment of Metformin:

- Change of 2-hour postprandial plasma glucose (2h PPG) from baseline at the end of double-blind treatment (Week 24)
- Change of fasting plasma glucose (FPG) from baseline at the end of double-blind treatment (Week 24)
- HbA1c response rate: the proportion of subjects with HbA1c< 7.0% at the end of double-blind treatment (Week 24)
- Change of HbA1c from baseline at each visit during the double-blind treatment period, except Week 24
- Safety profiles at the end of double-blind treatment (Week 24) and study treatment (Week 52).

2.2.2. Study Endpoints

2.2.2.1. Primary Endpoint

The primary endpoint is to compare the change of HbA1c level from baseline between the treatment group (HMS5552 tablets 75 mg BID combined with Metformin tablets 1500mg per day, oral) with the placebo group (placebo tablets BID combined with Metformin tablets 1500mg per day, oral) after 24-week double-blind treatment among subjects with T2DM whose glycemic level is not fully controlled after stable treatment of Metformin.

2.2.2.2. Secondary Endpoints

The secondary endpoints are to compare the following parameters between treatment group (HMS5552 tablets 75 mg BID combined with Metformin tablets 1500mg per day, oral) and placebo group (placebo tablets BID combined with Metformin tablets 1500mg per day, oral) after each of 24-week double-blind treatment and additional 28-week open-label treatment (Week 52) among subjects with T2DM whose glycemic level is not fully controlled after stable treatment of Metformin:

- Change of 0.5-hour postprandial plasma glucose (0.5h PPG) from baseline
- Change of 2-hour postprandial plasma glucose (2h PPG) from baseline
- Change of FPG from baseline
- HbA1c response rate: the proportion of subjects whose HbA1c< 7.0%
- Change of HbA1c from baseline at each visit, except Week 24

2.2.2.3. Other Efficacy Endpoints

- Change of HOMA2-β from baseline
- Change of HOMA2-IR from baseline

2.2.2.4. Safety Endpoints

- Adverse events (AEs) throughout the study
- Incidence of hypoglycemic events
- Physical examination
- Vital signs
- 12-lead ECG
- Clinical laboratory examinations (routine blood test, blood biochemistry and routine urine test)

2.3. Study Design

2.3.1. Study Design and Rationale

This is a 24-week multi-center, randomized, double-blind, placebo-controlled study.

*

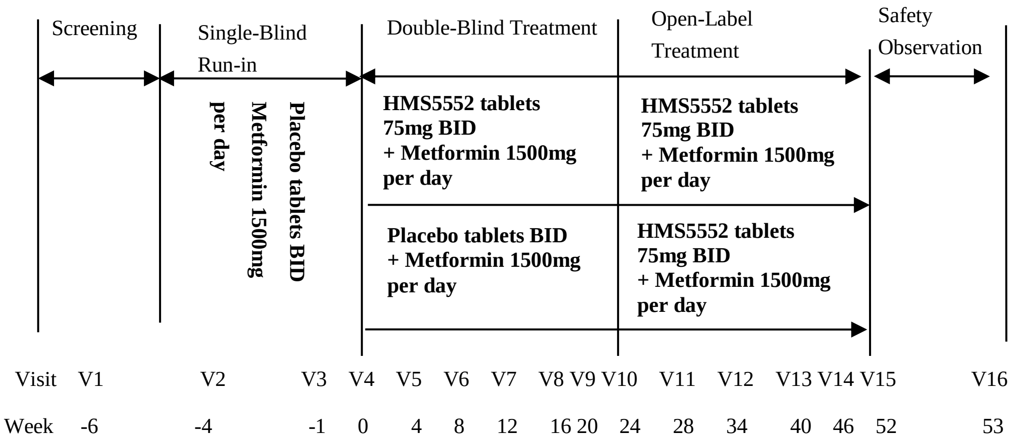

Note: More details in the Study Flow Chart (Chapter 2.3.2)

2.3.2. Study Flow Chart

Study Flow Chart

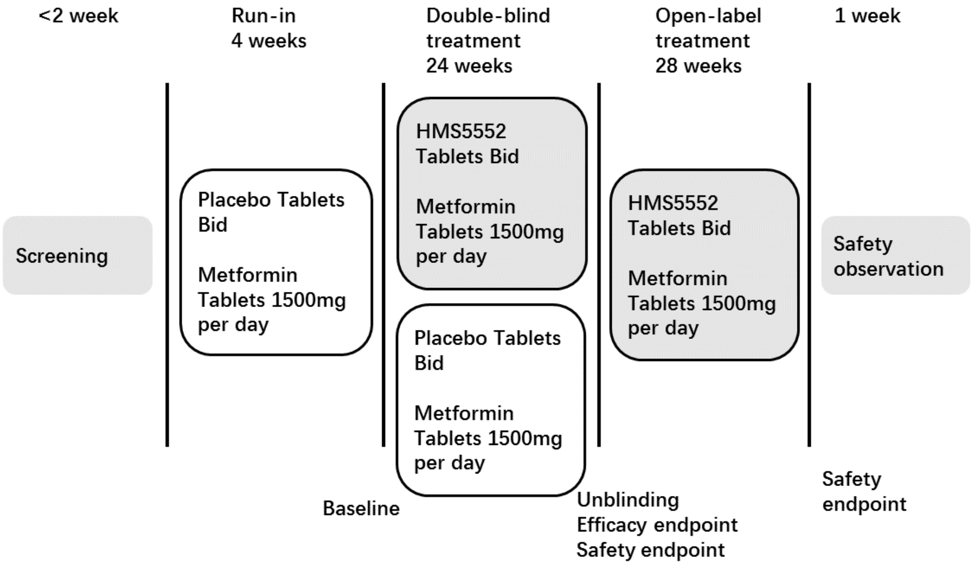

*

Schedule of Assessments

Stage	Screening	Run-in Period[1]		Randomization	Double-Blind Treatment Period						Open-Label Treatment Period					Safety Observation
Visit number	Visit 1	Visit 2	Visit 3	Visit 4	Visit 5	Visit 6	Visit 7	Visit 8	Visit 9	Visit 10[2]	Visit 11	Visit 12	Visit 13	Visit 14	Visit 15[3]	Visit 16[4]
Visit time	Week -6 to -4	Week -4± 3 days	Day -5±2 days	Day 1	Week 4 ± 4 days	Week 8 ± 4 days	Week 12 ± 4 days	Week 16 ± 4 days	Week 20 ± 4 days	Week 24 ± 4 days	Week 28 ± 4 days	Week 34 ± 6 days	Week 40 ± 6 days	Week 46 ±6 days	Week 52 ±6 days	Week 53 ±2 days
**Study Procedures**																
Informed consent	X															
Demographics	X															
Medical history and prior medications	X			X												
Inclusion/exclusion criteria	X			X												
Concomitant medications	X	X	X	X	X	X	X	X	X	X	X	X	X	X	X	X
Randomization				X												
Adverse events		X	X	X	X	X	X	X	X	X	X	X	X	X	X	X
Physical examination[5]	X			X	X	X	X	X	X	X	X	X	X	X	X	
Vital signs [6]	X			X	X	X	X	X	X	X	X	X	X	X	X	
12-Lead ECG	X		X	X	X	X	X	X	X	X	X	X	X	X	X	
**Laboratory Tests (in central laboratory)**																
HbA1c	X		X	X	X	X	X	X	X	X	X	X	X	X	X	
FPG	X		X	X	X	X	X	X	X	X	X	X	X	X	X	
Routine blood test[7]	X		X	X	X	X	X	X	X	X	X	X	X	X	X	
Routine urine test[8]	X		X	X	X	X	X	X	X	X	X	X	X	X	X	
Blood biochemistry[9]	X		X	X	X	X	X	X	X	X	X	X	X	X	X	
Virology test[10]	X															
Pregnant test[11]	X	X	X	X	X	X	X	X	X	X	X	X	X	X	X	
MMTT[12]				X			X			X					X	
Biomarker[13]	X			X			X			X					X	
PK sampling[14]					X		X			X					X	
DNA sampleing[15]				X												
**Instructions/Suggestions**																
Health education[16]	X	X	X	X	X	X	X	X	X	X	X	X	X	X	X	
Glucometer distribution		X														

*

**Stage**	**Screening**	**Run-in Period[1]**		**Randomization**	**Double-Blind Treatment Period**						**Open-Label Treatment Period**					**Safety Observation**
Visit number	Visit 1	Visit 2	Visit 3	Visit 4	Visit 5	Visit 6	Visit 7	Visit 8	Visit 9	Visit 10[2]	Visit 11	Visit 12	Visit 13	Visit 14	Visit 15[3]	Visit 16[4]
Visit time	Week -6 to -4	Week -4± 3 days	Day -5±2 days	Day 1	Week 4 ± 4 days	Week 8 ± 4 days	Week 12 ± 4 days	Week 16 ± 4 days	Week 20 ± 4 days	Week 24 ± 4 days	Week 28 ± 4 days	Week 34 ± 6 days	Week 40 ± 6 days	Week 46 ±6 days	Week 52 ±6 days	Week 53 ±2 days
SMBG check[17]			X	X	X	X	X	X	X	X	X	X	X	X	X	
Subject diary card		X		X	X	X	X	X	X	X	X	X	X	X		
**Study Medication**																
Drug distribution		X		X	X	X	X	X	X	X	X	X	X	X		
Review of drug usage/ recycling [18]				X	X	X	X	X	X	X	X	X	X	X	X	

ECG: electrocardiogram; eCRF: electronic case report form; FPG: fasting plasma glucose; HbA1c: glycated hemoglobin; MMTT: mixed-mean tolerance test; SMBG: self-monitoring of blood glucose.

Note: a. All laboratory sampling must be completed in central laboratory under fasting before dosing (except for 30 min and 120 min MMTT sampling), suggested time: 7:00 to 10:00 a.m. b. Mixed-meal tolerance test is suggested to start between 7:00 and 9:00 a.m. c. The schedule of visit window is based on normal working time. In case of the National Day, Spring Festival and other holidays, the Investigator can arrange for administration of the study medications accordingly and reschedule the next visit to a time closest to the visit scheduled per study protocol. These situations are not considered as protocol deviation, while causes for early/late visit need to be recorded on eCRF.

1. Single-blind placebo run-in period lasts from Day -28±3 days to Day -1 (inclusive).
2. The double-blind treatment period (Visit 10) ends when a subject finishes all the 24-week treatment. If early withdrawal occurs within the double-blind treatment period, a termination visit needs to be completed within 7 days (the seventh day inclusive) after the last dose of study medications. In addition, a telephone follow-up for safety is conducted within 1 week after the last dose of study medications. If administration of the study medications has been stopped by the subjects for ≥ 7 days before early withdrawal, a termination visit needs to be completed as much as possible. The tests in the termination visit are the same as Visit 15.
3. The open-label treatment period (Visit 15) ends when a subject finishes all the 52-week treatment. If early withdrawal occurs within the open-label treatment period, a termination visit needs to be completed within 7 days (the seventh day inclusive) after the last dose of study medications. In addition, a telephone follow-up for safety is conducted within 1 week after the last dose of study medications. If administration of the study medications has been stopped by the subjects for ≥ 7 days before early withdrawal, a termination visit needs to be completed as much as possible. The tests in the termination visit are the same as Visit 15. If administration of the study medications has been stopped at the termination visit, the MMTT could be omitted.
4. All study medications are stopped in the safety observation period. Visit 16 is conducted by telephone at which adverse events and concomitant medications occurring in the safety observation period are recorded. Any adverse event that occurs in the treatment period but doesn't have an outcome is also followed up by the Investigator. Additional visits are arranged if needed at which examinations are conducted according to the reported adverse event.
5. Physical examinations: general appearance, head, neck, chest (heart and lungs), abdomen, and limbs etc..
6. Vital signs: for Visit 1, blood pressure, pulse rate, breath rate, body temperature (axillary), height, weight. For other visits, all parameters except height are checked.
7. Routine blood test: hemoglobin, hematocrit, red blood cell count, white blood cell count, neutrophils count, basophils count, eosinophils count, monocytes count, lymphocytes count, and platelet count.

8. Routine urine test: pH, specific gravity, protein, glucose, red blood cells, white blood cells, and ketone bodies.
9. Blood biochemistry: blood glucose; total bilirubin, alanine aminotransferase, aspartate aminotransferase, gamma-glutamyl transferase, alkaline phosphatase, albumin, total protein, lactate dehydrogenase, urea nitrogen, creatinine, uric acid, sodium, potassium, chloride, calcium, creatine kinase, creatine kinase isoenzyme, total cholesterol, high-density lipoprotein cholesterol, low-density lipoprotein cholesterol, triglyceride.
10. Virology test: hepatitis A virus antibody IgM (IgM anti-HAV), hepatitis B surface antigen (HBsAg), hepatitis B surface antibody (anti-HBs), hepatitis B core antibody IgM (IgM anti-HBc), hepatitis C virus antibody (anti-HCV), human immunodeficiency virus (HIV) antibodies.
11. Pregnancy test (only for females with childbearing potential). From Visit 1 to Visit 15, blood pregnancy test must be performed in the central laboratory. During the study, if any amenorrhea or irregular menstruation is reported for any female subject at any time, a blood pregnancy test should be performed in the central laboratory immediately to exclude or validate pregnancy. Drug administration is stopped immediately once a positive result is shown.
12. Mixed meal tolerance test. The instant noodles standard meal provided by Sponsor should be taken before drug administration at Visit 4. The blood samples for evaluating plasma glucose, insulin and C-peptide are collected 30 minutes(±3 minutes) and 120 minutes(±5 minutes) after the standard meal. At Visits 7, 10 and 15, the standard meal is taken after drug administration (30-minute blood sampling point is applicable to subjects enrolled after the protocol v1.1 taking effect).
13. Biomarker: fasting insulin and C-peptide. At Visit 1, only C-peptide is tested and evaluated as one of the exclusion criteria.
14. Population pharmacokinetics. At Visit 5, blood samples are collected before drug administration. At Visits 7, 10 and 15, blood sampling schedule is the same as 120 minute MMTT. The time of blood sampling and the time for the closest drug administration before blood sampling are recorded in detail (not on the same day of visit, usually the day before the visit).
15. DNA sampling. At Visit 4, DNA samples are collected. This sampling is optional.
16. Diabetes education and health/exercise guidance. The Investigator fully assesses the life and disease status for each subject. If any inappropriate lifestyle is identified (e.g. overeating during holidays, etc.) for any subject, the diabetes education and health/exercise guidance should be given to him/her timely and repeatedly.
17. SMBG examination. For each subject, fingertip blood glucose under fasting or postprandial is required to be monitored at home at least twice a week. If suspicious hypoglycemia or hyperglycemia events occur, additional blood glucose tests are conducted. The results of the additional tests are recorded in subject diaries. At each visit, the Investigator should review the subject diaries carefully and then make suggestions on drug administration and blood glucose monitoring for the next stage.
18. Review of drug usage/recycling. The Investigator should review and confirm the drug compliance record from Visit 2 to Visit 4 (run-in period) and from Visit 4 to Visit 15 (treatment period). Queries should be made if any problem is found. At Visit 4, drug compliance is evaluated for the run-in period. Guidance should be given if any subject does not follow the drug administration specified in the study protocol.

2.4. Sample Size Determination

The number of subjects to be recruited in this study is planned as 750. The number of subjects may be increased by 20% if drop-out is considered. The total number of subjects will be no more than 938. Subjects are randomized at a ratio of 1:1 to the experimental group (HMS5552 75mg Bid + stable-dose Metformin) or the control group (placebo Bid + stable-dose Metformin). It is estimated that no more than 469 subjects in the experimental group and 469 subjects in the control group will be included.

Based on the planned sample size and the treatment assignment ratio (750 subjects, experimental group: control group = 1:1), a power of 95.4% would be provided to detect a difference of at least 0.4% in the change of HbA1c from baseline at week 24 between experimental group and control group, assuming there is no difference between the two groups with the standard deviation of 1.5% and the significance level of 0.05 (2-sided).

2.5. Statistical Hypotheses

H_0: After 24-week double-blind treatment, there is no difference in decrease of HbA1c between the experimental group and control group.

H_1: After 24-week double-blind treatment, there is difference in decrease of HbA1c between the experimental group and control group.

The adjusted least-square mean and 95% CI of treatment difference (experimental group vs. control group) in the primary efficacy endpoint will be calculated. The significance level of the between-group comparison is 0.05 (2-sided). If $p < 0.05$, it suggests that the between-group difference is statistically significant. If the upper limit of the 95% CI is less than 0, it may be claimed that HMS5552 is superior to the placebo in lowering HbA1c after 24-week treatment.

2.6. Randomization

At screening visit, a unique screening number is assigned to each subject via interactive web response service (IWRS) system. This number is used to identify the subject throughout the study and will not be assigned to any other subjects.

At the beginning of placebo run-in period, sites contact IWRS system to dispense study medications for run-in period. At the end of run-in period, subjects who are eligible for double-blind treatment are randomized via IWRS by the Investigator to treatment/placebo group at a 1:1 ratio (HMS5552: placebo) according to the

randomization factors (duration of T2DM and HbA1c value at baseline).

Randomization scheme is generated from IWRS system (Balance, MediData Solution) and preserved by the IWRS vendor throughout the study. At all visits, study medications are dispensed via the IWRS system along with an identification code (package number). The identification code (package number), which is provided to the IWRS vendor by the drug packaging vendor, should not be assigned to subjects in alphabetic or numerical order and should match the code printed on drug boxes. The IWRS system is available at any time, 24 hours a day and 7 days a week.

Eligible subjects are enrolled into the study. At each site, the Investigator assigns each subject an identification code and informs them of the drug administration instructions and time for the first dose of study medications.

Under any circumstance, subjects who failed to meet inclusion criteria or met any of exclusion criteria should not be enrolled or receive any study treatments.

If there is any subject who is not eligible but enrolled or randomized, the subject should not be discontinued from the study by the Investigator. The decision is made based on a full discussion among the Investigator, MediData Solution (randomization service vendor) and the Sponsor.

If incorrect drug administration occurs because of dispensing error made by the Investigator, the Investigator should not correct the drug administration or discontinue the subject from the study. The incorrect drug administration needs to be maintained. A final decision is made after the Investigator has a full discussion with MediData Solution and the Sponsor.

This is a double-blind study. The placebo tablets have the same size, color, odor, and appearance as the investigational drug. Unique identification number (drug box serial number) is provided by the drug packing vendor and marked on the label of drug boxes. By central randomization, the randomization codes are assigned to subjects who meet randomization criteria at randomization visit by IWRS system based on randomization factors (duration of T2DM ≤ 3 years or > 3 years, and baseline HbA1c level ≤ 8.5% or > 8.5%) and the block size. The corresponding drug box serial numbers are provided to subjects at each visit.

During the study, the team members or their representatives from Hua Medicine Ltd., staffs from sites, staff, data management and statistical teams or personnel from Contract Research Organization (CRO) are not accessible to the randomization scheme.

3. Planned Analyses

3.1. Double-Blind-Period Analysis – When Double-blind Treatment Period Finishes

The double-blind-period analysis will be conducted after all enrolled subjects complete 24-week double-blind treatment and 1st stage database lock (DBL). The double-blind-period analysis will include data from safety observation period of subjects who complete double-blind treatment or are early withdrawn from double-blind treatment. Unblinding and statistical analysis will be performed accordingly. The analysis results will be delivered to NMPA for the application of new-drug registration. There is no need to adjust the type I error rate (α) or establish the Independent Database Monitoring Committee (IDMC) in this study.

3.2. Open-Label-Period Analysis and Full-Treatment-Period Analysis – When Entire Study Finishes

The open-label-period analysis and the full-treatment-period analysis will be conducted after all enrolled subjects completed full-treatment-period treatment and final DBL. The open-label-period analysis will include data from safety observation period of subjects who complete open-label treatment or are early withdrawn from open-label treatment. The full-treatment-period analysis will include data from safety observation period of subjects who complete full-treatment-period treatment or are early withdrawn from full-treatment-period treatment.

The final statistical analysis report (SAR) will be based on all study data including data from the double-blind treatment period and from the open-label treatment period. There's no multiplicity adjustment for each analysis.

4. Analysis Population

Table 1. Definition of Analysis Population

Population	Definition
Randomized Analysis Set (RAND)	➢ All randomized subjects<br>➢ Any subject, who has been successfully randomized, will be included
Safety Analysis Set (SS)	➢ All randomized subjects who received at least one dose of study medication<br>➢ A subset of RAND<br>➢ Analyses will be performed based on the actual treatments subjects received
Full Analysis Set (FAS)	➢ All randomized subjects who received at least one dose of study medication and had at least one post-treatment measurement of the primary efficacy endpoint on the basis of the intention-to-treat approach<br>➢ A subset of Safety Set<br>➢ Analyses will be performed based on the assigned treatments to which subjects are randomized
Per Protocol Set (PPS)	➢ All subjects without any major protocol deviations, which will be reviewed and determined at the BDR Meeting before DBL<br>➢ A subset of Full Analysis Set<br>➢ Analysis will be performed based on the assigned treatments to which subjects are randomized

4.1. Protocol Deviation

A listing of all protocol deviations will be provided. Protocol deviations, categorized into major protocol deviations and minor protocol deviations before unblinding, will be reviewed at the BDR Meeting. The analysis population will be determined at the BDR Meeting.

Subjects will be excluded from Per Protocol Set (PPS), when

1. Compliance in the double-blind treatment period is less than 80% or greater than 120%.
2. No valid HbA1c data at Week 24.

Major protocol deviations, which leads to exclusion from PPS, will be reviewed and determined at the BDR Meeting.

4.2. Incorrect Treatments

Under specific circumstance, the following rules will be applied.

1. If the actual treatment of any subject is different from the randomization treatment during the study, the efficacy analysis will be based on groups of randomization treatment from IWRS, according to the intention-to-treat approach.
2. If the actual treatment of any subject is different from the randomization treatment during the study, the safety analysis will be based on groups of actual treatment.
3. If any subjects who are not randomized took double-blind treatment, these subjects will be included in safety analysis based on groups of actual treatment, but not included in efficacy analysis.

5. General Conventions of Statistical Analysis.

Two efficacy and safety statistical analyses will be performed for the double-blind period and the full treatment period respectively.

For categorical variables, the number and percentage of subjects for each category are calculated. For continuous variables, the descriptive statistics such as sample size (n), mean, standard deviation (SD), median, 1st quartile, 3rd quartile, minimum, and maximum are provided.

Unless otherwise specified, the significance level for between-group comparison is set as 0.05 (2-sided).

Efficacy analysis will be conducted according to the intention-to-treat (ITT) approach. All randomized subjects will be included in the intended treatment group at randomization no matter what happens during the study (eg. drop-out or treated incorrectly).

For analyses involving different visits, data will be summarized by visit (See chapter 6.6 for detailed mapping rules). Data observed in all visits, including scheduled visit, unscheduled and end of treatment visit will be listed and sorted by treatment group, study center, subject ID and visit (if available).

5.1. Multicenter Study

HMM0302 is a multicenter study. Subjects from 73 centers will be enrolled competitively.

Number of subjects in each site will be evaluated before unblinding. If the number of subjects meeting FAS criteria within the site is less than 10, the site will be pooled with other sites by city and then by province until the number of subjects in the pooled site is more than 10. If the number of subjects within the pooled site is still less than 10, then those sites will be further pooled by geographic regions defined as below:

- Beijing, Tianjin
- Three Northeast Provinces, Inner Mongolia
- Shandong, Hebei, Anhui, Henan
- Shanghai, Zhejiang, Jiangsu
- Hubei, Hunan, Jiangxi
- Yunnan, Sichuan, Guizhou, Chongqing
- Shaanxi, Shanxi
- Guangdong, Guangxi, Hainan
- Qinghai, Ningxia

Unless otherwise specified, efficacy analysis results of FAS and PPS population will be summarized by pooled sites. Listing will be provided by the original sites.

5.2. Stratification Factors

IWRS system will be used to assign the randomization codes to each subject who meets randomization criteria based on stratification factors (duration of T2DM ≤ 3 years or >

3 years, and baseline HbA1c level ≤ 8.5% or > 8.5%) and the block size.
`

Unless otherwise specified, pooled site, duration of T2DM will be included in models for any study analyses. Categorization of duration of T2DM is based on the data collected at randomization. Baseline of other corresponding parameters will also be included in the specified analyses.

5.3. Multiplicity

There's only one primary efficacy endpoint and only one primary statistical comparison (Week 24). Efficacy endpoints in open-label treatment period (Week 52) will be analyzed only with descriptive statistics. Thus no type I error rate (α) adjustment will be applied.

5.4. Study Treatment Display Descriptors

The following study treatment description will be shown in the summary tables, listings and figures for double blind treatment period (from baseline to Week 24) analysis.

Treatment Group	Description
HMS5552 75mg BID combined with Metformin 1500 mg per day	HMS5552 75mg BID
Placebo BID combined with Metformin 1500 mg per day	Placebo

The following study treatment description will be shown in the summary tables, listings and figures for open-label treatment period and full treatment period (from baseline to the end of the entire study) analyses.

Treatment Group	Description
HMS5552 75mg BID combined with Metformin 1500 mg per day to HMS5552 75mg BID in combination with Metformin 1500 mg per day	HMS5552 - HMS5552
Placebo combined with Metformin 1500 mg per day crossover to HMS5552 75mg	Placebo - HMS5552

BID combined with Metformin 1500 mg per day	

Two efficacy and safety analyses will be performed for the double-blind period and the full treatment period respectively. Descriptive statistics, as well as result of hypothesis test, of demographic characteristics, efficacy and safety endpoints will be provided by treatment group and corresponding descriptions above.

6. General Conventions for Data Handling

6.1. Premature Withdraw and Missing Data

In FAS, missing data will be imputed by LOCF method for ANCOVA. Missing values will be imputed with the last non-missing available on-treatment assessment (including unscheduled visit). If categorical parameter is derived with quantitative parameters, the derivation will be conducted after missing data imputation of quantitative parameter is completed.

When month and day of birth date are missing, then "July" and "15th" will be used to impute the missing month and missing day respectively. Same imputation rule will be used to impute the missing initial diagnosis date of T2DM.

6.1.1. Imputation on Start / End Date of Adverse Events

Partial start/end dates for any adverse events will be imputed using the following convention.

For start date of adverse events,

1. Day Missing – impute with the first day of the month. If both month and year are the same as those of the first dose administration in the double-blind treatment period, then impute with the first dose administration date in the double-blind treatment period.
2. Day and Month Missing – impute with January 1st. If year is the same as that of the first dose administration in the double-blind treatment period, then impute with the first dose administration date in the double-blind treatment period.
3. Completely Missing - impute with the first dose administration date in the double-blind treatment period. If the end time of adverse event is earlier than the date of the first dose administration in the double-blind treatment period, then impute with the date of informed consent form.

For end date of adverse events,

1. Day Missing – impute with the last day of the month. If both month and year are the same as those of the date withdrawn from the study, then impute with the date withdrawn from the study.
2. Day and Month Missing – impute with December 31st. If year is the same as that of the date withdrawn from the study, then impute with the date withdrawn from the study.
3. Completely missing – if the adverse event is on-going, then no imputation will be applied. If the adverse event ends, then impute with the date withdrawn from the study. Furthermore, if the subject hasn't withdrawn from the study in the double-blind-period analysis, then impute with the cut-off date of the double-blind-period analysis.

Imputation for the start / end date of adverse events will be applied to determine treatment emergent adverse event only. It will not be used in other analyses, such as calculating the duration of adverse events.

For other adverse event data, such as relativity to study medication (except Metformin), and severity, no imputation will be applied to missing data.

6.1.2. Missing data in Start / End Date of Study Medications

When the date and time of the first dose administration in the double-blind treatment period is missing, the date and time of first study medication dispensation in the double-blind treatment period after randomization will be used to impute the missing date and time respectively. When the date of the last dose of study medication is missing, the date withdrawn from the study will be used to impute the missing date.

Since the date and time of the last dose of study medication in the double-blind treatment period for subjects who complete the double-blind treatment period, as well as the date and time of the first dose of study medication in the open-label treatment period for subjects who enter the open-label treatment period, are not directly collected by the case report form (CRF), the two time points are estimated, for the purpose of calculating dosing exposure duration and identifying whether any date and time of a subject belongs to the double-blind treatment period or the open-label treatment period. Since Visit 10 could last for multiple days, the date and time of the last dose of study medication in the double-blind treatment period for subjects who complete the double-blind treatment period is defined by 10: 00 on the last day of Visit 10, while the date and time of the first dose of study medication in the open-label treatment period for subjects who enter the open-label treatment period is defined by 18: 00 on the last day

of Visit 10.

6.1.3. Imputation on Start / End Date of Concomitant Medications

When the start date of concomitant medicine is missing, the following imputation will be applied.

1. Day Missing – impute with the first day of the month. If both month and year are the same as those of the first dose administration in double-blind treatment period, then impute with the first dose administration in double-blind treatment period.
2. Day and Month Missing – impute with January 1st. If year is the same as that of the first dose administration in double-blind treatment period, then impute with the first dose administration date in double-blind treatment period.
3. Completely Missing - impute with the first dose administration date in double-blind treatment period. If the end time of concomitant medicine is earlier than the first dose administration date in double-blind treatment period, then impute with the date of informed consent form. If the end date of concomitant medicine is earlier than date of informed consent form, then no imputation will be applied. This concomitant medicine will be determined as prior run-in concomitant medicine.

When the end date of concomitant medicine is missing, impute following below rules.

1. Day Missing – impute with the last day of the month. If both month and year are the same as those of the date withdrawn from the study, then impute with the date withdrawn from the study.
2. Day and Month Missing – impute with December 31st. If year is the same as that of the date withdrawn from the study, then impute with the date withdrawn from the study.
3. Completely Missing – if the concomitant medicine is on-going, then no imputation will be applied. If a concomitant medicine ends, then impute with the date withdrawn from the study. Furthermore, if the subject hasn't withdrawn from the study in the double-blind-period analysis, then impute with the cut-off date of the double-blind-period analysis.

Imputation for the start / end date of concomitant medicine will be applied to determine the concomitant medicine in prior run-in period, in run-in period, and during study treatment only. It will not be used in other analyses, such as calculating the duration of dosing.

6.2. Derivation and Conversion of Data

1 Duration of T2DM (Month) = (Date of ICF – Initial Diagnosis Date +1)/30.4375, presented with 1 decimal places (dp).

2 Diabetes treatment Days = End date – Start date + 1

3 Duration of medical history = End date – Start date + 1

4 Duration of adverse events (days) = End date – Start date + 1

5 For subjects followed up in the study, "planned dosage" stands for the planned total dose that should be taken in certain periods (e.g. run-in or treatment periods). If early withdrawal from the study occurs, the planned dosage stands for the total dose that should be taken from the beginning of that period to the last day of administration. Planned dosage is calculated by the first dose administration date and the last dose administration date in certain period. Planned dosage of run-in period, double-blind treatment period and open-label treatment period will be summarized respectively.

6 Actual dosing exposure: actual dosing duration (Day) * actually daily dose (pills / day),

$$Percentage\ of\ Drug\ Compliance = \frac{Actual\ Dosage}{Planned\ Dosage} \times 100\%.$$

Compliance of HMS5552 and placebo in run-in period, double-blind treatment period and open-label treatment period will be calculated respectively.

7 For continuous parameters in laboratory test, if the measurement is lower than the lower limit of detection, then impute with ½ of the lower limit of detection. If the measurement is higher than the upper limit of detective, then impute with 1.1 times that of upper limit of detective.

8 Function of β cell will be calculated using below formula,

Name	Formula
HOMA2-β	Calculated using HOMA2 Calculator
HOMA2-IR	Calculated using HOMA2 Calculator

URL for HOMA2 Calculator: https://www.dtu.ox.ac.uk/homacalculator/

6.3. Definition of Baseline and Change from Baseline

The baseline in double-blind treatment period is defined as the last non-missing value before the first dose administration in the double-blind treatment period. The baseline of open-label treatment period is defined as the last non-missing value before the first dose administration in the open-label treatment period. The baseline in full treatment

period is defined as the last non-missing value before the first dose administration in the double-blind treatment period. For continuous parameters, value of change from baseline is calculated by subtracting baseline value from post-baseline value. If either the baseline value or the post-baseline value is missing, then set change from baseline as missing.

Change from Baseline = Post-Baseline Value – Baseline Value

6.4. Multiple Measurement

For data of scheduled visit in summary analyses, if there are multiple values in a same scheduled visit, then the last one will be taken.

In listing, all values in scheduled and unscheduled visit will be displayed. If there are multiple values in one visit, then all will be displayed.

6.5. Data Display

Analysis datasets will be created according to CDISC standard, e.g. date and description in SDTM and ADaM will be displayed with ISO 8601 format (YYYY-MM-DD).

Listings will be classified by treatment group, site ID, subject ID and visit or date if appropriate。

All data will be processed, summarized and analyzed using SAS® Statistical Package Version 9.4 or a later release (Windows OS).

Following descriptive statistics will be presented in the summary table of continuous variables.

Table 2 Specification of Number of Decimal Places for Descriptive Statistics

Label	Description	No. of decimal places (dp)
N	Number of subjects in the treatment group	Always present to 0 dp
n	Number of subjects with non-missing values	Always present to 0 dp
Mean	Arithmetic Mean	1 dp more than raw data
SD	Standard Deviation	2 dp more than raw data
Median	Median	1 dp more than raw data
Q1	1st quartile	1 dp more than raw data
Q3	3rd quartile	1 dp more than raw data
Min	Minimum	Same as raw data
Max	Maximum	Same as raw data

Following statistical parameter will be presented in the summary table of statistical analysis.

Table 3 Specification of Number of Decimal Places for Statistical Parameters

Label	Description	No. of decimal places (dp)
LS Mean	Mean adjusted by treatment groups	1 dp more than raw data
Standard Error	Standard Error	2 dp more than raw data
Difference	Difference compared with placebo	1 dp more than raw data
95% CI	95% CI of difference/ratio between treatment groups	The same dp as difference or ratio
p-value	p-value	Usually 4dp (or＜0.001，or＞0.999）

The maximum decimal places (dp) is 4 for data displaying.

6.6. General rule of End-Of-Treatment Visit and Unscheduled Visit

For efficacy endpoint, if no related data at the scheduled visit is missing, then corresponding unscheduled visit will not be included in analyses. If there is related data at the scheduled visit missing with non-missing data at corresponding unscheduled visit, the data at the corresponding unscheduled visit will be used for missing data imputation (eg. LOCF). Data at the end-of-treatment visit of subjects early withdrawn from the study will be mapped to the nearest scheduled visit. Mapping is based on the following rules:

1. The gap between end-of-treatment visit and nearest scheduled visit <= 7 days
2. Date of drug discontinuation before end-of-treatment visit <= 7 days.

For safety endpoint, if data is missing at the scheduled visit followed by a consequent unscheduled visit, then the data at the nearest consequent unscheduled visit will be mapped to that corresponding scheduled visit (eg. LOCF). For the subjects who early withdrawn from the study, end of trial (EOT) data will be mapped to the next scheduled visit.

6.7. Prohibited Therapies and/or Medications

Safety assessment will not be influenced by exposure to prohibited medications.

If a subject has been exposed to other antidiabetic medications during the study, then all efficacy measurements after the first exposure to other antidiabetic medications will be set to missing. In sensitivity analyses, the measurements after the first exposure to other antidiabetic medications will be imputed by LOCF method using the last

measurement before the first exposure to other antidiabetic medications. For subjects discussed at the BDR Meeting to be included in PPS, this rule will not be applied.

6.8. Missing Efficacy Data due to Coronavirus Disease 2019 (COVID-19)

1 To keep conservative, according to the trend in mean HbA1c at Visit 9 and Visit 10, missing HbA1c data due to COVID-19 will be imputed using following rules:

For subjects with missing HbA1c due to COVID-19 at Visit 10 in Placebo group,

(1) If mean HbA1c at Visit 9 is less than that at Visit 10 within Placebo group, then missing HbA1c data at Visit 10 due to COVID-19 = Hba1c data at Visit 9

(2) If mean HbA1c at Visit 9 is greater than that at Visit 10 within Placebo group, then missing HbA1c data at Visit 10 due to COVID-19 = Hba1c data at Visit 9 + (mean HbA1c at Visit 10 within Placebo group - mean HbA1c at Visit 9 within Placebo group)

For subjects with missing HbA1c due to COVID-19 at Visit 10 in HMS5552 group,

(3) If mean HbA1c at Visit 9 is less than that at Visit 10 within HMS5552 group, then missing HbA1c data at Visit 10 due to COVID-19 = Hba1c data at Visit 9 + (mean HbA1c at Visit 10 within HMS5552 group - mean HbA1c at Visit 9 within HMS5552 group)

(4) If mean HbA1c at Visit 9 is greater than that at Visit 10 within HMS5552 group, then missing HbA1c data at Visit 10 due to COVID-19 = Hba1c data at Visit 9

Sensitivity analyses on efficacy endpoints based on the data imputed by rules mentioned above with the same model as the primary efficacy analyses will be conducted.

2 Sensitivity analyses on primary efficacy endpoint (change from baseline at Week 24) with corresponding statistical methods are conducted. Subgroup analyses with the same model as the primary efficacy analyses using FAS from which those subjects with missing data at Visit 10 due to COVID-19 are excluded will be conducted.

3 If there is any missing data at Visit 15 due to COVID-19, no imputation will be applied.

4 If any subjects are exposed to prohibited medications or other restricted medications during the epidemic of COVID-19, please refer to Section 6.7 for imputation rules.

5 For missing safety endpoint due to COVID-19, no imputation will be applied.

7. Study Population Analyses

7.1. Disposition of Subjects

Unless otherwise specified, disposition of randomized subjects will be summarized for each treatment group. Summarization will be performed for the double-blind treatment period and the full treatment period respectively.

The number of screened subjects, the number of subjects in run-in period, the number and percentage of each analysis set (RAND, SS, FAS and PPS) will be summarized. Subjects in each analysis set will be listed. The number and percentage of subjects who participate in open-label treatment period (for analysis of the entire study only) will be summarized.

The number and percentage of subjects who complete the study and who are early withdrawn from the study will be summarized by completion status and withdrawal reasons. The subjects early withdrawn from the study will be listed.

The number and percentage of subjects who are excluded from SS, FAS and PPS will be summarized respectively.

7.2. Demographic and Baseline Characteristics

Demographics will be summarized for RAND and SS respectively. Baseline characteristics will be summarized for RAND, FAS and PPS.

Demographic characteristics, medical history and concomitant diseases will be summarized based on RAND and SS by treatment group and for the entire population. Medical history and concomitant diseases will be summarized based on RAND. Baseline disease characteristics, baseline vital signs, baseline 12-lead ECG, baseline hematology, baseline chemistry and baseline routine urine test will be summarized based on RAND, FAS and PPS by treatment group and for the entire population. Demographic characteristics, medical history and concomitant diseases will be listed by subjects. Major and minor protocol deviations will be summarized based on RAND. Listings of major and minor protocol deviations will be provided.

For continuous variables, number, mean, standard deviation, median, minimum, maximum, 1st quantile and 3rd quantile will be calculated.

- Demographics includes age, height, weight, BMI, blood pressure, respiration, heart rate and body temperature.

- Baseline characteristics includes baseline HbA1c, bassline fasting plasma glucose, baseline 2-hour postprandial plasma glucose and duration of T2DM.

For categorical variables, the number and percentage of each category will be displayed.

- Demographics category includes age (≤65, >65), nationality, race, sex, BMI (<24，24-<28, ≥28).

- Baseline disease characteristics category includes HbA1c (≤8.5%, >8.5%), duration of T2DM (≤3 years, >3 years).

7.3. Exposure to Study Drug and Compliance

For study drug exposure (dose and days) and compliance, mean, standard deviation, minimum, maximum, median, 1st quantile and 3rd quantile will be calculated. The number and percentage of subject with poor compliance in each group (compliance < 80% or compliance > 120%) will be calculated.

7.4. Prior and Concomitant Medication

Concomitant treatment will be coded using the World Health Organization (WHO) Drug dictionary and be summarized by ATC level 4 and preferred term (PT). If ATC level 4 is missing, then it will be imputed with level 3. If ATC level 3 is missing, then it will be imputed with level2. Concomitant medications will be summarized for prior to run-in period, during run-in period and during treatment period. Concomitant medications will be summarized following rules below.

Definition 1: According to the start date of concomitant medication which is in prior to run-in period, during run-in period or during treatment period, the concomitant medication will be classified as concomitant medication in prior to run-in period, concomitant medication during run-in period and concomitant medication during treatment period.

Definition 2: Concomitant medications will be determined by the start/end time of concomitant medications. Once exposed to concomitant medications during prior to run-in period, run-in period or treatment period, then those concomitant medications will be summarized in corresponding periods. For example, if the start date and the end date of one concomitant medication are both in the run-in period， then the concomitant medication will only be summarized in the run-in period. If the start date of one concomitant medication is in the prior to run-in period, while the end date is in the treatment period, then the concomitant medication will be summarized in the prior to

run-in period, the run-in period and the treatment period.

When the start date and the end date of concomitant medications are missing, please refer to Section 6.1.3 for imputation rules.

Usage of all concomitant medications will be listed based on RAND.

8. Primary Statistical Analyses

8.1. Primary Efficacy Analyses

The primary endpoint for this study is the change of HbA1c from baseline at the completion of double-blind treatment period (Week 24). The primary analysis method will be Mixed Model for Repeated Measures (MMRM) without imputation for missing value using FAS population. This model includes the factors of treatment group, visit, the interaction of visit with treatment group, pooled site, duration of T2DM (≤3 years, >3 years) and baseline HbA1c. Treatment group, visit, interaction of visit with treatment group, pooled site, duration of T2DM and baseline HbA1c will be specified as the fixed effects. An unstructured variance-covariance structure will be used for the within-subject error. The Kenward and Roger method will be applied to estimate the degrees of freedom of the denominator. If the model failed to converge, Heterogeneous first-order autoregressive, ARH (1), covariance structures would be adopted. If the model still failed to converge, alternative correlation structures would be considered. The adjusted mean difference in the change in HbA1c from baseline at fixed time points between treatment groups will be estimated. The primary comparison is the difference in the change from baseline in HbA1c at the end of double-blind treatment period (Week 24) between treatment groups. The point estimate and 95% CI of mean difference between treatment groups (HMS5552 75mg BID + stable-dose Metformin vs. Placebo BID + stable-dose Metformin) will be calculated. The significance level of the between-group comparison is 0.05 (2-sided). If $p<0.05$, it suggests that the between-group difference is statistically significant. If the upper limit of the 95% CI is less than 0, it may be claimed that HMS5552 is superior to the placebo in treatment effect.

Below is the sample SAS code for MMRM:

```
proc mixed data=dataset;
class TREATMENT CENTRE VISIT PATIENT DIAB_HIS;
model CHG = TREATMENT VISIT TREATMENT*VISIT BASELINE DIAB_HIS
CENTRE / solution ddfm=kr;
repeated VISIT / subject=PATIENT type=un;
lsmeans TREATMENT*VISIT/ diff cl alpha=0.05;
run;
```

8.1.1. Sensitivity Analyses of Primary Efficacy endpoint

1. The same analysis for the primary endpoint will be performed for PPS as for FAS.
2. Primary efficacy analysis based on LOCF imputation.

Change from baseline in HbA1c when treatment completes (Week 24) will be described for each group. Analysis of covariance (ANCOVA) will be used to compare the change from baseline (LOCF) between treatment groups. The model includes treatment group, pooled site, duration of T2DM (≤3 years, >3 years) and baseline HbA1c. A superiority test between treatment group and placebo group will be performed. Based on the model in ANCOVA, the adjusted mean difference between treatment group and placebo group, as well as its 95% CI, will be estimated.

8.1.2. Supplementary Analyses of Primary Efficacy endpoint

A supplementary analysis will be the conducted. MMRM without imputation for missing value using FAS will be performed. This model includes the factors of treatment groups, visit, interaction of visit with treatment group, pooled site, interaction of pooled site with treatment group, duration of T2DM (≤3 years, >3 years) and baseline HbA1c. Treatment group, visit, interaction of visit with treatment group, pooled site, interaction of pooled site with treatment group, duration of T2DM and baseline HbA1c will be specified as the fixed effects. An unstructured variance-covariance structure will be used for the within-subject error. The Kenward and Roger method will be applied to estimate the degrees of freedom of the denominator. If the model failed to converge, Heterogeneous first-order autoregressive, ARH (1), covariance structures would be adopted. If the model still failed to converge, alternative correlation structures would be considered. The adjusted mean difference in the change in HbA1c from baseline at fixed time points between treatment groups will be estimated. The primary comparison is the difference in the change from baseline in HbA1c at the end of double-blind treatment period (Week 24) between treatment groups. The point estimate and 95% CI of mean difference between treatment groups (HMS5552 75mg BID + stable-dose Metformin vs. Placebo BID + stable-dose Metformin) will be calculated.

9. Secondary Statistical Analyses

9.1. Secondary Efficacy Analyses

Secondary efficacy analyses will be conducted for FAS and PPS.

9.1.1. Change in Baseline in Postprandial Plasma Glucose (PPG)

The same analysis method, MMRM, with the same model as the primary endpoint analysis will be conducted. The model includes the factors of treatment group, visit, interaction of visit with treatment group, pooled site, duration of T2DM (≤3 years, >3 years) and baseline 2-hour postprandial plasma glucose. The point estimate and 95% CI of mean difference between treatment groups (HMS5552 75mg BID + stable-dose Metformin vs. Placebo BID + stable-dose Metformin) will be calculated.

9.1.2. Change from Baseline in Fasting Plasma Glucose (FPG)

The same analysis method, MMRM, with the same model as the primary endpoint analysis will be conducted. The model includes the factors of treatment group, visit, interaction of visit with treatment group, pooled site, duration of T2DM (≤3 years, >3 years) and baseline fasting plasma glucose. The point estimate and 95% CI of mean difference between treatment groups (HMS5552 75mg BID + stable-dose Metformin vs. Placebo BID + stable-dose Metformin) will be calculated.

9.1.3. Hba1c Response Rate (proportion of subjects with HbA1c< 7%)

HbA1c Response Rate will be calculated on the basis of data imputed using LOCF method. The percentage pf subjects that with HbA1c < 7% at the end of double-blind treatment period (Week 24) will be summarized on the basis of data imputed using LOCF method. Odds ratio (OR) and its 95% CI between two treatment groups will be estimated using logistic regression method. The model includes the factors of treatment group, pooled site, interaction of pooled site with treatment group, duration of T2DM (≤3 years, >3 years) and baseline HbA1c. If the interaction is not significant (2-sided alpha 0.1), then remove the interaction of pooled site with treatment group from the model.

9.1.4. Change from Baseline in HbA1c at Each Visit (Except Week 24)

The same analysis method, MMRM, with the same model as the primary endpoint analysis will be conducted. The model includes the factors of treatment group, visit, interaction of visit with treatment group, pooled site, duration of T2DM (≤3 years, >3

years) and baseline HbA1c. The point estimate and 95% CI of mean difference between treatment groups (HMS5552 75mg BID + stable-dose Metformin vs. Placebo BID + stable-dose Metformin) will be calculated.

9.2. Other Efficacy Analyses

Other efficacy analyses will be performed based on FAS and PPS. Other efficacy endpoints include HOMA2-β and HOMA2-IR. Change from baseline in those endpoints will be summarized for each treatment group.

MMRM will be conducted on the change from baseline in HOMA2-β and HOMA2-IR respectively. The model includes the factors of treatment group, visit, pooled site, duration of T2DM (≤3 years, >3 years) and baseline value of corresponding endpoint.

9.3. Safety Analyses

Safety Analyses will be performed using Safety set.

9.3.1. Exposure of Subjects

Duration of exposure (days), actual dose and compliance of subjects will be summarized for the double-blind period and the open-label period respectively.

9.3.2. Adverse Events

AEs will be coded using MedDRA version 22.0.

AEs start before the first exposure to study medication in the double-blind treatment period will be included in the listing of all AEs.

Adverse events during screening visit, during the run-in period, and during the treatment period will be summarized respectively.

Adverse events during screening visit are defined as any AEs occur after signing ICF and before the first dose administration of study medication in the run-in period or any events already occur before signing ICF that worsened after the first dose administration of study medication in the run-in period.

Adverse events during the run-in period are defined as any AEs occur after the first dose administration of study medication in the run-in period and before the first dose

administration of study medication in the double-blind period or any events already occur before the first dose administration of study medication in the run-in period that worsened after the first dose administration of study medication in the double-blind period. Adverse events during the run-in period will be summarized by System Organ Class (SOC)/Preferred Term (PT).

Treatment emergent adverse events (TEAEs) in the double-blind period, TEAEs in the open-label period, and TEAEs in the full treatment period will be summarized respectively.

TEAEs in the double-blind treatment period are defined as any AEs occur after the first dose administration of study medication in the double-blind period and before the end of the double-blind period or any events already occur before the first dose administration of study medication in the double-blind period that worsened after the first dose administration of study medication in the double-blind period and before the end of the double-blind treatment period.

TEAEs in the open-label period are defined as any AEs occur after the first dose administration of study medication in the open-label period and before the end of the entire study or any events already occur before the first dose administration of study medication in the open-label period that worsened after the first dose administration of study medication in the open-label period and before the end of the entire study.

TEAEs in the full treatment period are defined as any AEs occur after the first dose administration of study medication in the double-blind period or any events already occur before the first dose administration of study medication in the double-blind period that worsened after the first dose administration of study medication in the double-blind period.

Treatment related Adverse events: AEs that "Related" or "Probably related" to study treatments.

Important adverse events: any AEs, except serious adverse events, leads to actions of medical treatment targeting on those AEs (such as dose discontinuation, dose reducing or symptomatic approach treatment) and to obviously abnormal results in hematological or other lab tests.

Serious adverse events (SAE) are defined as AEs that leads to one or more of following outcomes during any period (including the run-in period, the treatment period, and the safety observation period),

1) Death

2) Instant life-threatening
Life-threatening events are defined as the events that cause direct or instant death threatening to the subjects, excluding those events may cause death if they occur in a more serious degree. For example, a relieved drug-induced hepatitis that didn't lead to liver failure cannot be determined life-threatening, despite drug-induced hepatitis may be fatal.
3) Hospitalization or prolonged duration of hospitalization
For hospitalization or prolonged duration of hospitalization due to elective surgery, routine clinical procedures, social reasons, or self-convenience, no AE records are needed. However, if the event matches the definition above, such as an event causes existing disease to worsen during study, that event should still be reported as "serious" or "non-serious" adverse event by the regular rules described above.
4) Permanent or obvious disability or organ function impairment
5) Congenital malformations or birth defects
6) Important medical events, defined as events that may not cause death immediately, that are not life-threatening, that will not cause hospitalization, but may already endanger the subjects, or that medical intervention are needed to avoid situation mentioned above. For example, allergic bronchospasm on which emergency treatments are performed in the emergency room or at home, severe blood disease or convulsion that will not cause hospitalization, drug dependence, or drug abuse.

Incidence rate of AEs, imputed treatment related Adverse events, SAEs, AEs result in withdrawal from study, AEs result in discontinuation of study treatment will be summarized and counted by SOC/PT for each treatment group. If more than one AEs occur on a given subject, that subject will be counted for only once for the frequency of subjects who have AEs. If an AE occurs more than once on a given subject, that subject will be counted for only once for the frequency of that AE.

AEs will be summarized by severities. If an AE occurs more than once on a given subject, then the worst will be counted in the related analyses and summaries.

AEs with incidence rate≥1%, ≥2%, and ≥5% will be summarized by PT name.

9.3.3. Incidence Rate of Hypoglycemia Events

The hypoglycemia events will be classified by definition 1 (hypoglycemia events including serious hypoglycemia, and fingertip/venous hypoglycemia ≤3.9mmol/L), and definition 2 (hypoglycemia events including serious hypoglycemia, fingertip/venous hypoglycemia ≤3.9mmol/L, and probably symptomatic hypoglycemia and comparative

hypoglycemia).

The hypoglycemia events will be summarized for the entire study, the double-blind period, and the open-label period respectively. The number and percentage of subjects who have at least one hypoglycemia events, number of hypoglycemia events, and exposure-adjusted hypoglycemia incidence rate will be calculated for each treatment group by classification category described above. Moreover, hypoglycemia events will be summarized by the time of occurrence, week 0-4, week 4-8, week 8-16, week 16-24, week 24-28, week 28-34, week 34-40, > week 40. When hypoglycemia events during the open-label period are summarized, the incidence will be calculated based on number of subjects as denominator who have at least one dose administration of study medication in open-label period.

The hypoglycemia events will furthermore be classified by definition 3 (hypoglycemia events including fingertip/venous hypoglycemia ≤3.9mmol/L), and definition 4 (hypoglycemia events including fingertip/venous hypoglycemia ≤3.9mmol/L, and probably symptomatic hypoglycemia and comparative hypoglycemia).

The number and percentage of subjects who have at least one hypoglycemia events, number of hypoglycemia events, and exposure-adjusted hypoglycemia incidence rate will be calculated for each treatment group by classification category described above. Moreover, the number and percentage of hypoglycemia events will be calculated by the following sub-categories,

1. Hypoglycemia by severity (Serious hypoglycemia, Non-serious hypoglycemia)
2. Hypoglycemia relationship to study treatment except Metformin (Related, Not Related)
3. Hypoglycemia relationship to Metformin (Related, Not Related)
4. Hypoglycemia with cause (Induced, Non-induced)
5. Hypoglycemia by clinical diagnosis (Symptomatic hypoglycemia, Non-symptomatic hypoglycemia)
6. Time of hypoglycemia occurrence (week 0-4, week 4-8, week 8-16, week 16-24, week 24-28, week 28-34, week 34-40, and > week 40).

Exposure-adjusted hypoglycemia incidence rate of subjects = events number / exposure of total population (Year)

Listing of all related information for hypoglycemia events, including duration, glucose level when the hypoglycemia events start, clinical manifestations, severity, category, with or without treatment / intervention, outcome, cause, and relationship to study

treatment, will be provided.

9.3.4. Death

If any death occurs during the study after subjects signing ICF, the number of deaths will be reported, no matter whether the death is related to study medication or operation. Detailed description recorded in CRF including date of death, status of study medication, diagnosis, rescue process (if any records), cause, autopsy results (if any), will be listed.

9.3.5. Physical Examination

Physical examination results will be summarized for the entire study, the double-blind period, and the open-label period respectively. Normality / abnormality of test results (including general appearance, head and neck, chest (heart, lungs), abdomen, limbs, and others) at the end of study treatment will be tabulated. A listing with change from baseline in normality / abnormality at the end of treatment will be provided.

9.3.6. Vital Sign

Vital sign results will be summarized for the entire study, the double-blind period, and the open-label period respectively. Change from baseline in all vital signs (including body temperature (axillary temperature), systolic blood pressure, diastolic blood pressure, pulse, respiratory rate), height, weight and BMI at each visit will be described. Number, mean, standard deviation, median, minimum, maximum, 1st quantile and 3rd quantile will be calculated.

Change from baseline in weight and BMI will be analysed with LOCF using ANCOVA respectively. The model includes treatment group, pooled site, duration of T2DM (≤3 years, >3 years) and baseline value of corresponding parameters.

9.3.7. 12-lead ECG

The 12-lead ECG results will be summarized for the entire study, the double-blind period, and the open-label period respectively. Normality / abnormality of test results from baseline to post-treatment will be described. Subjects who are normal at baseline while abnormal with clinical significance after treatment or who are abnormal without clinical significance at baseline while abnormal with clinical significance after treatment will be listed. Number, mean, standard deviation, median, minimum, maximum, 1st quantile and 3rd quantile of change from baseline in corresponding parameters (including PR interval, QRS interval, QT interval, heart rate) at each visit

post-treatment will be calculated.

9.3.8. Clinical Lab Test

The clinical lab test results will be summarized for the entire study, the double-blind period, and the open-label period respectively. Lab test includes hematology parameters (including hemoglobin concentration, hematocrit, red blood cell count, white blood cell count, neutrophil count, basophil count, eosinophil count, monocyte count, lymphocyte count, platelet count), chemistries parameters (including urine pH, urine specific gravity, urine protein, urine sugar, urine red blood cells, urine white blood cells, ketone bodies) and routine urine parameters (including blood glucose, total bilirubin, alanine aminotransferase, aspartate aminotransferase, γ-glutamyltransferase, alkaline phosphatase, albumin, total protein, lactate dehydrogenase, urea nitrogen, Creatinine, uric acid, sodium, potassium, chloride, calcium, creatine kinase, creatine kinase isoenzyme, total cholesterol, high-density lipoprotein cholesterol, low-density lipoprotein cholesterol, triglycerides).

Number of subjects, mean, standard deviation, median, minimum, maximum, 1st quantile and 3rd quantile of continuous parameters will be calculated.

Number and percentage of categorical parameters will be summarized for each visit.

Normality / abnormality from baseline to post-treatment will be described on the basis of results and determination from central lab.

Hematology parameters, chemistries parameters and routine urine parameters (quantitative parameters) will be classified by marks. If the tests have been done, results marked as “H/HT/HP/L/LT/LP” will be treated as abnormal while results without mark will be treated as normal. For routine urine parameters (quantitative parameters), “Negative/Normal” will be treated as normal while “positive/Trace/+～++++” will be treated as abnormal.

9.3.9. HCG

HCG results (HCG position/negative/not done) will be summarized for each visit on Safety set.

10. Database Lock

Database lock (DBL) consists of two phases.

When all subjects finish double-blind period, 1st stage DBL will be performed. After the BDR Meeting, database will be locked by Principle Investigator, sponsor, and statistical team members after related documents are signed. Only data of the double-blind period will be locked, while data of the open-label period will not be locked. If any question found after the DBL, discussion on whether to unlock the database and to revise the data will be needed. The official records of the discussion will be preserved.

Final DBL will be performed after all subjects finish open-label period. Regular data review meeting (non-blind) will be conducted by Principle Investigator, sponsor, and statistical team members. After related documents are signed, the DBL will be performed. After the DBL, no more changes will be made to the database in principle. If any question found after the DBL, discussion on whether to unlock the database and to revise the data will be needed. The official records of the discussion will be preserved.

11. Reference

1. *HMMS5552 Add-on Protocol ：A 24-week multi-center, randomized, double-blind, placebo-controlled, Phase III study to evaluate the efficacy and safety of HMS5552 with additional 28-week open-label treatment to evaluate the safety of HMS5552 combined with Metformin in subjects with type 2 diabetes mellitus*
2. *Statistical Guidance for Clinical Trials 2016 CFDA*
3. *ICH E9：Statistical Principles for Clinical Trials 1998*
4. *https://www.dtu.ox.ac.uk/homacalculator/*
