# Supplementary material for: Dorzagliatin add-on therapy to metformin in patients with type 2 diabetes: a randomized, double-blind, placebo-controlled phase 3 trial
Source: Nat Med. 2022 May 12;28(5):974–81. doi: 10.1038/s41591-022-01803-5 (PMC9117147; doi:10.1038/s41591-022-01803-5)
Supplement: Supplementary file 2 — Reporting Summary [file 41591_2022_1803_MOESM2_ESM.pdf]

nature research

Corresponding author(s): Li Chen, Dalong Zhu, Xiaoying Li

Last updated by author(s): Mar 25, 2022

Reporting Summary

Nature Research wishes to improve the reproducibility of the work that we publish. This form provides structure for consistency and transparency in reporting. For further information on Nature Research policies, see our Editorial Policies and the Editorial Policy Checklist.

Statistics

For all statistical analyses, confirm that the following items are present in the figure legend, table legend, main text, or Methods section.

n/a	Confirmed	
☐	☒	The exact sample size (*n*) for each experimental group/condition, given as a discrete number and unit of measurement
☐	☒	A statement on whether measurements were taken from distinct samples or whether the same sample was measured repeatedly
☐	☒	The statistical test(s) used AND whether they are one- or two-sided<br>*Only common tests should be described solely by name; describe more complex techniques in the Methods section.*
☐	☒	A description of all covariates tested
☐	☒	A description of any assumptions or corrections, such as tests of normality and adjustment for multiple comparisons
☐	☒	A full description of the statistical parameters including central tendency (e.g. means) or other basic estimates (e.g. regression coefficient) AND variation (e.g. standard deviation) or associated estimates of uncertainty (e.g. confidence intervals)
☐	☒	For null hypothesis testing, the test statistic (e.g. F, t, r) with confidence intervals, effect sizes, degrees of freedom and P value noted<br>*Give P values as exact values whenever suitable.*
☒	☐	For Bayesian analysis, information on the choice of priors and Markov chain Monte Carlo settings
☒	☐	For hierarchical and complex designs, identification of the appropriate level for tests and full reporting of outcomes
☒	☐	Estimates of effect sizes (e.g. Cohen's *d*, Pearson's *r*), indicating how they were calculated

Our web collection on statistics for biologists contains articles on many of the points above.

Software and code

Policy information about availability of computer code

Data collection	Commercially available software Electronic Data Capture (EDC) (Medidata RAVE, Classic Rave version 2018.2.4) was used for clinical trial patient data collection. Randomization and drug dispensing were performed with an interactive web response system (IWRS) (Medidata RAVE RTSM 2020.3.2).
Data analysis	SAS software (SAS Institute Inc., Cary, N.C., version 9.4) was used for analyses. Data were computed using the HOMA2 Calculator version 2.2.3: https://www.dtu.ox.ac.uk/homacalculator/download.php.

For manuscripts utilizing custom algorithms or software that are central to the research but not yet described in published literature, software must be made available to editors and reviewers. We strongly encourage code deposition in a community repository (e.g. GitHub). See the Nature Research guidelines for submitting code & software for further information.

Data

Policy information about availability of data

All manuscripts must include a data availability statement. This statement should provide the following information, where applicable:

- Accession codes, unique identifiers, or web links for publicly available datasets
- A list of figures that have associated raw data
- A description of any restrictions on data availability

Data from these analyses in the DAWN study cannot be made publicly available due to the sponsor's contractual obligations. We encourage researchers or parties interested in collaboration for non-commercial use to apply to the corresponding author (lichen@huamedicine.com). Applications should outline specifically what data they are interested in receiving and how the data will be used; the use of data must also comply with the requirements of the Human Genetics Resources Administration of China and other country- or region-specific regulations. All data shared will be de-identified and will be made available 2 years after the date of

publication. A signed data access agreement with the sponsor is required before accessing the shared data. The study protocol and statistical analysis plan are provided with the paper.

Field-specific reporting

Please select the one below that is the best fit for your research. If you are not sure, read the appropriate sections before making your selection.

☒ Life sciences ☐ Behavioural & social sciences ☐ Ecological, evolutionary & environmental sciences

For a reference copy of the document with all sections, see nature.com/documents/nr-reporting-summary-flat.pdf

Life sciences study design

All studies must disclose on these points even when the disclosure is negative.

Sample size	We hypothesized that dorzagliatin would show superiority to placebo in decreasing HbA1c levels in patients after 24 weeks of treatment. For the primary endpoint HbA1c levels, we calculated that a total sample size of 750 patients would provide the trial with 95.4% power to detect a difference of 0.4% between the dorzagliatin and metformin group and the placebo and metformin group in a 1:1 ratio of allocation at a 2-sided significance level of 0.05, assuming a standard deviation (SD) of 1.5%.
Data exclusions	The full-analysis set included all randomized patients who took at least one dose of the study drug and had at least one posttreatment measurement of the primary endpoint during the double-blind treatment period (16 randomized patients were excluded from the full-analysis set: 15 owing to lack of efficacy assessments and 1 owing to no drugs taken after randomization).
Replication	This Phase 3 RCT replicates previous findings in a previously published controlled Phase 2 trial (Zhu D 2018).
Randomization	At week 3 of the 4-week run-in period, the patients were reevaluated before randomization to confirm eligibility. Eligibility criteria included an HbA1c level of 7.5 to 10.0% and an FPG level of 126.0 to 239.4 mg/dl at randomization. Diet and exercise counseling was provided throughout the experiment. Randomization and drug dispensing were performed with an interactive web response system (IWRS) (Medidata RAVE RTSM 2020.3.2). A stratified randomization method with the permuted block randomization algorithm was used. The blocks were dynamically allocated to each site and stratum from the randomization list. A unique ID number was provided by the vendor and marked on the drug box. Through central randomization, the randomization codes were generated by the IWRS system based on stratification factors (T2D disease duration≤3 years or >3 years and HbA1c level≤8.5% or >8.5% at randomization) and the block size.
Blinding	During the double-blind treatment, the random allocation sequences were concealed from the patients, investigators, and other study members until week 24. And the blinding was maintained during the whole 52 weeks of treatment. The placebo tablets had the same size, color, smell and appearance as the active drug tablets.

Behavioural & social sciences study design

All studies must disclose on these points even when the disclosure is negative.

Study description	*Briefly describe the study type including whether data are quantitative, qualitative, or mixed-methods (e.g. qualitative cross-sectional, quantitative experimental, mixed-methods case study).*
Research sample	*State the research sample (e.g. Harvard university undergraduates, villagers in rural India) and provide relevant demographic information (e.g. age, sex) and indicate whether the sample is representative. Provide a rationale for the study sample chosen. For studies involving existing datasets, please describe the dataset and source.*
Sampling strategy	*Describe the sampling procedure (e.g. random, snowball, stratified, convenience). Describe the statistical methods that were used to predetermine sample size OR if no sample-size calculation was performed, describe how sample sizes were chosen and provide a rationale for why these sample sizes are sufficient. For qualitative data, please indicate whether data saturation was considered, and what criteria were used to decide that no further sampling was needed.*
Data collection	*Provide details about the data collection procedure, including the instruments or devices used to record the data (e.g. pen and paper, computer, eye tracker, video or audio equipment) whether anyone was present besides the participant(s) and the researcher, and whether the researcher was blind to experimental condition and/or the study hypothesis during data collection.*
Timing	*Indicate the start and stop dates of data collection. If there is a gap between collection periods, state the dates for each sample cohort.*
Data exclusions	*If no data were excluded from the analyses, state so OR if data were excluded, provide the exact number of exclusions and the rationale behind them, indicating whether exclusion criteria were pre-established.*
Non-participation	*State how many participants dropped out/declined participation and the reason(s) given OR provide response rate OR state that no participants dropped out/declined participation.*
Randomization	*If participants were not allocated into experimental groups, state so OR describe how participants were allocated to groups, and if allocation was not random, describe how covariates were controlled.*

Ecological, evolutionary & environmental sciences study design

All studies must disclose on these points even when the disclosure is negative.

Study description	*Briefly describe the study. For quantitative data include treatment factors and interactions, design structure (e.g. factorial, nested, hierarchical), nature and number of experimental units and replicates.*
Research sample	*Describe the research sample (e.g. a group of tagged Passer domesticus, all Stenocereus thurberi within Organ Pipe Cactus National Monument), and provide a rationale for the sample choice. When relevant, describe the organism taxa, source, sex, age range and any manipulations. State what population the sample is meant to represent when applicable. For studies involving existing datasets, describe the data and its source.*
Sampling strategy	*Note the sampling procedure. Describe the statistical methods that were used to predetermine sample size OR if no sample-size calculation was performed, describe how sample sizes were chosen and provide a rationale for why these sample sizes are sufficient.*
Data collection	*Describe the data collection procedure, including who recorded the data and how.*
Timing and spatial scale	*Indicate the start and stop dates of data collection, noting the frequency and periodicity of sampling and providing a rationale for these choices. If there is a gap between collection periods, state the dates for each sample cohort. Specify the spatial scale from which the data are taken*
Data exclusions	*If no data were excluded from the analyses, state so OR if data were excluded, describe the exclusions and the rationale behind them, indicating whether exclusion criteria were pre-established.*
Reproducibility	*Describe the measures taken to verify the reproducibility of experimental findings. For each experiment, note whether any attempts to repeat the experiment failed OR state that all attempts to repeat the experiment were successful.*
Randomization	*Describe how samples/organisms/participants were allocated into groups. If allocation was not random, describe how covariates were controlled. If this is not relevant to your study, explain why.*
Blinding	*Describe the extent of blinding used during data acquisition and analysis. If blinding was not possible, describe why OR explain why blinding was not relevant to your study.*

Did the study involve field work? ☐ Yes ☐ No

Field work, collection and transport

Field conditions	*Describe the study conditions for field work, providing relevant parameters (e.g. temperature, rainfall).*
Location	*State the location of the sampling or experiment, providing relevant parameters (e.g. latitude and longitude, elevation, water depth).*
Access & import/export	*Describe the efforts you have made to access habitats and to collect and import/export your samples in a responsible manner and in compliance with local, national and international laws, noting any permits that were obtained (give the name of the issuing authority, the date of issue, and any identifying information).*
Disturbance	*Describe any disturbance caused by the study and how it was minimized.*

Reporting for specific materials, systems and methods

We require information from authors about some types of materials, experimental systems and methods used in many studies. Here, indicate whether each material, system or method listed is relevant to your study. If you are not sure if a list item applies to your research, read the appropriate section before selecting a response.

Materials & experimental systems

n/a	Involved in the study	
☒	☐	Antibodies
☒	☐	Eukaryotic cell lines
☒	☐	Palaeontology and archaeology
☒	☐	Animals and other organisms
☐	☒	Human research participants
☐	☒	Clinical data
☒	☐	Dual use research of concern

Methods

n/a	Involved in the study	
☒	☐	ChIP-seq
☒	☐	Flow cytometry
☒	☐	MRI-based neuroimaging

Antibodies

Antibodies used	*Describe all antibodies used in the study; as applicable, provide supplier name, catalog number, clone name, and lot number.*
Validation	*Describe the validation of each primary antibody for the species and application, noting any validation statements on the manufacturer's website, relevant citations, antibody profiles in online databases, or data provided in the manuscript.*

Eukaryotic cell lines

Policy information about cell lines

Cell line source(s)	*State the source of each cell line used.*
Authentication	*Describe the authentication procedures for each cell line used OR declare that none of the cell lines used were authenticated.*
Mycoplasma contamination	*Confirm that all cell lines tested negative for mycoplasma contamination OR describe the results of the testing for mycoplasma contamination OR declare that the cell lines were not tested for mycoplasma contamination.*
Commonly misidentified lines (See ICLAC register)	*Name any commonly misidentified cell lines used in the study and provide a rationale for their use.*

Palaeontology and Archaeology

Specimen provenance	*Provide provenance information for specimens and describe permits that were obtained for the work (including the name of the issuing authority, the date of issue, and any identifying information).*
Specimen deposition	*Indicate where the specimens have been deposited to permit free access by other researchers.*
Dating methods	*If new dates are provided, describe how they were obtained (e.g. collection, storage, sample pretreatment and measurement), where they were obtained (i.e. lab name), the calibration program and the protocol for quality assurance OR state that no new dates are provided.*

☐ Tick this box to confirm that the raw and calibrated dates are available in the paper or in Supplementary Information.

Ethics oversight	*Identify the organization(s) that approved or provided guidance on the study protocol, OR state that no ethical approval or guidance was required and explain why not.*

Note that full information on the approval of the study protocol must also be provided in the manuscript.

Animals and other organisms

Policy information about studies involving animals; ARRIVE guidelines recommended for reporting animal research

Laboratory animals	*For laboratory animals, report species, strain, sex and age OR state that the study did not involve laboratory animals.*
Wild animals	*Provide details on animals observed in or captured in the field; report species, sex and age where possible. Describe how animals were caught and transported and what happened to captive animals after the study (if killed, explain why and describe method; if released, say where and when) OR state that the study did not involve wild animals.*
Field-collected samples	*For laboratory work with field-collected samples, describe all relevant parameters such as housing, maintenance, temperature, photoperiod and end-of-experiment protocol OR state that the study did not involve samples collected from the field.*
Ethics oversight	*Identify the organization(s) that approved or provided guidance on the study protocol, OR state that no ethical approval or guidance was required and explain why not.*

Note that full information on the approval of the study protocol must also be provided in the manuscript.

Human research participants

Policy information about studies involving human research participants

Population characteristics	Among the 767 randomized patients, 475 (62%) were male and 292 (38%) were female. The average age of the study patients was 54.5±9.6 years, and the average disease duration was 71.5±55.8 months. The patients had an average body mass index (BMI) of 25.9±3.1 kg/m2 and a mean HbA1c value of 8.3±0.6% at baseline. The demographic and baseline characteristics were similar between the two groups (Table 1).
Recruitment	Patients were recruited at the study site by investigators. The patients were required to sign an informed consent form prior to participating in any study procedures. Patients were recruited from October 11, 2017, to August 30, 2019, and the last patient visit was conducted on August 31, 2020. Of the 1721 patients screened, 767 eligible patients were randomly assigned to one of the two treatment groups; 382 patients were assigned to receive dorzagliatin (75 mg twice a day) and metformin (1500 mg daily), and 385 patients were assigned to receive placebo and metformin (1500 mg daily) (Figure 1). In total, 766

	patients took at least one dose of the study drug and were included in the safety analysis set; 751 patients completed at least one post-randomization measurement and were included in the full-analysis set (FAS).
Ethics oversight	The trial was conducted in accordance with the principles of the Declaration of Helsinki, Good Clinical Practice (GCP) guidelines, and laws and regulations in China. The study protocol was amended once during the study. The important changes to the protocol, which mainly involves exclusion criteria and randomization criteria are listed as follows: adjusted to fasting C-peptide <0.81 ng/ml (0.27 nmol/L) at screening; short-term external use of corticosteroids treatment within 1 year at screening was permissible; inhaled glucocorticoid treatment was unacceptable; stable coronary heart disease was allowed; exclusion and randomization criteria related to blood pressure and antihypertensive drugs were redefined as systolic blood pressure ≥ 160 mmHg or diastolic blood pressure ≥ 100 mmHg at screening, or who added/changed antihypertensive drugs or adjusted dose within 4 weeks before screening; 12-lead electrocardiogram was added to Visit 3 as a pre-randomization criterion to ensure smooth conduct of the study; a visit window period of ± 3 days for Visit 2 was added to match actual operational needs; clarified that all blood samples were collected in fasting status except for MMTT 30-min and 120-min; 30-minute (±3 minutes were allowed) testing point for the MMTT was added to optimize the study protocol; clarified the blood sampling time (MMTT 120 min) for pharmacokinetics was before taking study medications; the rule that patients need not to be in fasting status at Visit 2 was removed for compliance. Minor protocol revisions involve wording, consistency, and accuracy. The trial protocol and amendments were approved by the local ethics committees of all study sites: China-Japan Friendship Hospital, Beijing, China; Affiliated Drum Tower Hospital, Medical School of Nanjing University, Nanjing, China; The First People's Hospital of Changde City, Changde, China; Central Hospital Affiliated to Shandong First Medical University, Jinan, China; Cangzhou People's Hospital, Cangzhou, China; Peking Union Medical College Hospital, Beijing, China; The First Affiliated Hospital of Henan University of Science and Technology, Luoyang, China; The Affiliated Hospital of Qingdao University, Qingdao, China; Qingdao Central Hospital, Qingdao, China; Chenzhou First People's Hospital, Chenzhou, China; The Second Affiliated Hospital of Nanjing Medical University, Nanjing, China; Tongji Hospital of Tongji University, Shanghai, China; The First Affiliated Hospital of Kunming Medical University, Kunming, China; The First Bethune Hospital of Jilin University, Changchun, China; Huzhou Central Hospital, Huzhou, China; The Affiliated Hospital of Jiangsu University, Zhenjiang, China; Nanjing First Hospital, Nanjing, China;The Affiliated Hospital of Xuzhou Medical University, Xuzhou, China; Inner Mongolia Baogang Hospital, Baotou, China; The First People's Hospital of Yue Yang, Yueyang, China; Jingzhou Hospital Affiliated to Yangtze University, Jingzhou, China; Jiangxi Pingxiang People's Hospital, Pingxiang, China; Jiangxi Provincial People's Hospital, Nanchang, China; The First People's Hospital of Shunde, Foshan, China;Chongqing General Hospital, Chongqing, China; Qinghai University Affiliated Hospital, Xining, China; Luoyang Central Hospital, Luoyang, China; General Hospital of Ningxia Medical University, Yinchuan, China; The General Hospital of Xuzhou City Mining Group, Xuzhou, China; Shanghai Pudong New Area People's Hospital, Shanghai, China; Beijing Pinggu Hospital, Beijing, China; Xinhua Hospital Affiliated to Shanghai Jiao Tong University School of Medicine, Shanghai, China; Baotou Central Hospital, Baotou, China; West China Hospital of Sichuan University, Chengdu, China; The 960th Hospital of the PLA Joint Logistics Support Force, Jinan, China; Yiyang Central Hospital, Yiyang, China; Yangpu Hospital, Tongji University, Shanghai, China; The First Affiliated Hospital of Anhui Medical University, Hefei, China; Taihe Hospital, Shiyan, China; The First Hospital of China Medical University, Shenyang, China; Southern Medical University Nanfang Hospital, Guangzhou, China; The Central Hospital of Wuhan, Wuhan, China; The Affiliated Hospital of Guizhou Medical University, Guiyang, China; Anhui Provincial Hospital, Hefei, China; The First Affiliated Hospital of Bengbu Medical College, Bengbu, China; Huadong Hospital Affiliated to Fudan University, Shanghai, China; Jiangsu Province Hospital, Nanjing, China; Jining No. 1 People's Hospital, Jining, China; Tianjin People's Hospital, Tianjin, China; The First Hospital of Qiqihar, Qiqihar, China; Jilin Central General Hospital, Jilin, China; Third People's Hospital of Yunnan Province, Kunming, China; Peking University First Hospital, Beijing, China; Ningbo First Hospital, Ningbo, China; The Affiliated Huai'an No. 1 People's Hospital of Nanjing Medical University, Huai'an, China; Shanghai Tenth People's Hospital, Shanghai, China; Emergency General Hospital, Beijing, China; The First Affiliated Hospital of Hainan Medical University, Haikou, China; Peking University Shougang Hospital, Beijing, China; Qinghai Provincial People's Hospital, Xining, China; Hainan General Hospital, Haikou, China; Liuzhou People's Hospital, Liuzhou, China; The First Affiliated Hospital of Wannan Medical University, Wuhu, China; The Second People's Hospital of Huai'an, Huai'an, China; Jiangsu Taizhou People's Hospital, Taizhou, China; Zhejiang Hospital, Hangzhou, China; Aerospace Center Hospital, Beijing, China; Yanbian University Hospital, Yanji, China; The Third Affiliated Hospital of Southern Medical University, Guangzhou, China; Beijing Luhe Hospital Affiliated to Capital Medical University, Beijing, China; Shanghai East Hospital, Tongji University, Shanghai, China; Zhongshan Hospital, Fudan University, Shanghai, China. All patients provided written informed consent before trial entry. The trial was conducted in accordance with the Chinese Diabetes Society guidelines, which require physicians to educate and strictly enforce improved exercise and dietary control and self-monitoring of blood glucose levels (at least two times/week) while treating patients with T2D.

Note that full information on the approval of the study protocol must also be provided in the manuscript.

Clinical data

Policy information about clinical studies

All manuscripts should comply with the ICMJE guidelines for publication of clinical research and a completed CONSORT checklist must be included with all submissions.

Clinical trial registration	NCT03141073
Study protocol	Refer to the supplemental material
Data collection	All laboratory tests were conducted in the central laboratory. All samples were collected at the site.
Outcomes	The primary efficacy endpoint was the change in HbA1c level from baseline to week 24. Key secondary efficacy endpoints included the changes in 2h-PPG and FPG levels from baseline to week 24, HbA1c level at each visit (except week 24), and the percentage of patients who reached an HbA1c level of less than 7.0% at week 24. Additional efficacy endpoints included the changes in the HOMA2-β (an index of β-cell function) and HOMA2-IR (an index of insulin resistance) from baseline. A complete list of other efficacy endpoints is provided in the statistical analysis plan as the supplemental material. During the study, HbA1c and FPG levels were measured and evaluated at each visit from visits 4 to 15. At visits 4, 7, 10, and 15, 2h-PPG levels were measured using a standardized mixed-meal tolerance test (MMTT). Safety assessments were performed at each study visit. Blood and urine samples were collected

at each visit. Adverse events and serious adverse events were assessed throughout the trial. Hypoglycemic episodes were classified according to the American Diabetes Association (ADA) definitions. Vital signs and clinical laboratory test results were assessed, and physical examinations were performed.

Dual use research of concern

Policy information about dual use research of concern

Hazards

Could the accidental, deliberate or reckless misuse of agents or technologies generated in the work, or the application of information presented in the manuscript, pose a threat to:

No	Yes	
☐	☐	Public health
☐	☐	National security
☐	☐	Crops and/or livestock
☐	☐	Ecosystems
☐	☐	Any other significant area

Experiments of concern

Does the work involve any of these experiments of concern:

No	Yes	
☐	☐	Demonstrate how to render a vaccine ineffective
☐	☐	Confer resistance to therapeutically useful antibiotics or antiviral agents
☐	☐	Enhance the virulence of a pathogen or render a nonpathogen virulent
☐	☐	Increase transmissibility of a pathogen
☐	☐	Alter the host range of a pathogen
☐	☐	Enable evasion of diagnostic/detection modalities
☐	☐	Enable the weaponization of a biological agent or toxin
☐	☐	Any other potentially harmful combination of experiments and agents

ChIP-seq

Data deposition

☐ Confirm that both raw and final processed data have been deposited in a public database such as GEO.

☐ Confirm that you have deposited or provided access to graph files (e.g. BED files) for the called peaks.

Data access links<br>*May remain private before publication.*	*For "Initial submission" or "Revised version" documents, provide reviewer access links. For your "Final submission" document, provide a link to the deposited data.*
Files in database submission	*Provide a list of all files available in the database submission.*
Genome browser session<br>(e.g. UCSC)	*Provide a link to an anonymized genome browser session for "Initial submission" and "Revised version" documents only, to enable peer review. Write "no longer applicable" for "Final submission" documents.*

Methodology

Replicates	*Describe the experimental replicates, specifying number, type and replicate agreement.*
Sequencing depth	*Describe the sequencing depth for each experiment, providing the total number of reads, uniquely mapped reads, length of reads and whether they were paired- or single-end.*
Antibodies	*Describe the antibodies used for the ChIP-seq experiments; as applicable, provide supplier name, catalog number, clone name, and lot number.*
Peak calling parameters	*Specify the command line program and parameters used for read mapping and peak calling, including the ChIP, control and index files used.*
Data quality	*Describe the methods used to ensure data quality in full detail, including how many peaks are at FDR 5% and above 5-fold enrichment.*
Software	*Describe the software used to collect and analyze the ChIP-seq data. For custom code that has been deposited into a community*

Software	*repository, provide accession details.*

Flow Cytometry

Plots

Confirm that:

- [ ] The axis labels state the marker and fluorochrome used (e.g. CD4-FITC).
- [ ] The axis scales are clearly visible. Include numbers along axes only for bottom left plot of group (a 'group' is an analysis of identical markers).
- [ ] All plots are contour plots with outliers or pseudocolor plots.
- [ ] A numerical value for number of cells or percentage (with statistics) is provided.

Methodology

Sample preparation	*Describe the sample preparation, detailing the biological source of the cells and any tissue processing steps used.*
Instrument	*Identify the instrument used for data collection, specifying make and model number.*
Software	*Describe the software used to collect and analyze the flow cytometry data. For custom code that has been deposited into a community repository, provide accession details.*
Cell population abundance	*Describe the abundance of the relevant cell populations within post-sort fractions, providing details on the purity of the samples and how it was determined.*
Gating strategy	*Describe the gating strategy used for all relevant experiments, specifying the preliminary FSC/SSC gates of the starting cell population, indicating where boundaries between "positive" and "negative" staining cell populations are defined.*

- [ ] Tick this box to confirm that a figure exemplifying the gating strategy is provided in the Supplementary Information.

Magnetic resonance imaging

Experimental design

Design type	*Indicate task or resting state; event-related or block design.*
Design specifications	*Specify the number of blocks, trials or experimental units per session and/or subject, and specify the length of each trial or block (if trials are blocked) and interval between trials.*
Behavioral performance measures	*State number and/or type of variables recorded (e.g. correct button press, response time) and what statistics were used to establish that the subjects were performing the task as expected (e.g. mean, range, and/or standard deviation across subjects).*

Acquisition

Imaging type(s)	*Specify: functional, structural, diffusion, perfusion.*
Field strength	*Specify in Tesla*
Sequence & imaging parameters	*Specify the pulse sequence type (gradient echo, spin echo, etc.), imaging type (EPI, spiral, etc.), field of view, matrix size, slice thickness, orientation and TE/TR/flip angle.*
Area of acquisition	*State whether a whole brain scan was used OR define the area of acquisition, describing how the region was determined.*

Diffusion MRI ☐ Used ☐ Not used

Preprocessing

Preprocessing software	*Provide detail on software version and revision number and on specific parameters (model/functions, brain extraction, segmentation, smoothing kernel size, etc.).*
Normalization	*If data were normalized/standardized, describe the approach(es): specify linear or non-linear and define image types used for transformation OR indicate that data were not normalized and explain rationale for lack of normalization.*
Normalization template	*Describe the template used for normalization/transformation, specifying subject space or group standardized space (e.g. original Talairach, MNI305, ICBM152) OR indicate that the data were not normalized.*
Noise and artifact removal	*Describe your procedure(s) for artifact and structured noise removal, specifying motion parameters, tissue signals and physiological signals (heart rate, respiration).*

Volume censoring | *Define your software and/or method and criteria for volume censoring, and state the extent of such censoring.*

Statistical modeling & inference

Model type and settings | *Specify type (mass univariate, multivariate, RSA, predictive, etc.) and describe essential details of the model at the first and second levels (e.g. fixed, random or mixed effects; drift or auto-correlation).*

Effect(s) tested | *Define precise effect in terms of the task or stimulus conditions instead of psychological concepts and indicate whether ANOVA or factorial designs were used.*

Specify type of analysis: ☐ Whole brain ☐ ROI-based ☐ Both

Statistic type for inference
(See Eklund et al. 2016) | *Specify voxel-wise or cluster-wise and report all relevant parameters for cluster-wise methods.*

Correction | *Describe the type of correction and how it is obtained for multiple comparisons (e.g. FWE, FDR, permutation or Monte Carlo).*

Models & analysis

n/a	Involved in the study
☐	☐ Functional and/or effective connectivity
☐	☐ Graph analysis
☐	☐ Multivariate modeling or predictive analysis

Functional and/or effective connectivity | *Report the measures of dependence used and the model details (e.g. Pearson correlation, partial correlation, mutual information).*

Graph analysis | *Report the dependent variable and connectivity measure, specifying weighted graph or binarized graph, subject- or group-level, and the global and/or node summaries used (e.g. clustering coefficient, efficiency, etc.).*

Multivariate modeling and predictive analysis | *Specify independent variables, features extraction and dimension reduction, model, training and evaluation metrics.*
